# Sydnone Methides—A Forgotten Class of Mesoionic Compounds for the Generation of Anionic N‐Heterocyclic Carbenes

**DOI:** 10.1002/anie.202107495

**Published:** 2021-07-14

**Authors:** Sebastian Mummel, Felix Lederle, Eike G. Hübner, Jan C. Namyslo, Martin Nieger, Andreas Schmidt

**Affiliations:** ^1^ Clausthal University of Technology Institute of Organic Chemistry Leibnizstrasse 6 D-38678 Clausthal-Zellerfeld Germany; ^2^ Fraunhofer Heinrich Hertz Institute HHI Fiber Optical Sensor Systems Am Stollen 19H D-38640 Goslar Germany; ^3^ University of Helsinki Department of Chemistry P.O. Box 55 FIN-00014 Helsinki Finland

**Keywords:** betaines, carbenes, heterocycles, rhodium, selenium

## Abstract

Sydnone methides are described from which only one single example has been mentioned in the literature so far. Their deprotonation gave anions which can be formulated as π‐electron rich anionic N‐heterocyclic carbenes. Sulfur and selenium adducts were stabilized as their methyl ethers, and mercury, gold as well as rhodium complexes of the sydnone methide carbenes were prepared. Sydnone methide anions also undergo C−C coupling reactions with 1‐fluoro‐4‐iodobenzene under Pd(PPh_3_)_4_ and CuBr catalysis. ^77^Se NMR resonance frequencies and ^1^ *J*_C4‐Se_ as well as ^1^ *J*_C4‐H_ coupling constants have been determined to gain knowledge about the electronic properties of the anionic N‐heterocyclic carbenes. The carbene carbon atom of the sydnone methide anion **3 j** resonates at *δ*=155.2 ppm in ^13^C NMR spectroscopy at −40 °C which is extremely shifted upfield in comparison to classical N‐heterocyclic carbenes.

## Graphical abstract

Except for a single example, sydnone methides have been unknown to date. Syntheses are described here, leading to 10 stable and intensely colorful, slightly fluorescent sydnone methides with Z=CN, COOMe, and SO_2_Me, respectively. On deprotonation, unique anionic π‐electron‐rich N‐heterocyclic carbenes are formed which react to thioethers and selenoethers, mercury, gold and rhodium complexes, and 4‐acylsydnone methide on treatment with acyl chlorides.

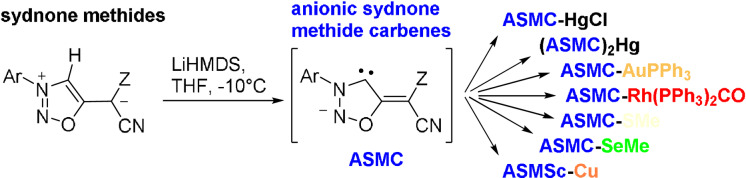

Undoubtedly, sydnones are the best known mesoionic compounds. They are widely applied as masked nitrile imines in 1,3‐dipolar cycloadditions (“Huisgen reactions”^[1]^) which proceed in a copper‐catalyzed,^[2]^ metal‐free^[3]^ or metal‐free strain‐promoted click fashion[^4^, ^5^] with sub‐millisecond intermediates,^[6]^ as summarized in numerous review articles and compilations about [2+3]‐cycloadditions.^[7]^ Recent developments include the synergistic combination of organocatalysis and visible‐light photocatalysis of cycloadditions of sydnones to form pyrazoles.^[8]^ Apart from cycloadditions, sydnones are versatile precursors for nucleophilic radiofluorination for the synthesis of [^18^F]fluoroarenes,^[9]^ and co‐catalysts or ligands of Pd catalysts for Suzuki–Miyaura reactions under acidic conditions.^[10]^ They are of ongoing interest as biologically active compounds^[11]^ from which the sydnone imine^[12]^ Molsidomine^[13]^ and Sydnocarb^[14]^ are in clinical use.^[15]^ Recently they have been applied for in vitro bioconjugation of purified proteins,^[5]^ as sialic acid‐substituted bioorthogonal reporters,^[4]^ as imaging reagents,^[16]^ labeling and imaging tools in live cells,^[17]^ and fluorogenic compounds.^[18]^ In view of this remarkable career it is surprising that some members of the sydnone family of compounds (Scheme 1) have remained almost unknown to date. Thus, only one sydnone methide (sydnone methanide) (**2 a**) has been prepared,^[19]^ examined by ^17^O,^[20]^ ^15^N, and ^14^N NMR spectroscopy,^[21]^ and repeatedly mentioned in monographs.^[15]^

**Scheme 1:**
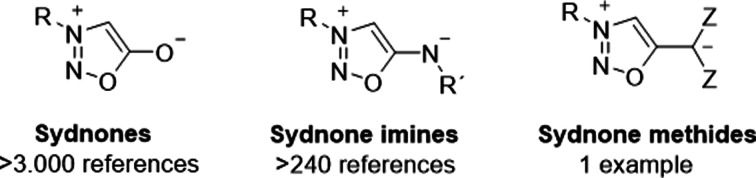
Some members of the sydnone family of compounds.

Our interest in mesomeric betaines^[22]^ and their relationship to neutral[^12a^, ^12b^, ^23^, ^24^] as well as anionic N‐heterocyclic carbenes^[25]^ in combination with the introduction of an energy‐based quantitative index of the ease of NHC formation from precursors by deprotonation (carbene relative energy of formation, CREF)^[26]^ stimulated initial calculations which suggested that sydnone methides are interesting precursors for the generation of unique π‐electron‐rich anionic N‐heterocyclic carbenes. This index supplements other measures of σ‐donor and/or π‐properties of NHCs such as molecular electrostatic potentials (MESP),^[27]^ computationally derived ligand electronic parameters (CEP),^[28]^ Tolman parameters (TEP),^[29]^ HOMO energies,[^30^, ^31^] calculated proton affinities,[^30^, ^32^] ^1^ *J*_CH_ and ^1^ *J*_CSe_ coupling constants^[33]^ of precursor salts and Se adducts, respectively, and Huynh electronic parameters (HEP)^[34]^ of Pd complexes (review^[35]^). We calculated the CREF value of sydnone methide **2 a** to be 0.534 (B3LYP/6‐311++G**) which seemed to be a promising value in comparison to other mesomeric betaines as carbene precursors like 1,3‐dimethylimidazolium‐4‐olate and its 4‐aminide, and 1,3‐dimethyl‐6‐oxo‐pyrimidinium‐4‐olate (CREFs=0.576, 0.557, 0.547, resp.).[^24^, ^25^] We therefore report here on the syntheses of a series of sydnone methides, their conversion into anionic NHCs by deprotonation, and trapping reactions with sulfur, selenium, C‐electrophiles, mercury, gold, and rhodium as well as C−C coupling reactions under Pd^0^/Cu^I^‐catalysis.

We first developed a reliable synthetic method which lead to a series of sydnone methides **2 a**–**l** (Scheme 2, Table 1). Thus, we started from the sydnones **1 a**–**l** which we treated with Tf_2_O to obtain a mixture of inseparable bis‐sydnone ethers and sydnone 5‐triflates which proved to be very sensitive towards minute traces of water. Trapping with in situ generated malodinitrile anions for the synthesis of **2 b**–**d**, methyl 2‐cyanoacetate anions for **2 e**–**h**, and 2‐(methylsulfonyl)acetonitrile anions for the preparation of **2 i**–**l** gave the desired sydnone methides, respectively. These compounds are stable, brilliant yellow to orange in color and slightly fluorescent (see Supporting Information for fluorescence spectra). Sydnone methides can be represented by several resonance structures three of which are shown in Scheme 2. It is interesting to note that carbon atom C4 is a site of negative charge according to the rules of resonance as indicated by mesomeric structure **2 a**–**lB**, although it is a site of deprotonation for the formation of anionic N‐heterocyclic carbenes. Correspondingly, the C4 carbon atoms resonate at high field between *δ*=108.2 ppm (**2 j**) and 110.9 ppm (**2 f**) in ^13^C NMR spectroscopy. Further DFT calculations predict that the *E* isomer of **2 e** is by Δ*G*_vac_=4.6 kJ mol^−1^ more stable in vacuo than the corresponding *Z* isomer and that the rotation barrier is Δ*E*_vac_ *=*82.7 kJ mol^−1^ (PBE0‐d3/ 6‐31G**). We also calculated the solvent dependence. In THF, dichloromethane (DCM) and DMSO the rotation barriers decrease to Δ*E*_THF_=65.8 kJ mol^−1^, Δ*E*_DCM_=65.0 kJ mol^−1^, and Δ*E*_DMSO_=61.6 kJ mol^−1^, respectively (see Supporting Information). These results are in agreement with the fact that the bonds C5‐C6 of **2 e** and **2 i** [crystallographic numbering, Figure 1] display a considerable double bond character, as determined by single crystal X‐ray analyses. As N2 resonates considerably more upfield in ^15^N NMR spectroscopy than N3, the mesomeric structures **2 a**‐**lC** can be identified as the most suitable representation for sydnone methides. In the elemental cells, the phenyl ring is twisted by −52.65(17)° (**2 e**) and −12.74(17)° (**2 i**) out of the plane of the sydnone rings. As determined by HMBC measurements, the ^1^ *J*_C4‐H_ coupling constants of **2 a,e,i** are 214 Hz, 220 Hz, and 219 Hz, respectively, and these values are between those of 1,3‐dimesitylimidazolium and 1,3‐dimesitylimidazolidinium (^1^ *J*_CH_=225 Hz and 206 Hz) as precursors of normal N‐heterocyclic carbenes, respectively, thus indicating strong σ‐donor capacities of the corresponding N‐heterocyclic carbenes.^[33]^ We next performed a base screening which revealed that deprotonation of the sydnone methides **2 a**–**l** can best be accomplished by LiHMDS in THF at −10 °C. According to the rules of resonance, the resulting anions **3 a**–**l** can be represented by a number of canonical forms, among those the mesomeric structures of abnormal N‐heterocyclic carbenes **3 a**‐**lA**, as structures possessing two formal negative charges at C4 **3 a**‐**lB**, and as normal anionic N‐heterocyclic carbenes **3 a**‐**lC** (Scheme 2).

**Figure 1:**
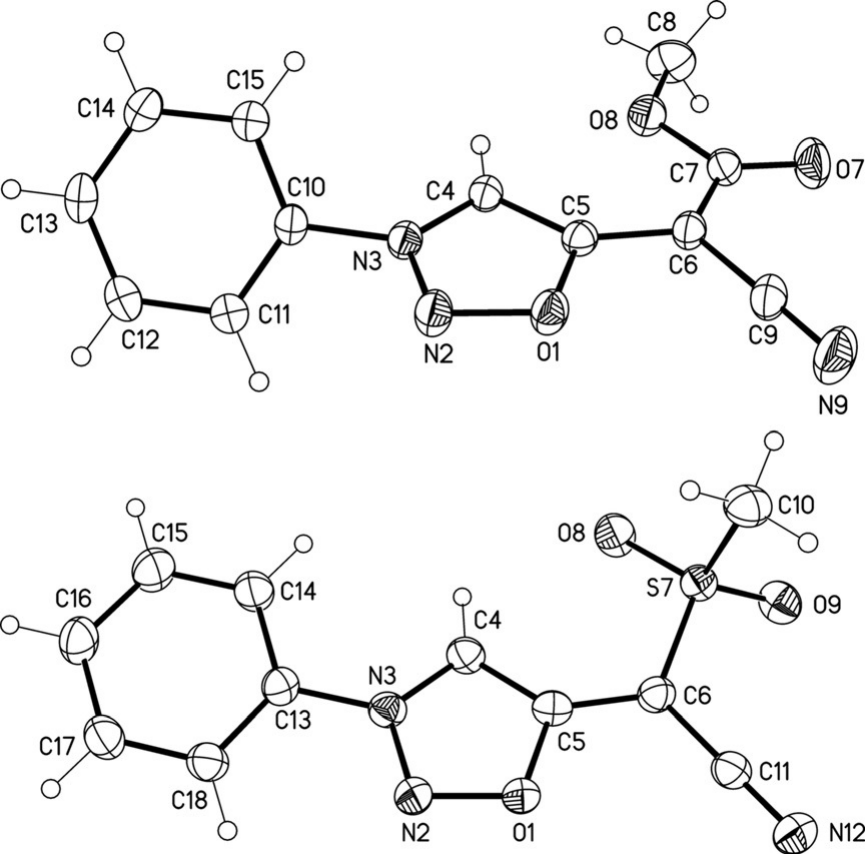
Molecular drawing of sydnone methide **2 e** (above) and **2 i** (below) (displacement parameters are drawn at 30 % (**2 e**) and 50 % (**2 i**) probability level). Selected bond lengths of **2 e** [pm] (crystallographic numbering): N3–C4: 134.12(15), C4‐C5: 138.91(15), C5–C6: 139.87(16), C6–C7: 143.83(17), C7–O7: 121.05(15), C9–N9: 114.51(18) pm. Selected torsion angles of **2 e** [°]: N2‐N3‐C10‐C15: 128.96(13), C4‐C5‐C6‐C7: −1.8(2) °. Selected bond lengths of **2 i** [pm]: N3–C4: 135.00(17), C4–C5: 138.10(17), C5–C6: 139.80(18), C6–S7: 172.16(13), S7–O9: 144.04(10), C11–N12: 115.02(18) pm. Selected torsion angles of **2 i** [°]: N2‐N3‐C13‐C18: −12.74(17), C4‐C5‐C6‐S7: 11.6(2) °.^[46]^

**Scheme 2:**
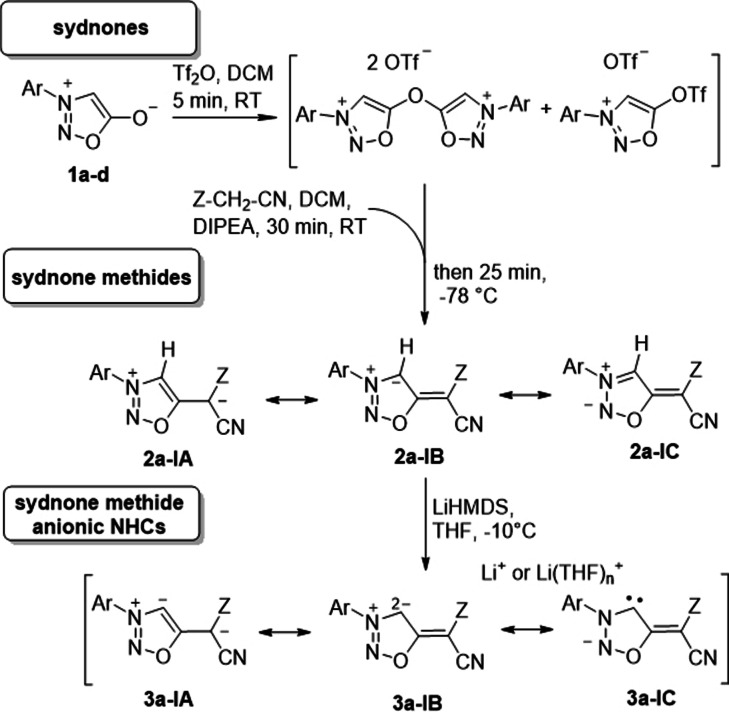
Syntheses of sydnone methides **2 a**–**l** and generation of anionic N‐heterocyclic carbenes **3 a**–**l** derived thereof (for substitution patterns, see Table 1). Selected mesomeric structures **A**–**C**. Several Li species detectable by ^7^Li NMR due to rapid decomposition of the anions.

**Table 1:** Substitution pattern and numbering of the sydnone methides **2 a**–**l**

Compd.	Ar	Z	Yield	
**2 a**	Ph	CN	53 %	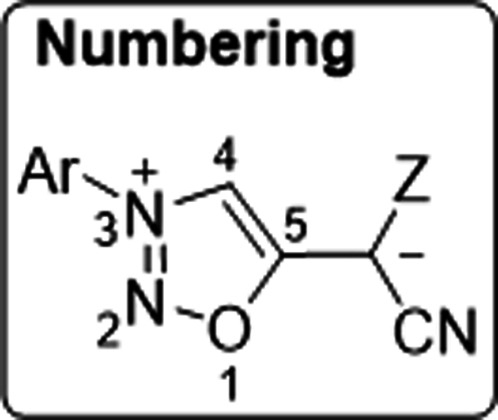
**2 b**	4‐C_6_H_4_Me	CN	38 %	
**2 c**	4‐C_6_H_4_OMe	CN	28 %	
**2 d**	4‐C_6_H_4_Cl	CN	33 %	
**2 e**	Ph	COOMe	61 %	
**2 f**	4‐C_6_H_4_Me	COOMe	65 %	
**2 g**	4‐C_6_H_4_OMe	COOMe	71 %	
**2 h**	4‐C_6_H_4_Cl	COOMe	17 %	
**2 i**	Ph	SO_2_Me	63 %	
**2 j**	C_6_H_4_Me	SO_2_Me	37 %	
**2 k**	4‐C_6_H_4_OMe	SO_2_Me	12 %	
**2 l**	4‐C_6_H_4_Cl	SO_2_Me	9 %	

Although the anions **3 a**–**l** are instable and decompose rapidly even at low temperatures, we successfully generated **3 j** quantitatively at −50 °C and immediately measured NMR spectra at −40 °C. The ^1^H NMR spectra show the absence of the proton at C4. In the ^13^C NMR spectra the signal of C4 of the precursor shifted considerably from 108.2 ppm (**2 j**) to 155.2 ppm (**3 j**). The carbene's resonance frequency is thus extremely shifted upfield in comparison to other NHCs. All chemical shift differences are summarized in Table S2 (Supporting Information). Moreover, the mass of the sydnone methide anion **3 j** was confirmed by high resolution electrospray ionization mass spectrometry in the anion detection mode on spraying a cooled in situ prepared sample of **3 j**. To gain insight into the electronic properties of the sydnone methide anions we performed DFT calculations (B3LYP/6‐311++G**). In contrast to 1,3‐dimesitylimidazol‐2‐ylidene and 1,3‐dimesitylimidazolidin‐2‐ylidene which we chose as examples, the highest occupied molecular orbitals (HOMOs) of the sydnone methide carbenes **3 a**, **3 e** and **3 i** are π‐orbitals with significant atomic orbital coefficients on C4 as legacy of their origin from mesoionic compounds (Figure 2 and Supporting Information). Vice versa, the HOMOs‐1 display the characteristic geometries of N‐heterocyclic carbenes. Their energies are considerably higher than those of the aforementioned N‐heterocyclic carbenes of imidazole, but slightly lower than those of N‐phenylsydnone (Figure 3). Concerning the substituent effects, the different electron‐withdrawing capacities of the COOMe group (Hammett constants^[36]^ *σ*_m_=0.37; *σ*_p_=0.45), CN group (*σ*_m_=0.56; *σ*_p_=0.66) and SO_2_Me group (*σ*_m_=0.60; *σ*_p_=0.72) correlates with the HOMO energies within the series of sydnone methide anions.

**Figure 2:**
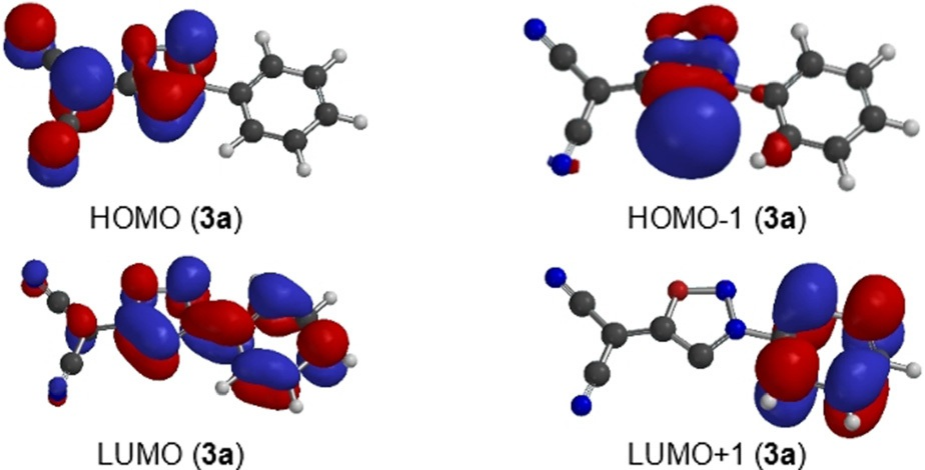
Calculated frontier orbital profile of the anion of sydnone methide **3 a**.

**Figure 3:**
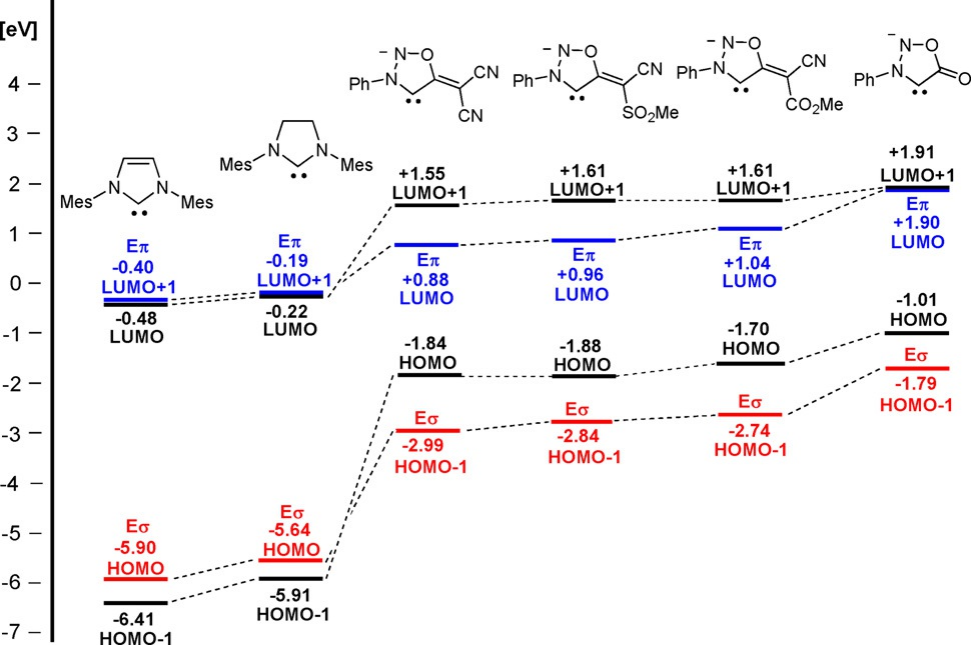
Comparison of selected molecular orbital energies in vacuo of imidazole‐2‐ylidene, imidazolidin‐2‐ylidene (left) and members of the sydnone family of carbenes (right) which are π‐electron‐rich N‐heterocyclic carbenes calculated on B3LYP/ 6‐311++G** level. Pictures of selected orbitals and HOMO/LUMO energies in THF are presented in the Supporting Information (see Table S1).

Reaction of the in situ generated anionic sydnone methide carbenes **3 a,e,i** with sulfur and selenium gave the thioethers **4 a**–**c** and selenium ethers **5 a**–**c a**fter methylation as stable compounds, respectively (Scheme 3). The ^77^Se NMR resonance frequencies of the selenium ethers **5 a**–**c** were detected at *δ*=86 ppm, 101 ppm and 109 ppm, respectively, and these values correspond to those measured for Molsidomine [that is, N‐(ethoxycarbonyl)‐3‐(4‐morpholino)sydnone imine; *δ*=97 ppm].^[37]^ As expected they are considerably more upfield than those of the selenium ethers of 1,3‐dimesitylimidazol‐2‐ylidene and its imidazolidine derivative which are cations and which resonate at *δ*=199 ppm and 271 ppm, respectively. Treatment of **3 a,e,i** with acetyl chloride (R=Me) and benzoyl chloride (R=Ph) gave **6 a**–**e** which slowly reconstitute the corresponding sydnone methides on exposure to water. The acetyl derivative of **6 f** (Z=SO_2_Me, R=Me) could not be isolated as it is unstable under ambient conditions. These reactions correspond to rare trapping reactions of normal[^38^, ^39^] as well as abnormal imidazolylidenes with acyl chlorides.^[39]^ Mesoionic compounds such as 1,3‐dimesitylimidazolium‐4‐olates, however, undergo electrophilic heteroaromatic substitutions to give these structures.^[40]^

**Scheme 3:**
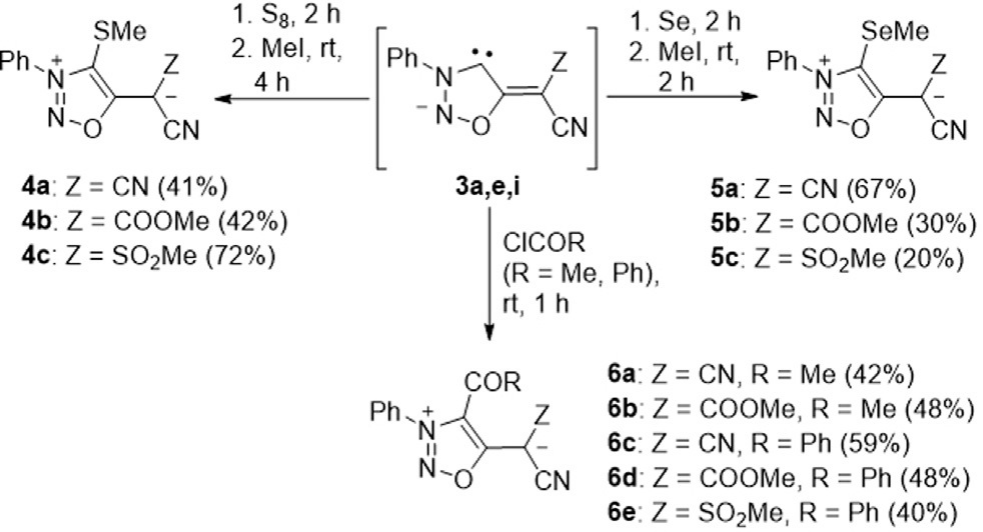
Chemistry of sydnone methide anionic carbenes with sulfur, selenium, and acyl chlorides, respectively.

The reaction of **3 e,f** with 0,5 equiv of mercury(II)‐chloride lead to the predominant formation of the dimeric mercury complexes **7_2_(a,b)**, whereas 3–4 equiv of HgCl_2_ yielded the monomeric complexes **7 a**,**b** from **3 b,f**. Chloro(triphenylphosphine)gold(I) converted the sydnone methide carbenes **3 c,f,i** into the gold(I) complexes **8 a**–**c** as yellow solids (Scheme 4). We also reacted the sydnone methide carbenes **3 a,f,i** with RhCO(PPh_3_)Cl which resulted in the formation of the rhodium complexes **9 a**–**c** as pale yellow solids.

**Scheme 4:**
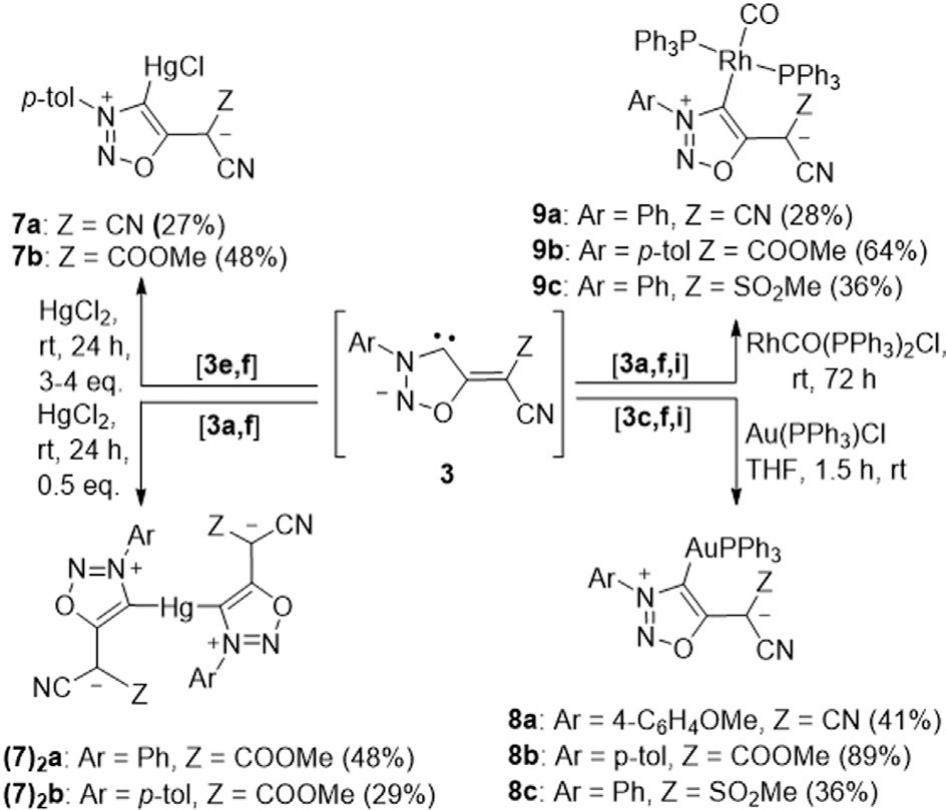
Trapping of the sydnone methide anionic carbenes with as mercury, gold, and rhodium complexes, respectively.

Single crystals of the gold complex **8 a** were subjected to an X‐ray analysis (Figure 4). The results show that complex formation does not influence the starting mesoion's geometry (**2 a**) significantly.

**Figure 4:**
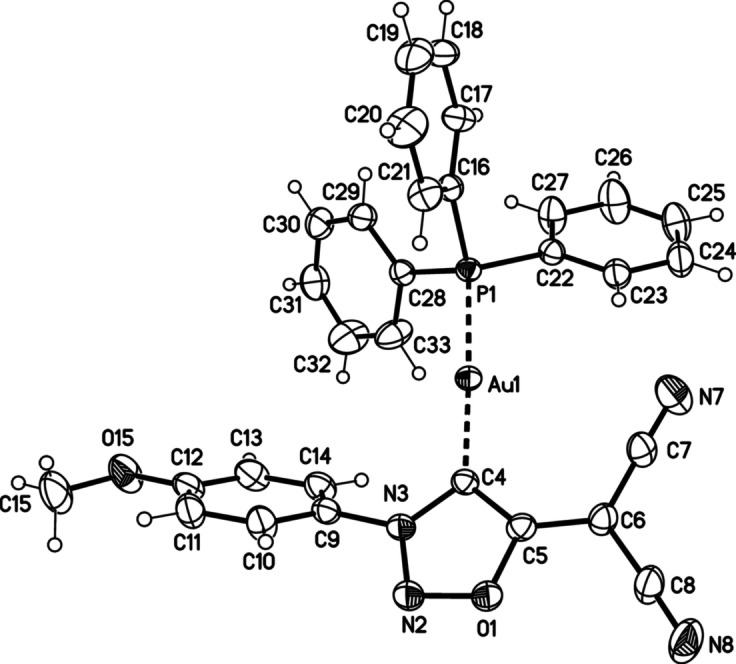
Molecular drawing of gold complex **8 a** (displacement parameters are drawn at 30 % probability level). Selected bond lengths [pm] (crystallographic numbering): N3–C4: 136.2(3), C4–C5: 138.7(3), C5–C6: 140.1(3), C6–C7: 141.3(3), C4–Au1: 204.07(19), C8–N8: 113.7(3) pm. Selected bonding angles [°]: N2‐N3‐C4: 116.39 (17), N3‐C4‐C5: 102.07(17), C4‐C5‐C6: 134.7(2) °. Selected torsion angles [°]: N2‐N3‐C9‐C10: 46.6(3), C4‐C5‐C6‐C7: 4.7(4)°.^[46]^

Concerning C−C coupling reactions, treatment of **3 a,e,i** with CuBr gave in situ generated, non‐isolable copper compounds, which coupled with 4‐fluoro‐1‐iodobenzene in the presence of Pd(PPh_3_)_4_ in acceptable yields (Scheme 5). These reactions proceed in analogy to those of sydnones.^[41]^

**Scheme 5:**
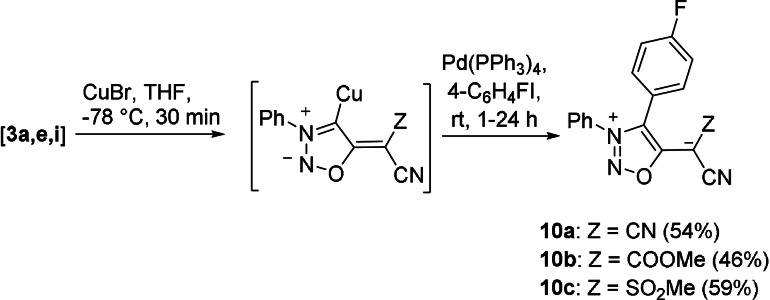
Copper‐catalysed C−C coupling reactions.

In summary, we present the syntheses, spectroscopic characterizations, results of calculations[^42^, ^43^] and single crystal X‐ray analyses[^44^, ^45^] of new stable members of the substance class of syndone methides from which only one single example had been described in 1984. Deprotonation yielded sydnone methide anions which can be formulated by a number of resonance forms, among those mesomeric structures of anionic N‐heterocyclic carbenes. The frontier orbital profile sets these N‐heterocyclic carbenes apart from other examples of this class of compounds, as their highest occupied molecular orbitals are π‐orbitals with considerable atomic orbital coefficients on the carbene carbon atom, and extremely upfield shifted ^13^C NMR resonance frequencies of the carbene carbon atom (**3 j**: *δ*=155.2 ppm). The sydnone methide anions can be reacted with sulfur and selenium, respectively, and stabilized by S‐ and Se‐methylation. Reaction with acyl chlorides gave sydnone methide ketones, and trapping reactions with mercury, gold, and rhodium gave the corresponding complexes. Finally, we presented a Pd^0^/Cu^I^‐catalyzed C−C coupling reaction at C4 of the sydnone methide. Our results supplement the knowledge about the sydnone family of compounds as well as about anionic N‐heterocyclic carbenes.

## Conflict of interest

The authors declare no conflict of interest.

## Supporting information

As a service to our authors and readers, this journal provides supporting information supplied by the authors. Such materials are peer reviewed and may be re‐organized for online delivery, but are not copy‐edited or typeset. Technical support issues arising from supporting information (other than missing files) should be addressed to the authors.

Supporting Information

Supporting Information
