# Supplementary material for: Sydnone Methides—A Forgotten Class of Mesoionic Compounds for the Generation of Anionic N‐Heterocyclic Carbenes
Source: Angew Chem Int Ed Engl. 2021 Jul 14;60(34):18882–7. doi: 10.1002/anie.202107495 (PMC8456854; doi:10.1002/anie.202107495)

Angewandte Chemie
Eine Zeitschrift der Gesellschaft Deutscher Chemiker

Supporting Information

Sydnone Methides—A Forgotten Class of Mesoionic Compounds for the Generation of Anionic N-Heterocyclic Carbenes

*Sebastian Mummel, Felix Lederle, Eike G. Hübner, Jan C. Namyslo, Martin Nieger, and Andreas Schmidt**

anie_202107495_sm_miscellaneous_information.pdf

S1. Crystal structure determinations …………………………………………… 2

S2. Experimental Section ……………………………………………………….. 2

S3. Calculations ………………………………………………………………….. 8

S4. NMR spectra ………………………………………………………………… 26

S5. Spectra of the anionic NHC formation …………………………………..... 141

S6. Fluorescence spectra ………………………………………………………. 146

S1. Crystal Structure Determination of 2e, 2i and 8a:

The single-crystal X-ray diffraction study were carried out on a Bruker D8 Venture diffractometer with PhotonII detector at 173(2) K (**2e**) or 298(2) K (**2i**, **8a**) using Cu-Kα radiation (λ = 1.54178 Å, **2e**, **2i**) or Mo-Kα radiation (λ = 0.71073 Å, **8a**). Dual space methods (SHELXT)[35] were used for structure solution and refinement was carried out using SHELXL-2014 (full-matrix least-squares on F^2).[36] Hydrogen atoms were localized by difference electron density determination and refined using a riding model. Semi-empirical absorption corrections were applied. For **8a** an extinction correction were applied.

2e: yellow crystals, $C_{11}H_9N_3O_3S$, M_r = 263.27, crystal size 0.36 × 0.09 × 0.03 mm, monoclinic, space group *P*2$_1$/n (No. 14), *a* = 11.7462(4) Å, *b* = 5.5324(2) Å, *c* = 17.8207(6) Å, *β* = 99.383(1)°, *V* = 1142.58(7) Å^3, *Z* = 4, *ρ* = 1.530 Mg/m^{-3}, μ(Cu-K$_\alpha$) = 2.59 mm^{-1}, *F*(000) = 544, *T* = 173(2) K $2\theta_{max}$ = 144.4°, 11557 reflections, of which 2236 were independent (R_{int} = 0.028), 164 parameters, R_1 = 0.028 (for 2183 I > 2σ(I)), wR_2 = 0.078 (all data), *S* = 1.06, largest diff. peak / hole = 0.27 / -0.33 e Å^{-3}.

2i: yellow crystals, $C_{12}H_9N_3O_3$, M_r = 243.22, crystal size 0.18 × 0.10 × 0.06 mm, triclinic, space group *P-1* (No. 2), *a* = 7.3046(5) Å, *b* = 7.6920(6) Å, *c* = 10.4222(8) Å, *α* = 81.770(2)°, *β* = 89.491(2)°, *γ* = 81.031(2)°, *V* = 572.42(7) Å^3, *Z* = 2, *ρ* = 1.411 Mg/m^{-3}, μ(Cu-K$_\alpha$) = 0.88 mm^{-1}, *F*(000) = 252, *T* = 298(2) K, $2\theta_{max}$ = 144.0°, 8956 reflections, of which 2197 were independent (R_{int} = 0.033), 164 parameters, R_1 = 0.041 (for 2130 I > 2σ(I)), wR_2 = 0.112 (all data), *S* = 1.05, largest diff. peak / hole = 0.21 / -0.19 e Å^{-3}.

8a: yellow crystals, $C_{30}H_{22}AuN_4O_2P$, M_r = 698.45, crystal size 0.14 × 0.10 × 0.06 mm, monoclinic, space group *P*2$_1$/n (No. 14), *a* = 11.9653(2) Å, *b* = 15.3853(3) Å, *c* = 15.4100(3) Å, *β* = 106.669(1)°, *V* = 2717.61(9) Å^3, *Z* = 4, *ρ* = 1.707 Mg/m^{-3}, μ(Mo-K$_\alpha$) = 5.51 mm^{-1}, *F*(000) = 1360, *T* = 298(2) K $2\theta_{max}$ = 55.0°, 65052 reflections, of which 6247 were independent (R_{int} = 0.031), 345 parameters, 300 restraints (see cif-file), R_1 = 0.015 (for 5621 I > 2σ(I)), wR_2 = 0.034 (all data), *S* = 1.04, largest diff. peak / hole = 0.48 / -0.37 e Å^{-3}.

CCDC 1055127 (**2e**), 1055128 (**2i**), and 1055129 (**8a**) contain the supplementary crystallographic data for this paper. These data can be obtained free of charge from The Cambridge Crystallographic Data Centre via www.ccdc.cam.ac.uk/data_request/cif.

S2. Experimental Section

All reactions were carried out under an atmosphere of nitrogen in flame or oven-dried glassware. All chemicals were purchased and used without further purification unless otherwise mentioned. Anhydrous solvents were dried according to standard procedures before usage. Abbreviations are as follows: DCM = dichloromethane, EE = ethyl acetate, PP = petroleum ether. Melting points are uncorrected and were determined in an apparatus according to Dr. Tottoli (Büchi). The ATR-IR spectra were obtained on a Bruker Alpha in the range of 400 to 4000 cm^{-1}. ^{1}H NMR spectra were recorded at 400 MHz or 600 MHz. ^{13}C NMR spectra were recorded at 100 MHz or 150 MHz, with the solvent peak used as the internal reference. Multiplicities are described by using the following abbreviations: s = singlet, d = doublet, t = triplet, q = quartet, and m = multiplet. Signal orientations in DEPT experiments were described as follows: o = no signal; + = up (CH, CH_3); - = down (CH_2). ^{77}Se NMR spectra were recorded at 114 MHz. The HR-MS spectra were obtained with a Bruker Impact II, a Bruker Daltonik Tesla-Fourier transform-ion cyclotron resonance mass spectrometer, or with a Waters Micromass LCT with direct inlet. Chromatography: The reactions were traced by thin layer chromatography with silica gel 60 (F254, company MERCK KGAA). For the detection of substances, quenching was used at either 254 nm or 366 nm with a mercury lamp. The preparative column chromatography was conducted through silica gel 60 (230 - 400 mesh) of the company MERCK KGAA.

General Procedure for the Synthesis of *N*-Aryl Sydnone Methides 2a-j: Under an inert atmosphere the corresponding *N*-aryl sydnone was dissolved in anhyd. Dichloromethane (6.5 ml/mmol). Then, under vigorous stirring trifluoromethylsulfonic anhydride (1.2 equiv.) were added and the mixture was then stirred for 5 minutes. The precipitated solid was resolved again, and the mixture was cooled to -78 °C. In a second flask, the malonic derivates (4 equiv.) were dissolved in anhyd. Dichloromethane (0.5 ml/mmol) under an inert atmosphere. Then, *N,N*-diisopropylethylamine (4 equiv.) was added and the mixture was stirred for 30 minutes. The solution of the second flask then was added slowly and under vigorous stirring to the solution of the first flask. The reaction mixture was stirred for 30 minutes at -78 °C. After the addition of water (50 mL) the mixture was allowed to warm to room temperature. The aqueous phase was extracted three times with dichloromethane. The combined organic phases were finally dried over $MgSO_4$ and were then purified by a flash column chromatography.

5-(Dicyanomethylene)-3-phenylsydnone (2a): *N*-Phenylsydnone (1.000 g, 6.17 mmol), malononitrile (0.815 g, 12.33 mmol), N,N-diisopropylethylamine (4.30 ml, 24.67 mmol) and Tf_2O (1.30 ml, 7.70 mmol) were used. The product was isolated as slightly orange solid in 53 % yield (0.689 g, 3.28 mmol). As eluent during flash chromatography DCM and EE were used (5:1). The obtained spectroscopic data were consistent with those reported in literature.[19] ^{1}H NMR (400 MHz, DMSO-d$_6$): δ = 9.12 (s, 1H; 4-H), 8.06 - 8.03 (m, 2H; 2'-H, 6'-H), 7.83 - 7.72 (m, 3H; 3'-H, 4'-H, 5'-H) ppm. ^{15}N NMR (61 MHz, DMSO-d$_6$): δ = -15.04 (s, 2N; N-7, N-8), -98.74 (s, 1N; N-3), -113.81 (s, 1N; N-2) ppm.

5-(Dicyanomethylene)-3-(4-tolyl)sydnone (2b): *N*-(4-Tolyl)sydnone (0.520 g, 2.95 mmol), malononitrile (0.390 g, 5.90 mmol), *N,N*-diisopropylethylamine (2.06 ml, 11.81 mmol) and Tf_2O (0.60 ml, 3.54 mmol) were used during synthesis. As eluent during flash chromatography DCM and EE were used (5:3). The product was isolated as slightly orange solid in 38 % yield (0.253 g, 1.13 mmol). Mp.:

157 °C (dec.).^{1}H NMR (600 MHz, DMSO-d$_6$): δ = 9.06 (s, 1H; 4-H), 7.93 (d, *J* = 8.4 Hz, 2H; 2'-H, 6'-H), 7.54 (d, *J* = 8.4 Hz, 2H; 3'-H, 5'-H), 2.44 (s, 3H; 7'-H) ppm. ^{13}C NMR (150 MHz, DMSO-d$_6$): δ = 174,6 (o, 1C; C-5), 144.5 (o, 1C; C-4'), 131.2 (+, 2C; C-3', C-5'), 130.7 (o, 1C; C-1'), 122.6 (+, 2C; C-2', C-6'), 116.8 (o, 2C; C-7, C-8), 109.9 (+, 1C; C-4), 34.1 (o, 1C; C-6), 21.4 (+, 1C; C-7') ppm. IR (ATR): $\tilde{\nu}$ = 3103, 2205, 2183, 1733, 1569, 1510, 1435, 1370, 1280, 1241, 1125, 1028, 1002, 943, 919, 810, 755, 607, 540, 502 cm^{-1}. HRMS (ESI) [$C_{12}H_8N_4ONa$]$^+$: 247.0590 (calcd.), 247.0591 (found).

5-(Dicyanomethylene)-3-(4'-methoxyphenyl)sydnone (2c): *N*-(4'-methoxyphenyl)sydnone (0.400 g, 2.08 mmol), malononitrile (0.275 g, 4.16 mmol), *N,N*-diisopropylethylamine (1.42 ml, 8.33 mmol) and Tf_2O (0.42 ml, 2.50 mmol) were used during synthesis. As eluent during flash chromatography DCM, EE and PE were used (5:1:1). The product was isolated as slightly orange solid in 65 % yield (0.400 g, 1.67 mmol). Mp.: 245 °C (dec.). ^{1}H NMR (600 MHz, DMSO-d$_6$): δ = 9.02 (s, 1H; 4-H), 7.99 (d, *J* = 9.27 Hz, 2H; 2'-H, 6'-H), 7.25 (d, *J* = 9 .27 Hz, 2H; 3'-H, 5'-H), 3.88 (s, 3H; 7'-H) ppm.^{13}C NMR (150 MHz, DMSO-d$_6$): δ = 174.4 (o, 1C; C-5), 163.3 (o, 1C; C-4'), 125.8 (o, 1C; C-1'), 124.6 (+, 2C; C-3', C-5'), 116.3 (o, 2C; C-7, C-8), 115.8 (+, 2C; C-2', C-6'), 109.6 (+, 1C, C-4), 56.5 (+, 1C; C-6), 33.8 (o, 1C, C-7') ppm. IR (ATR): $\tilde{\nu}$ = 3106, 2179, 1566, 1504, 1449, 1371, 1258, 1173, 986, 903, 837, 756, 619, 515 cm^{-1}. HRMS (ESI) [$C_{12}H_8N_4O_2Na$]$^+$: 263.0539 (calcd.), 263.0540 (found).

3-(4'-Chlorophenyl)-5-(dicyanomethylene)sydnone (2d): *N*-(4'-Chlorophenyl)sydnone (0.500 g, 2.54 mmol), malononitrile (0.336 g, 5.09 mmol), *N,N*-diisopropylethylamine (1.73 ml, 10.17 mmol) and Tf_2O (0.51 ml, 3.06 mmol) were used during synthesis. As eluent during flash chromatography DCM, EE and PE were used (8:3:3). The product was isolated as slightly orange solid in 33 % yield (0.207 g, 0.85 mmol). Mp.: 244 ° (dec.). ^{1}H NMR (600 MHz, DMSO-d$_6$): δ = 9.13 (s, 1H; 4-H), 8.08 (d, *J* = 9.08 Hz, 2H; 2'-H, 6'-H), 7.85 (d, *J* = 9.08 Hz, 2H; 3'-H, 5'-H) ppm. ^{13}C NMR (150 MHz, DMSO-d$_6$): δ = 174.7 (o, 1C; C-5), 138.5 (o, 1C; C-4'), 131.2 (+, 2C; C-3', C-5'), 130.7 (o, 1C; C-1'), 124.9 (+, 2C; C-2', C-6'), 116.1 (o, 2C; C-7, C-8), 110.7 (+, 1C; C-4) ppm. IR (ATR): $\tilde{\nu}$ = 3104, 2921, 2852, 2184, 1567, 1433, 1276, 1119, 1091, 1000, 918, 833, 819, 754, 539, 504, 473, 440 cm^{-1}. HRMS (ESI) [$C_{11}H_5ClN_4ONa^+$]: 267.0044 (calcd.), 267.0034 (found).

(*E*)-5-(1-Cyano-2-methoxy-2-oxoethylidene)-3-phenylsydnone (2e): *N*-Phenylsydnone (1.000 g, 6.17 mmol), methyl cyanoacetate (1.08 ml, 12.33 mmol), *N,N*-diisopropylethylamine (4.30 ml, 24.67 mmol) and Tf_2O (1.30 ml, 7.70 mmol) were used during synthesis. As eluent during flash chromatography DCM and EE were used (5:3). The product was isolated as slightly yellow solid in 61 % yield (0.920 g, 3.78 mmol). Mp.: 189 °C (dec.).^{1}H NMR (600 MHz, DMSO-d$_6$): δ = 8.84 (s, 1H; 4-H), 8.09 - 8.07 (m, 2H; 2'-H, 6'-H), 7.81 - 7.78 (m, 1H; 4'-H), 7.75 - 7.72 (m, 2H; 3'-H, 5'-H), 3.68 (s, 3H; 9-H) ppm. ^{13}C NMR (150 MHz, DMSO-d$_6$): δ = 173.3 (o, 1C; C-5), 164.8 (o, 1C, C-8), 133.2 (+, 1C; C-4'), 132.8 (o, 1C; C-1'), 130.2 (+, 2C; C-3', C-5'), 122.9 (+, 2C; C-2', C-6'), 117.2 (o, 1C; C-7), 110.8 (+, 1C; C-4), 57.1 (o, 1C; C-6), 50.9 (+, 1C; C-9) ppm. ^{15}N NMR (61 MHz, DMSO-d$_6$): δ = -20.74 (s, 1N; N-7), -99.32 (s, 1N; N-3), -115.95 (s, 1N; N-2) ppm. IR (ATR): $\tilde{\nu}$ = 2190, 1676, 1555, 1469, 1435, 1374, 1322, 1218, 1178, 1142, 1091, 1054, 1001, 878, 772, 757, 680, 528, 507, 443 cm^{-1}. HRMS (EI) [$C_{12}H_9N_3O_3Na$]$^+$: 266.0536 (calcd.), 266.0540 (found).

(*E*)-5-(1-Cyano-2-methoxy-2-oxoethylidene)-3-(4-tolyl)sydnone (2f): *N*-(*p*-Tolyl)sydnone (0.504 g, 2.86 mmol), methyl cyanoacetate (0.51 ml , 5.72 mmol), *N,N*-diisopropylethylamine (1.96 ml, 11.44 mmol) and Tf_2O (0.58 ml, 3.43 mmol) were used during synthesis. As eluent during flash chromatography DCM and EE were used (5:1). The product was isolated as pale yellow solid in 65 % yield (0.476 g, 1.85 mmol). Mp.: 201 °C (dec.). ^{1}H NMR (600 MHz, DMSO-d$_6$): δ = 8.78 (s, 1H; 4-H), 7.96 (d, *J* = 8.4 Hz, 2H; 2'-H, 6'-H), 7.52 (d, *J* = 8.4 Hz, 2H; 3'-H, 5'-H), 3.68 (s, 3H; 9-H), 2.44 (s, 3H; 7'-H) ppm. ^{13}C NMR (150 MHz, DMSO-d$_6$): δ = 173.7 (o, 1C; C-5), 165.3 (o, 1C; C-8), 144.2 (o, 1C; C-4'), 131.1 (o, 1C; C-1'), 130.9 (+, 2C; C-3', C-5'), 123.0 (+, 2C; C-2', C-6'), 117.7 (o, 1C; C-7), 110.9 (+, 1C, C-4), 56.5 (o, 1C; C-6), 51.4 (+, 1C; C-9), 21.4 (+, 1C; C-7') ppm. IR (ATR): $\tilde{\nu}$=2192, 1674, 1556, 1435, 1364, 1322, 1219, 1185, 1143, 1091, 1055, 1001, 931, 827, 780, 553, 510, 450 cm^{-1}. HRMS (ESI) [$C_{13}H_{11}N_3O_3Na^+$]: 280.0693 (calcd.), 280.0696 (found).

(*E*)-5-(1-Cyano-2-methoxy-2-oxoethylidene)-3-(4-methoxyphenyl)sydnone (2g): *N*-(4'-Methoxyphenyl)sydnone (0.406 g, 2.11 mmol), methyl cyanoacetate (0.38 ml , 4.23 mmol), *N,N*-diisopropylethylamin (1.44 ml, 8.45 mmol) and Tf_2O (0.43 ml, 2.54 mmol) were used during synthesis. As eluent during flash chromatography DCM, EE and PE were used (5:1:1). The product was isolated as pale yellow solid in 71 % yield (0.403 g, 1.47 mmol). Mp.: 189 °C. ^{1}H NMR (600 MHz, DMSO-d$_6$): δ = 8.74 (s, 1H; 4-H), 8.02 (d, *J* = 9.06 Hz, 2H; 2'-H, 6'-H), 7.23 (d, *J* = 9.06 Hz, 2H; 3'-H, 5'-H), 3.89 (s, 3H; 7'-H), 3.68 (s, 3H; 9-H) ppm. ^{13}C NMR (150 MHz, DMSO-d$_6$): δ = 173.5 (o, 1C; C-5), 165.3 (o, 1C; C-8), 163.1 (o, 1C; C-4'), 126.0 (o, 1C; C-1'), 124.9 (+, 2C; C-3', C-5'), 117.8 (o, 1C; C-7), 115.7 (+, 2C; C-2', C-6'), 110.6 (+, 1C; C-4), 57.2 (o, 1C; C-6), 56.5 (+, 1C; C-7'), 51.4 (+, 1C; C-9) ppm. IR (ATR): $\tilde{\nu}$= 3120, 2203, 1675, 1573, 1509, 1434, 1374, 1337, 1305, 1265, 1207, 1184, 1143, 1020, 991, 912, 830, 785, 756, 638, 556, 516, 418 cm^{-1}. HRMS (ESI) [$C_{13}H_{11}N_3O_4Na^+$]: 296.0642 (calcd.), 296.0642 (found).

(*E*)-3-(4-Chlorophenyl)-5-(1-cyano-2-methoxy-2-oxoethylidene)sydnone (2h): *N*-(4'-Chlorphenyl)sydnone (0.500 g, 2.54 mmol), methyl cyanoacetate (0.44 ml , 5.09 mmol), *N,N*-diisopropylethylamine (1.73 ml, 10.17 mmol) and Tf_2O (0.51 ml, 3.06 mmol) were used during synthesis. As eluent during flash chromatography DCM, EE and PE were used (8:3:3). The product was isolated as shiny yellow solid in 17 % yield (0.119 g, 0.42 mmol). Mp.: 212 °C. ^{1}H NMR (600 MHz, DMSO-d$_6$): δ = 8.87 (s, 1H; 4-H), 8.11 (d, *J* = 8.76 Hz, 2H; 2'-H, 6'-H), 7.82 (d, *J* = 8.76 Hz, 2H; 3'-H, 5'-H), 3.68 (s, 3H; 9-H) ppm. ^{13}C NMR (150 MHz, DMSO-d$_6$): δ = 173.8 (o, 1C; 5-C), 165.2 (o, 1C; C-8), 138.3 (o, 1C; C-4'), 132.0 (o, 1C; C-1'), 130.7 (+, 2C; C-3', C-5'), 125.4 (+, 2C; C-2', C-6'), 117.6 (o, 1C; C-7), 110.6 (+, 1C; 4-C), 57.8 (o, 1C; C-6), 51.5 (+, 1C; C-9) ppm. IR (ATR): $\tilde{\nu}$ = 3333, 3138, 3104, 3068, 2961, 2189, 2019, 1982, 1924, 1678, 1595, 1563, 1492, 1436, 1426, 1370, 1357, 1321, 1218, 1181, 1143, 1086, 1053, 1016, 997, 963, 956, 928, 875, 839, 770, 741, 694, 641, 619, 568, 523, 487, 449 cm^{-1}. HRMS (ESI) [$C_{12}H_8ClN_3O_3Na^+$]: 300.0146 (calcd.), 300.0142 (found).

(*E*)-5-(Cyano(methylsulfonyl)methylene)-3-phenylsydnone (2i): *N*-Phenylsydnone (1.000 g, 6.17 mmol), methylsulfonyl acetonitrile (1.470 g , 12.33 mmol), *N,N*-diisopropylethylamine (4.30 ml, 24.67 mmol) and Tf_2O (1.24 ml, 7.40 mmol) were used during synthesis. As eluent during flash chromatography DCM, EE and PE were used (2:2:1). The product was isolated as shiny greenish-yellow solid in 60 %

yield (0.970 g, 3.68 mmol). Mp.: 195 °C. ^{1}H NMR (600 MHz, DMSO-d$_6$): δ = 8.65 (s, 1H; 4-H), 8.06 (d, *J* = 6.91 Hz, 2H; 2'-H, 6'-H), 7.81-7.72 (m, 3H; 3'-H, 4'-H, 5'-H), 3.15 (s, 3H; 9-H) ppm. ^{13}C NMR (150 MHz, DMSO-d$_6$): δ = 172.1 (o, 1C; C-5), 133.8 (+, 1C; C-4'), 133.2 (o, 1C; C-1'), 130.8 (+, 2C; C-3', C-5'), 123.4 (+, 2C; C-2', C-6'), 116.0 (o, 1C; C-7), 109.8 (+, 1C; C-4), 67.0 (o, 1C, C-6), 44.9 (o, 1C; C-8) ppm. ^{15}N NMR (61 MHz, DMSO-d$_6$): δ = -19.64 (s, 1N; N-7), -99.39 (s, 1N; N-3), -112.30 (s, 1N; N-2) ppm. IR (ATR): $\tilde{\nu}$ = 3190, 2922, 2183, 1601, 1568, 1489, 1470, 1397, 1373, 1321, 1292, 1246, 1228, 1164, 1145, 1117, 1087, 1067, 1040, 1022, 992, 959, 942, 919, 906, 762, 722, 684, 670, 569, 553, 537, 490, 479, 433 cm^{-1}. HRMS (ESI) [$C_{11}H_9N_3O_3SNa^+$]: 286.0257 (calcd.), 286.0254 (found).

(*E*)-5-(Cyano(methylsulfonyl)methylene)-3-(4-tolyl)sydnone (2j): *N*-Tolylsydnone (0.585 g, 3.32 mmol), methylsulfonyl acetonitrile (0.791 g , 6.64 mmol), *N,N*-diisopropylethylamine (2.26 ml, 13.28 mmol) and Tf_2O (0.70 ml, 3.98 mmol) were used during synthesis. As eluent during flash chromatography DCM, EE and PE were used (5:1:2). The product was isolated as pale greenish-yellow solid in 37 % yield (0.339 g, 1.22 mmol). Mp.: 217 °C (dec.). ^{1}H NMR (600 MHz, DMSO-d$_6$): δ = 8.59 (s, 1H; 4-H), 7.95 (d, *J* = 8.02 Hz, 2H; 2'-H, 6'-H), 7.52 (d, *J* = 8.02 Hz, 2H; 3'-H, 5'-H), 3.15 (s, 3H; 9-H), 2.44 (s, 3H; 7'-H) ppm. ^{13}C NMR (150 MHz, DMSO-d$_6$): δ = 171.5 (o, 1C; C-5), 143.8 (o, 1C; C-1'), 130.6 (+, 2C; C-3', C-5'), 130.3 (o, 1C; C-4'), 122.5 (+, 2C; C-2', C-6'), 115.5 (o, 1C; C-7), 108.9 (+, 1C; C-4), 66.3 (o, 1C, C-6), 44.4 (o, 1C; C-8), 20.9 (+, 1C; C-7') ppm. IR (ATR): $\tilde{\nu}$ = 2182, 1569, 1432, 1371, 1287, 1147, 1121, 1037, 954, 936, 905, 818, 758, 726, 567, 536, 506, 488 cm^{-1}. HRMS (ESI) [$C_{12}H_{11}N_3O_3SNa^+$]: 300.0413 (calcd.), 300.0413 (found).

(*E*)-5-(Cyano(methylsulfonyl)methylene)-3-(4-methoxyphenyl)sydnone (2k): *N*-(4-Methoxyphenyl)sydnone (0.500 g, 2.60 mmol), methylsulfonyl acetonitrile (0.620 g , 5.20 mmol), *N,N*-diisopropylethylamine (1.77 ml, 10.41 mmol) and Tf_2O (0.52 ml, 3.12 mmol) were used during synthesis. As eluent during flash chromatography DCM, EE and PE were used (2:2:1). The product was isolated as pale greenish-yellow solid in 12 % yield (0.091 g, 0.31 mmol). Mp.: 207 °C (dec.). ^{1}H NMR (600 MHz, DMSO-d$_6$): 8.57 (s, 1H; 4-H), 8.02 (d, J=9.12 Hz, 2H; 2'-H, 6'-H), 7.24 (d, J=9.12 Hz, 2H; 3'-H, 5'-H), 3.89 (s, 3H; 7'-H), 3.14 (s, 3H; 7-H) ppm. ^{13}C NMR (150 MHz, DMSO-d$_6$): δ = 171.3 (o, 1C; C-5), 162.7 (o, 1C; C-4'), 125.4 (o, 1C; C-1'), 124.4 (+, 2C; C-3', C-5'), 115.6 (o, 1C; C-7, C-8), 115.3 (+, 2C; C-2', C-6'), 108.6 (+, 1C, C-4), 66.1 (o, 1C; C-6), 56.0 (+, 1C; C-7'), 44.4 (+, 1C; C-7) ppm. IR (ATR): $\tilde{\nu}$ = 3152, 2179, 1562, 1504, 1441, 1365, 1297, 1253, 1120, 1015, 960, 898, 833, 755, 565, 530 cm-1. HRMS (ESI) [$C_{12}H_{11}N_3O_4SNa$]$^+$: 316.0362 (calcd.), 316.0358 (found.).

(*E*)-3-(4-Chlorophenyl)-5-(Cyano(methylsulfonyl)methylene)sydnone (2l): *N*-(4-Chlorophenyl)sydnone (1.00 g, 5.09 mmol), methylsulfonyl acetonitrile (1.21 g , 10.17 mmol), *N,N*-diisopropylethylamine (3.46 ml, 20.35 mmol) and Tf_2O (1.03 ml, 6.10 mmol) were used during synthesis. As eluent during flash chromatography DCM, EE and PE were used (1:1:1). The product was isolated as pale greenish-yellow solid in 9 % yield (0.142 g, 0.48 mmol). Mp.: 215 °C (dec.). ^{1}H NMR (600 MHz, DMSO-d$_6$): δ = 8.69 (s, 1H; 4-H), 8.10 (d, *J*= 8.35 Hz, 2H; 2'-H, 6'-H), 7.82 (d, *J*=8.35 Hz, 2H; 3'-H, 5'-H), 3.15 (s, 3H; H-7) ppm. ^{13}C NMR (150 MHz, DMSO-d$_6$): δ = 171.6 (o, 1C; C-5), 137.9 (o, 1C; C-4'), 131.4 (o, 1C; C-1'), 130.7 (+, 2C; C-3', C-5'), 124.9 (+, 2C; C-2', C-6'), 115.4 (o, 1C; C-8), 109.7 (+, 1C; C-4), 66.7 (o, 1C; C-6), 44.4 (+, 1C; C-7) ppm. IR (ATR): $\tilde{\nu}$ = 3164, 2183, 1565, 1486,1429, 1350, 1291, 1119, 1088, 1037, 963, 831, 760, 566, 535, 497 cm^{-1}. HRMS (ESI) [$C_{11}H_8ClN_3O_3SNa^+$]: 319.9867 (calcd.), 319.9862 (found).

(*E*)-5-(Cyano(methylsulfonyl)methylen)-3-(4-tolyl)-sydnonide (3j): ***(E)*-5-(Cyano(methylsulfonyl)methylene)-3-(4-tolyl)sydnone** (0.02 g, 0.07 mmol) was dissolved in 0.6 mL anhyd. THF-d$_8$ and was cooled down to -50 °C. Than LHMDS (0.09 mL, 0.09 mmol) in THF was added. The reaction occurred in quantative yields. ^{1}H NMR (600 MHz, THF-d$_8$, -40 °C): δ = 7.98 (d, *J*=6.60 Hz, 2H; 2'-H, 6'-H), 7.38 (s, 2H; 3'-H, 5'-H), 3.04 (s, 3H; 9-H), 2.39 (s, 3H; 7'-H) ppm. ^{13}C NMR (150 MHz, THF-d$_8$, -40 °C): δ = δ = 181.2 (o, 1C; C-5), 155.2 (o, 1C; C-4), 143.8 (o, 1C; C-1'), 137.3 (o, 1C; C-4'), 130.6 (+, 2C; C-3', C-5'), 122.3 (+, 2C; C-2', C-6'), 118.2 (o, 1C; C-7), 63.8 (o, 1C, C-6), 44.1 (o, 1C; C-8), 21.0 (+, 1C; C-7') ppm. IR (ATR): $\tilde{\nu}$ = 2186, 1602, 1584, 1507, 1460, 1365, 1301, 1291, 1150, 1123, 1069, 1031, 837, 775, 754, 730, 682, 618 cm^{-1}. HRMS (ESI) [$C_{12}H_{10}N_3O_3S^-$]: 276.0448 (calcd.), 276.0453 (found).

General Procedure for Carbene Trapping Reactions of *N*-Phenylsydnone Methides with Chalcogenes 4a-5b: Under an inert atmosphere the corresponding *N*-aryl sydnone methides were dissolved in anhyd. THF (28.0 ml/mmol) and the resulting solutions were cooled to -10 °C. Then a 1 M solution of LHMDS (1.2 equiv) in THF was added dropwise and under vigorous stirring. After 10 minutes the chalcogen (1.5 equiv) was added and the solution was stirred for 2 hours. Then iodomethane was added, the mixture was stirred for 30 minutes and was allowed to warm to room temperature. After 2 additional hours of stirring the solution was filtered and was purified by a flash column. The separated product was obtained as oil which was resolved in THF and was precipitated through the addition of water. The solid was filtered off, washed with water, dried and subjected to column chromatography.

5-(Dicyanomethylene)-4-(methylthio)-3-phenylsydnone (4a): 5-(Dicyanomethylene)-3-phenylsydnone **2a** (30.0 mg, 0.14 mmol), LHMDS (0.17 ml, 0.17 mmol), sulfur (54.9 mg, 0.21 mmol) and iodomethane (0.008 ml, 0.14 mmol) were used for synthesis. As eluent during flash chromatography DCM, EE and PE were used (1:2:2). The product was isolated as yellow solid in 41 % yield (15.0 mg, 0.06 mmol). Mp.: 140 °C (dec.). ^{1}H NMR (600 MHz, DMSO-d$_6$): δ = 7.84 - 7.81 (m, 3H; 3'-H, 4'-H, 5'-H), 7.78-7.75 (m, 2H; 2'- H, 6'-H), 2.32 (s, 3H; 10-H) ppm. ^{13}C NMR (150 MHz, DMSO-d$_6$): δ = 174,2 (o, 1C; C-5), 133.6 (+, 1C; C-4'), 131.8 (o, 1C; C-1'), 130.5 (+, 2C; C-3', C-5'), 126.6 (+, 2C; C-2', C-6'), 117.6 (o, 2C; C-7, C-8), 115.6 (o, 1C; C-4), 35.6 (o, 1C; C-6), 19.8 (+, 1C; C-10) ppm. IR (ATR): $\tilde{\nu}$ = 2926, 2205, 1567, 1488, 1462, 1386, 1322, 1271, 1230, 1172, 1075, 1014, 910, 765, 703, 689, 650, 599, 547, 457 cm^{-1}. HRMS (ESI) [$C_{12}H_8N_4NaOSNa^+$]: 279.0311 (calcd.), 279.0311 (found).

(*E*)-5-(1-Cyano-2-methoxy-2-oxoethylidene)-4-(methylthio)-3-phenylsydnone (4b): (*E*)-5-(1-Cyano-2-methoxy-2-oxoethylidene)-3-phenylsydnone **2e** (30.0 mg, 0.12 mmol), LHMDS (0.15 ml, 0.15 mmol), sulfur (47.5 mg, 0.19 mmol) and iodomethane (0.015 ml, 0.21 mmol) were used for synthesis. As eluent during flash chromatography DCM, Et_2O and PE were used (1:2:2). The product was isolated as yellow solid in 42 % yield (15.1 mg, 0.05 mmol). Mp.: 161 °C (dec.). ^{1}H NMR (600 MHz, DMSO-d$_6$): δ = 7.85 - 7.79 (m, 3H; 3'-H, 4'-H, 5'-H), 7.76-7.74 (m, 2H; 2'- H, 6'-H), 3.68 (s, 3H; 9-H), 2.32 (s, 3H; 11-H) ppm. ^{13}C NMR (150 MHz, DMSO-d$_6$): δ = 173.8 (o, 1C; C-5), 164.9 (o, 1C; C-8), 133.3 (+, 1C; C-4'), 132.3 (o, 1C; C-1), 130.4 (+, 2C; C-3', C-5'), 126.8 (+, 2C; C-2', C-6'), 117.5 (o, 1C; C-4),

117.2 (o, 1C; C-7), 59.4 (o, 1C; C-6), 51.4 (+, 1C; C-9), 20.2 (+, 1C; C-11) ppm. IR (ATR): $\tilde{\nu}$ = 2191, 1681, 1537, 1432, 1371, 1268, 1192, 1123, 1091, 979, 921, 758, 690, 578, 525, 481 cm^{-1}. HRMS (ESI) [$C_{13}H_{11}N_3NaO_3SNa^+$]: 312.0413 (calcd.), 312.0403 (found).

(*E*)-5-(Cyano(methylsulfonyl)methylene)-4-(methylthio)-3-phenylsydnone (4c): (*E*)-5-(Cyano(methylsulfonyl)methylene)-3-phenylsydnone **2i** (100 mg, 0.38 mmol), LHMDS (0.40 ml, 0.40 mmol), sulfur (146 mg, 0.57 mmol) and iodomethane (0.03 ml, 0.57 mmol) were used for synthesis. For purification the solvent was removed under reduced pressure. Then, THF was added again to dissolve the solid, water was added to the solution, the resulting precipitate was filtered off and dried *in vacuo*. The product was isolated as yellow solid in 72 % yield (85.0 mg, 0.27 mmol). Mp.: 182 °C (dec.). ^{1}H NMR (600 MHz, DMSO-d$_6$): δ = 7.85-7.75 (m, 5H; 2‘-H, 3‘-H, 4‘-H, 5‘-H, 6‘-H), 3.21 (s, 3H; 8-H), 2.33 (s, 3H; 9-H) ppm. ^{13}C NMR (150 MHz, DMSO-d$_6$): δ = 170.7 (o, 1C; C-5), 133.5 (+, 1C; C-4‘), 132.0 (o, 1C, C-1‘), 130.5 (+, 2C; C-3‘, 5‘-C), 126.7 (+, 2C; C-2‘, C-6‘), 116.2 (o, 1C, C-4), 115.4 (o, 1C; C-7), 70.0 (o, 1C; C-6), 45.0 (+, 1C; C-8), 19.9 (+, 1C; C-9) ppm. IR (ATR): $\tilde{\nu}$ = 2920, 2852, 2181, 1544, 1489, 1418, 1376, 1304, 1264, 1141, 1107, 1008, 954, 886, 801, 755, 685, 653, 549, 489, 461 cm^{-1}. HRMS (EI) [$C_{12}H_{11}N_3O_3S_2Na$]$^+$: 332.0134 (calcd.), 332.0141 (found).

5-(Dicyanomethylene)-4-(methylselanyl)-3-phenylsydnone (5a): 5-(Dicyanomethylene)-3-phenylsydnone **2a** (38.0 mg, 0.18 mmol), LHMDS (0.22 ml, 0.22 mmol), selenium (17.3 mg, 0.22 mmol) and iodomethane (0.01 ml, 0.18 mmol) were used for synthesis. As eluent during flash chromatography DCM, EE and PE were used (6:3:3). The product was isolated as yellow solid in 67 % yield (37.0 mg, 0.12 mmol). Mp.: 150 °C (dec.). ^{1}H NMR (600 MHz, DMSO-d$_6$): δ = 7.80 - 7.76 (m, 3H; 3‘-H, 4’-H, 5‘-H), 7.74-7.71 (m, 2H; 2‘- H, 6‘-H), 2.18 (s, 3H; 10-H) ppm. ^{13}C NMR (150 MHz, DMSO-d$_6$): δ = 175,2 (o, 1C; C-5), 133.5 (+, 1C; C-4’), 132.8 (o, 1C; C-1), 130.4 (+, 2C; C-3’, C-5’), 126.7 (+, 2C; C-2’, C-6’), 115.9 (o, 2C; C-7, C-8), 110.6 (o, 1C; C-4), 35.6 (o, 1C; C-6), 12.1 (+, 1C; C-10) ppm. ^{77}Se NMR (114 MHz, diphenyldiselenid): δ = 85.86 (q, *J* = 12.1 Hz, 1Se; 9-Se) ppm. IR (ATR): $\tilde{\nu}$= 2961, 2191, 1721, 1563, 1486, 1461, 1371, 1261, 1220, 1171, 1011, 910, 797, 763, 688, 653, 592, 548 cm^{-1}. EIMS [$C_{12}H_8N_4OSe$]: 303.9863 (calcd.), 303.9861 (found).

(*E*)-5-(1-Cyano-2-methoxy-2-oxoethylidene)-4-(methylselanyl)-3-phenylsydnone (5b): (*E*)-5-(1-Cyano-2-methoxy-2-oxoethylidene)-3-phenylsydnone **2e** (120.0 mg, 0.49 mmol), LHMDS (0.59 ml, 0.59 mmol), selenium (46.7 mg, 0.59 mmol) and iodomethane (0.03 ml, 0.50 mmol) were used for synthesis. As eluent during flash chromatography $CHCl_3$ and Et_2O were used (1:2). The product was isolated as yellow solid in 30 % yield (49.0 mg, 0.15 mmol). Mp.: 167 °C (dec.). ^{1}H NMR (600 MHz, DMSO-d$_6$): δ = 7.84 - 7.72 (m, 5H; 2‘-H 3‘-H, 4’-H, 5‘-H, 6‘-H), 3.67 (s, 3H; 9-H), 2.20 (s, 3H; 11-H) ppm. ^{13}C NMR (150 MHz, DMSO-d$_6$): δ = 173.9 (o, 1C; C-5), 165.2 (o, 1C; C-8), 133.3 (+, 1C; C-4’), 133.2 (o, 1C; C-1‘), 130.3 (+, 2C; C-3’, C-5’), 126.8 (+, 2C; C-2’, C-6’), 117.8 (o, 1C; C-7), 110.7 (o, 1C; C-4), 59.2 (o, 1C; C-6), 51.4 (+, 1C; C-9), 12.3 (+, 1C; C-11) ppm. ^{77}Se NMR (114 MHz, diphenyldiselenid): δ = 100.61 (s, 1Se; 10-Se) ppm. IR (ATR): $\tilde{\nu}$ = 2924, 2189, 1668, 1531, 1463, 1367, 1258, 1189, 1121, 1079, 920, 757, 690, 571, 527, 431 cm^{-1}. HRMS (ESI) [$C_{13}H_{11}N_3O_3SeNa^+$]: 359.9858 (calcd.), 359.9860 (found).

(*E*)-5-(Cyano(methylsulfonyl)methylene)-4-(methylselanyl)-3-phenylsydnone (5c): (*E*)-5-(Cyano(methylsulfonyl)methylene)-3-phenylsydnone **2i** (300 mg, 1.14 mmol), LHMDS (1.70 ml, 1.71 mmol), selenium (270 mg, 3.42 mmol) and iodomethane (0.21 ml, 3.42 mmol) were used for synthesis. As solvent, instead of THF, acetonitrile was used. For purification the solvent was removed under reduced pressure. Then, THF was added again to dissolve the solid and the resulting solution was cooled to -20 °C. After 24 h the resulting precipitate was filtered off, washed with cold THF and dried *in vacuo*. The product was isolated as yellow solid in 20 % yield (81.0 mg, 0.27 mmol). Mp.: 211 °C. ^{1}H NMR (600 MHz, DMSO-d$_6$): δ = 7.85-7.79 (m, 3H; 3‘-H, 4‘-H, 5‘-H), 7.76-7.73 (m, 2H; 2‘-H, 6‘-H), 3.20 (s, 3H; 8-H), 2.21 (s, 3H; 10-H) ppm. ^{13}C NMR (150 MHz, DMSO-d6): δ = 171.8 (o, 1C; C-5), 133.3 (+, 1C; C-4‘), 133.0 (o, 1C, C-1‘), 130.4 (+, 2C; C-3‘, 5‘-C), 126.8 (+, 2C; C-2‘, C-6‘), 115.6 (o, 1C, C-7), 109.6 (o, 1C; C-4), 69.9 (o, 1C; C-6), 44.9 (+, 1C; C-8), 12.2 (+, 1C; C-10) ppm. ^{77}Se NMR (114 MHz, diphenyldiselenid): δ = 109.4 (s, 1Se; 9-Se) ppm. IR (ATR): $\tilde{\nu}$ = 2181, 1547, 1366, 1296, 1254, 1137, 1116, 1006, 926, 886, 757, 686, 575, 560, 538, 487, 423 cm^{-1}. HRMS (EI) [$C_{12}H_{11}N_3O_3SSeNa$]$^+$: 379.9579 (calcd.), 379.9572 (found).

General Procedure for Carbene Trapping Reaction of *N*-Phenylsydnone Methides with Acyl halides 6a-e: Under an inert atmosphere *N*-aryl sydnone methide was dissolved in anhyd. THF (28.0 ml/mmol) and was cooled to -10 °C. Then a 1 M solution of LHMDS (1.2 equiv) in THF was added dropwise and under vigorous stirring. After 10 minutes the corresponding acyl halide (2.5 equiv.) was added and the mixture was stirred for 10 minutes. Then the solution was allowed to warm to room temperature and was stirred for an additional hour. Afterwards water was added and the aqueous phase was extracted three times with dichloromethane. The combined organic phases were dried over $MgSO_4$ and purified by a flash column chromatography.

5-(Dicyanomethylene)-4-(methoxycarbonyl)-3-phenylsydnone (6a): 5-(Dicyanomethylene)-3-phenylsydnone **2a** (30.0 mg, 0.14 mmol), LHMDS (0.17 ml, 0.17 mmol) and methyl chloroformate (20.2 mg, 0.21 mmol) were used for synthesis. As eluent during flash chromatography $CHCl_3$ and Et_2O were used (10:1). The product was isolated as yellow solid in 42 % yield (16.0 mg, 0.59 mmol). Mp.: 168 °C (dec.). ^{1}H NMR (400 MHz, DMSO-d$_6$): δ = 7.82 - 7.78 (m, 3H; 3‘-H, 4’-H, 5‘-H), 7.74 - 7.69 (m, 2H; 2‘- H, 6‘-H), 3.73 (s, 3H; 10-H) ppm. ^{13}C NMR (100 MHz, DMSO-d$_6$): δ = 172.3 (o, 1C; C-5), 154.4 (o, 1C; C-9), 133.0 (+, 1C; C-4’), 132.2 (o, 1C; C-1‘), 129.6 (+, 2C; C-3’, C-5’), 125.8 (+, 2C; C-2’, C-6’), 115.2 (o, 1C; C-4), 113.9 (o, 2C; C-7, C-8), 52.1 (+, 1C; C-10), 38.3 (o, 1C; C-6) ppm. IR (ATR): $\tilde{\nu}$ = 2953, 2921, 2852, 2193, 1724, 1584, 1456, 1386, 1335, 1317, 1257, 1196, 1161, 1091, 1045, 1020, 925, 809, 757, 687, 633, 549, 420 cm^{-1}. HRMS (ESI) [$C_{13}H_8N_4O_3Na^+$]: 291.0489 (calcd.), 291.0490 (found).

(*E*)-5-(1-Cyano-2-methoxy-2-oxoethylidene)-4-(methoxycarbonyl)-3-phenylsydnone (6b): (*E*)-5-(1-Cyano-2-methoxy-2-oxoethylidene)-3-phenylsydnone **2e** (42.0 mg, 0.17 mmol), LHMDS (0.26 ml, 0.26 mmol) and methyl chloroformate (46.6 mg, 0.49 mmol) were used for synthesis. As eluent during flash chromatography $CHCl_3$ and Et_2O were used (10:1). The product was isolated as yellow solid in 48 % yield (25.0 mg, 0.08 mmol). Mp.: 169 °C (dec.). ^{1}H NMR (600 MHz, DMSO-d$_6$): δ = 7.85 - 7.78 (m, 3H; 3‘-H, 4’-H, 5‘-H), 7.73 - 7.71 (m, 2H; 2‘- H, 6‘-H), 3.71 (s, 3H; 11-H), 3.66 (s, 3H; 9-H) ppm. ^{13}C NMR (150 MHz, DMSO-d$_6$): δ = 171.3 (o, 1C; C-5), 164.8 (o, 1C; C-8), 156.3 (o, 1C; C-10), 133.6 (+, 1C; C-4’), 132.4 (o, 1C; C-1‘), 130.3 (+, 2C; C-3’, C-5’), 126.1 (+, 2C; C-2’, C-6’), 123.4 (o, 1C; C-7),

117.2 (o, 1C; C-4), 79.6 (o, 1C; C-6), 53.3 (+, 1C; C-11), 51.7 (+, 1C; C-9) ppm; the CN carbon atom was not detected. IR (ATR): $\tilde{\nu}$ = 2947, 2196, 1732, 1698, 1549, 1491, 1448, 1422, 1337, 1268, 1229, 1163, 1127, 1081, 1049, 937, 887, 806, 755, 682, 605 cm^{-1}. HRMS (ESI) [$C_{14}H_{11}N_3O_5Na^+$]: 324.0591 (calcd.), 324.0586 (found).

4-Benzoyl-5-(dicyanomethylene)-3-phenylsydnone (6c): 5-(Dicyanomethylene)-3-phenylsydnone **2a** (40.0 mg, 0.19 mmol), LHMDS (0.29 ml, 0.29 mmol) and benzoyl chloride (80.3 mg, 0.57 mmol) were used for synthesis. As eluent during flash chromatography $CHCl_3$ and Et_2O were used (10:1). The product was isolated as yellow solid in 59 % yield (35.0 mg, 0.11 mmol). Mp.: 176 °C (dec.). ^1H NMR (600 MHz, DMSO-d_6): δ = 7.92 - 7.90 (m, 3H; 3‘-H, 4’-H, 5‘-H), 7.69-7.67 (m, 3H; 2‘- H, 4“-H, 6‘-H), 7.60 - 7.57 (m, 2H; 2“-H, 6“-H), 7.49-7.46 (m, 2H; 3“-H, 5“-H) ppm. ^{13}C NMR (150 MHz, DMSO-d_6): δ = 180.2 (o, 1C; C-9), 172.9 (o, 1C; C-5), 136.1 (o, 1C; C-1“) 135.7 (+, 1C; C-4“), 133.7 (+, 1C; C-4’), 132.2 (o, 1C; C-1‘), 130.6 (+, 2C; C-2“ o. C-3‘ o. C-3“ o. C-5‘ o. C-5“ o. C-6“), 130.2 (+, 2C; C-2“ o. C-3‘ o. C-3“ o. C-5‘ o. C-5“ o. C-6“), 129.6 (+, 2C; C-2“ o. C-3‘ o. C-3“ o. C-5‘ o. C-5“ o. C-6“), 125.8 (+, 2C; C-2’, C-6’), 120.5 (o, 2C; C-7, C-8), 114.7 (o, 1C; C-4), 37.1 (o, 1C; C-6) ppm. IR (ATR): $\tilde{\nu}$ = 2922, 2207, 1722, 1650, 1558, 1488, 1465, 1450, 1283, 1207, 1174, 1073, 1019, 973, 929, 889, 789, 761, 726, 683, 552, 515 cm^{-1}. HRMS (ESI) [$C_{36}H_{20}N_8O_4Na^+$]: 651.1500 (calcd.), 651.1501 (found).

(*E*)-4-Benzoyl-5-(1-cyano-2-methoxy-2-oxoethylidene)-3-phenylsydnone (6d): (*E*)-5-(1-Cyano-2-methoxy-2-oxoethylidene)-3-phenylsydnone **2e** (75.0 mg, 0.31 mmol), LHMDS (0.46 ml, 0.46 mmol) and benzoyl chloride (108.4 mg, 0.77 mmol) were used for synthesis. As eluent during flash chromatography $CHCl_3$ and Et_2O were used (10:1). The product was isolated as yellow solid in 48 % yield (25.0 mg, 0.08 mmol). Mp.: 184 °C. ^1H NMR (600 MHz, DMSO-d_6): δ = 7.92 - 7.90 (m, 3H; 3‘-H, 4’-H, 5‘-H), 7.70 - 7.58 (m, 5H; 2‘-H, 3“-H, 4“-H, 5“-H 6‘-H), 7.51 - 7.46 (m, 2H; 2“-H, 6“-H), 3.32 (s, 3H; 9-H) ppm. ^{13}C NMR (150 MHz, DMSO-d_6): δ = 180.2 (o, 1C; C-10), 170.9 (o, 1C; C-5), 164.1 (o, 1C; C-8), 136.5 (o, 1C; C-1“) 134.5 (+, 1C; C-4“), 133.1 (+, 1C; C-4’), 131.6 (o, 1C; C-1‘), 130.0 (+, 2C; C-2“ o. C-3‘ o. C-3“ o. C-5‘ o. C-5“ o. C-6“), 129.1 (+, 2C; C-2“ o. C-3‘ o. C-3“ o. C-5‘ o. C-5“ o. C-6“), 128.8 (+, 2C; C-2“ o. C-3‘ o. C-3“ o. C-5‘ o. C-5“ o. C-6“), 128.5 (+, 2C; C-2’, C-6’), 125.5 (+, 2C; C-2’, C-6’), 120.9 (o, 1C; C-4), 116.8 (o, 1C; C-7), 50.8 (+, 1C; C-9) ppm. IR (ATR): $\tilde{\nu}$ = 2922, 2202, 1671, 1544, 1469, 1422, 1277, 1208, 1076, 1001, 917, 878, 683, 667, 555 cm^{-1}. HRMS (ESI) [$C_{19}H_{13}N_3O_4Na^+$]: 370.0798 (calcd.), 370.0803 (found).

(*E*)-4-Benzoyl-5-(cyano(methylsulfonyl)methylene))-3-phenylsydnone (6e): (*E*)-5-(Cyano(methylsulfonyl)methylene))-3-phenylsydnone **2i** (100.0 mg, 0.38 mmol), LHMDS (0.40 ml, 0.40 mmol) and benzoyl chloride (160.2 mg, 1.14 mmol) were used for synthesis. As eluent during flash chromatography $CHCl_3$ and Et_2O were used (10:1). The product was isolated as yellow solid in 40 % yield (56.0 mg, 0.15 mmol). Mp.: 211 °C (dec.). ^1H NMR (600 MHz, DMSO-d_6): δ = 7.91-7.90 (m, 2H; 2“-H, 6“-H), 7.69-7.68 (m, 2H; 3‘-H, 5‘-H), 7.64-7.62 (m, 2H; 4‘-H, 4“-H), 7.57-7.55 (m, 2H; 3“-H, 5“-H), 7.47-7.44 (m, 2H; 2‘-H, 6‘-H), 2.99 (s, 3H; 8-H) ppm. ^{13}C NMR (150 MHz, DMSO-d_6): δ = 181.3 (o, 1C; C-9‘), 169.6 (o, 1C; C-5), 136.3 (+, 1C; C-1‘), 135.5 (+, 1C, C-4“), 133.6 (+, 1C; C-4‘), 132.1 (o, 1C; C-1“), 130.5 (+, 2C, C-3“, C-5“), 130.3 (+, 2C; C-2“, C-6“), 129.4 (+, 2C; C-2‘, C-6‘), 125.9 (+, 2C; C-3‘, C-5‘), 119.7 (o, 1C; C-4), 115.1 (o, 1C; C-7), 69.1 (o, 1C, C-6), 44.3 (o, 1C; C-8) ppm. IR (ATR): $\tilde{\nu}$ = 2193, 1651, 1594, 1540, 1466, 1449, 1308, 1243, 1151, 1015, 965, 922, 874, 796, 758, 731, 688, 600, 599, 540, 488, 419 cm^{-1}. HRMS (EI) [$C_{18}H_{13}N_3O_4SNa$]$^+$: 390.0519 (calcd.), 390.0526 (found).

General Procedure for the Reaction of *N*-Aryl Sydnone Methides with Transition metals (7a-9c): Under an inert atmosphere *N*-aryl sydnone methide was dissolved in anhyd. THF (28.0 ml/mmol) and was cooled to -10 °C. Then a 1 M solution of LHMDS (1.2 equiv) in THF was added dropwise and under vigorous stirring. After 10 minutes the metal compound was added and the solution was stirred for several hours (tlc monitoring). Then water was added and the aqueous phase was extracted three times with dichloromethane. The combined organic phases were dried over $MgSO_4$ and were purified by a flash column chromatography.

(5-(Dicyanomethylene)-3-(4-tolyl)sydnone-4-yl)mercury(II) chloride (7a): 5-(Dicyanomethylene)-3-(4-tolylsydnone (100.0 mg, 0.45 mmol), LHMDS (0.54 ml, 0.54 mmol) and $HgCl_2$ (484.0 mg, 1.78 mmol) were used for the synthesis. As eluent during flash chromatography DCM, EE, and PP were used (5:2:2). The product was isolated as yellow solid in 27 % yield (56.0 mg, 0.12 mmol). Mp.: 207 °C (dec.). ^1H NMR (600 MHz, DMSO-d_6): δ = 7.89 (d, *J* = 8.12 Hz, 2H; 2‘-H, 6‘-H), 7.52 (d, *J* = 8.12 Hz, 2H; 3‘-H, 5‘-H), 2.45 (s, 3H; 7‘-H) ppm. ^{13}C NMR (150 MHz, DMSO-d_6): δ = 179.1 (o, 1C; C-5), 143.6 (o, 1C; C-1‘), 139.5 (o, 1C; C-4), 133.3 (o, 1C; C-4‘), 130.9 (+, 2C; C-3‘, C-5‘), 124.7 (+, 2C; C-2‘, C-6‘), 117.4 (o, 2C; C-7, C-8), 34.1 (o, 1C; C-6), 21.4 (+, 1C; C-7‘) ppm. IR (ATR): $\tilde{\nu}$ = 2921, 2852, 2194, 2164, 1540, 1502, 1416, 1384, 1296, 1263, 1225, 1207, 1178, 1117, 1105, 1036, 1012, 944, 928, 815, 711, 663, 578, 545, 5211, 496, 442, 416 cm^{-1}. HRMS (ESI) [$C_{12}H_7ClHgN_4ONa^+$]: 482.9907 (calcd.), 482.9906 (found).

(*E*)-(5-(1-Cyano-2-methoxy-2-oxoethylidene)-3-(4-tolyl)sydnone-4-yl)mercury(II) chloride (7b): (*E*)-5-(1-Cyano-2-methoxy-2-oxoethylidene)-3-(4-tolyl)sydnone (60.0 mg, 0.23 mmol), LHMDS (0.28 ml, 0.28 mmol) and $HgCl_2$ (253.0 mg, 0.93 mmol) were used for the synthesis. As eluent during flash chromatography DCM, EE and PE were used (2:1:1). The product was isolated as yellow solid in 47 % yield (60.0 mg, 0.12 mmol). Mp.: 238 °C (dec.). ^1H NMR (600 MHz, DMSO-d_6): δ = 7.99 (d, *J* = 8.16 Hz, 2H; 3‘-H, 5‘-H), 7.57 (d, *J* = 8.16 Hz, 2H; 2‘-H, 6‘-H), 3.70 (s, 3H; 9-H), 2.46 (s, 3H; 7‘-H) ppm. ^{13}C NMR (150 MHz, DMSO-d_6): δ = 177.0 (o, 1C; C-5), 167.6 (o, 1C; C-8), 143.9 (o, 1C; C-4‘), 140.9 (o, 1C; C-4), 133.9 (o, 1C; C-1‘), 131.2 (+, 2C; C-3‘, C-5‘), 124.4 (+, 2C; C-2‘, C-6‘), 117.9 (o, 1C; C-7), 57.9 (o, 1C; C-6), 52.0 (+, 1C; C-9), 21.3 (+, 1C; C-7‘) ppm. IR (ATR): $\tilde{\nu}$ = 2943, 2190, 1643, 1529, 1435, 1376, 1317, 1228, 1201, 1180, 1146, 1065, 1013, 937, 820, 749, 574, 447 cm^{-1}. HRMS (ESI) [$C_{12}H_8ClHgN_3O_3Na^+$]: 516.0009 (calcd.), 516.0009 (found).

Bis((*E*)-5-(1-cyano-2-methoxy-2-oxoethylidene)-3-phenylsydnone-4-yl)mercury ($(7)_2$a): (*E*)-5-(1-Cyano-2-methoxy-2-oxoethylidene)-3-phenylsydnone (115.0 mg, 0.47 mmol), LHMDS (0.57 ml, 0.57 mmol) and $HgCl_2$ (64.0 mg, 0.24 mmol) were used for the synthesis. The crude product was washed with water. The product was isolated as yellow solid in 48 % yield (143.0 mg, 0.23 mmol). Mp.: 285 °C (dec.). ^1H NMR (600 MHz, DMSO-d_6): δ = 8.06-8.05 (m, 4H; 2‘-H, 6‘-H), 7.78-7.77 (m, 2H; 4‘-H), 7.70-7.67 (m, 4H; 3‘-H, 5‘-H), 3.53 (s, 6H; 9-H) ppm. ^{13}C NMR (150 MHz, DMSO-d_6): δ = 178.6 (o, 2C; C-5), 167.1 (o, 2C; C-8), 146.0 (o, 2C; C-4), 136.1 (o, 2C; C-1‘), 133.2 (+, 2C; C-4), 130.6 (+, 4C; C-3‘, C-5‘), 125.1 (+, 4C; C-2‘, C-6‘), 118.1 (+, 2C; C-7), 57.6 (o, 2C; C-6), 51.7 (+, 2C; C-9) ppm. IR (ATR): $\tilde{\nu}$ = 2923,

2186, 1642, 1519, 1440, 1374, 1290, 1197, 1140, 1061, 1014, 940, 799, 759, 685, 587, 534, 440 cm^{-1}. HRMS (ESI) [$C_{24}H_{16}HgN_6O_6Na^+$]: 709.0730 (calcd.), 709.0733 (found).

Bis((*E*)-5-(1-cyano-2-methoxy-2-oxoethylidene)-3-(4-tolyl)-sydnone-4-yl)mercury ((7)$_2$b): (*E*)-5-(1-Cyano-2-methoxy-2-oxoethylidene)-3-(4-tolyl)sydnone (75.0 mg, 0.29 mmol), LHMDS (0.35 ml, 0.35 mmol) and $HgCl_2$ (199.0 mg, 0.44 mmol) were used for the synthesis. As eluent during flash chromatography DCM, EE and PE were used (6:2:4). The product was isolated as yellow solid in 29 % yield (60.0 mg, 0.09 mmol). Mp.: 278 °C (dec.). ^{1}H NMR (600 MHz, DMSO-d$_6$): δ = 7.92 (d, *J* = 8.05 Hz, 4H; 2'-H, H-6'),), 7.46 (d, *J* = 8.05 Hz, 4H; 3'-H, 5'-H), 3.54 (s, 6H; 9-H), 2.43 (s, 6H; 7'-H) ppm. ^{13}C NMR (150 MHz, DMSO-d$_6$): δ = 178.5 (o, 2C; C-5), 167.2 (o, 2C; C-8), 145.8 (o, 2C; C-4'), 143.6 (o, 2C; C-4), 133.7 (o, 2C; C-1'), 130.9 (+, 4C; C-3', C-5'), 124.8 (+, 4C; C-2', C-6'), 118.2 (o, 2C; C-7), 57.5 (o, 2C; C-6), 51.6 (+, 2C; C-9), 21.3 (+, 2C; C-7') ppm. IR (ATR): $\tilde{\nu}$ = 2187, 1641, 1524, 1434, 1367, 1292, 1193, 1141, 1060, 1005, 936, 819, 755, 570, 522, 450 cm^{-1}. HRMS (EI) [$C_{26}H_{20}HgN_6O_6Na$]$^+$: 737.1043 (calcd.), 737.1037 (found).

(*E*)-(5-(Dicyanomethylene)-3-(4'-methoxyphenyl)sydnone-4-yl)(triphenylphosphine)gold (8a): (*E*)-(5-(Dicyanomethylene)-3-(4'-methoxyphenyl)sydnone (50.0 mg, 0.21 mmol), LHMDS (0.31 ml, 0.31 mmol) and triphenylphosphinegold(I)-chloride (154.0 mg, 0.31 mmol) were used for the synthesis. As eluent during flash chromatography DCM, EE and PE were used (6:2:4). The product was isolated as yellow solid in 41 % yield (60.0 mg, 0.09 mmol). Mp.: 189 °C. ^{1}H NMR (600 MHz, DMSO-d$_6$): δ = 8.07 (d, *J* = 9.07 Hz, 2H; 2'-H, 6'-C), 7.64-7.48 (m, 15H; 2''-H, 3''-H, 4''-H, 5''-H, 6''-H), 7.13 (d, *J* = 9.07 Hz, 2H; 2'-H, 6'-H), 3.84 (s, 3H; 7'-H) ppm. ^{13}C NMR (150 MHz, DMSO-d$_6$): δ = 181.1 (o, 1C; C-5), 162.2 (o, 1C; C-4'), 155.9 (o, 1C; C-4), 134.4 (+, $J_{C,P}$ = 13.1 Hz, 6C, C-2'', C-6''),132.6 (+, 3C; C-4''),130.0 (+, $J_{C,P}$=11.4 Hz, 6C, C-3'', C-5''), 129.8 (o, 1C; C-1), 128.9 (o, $J_{C,P}$ = 57.2 Hz, 3C; C-1''), 126.7 (+, 2C; C-2', C-6'), 115.2 (+, 2C, C-3', C-5') 56.4 (o, 1C; C-7'), 33.6 (+, 1C; C-6) ppm; the CN carbon atom was not detected. IR (ATR): $\tilde{\nu}$ = 2198, 2165, 1537, 1505, 1432, 1368, 1252, 1206, 1166, 1100, 1018, 999, 920, 831, 800, 749, 710, 689, 581, 537, 496 cm^{-1}. HRMS (ESI) [$C_{30}H_{22}AuN_4O_2PNa^+$]: 721.1038 (cal.), 721. 1045 (found).

(*E*)-(5-(1-Cyano-2-methoxy-2-oxoethylidene)-3-(4-tolyl)sydnone-4-yl)(triphenylphosphine)gold (8b): (*E*)-(5-(1-Cyano-2-methoxy-2-oxoethylidene)-3-(4-tolyl)sydnone (50.0 mg, 0.19 mmol), LHMDS (0.23 ml, 0.23 mmol) and triphenylphosphinegold(I)-chloride (144.0 mg, 0.29 mmol) were used for the synthesis. As eluent during flash chromatography DCM, EE and PE were used (6:2:4). Mp.: 194 °C. The product was isolated as yellow solid in 89 % yield (123.0 mg, 0.17 mmol). ^{1}H NMR (600 MHz, DMSO-d$_6$): δ = 7.97 (d, *J* = 8.75 Hz, 2H; 3'-H, 5'-C), 7.62-7.47 (m, 15H; 2''-H, 3''-H, 4''-H, 5''-H, 6''-H), 7.41 (d, *J* = 8.75 Hz, 2H; 2'-H, 6'-H), 3.61 (s, 3H; 9-H), 2.40 (s, 3H; 7'-H) ppm. ^{13}C NMR (150 MHz, DMSO-d$_6$): δ = 180.5 (o, 1C; C-5), 165.6 (o, 1C; C-8), 157.3 (o, 1C; C-4), 142.5 (o, 1C; C-4'), 135.1 (o, 1C, C-1'),134.5 (+, $J_{C,P}$ = 14.2 Hz, 6C; C-2'', C-6''), 132.4 (+, 1C; C-4''), 130.5 (+, 2C, C-2', C-6'), 130.0 (+, $J_{C,P}$=10.7 Hz, 6C; C-3'', C-5''), 129.3 (o, $J_{C,P}$ = 56.9 Hz, 1C; C-1''), 125.2 (+, 2C, C-3', C-5'), 121.9 (o, 1C, C-7), 58.1 (o, 1C; C-6), 50.9 (+, 1C; C-9), 21.3 (+, 1C; C-7') ppm. IR (ATR): $\tilde{\nu}$ = 2181, 1660, 1514, 1514, 1431, 1280, 1181, 1115, 818, 750, 691, 534, 502 cm^{-1}. HRMS (ESI) [$C_{31}H_{25}AuN_3O_3PNa^+$]: 738.1191 (calcd.), 738.1183 (found).

(*E*)-(5-(Cyano(methylsulfonyl)methylene)-3-phenylsydnone-4-yl)(triphenylphosphin)gold (8c): (*E*)-(5-(Cyano(methylsulfonyl)methylene)-3-phenylsydnone (50.0 mg, 0.19 mmol), LHMDS (0.23 ml, 0.23 mmol) and triphenylphosphinegold(I)-chloride (113.0 mg, 0.23 mmol) were used for the synthesis. As eluent during flash chromatography DCM, EE and PE were used (5:2:3). The product was isolated as yellow solid in 36 % yield (49.0 mg, 0.07 mmol). Mp.: 192 °C. ^{1}H NMR (600 MHz, DMSO-d$_6$): δ = 8.14-8.12 (m, 2H; 3'-H, 5'-C), 7.76-7.73 (m, 1H; 4'-H), 7.67-7.46 (m, 17H; 2'-H, 2''-H, 3''-H, 4''-H, 5''-H, 6'-H, 6''-H), 3.14 (s, 3H; 8-H) ppm. ^{13}C NMR (150 MHz, DMSO-d$_6$): δ = 178.3 (o, 1C; C-5), 155.9 (o, 1C; C-4), 137.2 (o, 1C, C-1'),134.4 (+, $J_{C,P}$=12.8 Hz, 6C; C-3'', C-5''), 132.5 (+, 2C; C-4', C-4''), 130.3 (+, 2C, C-2', C-6'), 130.0 (+, $J_{C,P}$ = 11.1 Hz, 6C; C-2'', C-6''), 129.2 (o, $J_{C,P}$ = 57.3 Hz, 3C; C-1''),125.4 (+,2C, C-3', C-5'), 119.5 (o, 1C; C-7), 68.1 (o, 1C, C-6), 45.1 (o, 1C; C-8) ppm. IR (ATR): $\tilde{\nu}$ = 2172, 1517, 1435, 1370, 1302, 1223, 1099, 932, 890, 745, 687, 572, 534, 497 cm^{-1}. HRMS (ESI) [$C_{30}H_{25}AuN_3O_3PSNa^+$]: 744.0755 (calcd.), 744.0744 (found).

5,5-(Dicyanomethylene)-3-phenylsydnone-4-yl)(bisphenylphosphine)carbonylrhodium(I) (9a): 5-(Dicyanomethylene)-3-phenylsydnone (50.0 mg, 0.24 mmol), LHMDS (0.29 ml, 0.29 mmol) and $RhCO(PPh_3)Cl$ (181.0 mg, 0.26 mmol) were used for the synthesis. As eluent during flash chromatography DC, EE and PE were used (1:1:1). The product was isolated as yellow solid in 28 % yield (59.0 mg, 0.07 mmol). Mp.: 205 °C (dec.). ^{1}H NMR (600 MHz, $CDCl_3$): δ = 7.73-7.71 (m, 3H; 2'-H, 4'-H, 6'-H), 7.49-7.35 (m, 30H; 2''-H, 3''-H, 4''-H, 5''-H, 6''-H), 7.09-7.07 (m, 2H; 3'-H, 5'-H) ppm. ^{13}C NMR (150 MHz, DMSO-d$_6$): δ = 191.1 (o, d, $J_{C,Rh}$ = 74.6 Hz, 1C; C-10), 178.8 (o, 2C; C-5), 145.2 (m, 1C; C-4), 146.0 (o, 2C; C-4), 136.8 (o, 1C; C-1'), 135.0 (+, t, $J_{C,P}$ = 5.9 Hz, 12C; C-2'', C-6''), 132.7 (o, t, $J_{C,P}$ = 22.1 HZ, 6C; C-1''), 130.7 (+, 2C; C-2', C-6'), 130.2 (+, 1C; C-4'), 128.5 (+, t, $J_{C,P}$ = 5.4 Hz, 12C; C-3'', C-5''), 123.8 (+, 2C, C-3', C-5'), 34.9 (o, 1C; C-6) ppm; the CN carbon atom was not detected. IR (ATR): $\tilde{\nu}$ = 3050, 2193, 2171, 2007, 1525, 1432, 1181, 1092, 1007, 911, 742, 689, 558, 515 cm^{-1}. HRMS (EI) [$C_{48}H_{35}N_4O_2P_2RhNa$]$^+$: 887.1182 (calcd.), 887.1190 (found).

((*E*)-5-(1-Cyano-2-methoxy-2-oxoethylidene)-3-(4-tolyl)sydnone-4-yl)(bisphenylphosphine)carbonylrhodium(I) (9b): (*E*)-5-(1-Cyano-2-methoxy-2-oxoethylidene)-3-(4-tolyl)sydnone (50.0 mg, 0.20 mmol), LHMDS (0.23 ml, 0.23 mmol) and $RhCO(PPh_3)Cl$ (162.0 mg, 0.23 mmol) were used for the synthesis. As eluent during flash chromatography DC, EE and PE were used (1:1:1). The product was isolated as yellow solid in 64 % yield (114.0 mg, 0.06 mmol). Mp.: 172 °C. ^{1}H NMR (600 MHz, DMSO-d$_6$): δ = 7.48-7.40 (m, 33H; 1''-H, 2''-H, 3'-H 3''-H, 4''-H, 5'-H, 5''-H, 6''-H), 7.04-7.03 (m, 2H; 2'-H, 6'-H), 3.41 (s, 3H; 9-H), 2.38 (s, 3H; 7'-H) ppm. ^{13}C NMR (150 MHz, DMSO-d$_6$): δ = 177.8 (o, 1C; C-5), 165.5 (o, 1C; C-8), 141.8 (o, 1C; C-4'), 134.4 (o, 1C; C-1'), 134.0 (+, $J_{C,P}$ = 12.1 Hz, 6C; C-2'', C-6''), 133.0 (o, $J_{C,P}$ = 44.1 Hz, 3C; C-1''), 132.0 (o, $J_{C,P}$ = 9.8 Hz, 1C; C-4), 131.0 (+, 3C; C-4''), 129.3 (+, 2C; C-3', C-5'), 128.8 (+, $J_{C,P}$ =9.8 Hz, 6C; C-3'', C-5'') 124.1 (+, 2C; C-2', C-6'), 60.2 (o, 2C; C-6), 50.2 (+, 2C; C-9), 21.3 (+, 1C; C-7'); the CN carbon atom was not detected. IR (ATR): $\tilde{\nu}$ = 2167, 1989, 1673, 1508, 1431, 1267, 1177, 1092, 1075, 1009, 939, 742, 690, 562, 515, 414 cm^{-1}. HRMS (EI) [$C_{50}H_{40}N_3O_4P_2RhNa$]$^+$: 934.1441 (calcd.), 934.1445 (found).

((*E*)-5-(Cyano(methylsulfonyl)methylene)-3-phenylsydnone-4-yl)(bisphenylphosphine)carbonylrhodium(I) (9c): (*E*)-5-(Cyano(methylsulfonyl)methylene)-3-phenylsydnone (50.0 mg, 0.21 mmol), LHMDS (0.23 ml, 0.23 mmol) and RhCO(PPh_3)Cl (144.0 mg, 0.24 mmol) were used for the synthesis. As eluent during flash chromatography DC, EE and PE were used (1:1:1). The product was isolated as yellow solid in 36 % yield (63.0 mg, 0.08 mmol). Mp.: 192 °C (dec.). ^{1}H NMR (600 MHz, $CDCl_3$): δ = 7.73-7.71 (m, 2H; 3'-H, 5'-H), 7.48-7.33 (m, 31H; 2''-H, 3''-H, 4'-H, 4''-H, 5''-H, 6''-H), 7.09-7.07 (m, 2H; 2'-H, 6'-H), 2.59 (s, 3H; 8-H) ppm. ^{13}C NMR (150 MHz, $CDCl_3$): δ = 190.9 (o, d, $J_{C,Rh}$ = 58.6 Hz, 1C; C-9), 175.4 (o, 1C; C-5), 146.2-145.7 (o, m, 1C; C-4), 146.0 (o, 2C; C-4), 136.1 (o, 1C; C-1'), 134.1 (+, t, $J_{C,P}$ = 5.9 Hz, 12C; C-2'', C-6''), 132.8 (o, t, $J_{C,P}$ = 22.4 Hz, 6C; C-1''), 132.0 (+, 6C; C-4''), 130.7 (+, 1C; C-4'), 130.5 (+, 2C; C-2', C-6'), 128.4 (+, t, $J_{C,P}$ = 3.9 Hz, 12C; C-3'', C-5''), 124.0 (+, 2C; C-3', C-5') 120.4 (o, 1C; C-7), 68.1 (o, 1C; C-6), 44.5 (+, 1C; C-8) ppm. IR (ATR): $\tilde{\nu}$ = 3050, 2922, 2851, 2169, 1979, 1734, 1507, 1433, 1298, 1215, 1124, 1092, 887, 744, 691, 562, 513 cm^{-1}. HRMS (EI) $[C_{48}H_{38}N_3O_4P_2RhSNa]^+$: 940.1006 (calcd.), 940.1018 (found).

General Procedure for the Palladium-catalysed Cross-Coupling Reaction 10a-c: Under an inert atmosphere *N*-aryl sydnone methide was dissolved in anhyd. THF (28.0 ml/mmol) and was cooled to -78 °C. Then a 2.8 M solution of BuLi (1.2 equiv.) in *n*-hexan was added and the mixture was stirred for 30 minutes. Afterwards copper(I)-iodid was added and the solution was stirred for additional 30 minutes. The mixture was then allowed to warm to room temperature and 4-fluoroiodobenzene (1.5 equiv.) and tetrakis(triphenylphosphine)palladium(0) (0.05 equiv.) were added. The mixture was stirred for several hours (tlc monitoring). Then water was added and the aqueous phase was extracted three times with dichloromethane. The combined organic phases were dried over $MgSO_4$ and the solution was filtered through celite. The crude product was purified by a flash column chromatography.

5,5-(Dicyanomethylene)-4-(4''-fluorophenyl)-3-phenylsydnone (10a): 5-Dicyanomethylene)-3-phenylsydnone (100.0 mg, 0.48 mmol), *n*BuLi (0.20 mL, 0.56 mmol), CuI (102.0 mg, 0.71 mmol), 4-fluoroiodobenzene (158.0 mg, 0.71 mmol) and tetrakis(triphenylphospine)palladium(0) (28.0 mg, 0.02 mmol) were used for the synthesis. As eluent during flash chromatography DCM, EE and PE (2:2:3) were used. The product was isolated as yellow solid in 54 % yield (78.0 mg, 0.26 mmol). Mp.: 186 °C. ^{1}H NMR (600 MHz, DMSO-d$_6$): δ = 7.68-7.58 (m, 5H; 2'-H, 3'-H, 4'-H, 5'-H, 6'-H), 7.53-7.49 (m, 2H; 3'-H, 5'-H), 7.30-7.26 (m, 2H; 2''-H, 6''-H) ppm. ^{13}C NMR (150 MHz, DMSO-d$_6$): δ = 171.2 (o, 1C; C-5), 164.0 (o, d, $J_{C,F}$ = 249.6 Hz, 1C; C-4''), 134.7 (+, d, $J_{C,F}$ = 9.1 Hz, 2C, C-2'', C-6''), 133.3 (+, 1C; C-4'), 131.6 (o, 1C; C-1'), 130.5 (+, 2C, C-3', C-5'), 126.4 (+, 2C; C-2'', C-6''), 122.8 (o, 1C; C-4), 117.7 (o, d, $J_{C,F}$ = 3.1 Hz, 1C; C-1''), 116.3 (+, d, $J_{C,F}$ = 22.5 Hz, 2C; C-3'', C-5''), 115.7 (o, 2C; C-7, C-8), 34.4 (o, 1C; C-6) ppm. IR (ATR): $\tilde{\nu}$ = 2173, 1583, 1518, 1467, 1438, 1278, 1227, 1162, 1002, 907, 847, 761, 723, 684, 612, 549, 523, 508 cm^{-1}. HRMS (EI) $[C_{17}H_9FN_4ONa]^+$: 327.0653 (calcd.), 327.0645 (found).

(*E*)-5-(1-Cyano-2-methoxy-2-oxoethylidene)-4-(4''-fluorophenyl)-3-phenylsydnone (10b): (*E*)-5-(1-Cyano-2-methoxy-2-oxoethylidene)-3-phenylsydnone (80.0 mg, 0.33 mmol), *n*BuLi (0.14 mL, 0.39 mmol), CuI (71.0 mg, 0.49 mmol), 4-fluoroiodobenzene (109.0 mg, 0.49 mmol) and tetrakis(triphenylphospine)palladium(0) (19.0 mg, 0.02 mmol) were used for the synthesis. As eluent during flash chromatography DCM, EE and PE (5:3:3) were used. The product was isolated as yellow solid in 46 % yield (51.0 mg, 0.15 mmol). Mp.: 149 °C. ^{1}H NMR (600 MHz, DMSO-d$_6$): δ = 7.66-7.48 (m, 7H; 2'-H, 2''-H, 3'-H, 4'-H, 5'-H, 6'-H, 6''-H), 7.24-7.21 (m, 2H; 3''-H, 5''-H), 3.58 (s, 3H; 8-H) ppm. ^{13}C NMR (150 MHz, DMSO-d$_6$): δ = 165.1 (o, 1C; C-5), 163.7 (o, d, $J_{C,F}$ = 248.8 Hz, 1C; C-4''), 134.8 (+, d, $J_{C,F}$ = 6.8 Hz, 2C, C-2'', C-6''), 132.9 (+, 1C; C-4'), 132.1 (o, 1C; C-1''), 130.3 (+, 2C, C-3', C-5'), 126.6 (+, 2C; C-2', C-6'), 123.1 (o, 1C; C-4), 119.3 (o, 1C; C-1''), 117.5 (o, 1C; C-7), 115.9 (+, d, $J_{C,F}$ = 19.8 Hz, 2C; C-3'', C-5''), 68.7 (o, 1C, C-6), 51.2 (o, 1C; C-8) ppm. IR (ATR): $\tilde{\nu}$ = 2992, 2191, 1673, 1560, 1515, 1433, 1268, 1227, 1140, 1068, 1001, 921, 840, 757, 688, 598, 525 cm^{-1}. HRMS (EI) $[C_{18}H_{12}FN_3O_3Na]^+$: 360.0755 (calcd.), 360.0752 (found).

(*E*)-5-(Cyano(methylsulfonyl)methylene)-4-(4''-fluorophenyl)-3-phenylsydnone (10c): ((*E*)-5-(Cyano(methylsulfonyl)methylene))-3-phenylsydnone (100.0 mg, 0.38 mmol), *n*BuLi (0.16 mL, 0.45 mmol), CuI (82.0 mg, 0.57 mmol), 4-fluoroiodobenzene (127.0 mg, 0.57 mmol) and tetrakis(triphenylphospine)palladium(0) (22.0 mg, 0.02 mmol) were used for the synthesis. As eluent during flash chromatography DCM, EE and PE (5:1:1) were used. The product was isolated as yellow solid in 59 % yield (80.0 mg, 0.22 mmol). Mp.: 263 °C. ^{1}H NMR (600 MHz, DMSO-d$_6$): δ = 7.67-7.64 (m, 3H; 3'-H, 4'-H, 5'-H), 7.60-7.57 (m, 2H; 2'-H, 6'-H), 7.52-7.50 (m, 2H; 2''-H, 6''-H), 7.27-7.24 (m, 2H; 3''-H, 5''-H) 3.19 (s, 3H; 8-H) ppm. ^{13}C NMR (150 MHz, DMSO-d$_6$): δ = 167.8 (o, 1C; C-5), 163.9 (o, d, $J_{C,F}$ = 249.6 Hz, 1C; C-4''), 134.9 (+, d, $J_{C,F}$ = 9.1 Hz, 2C, C-2'', C-6''), 133.2 (+, 1C; C-4'), 131.9 (o, 1C; C-1'), 130.4 (+, 2C, C-3', C-5'), 126.5 (+, 2C; C-2'', C-6''), 121.6 (o, 1C; C-4), 118.4 (o, d, $J_{C,F}$ = 2.8 Hz, 1C; C-1''), 116.1 (+, d, $J_{C,F}$ = 22.2 Hz, 2C; C-3'', C-5''), 115.1 (o, 1C; C-7), 68.6 (o, 1C, C-6), 45.1 (o, 1C; C-8) ppm. IR (ATR): $\tilde{\nu}$ = 2926, 2178, 1569, 1513, 1472, 1444, 1303, 1235, 1161, 1125, 1004, 962, 898, 838, 760, 685, 602, 565, 540, 522, 492 cm^{-1}. HRMS (EI) $[C_{17}H_{12}FN_3O_3SNa]^+$: 380.0476 (calcd.), 380.0474 (found).

S3. Calculations

Density-functional theory (DFT) calculations were carried out by using the multithreaded Firefly 8.2.0 QC package,[1] which is partially based on the GAMESS (US)[2] source code, running on Windows 10 Pro (Version 10.0.17763.914) (x86_64) on an 16 core AMD 2950X processor workstation. MM2 optimized structures were used as starting geometries. Minima were proven to be true minima by the absence of imaginary frequencies. Rotational barriers have been calculated as a relaxed scan calculation with a fixed dihedral angle of -O-C=C-Z varied with a step size of 10° from 180° to -180°. Solvent effects in CH_2Cl_2, THF and DMSO were estimated by help of the polarizable continuum model implemented in Firefly. Molecular plots were obtained using Jmol 14.27.2.[3]

[1] Alex A. Granovsky, Firefly version 8, http://classic.chem.msu.su/gran/firefly/index.html.

[2] M. W. Schmidt, K. K. Baldridge, J. A. Boatz, S. T. Elbert, M. S. Gordon, J. H. Jensen, S. Koseki, N. Matsunaga, K. A. Nguyen, S. Su, T. L. Windus, M. Dupuis, and J. A. Montgomery. *J. Comput. Chem*. **1993**, *14*, 1347-1363.

[3] an open-source Java viewer for chemical structures in 3D. http://jmol.sourceforge.net/ (accessed Nov 2019).]

Calculation of 2a (B3LYP / 6-311++G)**

Charge: 0

Number of imaginary frequencies: 0

E: -717.0614310864 H

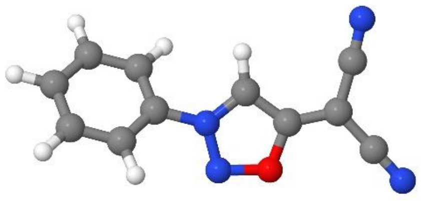

ATOM	ATOMIC CHARGE	COORDINATES (BOHR) X	Y	Z
C	6.0	5.7004533437	6.1125137946	-2.0708393670
C	6.0	6.8410372310	3.7634991830	-1.7228859853
C	6.0	5.3751880139	1.6487633677	-1.1793724057
C	6.0	2.7685054656	1.9393749000	-0.9752196232
C	6.0	1.5904974421	4.2660510590	-1.3218558659
C	6.0	3.0840519340	6.3579929977	-1.8767966193
N	7.0	1.2439934803	-0.2427485489	-0.3968651631
N	7.0	-0.9396663159	-0.4992656423	-1.5304835198
O	8.0	-1.9032395570	-2.7134426257	-0.5452048845
C	6.0	-0.2455529025	-3.7656648091	1.1404213708
C	6.0	1.8196890958	-2.1088776642	1.2345202832
C	6.0	-0.7576289982	-6.0168388506	2.3846170518
C	6.0	1.0971371939	-6.9407032766	4.0781007883
N	7.0	2.6842142318	-7.5995071574	5.4349846176
C	6.0	-3.0385511084	-7.3527240432	1.9819844446
N	7.0	-4.9070707272	-8.4394941996	1.6460119265
H	1.0	6.8509620726	7.7511444508	-2.4982462837
H	1.0	8.8709715864	3.5656165117	-1.8928611811
H	1.0	6.2387871840	-0.1931867018	-0.9603701553
H	1.0	-0.4406463379	4.4224315651	-1.1491972589
H	1.0	2.1984979257	8.1835648110	-2.1454098497
H	1.0	3.5008064645	-2.1366075054	2.3706406374

Calculation of 3a (B3LYP / 6-311++G)**

Charge: -1

Number of imaginary frequencies: 0

E: -716.5141718208 H

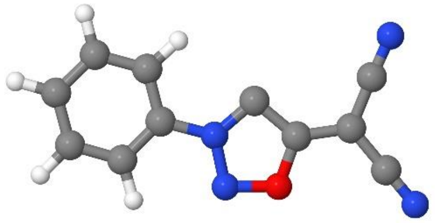

ATOM	ATOMIC CHARGE	COORDINATES (BOHR) X	Y	Z
C	6.0	5.6921215413	6.1253979473	-2.2113140483
C	6.0	6.7966400054	3.9901038016	-1.1270931325
C	6.0	5.3205957158	1.9284050398	-0.4294572695
C	6.0	2.7156592745	2.0087297385	-0.8222708598
C	6.0	1.5850153466	4.1332222097	-1.9051821954
C	6.0	3.0867277862	6.1836336373	-2.5944087771
N	7.0	1.2186787091	-0.1290002642	-0.0947943316
N	7.0	-1.2820185494	0.0320951085	-0.5396150746
O	8.0	-2.1249403363	-2.2490783352	0.3506330134
C	6.0	-0.1299526862	-3.6261860135	1.2607043284

C	6.0	2.1485959279	-2.2598856789	0.9919285816
C	6.0	-0.6259528818	-6.0549225012	2.2686218828
C	6.0	1.3584806479	-7.5542897906	3.2335746240
N	7.0	2.9330949419	-8.8510803292	4.0476591902
C	6.0	-3.0986217224	-7.0472460356	2.3419867202
N	7.0	-5.1342176972	-7.8739086189	2.4068761359
H	1.0	6.8504631849	7.7279707394	-2.7509971530
H	1.0	8.8219785468	3.9240314173	-0.8200201960
H	1.0	6.1090990595	0.2420587989	0.4170512175
H	1.0	-0.4369103494	4.1598616789	-2.1942290344
H	1.0	2.2071849967	7.8338105202	-3.4340273227

Rotational energy plot of 2e (PBE0-d3 / 6-31G)**

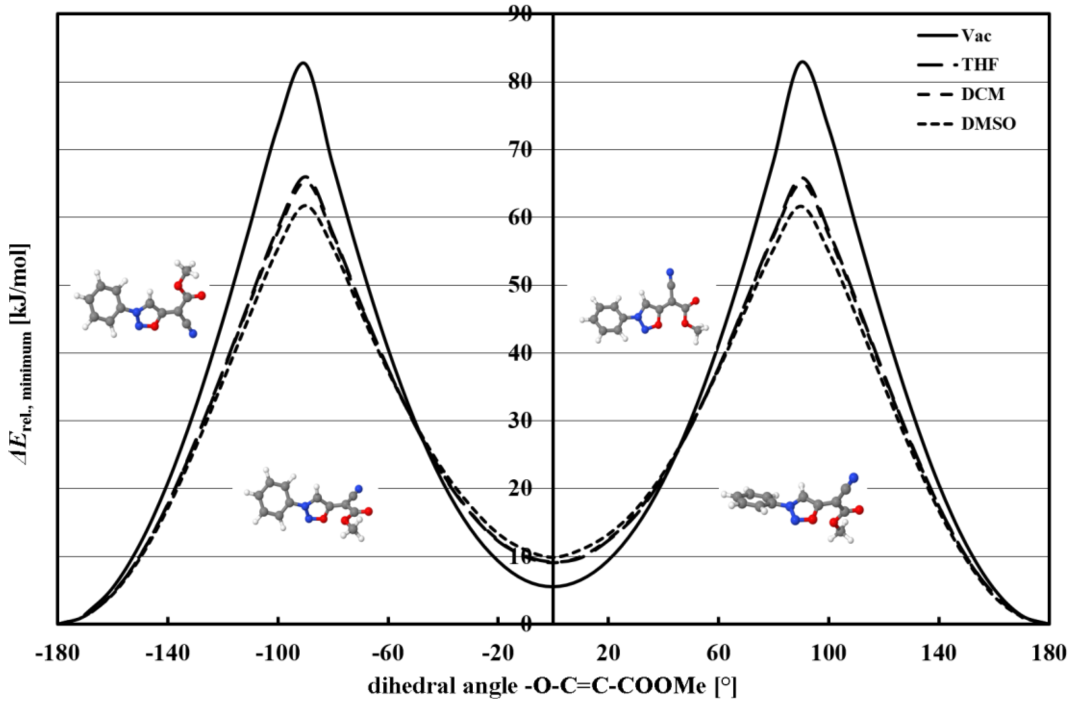

Calculation of (*E*)-2e in vacuo (PBE0-d3 / 6-31G)**

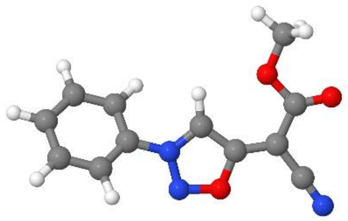

Charge: 0

Number of imaginary frequencies: 0

E: -851.5786802225 H

ATOM	ATOMIC CHARGE	COORDINATES (BOHR) X	Y	Z
C	6.0	-5.4258931453	8.0504298260	-2.0421344272
C	6.0	-4.3424942603	5.7109375443	-2.5712936471
C	6.0	-5.6997862704	3.5009198481	-2.1547772221
C	6.0	-8.1366655880	3.6855574289	-1.1879895569
C	6.0	-9.2556140008	6.0018533224	-0.6473880452
C	6.0	-7.8765107717	8.1920459018	-1.0912091231
N	7.0	-9.5368335942	1.4215672649	-0.7330512202
N	7.0	-11.9596117754	1.4305963763	-1.1561646792
O	8.0	-12.7107325568	-0.9349986924	-0.5121233389
C	6.0	-10.7166519753	-2.3859927724	0.2745205142
C	6.0	-8.6128727921	-0.7837374544	0.1198653281
C	6.0	-11.1061320886	-4.8689284951	1.0108863141
C	6.0	-13.5899937771	-5.8621761056	0.9431887654
C	6.0	-9.0768534683	-6.5225257823	1.8579522704
O	8.0	-9.3242167284	-8.6993806747	2.5166711135
O	8.0	-6.7847800843	-5.3426204738	1.8370726864
C	6.0	-4.7486587656	-6.9025515959	2.6532907534
N	7.0	-15.6511369594	-6.6245124116	0.8678567231
H	1.0	-4.3578804104	9.7694248652	-2.3764817809
H	1.0	-2.4411671061	5.6001769166	-3.3311449633

H	1.0	-4.9015243811	1.6690269010	-2.6149406514
H	1.0	-11.1582734119	6.0625985687	0.1102768578
H	1.0	-8.7193683079	10.0148719430	-0.6769395824
H	1.0	-6.6758392629	-1.0788824395	0.6433345827
H	1.0	-3.0576505703	-5.7289012808	2.5437471094
H	1.0	-5.0510206148	-7.5507597822	4.5909138773
H	1.0	-4.5574222611	-8.5582275818	1.4333081333

Calculation of (*Z*)-2e in vacuo (PBE0-d3 / 6-31G)**

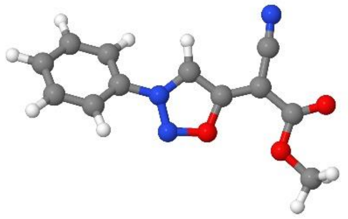

Charge: 0

Number of imaginary frequencies: 0

E: -851.5765720342 H

ATOM	ATOMIC CHARGE	COORDINATES (BOHR) X Y Z		
C	6.0	-7.8382683841	9.1946627744	-1.4621453540
C	6.0	-8.0893884198	8.1422062650	0.9369432205
C	6.0	-8.2882291821	5.5388855395	1.2241721430
C	6.0	-8.2508711864	4.0302642612	-0.9276533269
C	6.0	-8.0008812070	5.0409578232	-3.3412852337
C	6.0	-7.7865843746	7.6462060694	-3.5905401096
N	7.0	-8.4792521475	1.3534539764	-0.6487996707
N	7.0	-7.2148477353	-0.1052445171	-2.1775408630
O	8.0	-7.8614364264	-2.4872669750	-1.4561662605
C	6.0	-9.4973118620	-2.4483688524	0.5380749478
C	6.0	-9.8956132169	0.1221292200	1.0519273861
C	6.0	-10.4914419746	-4.5744486935	1.7044838323
C	6.0	-12.1448767453	-4.0484623238	3.7422794483
C	6.0	-10.0062264459	-7.2063231808	1.0425278883
O	8.0	-10.9196671427	-9.0078746852	2.1240521650
O	8.0	-8.4021494319	-7.4186868233	-0.9310964079
C	6.0	-7.8625816005	-9.9536448165	-1.6483608561
N	7.0	-13.4691268941	-3.4152566731	5.3827204622
H	1.0	-7.6794728081	11.2287677553	-1.6734961033
H	1.0	-8.1121180456	9.3449451342	2.5974455670
H	1.0	-8.4258976201	4.6920482401	3.0867145581
H	1.0	-7.9879857159	3.7987539136	-4.9704559209
H	1.0	-7.5947167016	8.4672712839	-5.4597645963
H	1.0	-11.1249348636	1.0318660535	2.3922761118
H	1.0	-6.5678697607	-9.8185539650	-3.2449451061
H	1.0	-6.9842274487	-10.9876708270	-0.0898886026
H	1.0	-9.5893093990	-10.9463973187	-2.1980708476

Calculation of (*E*)-2e in THF (PBE0-d3 / 6-31G)**

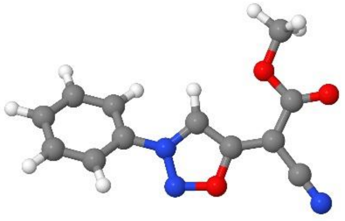

Charge: 0

Number of imaginary frequencies: 0

E: -851.6029143499 H

ATOM	ATOMIC CHARGE	COORDINATES (BOHR) X Y Z		
C	6.0	-5.4312958722	8.0598576696	-2.0653100284
C	6.0	-4.3561343034	5.7177159919	-2.6070831702
C	6.0	-5.7120203574	3.5094255054	-2.1781531343
C	6.0	-8.1408929053	3.7097723795	-1.1918767235
C	6.0	-9.2555478604	6.0254994654	-0.6334324178
C	6.0	-7.8720604667	8.2114496097	-1.0872236907
N	7.0	-9.5394811005	1.4426698365	-0.7309403961
N	7.0	-11.9598838960	1.4350315635	-1.1365039686
O	8.0	-12.6954711286	-0.9329955827	-0.4899455131
C	6.0	-10.6927544987	-2.3624259979	0.2733526635
C	6.0	-8.6007218532	-0.7662404802	0.1123177620

C	6.0	-11.0894779323	-4.8675811204	1.0039774754
C	6.0	-13.5614796996	-5.8613257288	0.9299323366
C	6.0	-9.0815966808	-6.5353248973	1.8529577242
O	8.0	-9.3823692704	-8.7278456193	2.4919723930
O	8.0	-6.8047488202	-5.3869232131	1.8634438145
C	6.0	-4.7487948259	-6.9274185019	2.7007228792
N	7.0	-15.6158501035	-6.6583082736	0.8605340344
H	1.0	-4.3610967242	9.7740093407	-2.4042777624
H	1.0	-2.4646356149	5.6028905633	-3.3840100516
H	1.0	-4.9197318923	1.6772982322	-2.6392633164
H	1.0	-11.1418781480	6.0987565884	0.1627073091
H	1.0	-8.7006770268	10.0345855659	-0.6555630005
H	1.0	-6.6512425876	-1.0442966720	0.6010481812
H	1.0	-3.0795638345	-5.7301220439	2.5995204863
H	1.0	-5.0636553237	-7.5639576295	4.6387560736
H	1.0	-4.5296829714	-8.5737875867	1.4755397327

Calculation of (*Z*)-2e in THF (PBE0-d3 / 6-31G)**

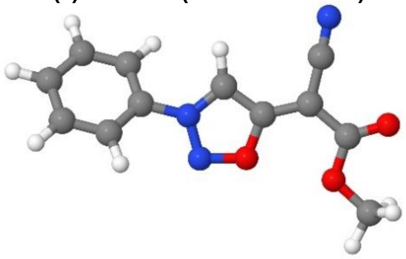

Charge: 0
Number of imaginary frequencies: 0
E: -851.5994138674 H

ATOM	ATOMIC CHARGE	COORDINATES (BOHR) X Y Z		
C	6.0	-6.8466875121	8.2936942701	-3.9978989218
C	6.0	-9.0395124800	6.9718516323	-4.6134677586
C	6.0	-9.5872666050	4.6939190689	-3.4256123723
C	6.0	-7.8912846506	3.7799001160	-1.6366483336
C	6.0	-5.6869058972	5.0620452771	-0.9923273138
C	6.0	-5.1817575374	7.3435872174	-2.1914927110
N	7.0	-8.4388252365	1.4282096522	-0.4202543033
N	7.0	-7.8467645928	1.1458052006	1.9465294031
O	8.0	-8.5713668475	-1.2512764848	2.5023866737
C	6.0	-9.6318943772	-2.3964221709	0.4616903281
C	6.0	-9.5331769741	-0.6063299657	-1.4744191251
C	6.0	-10.5502275749	-4.8733447851	0.5014312685
C	6.0	-11.5758575322	-5.7828302850	-1.7867020366
C	6.0	-10.5479788008	-6.5982660054	2.6408450172
O	8.0	-11.3639323061	-8.7535534535	2.5561267052
O	8.0	-9.5519419546	-5.5683331417	4.7377002438
C	6.0	-9.5125506135	-7.1881912586	6.8971110743
N	7.0	-12.4056948554	-6.4579349433	-3.7148973167
H	1.0	-6.4328280420	10.0707039014	-4.9306582887
H	1.0	-10.3418833798	7.7180440080	-6.0074544706
H	1.0	-11.3154457132	3.6762108435	-3.8457948660
H	1.0	-4.3972849796	4.2710437155	0.3891720880
H	1.0	-3.4714571281	8.3695800021	-1.7228406319
H	1.0	-10.0748612988	-0.7031803194	-3.4320714562
H	1.0	-8.6625574714	-6.0780130647	8.4074406650
H	1.0	-11.4280563827	-7.7599638027	7.4140758916
H	1.0	-8.3842159310	-8.8763762031	6.5217698918

Calculation of (*E*)-2e in DCM (PBE0-d3 / 6-31G)**

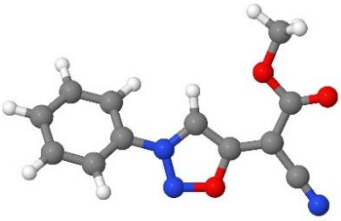

Charge: 0
Number of imaginary frequencies: 0
E: -851.6038071042 H

ATOM	ATOMIC CHARGE	COORDINATES (BOHR) X Y Z		
C	6.0	-5.4339490477	8.0594910627	-2.0641667441
C	6.0	-4.3605524831	5.7178369344	-2.6119208691
C	6.0	-5.7157657945	3.5092894451	-2.1821895893

C	6.0	-8.1413861238	3.7093131760	-1.1876890904
C	6.0	-9.2541570220	6.0241974441	-0.6225494850
C	6.0	-7.8715880352	8.2104102603	-1.0780434011
N	7.0	-9.5397059779	1.4419857556	-0.7271363775
N	7.0	-11.9609326940	1.4356702909	-1.1265866859
O	8.0	-12.6961552095	-0.9320242634	-0.4785013317
C	6.0	-10.6924691501	-2.3620348246	0.2797040330
C	6.0	-8.6002191860	-0.7673289624	0.1147706265
C	6.0	-11.0893683282	-4.8673486841	1.0124377792
C	6.0	-13.5612321455	-5.8604583445	0.9448120401
C	6.0	-9.0815626658	-6.5354439501	1.8599232547
O	8.0	-9.3847692226	-8.7269839042	2.5029233559
O	8.0	-6.8043330805	-5.3904740085	1.8639408125
C	6.0	-4.7467671497	-6.9319028220	2.6970284646
N	7.0	-15.6157310507	-6.6584273263	0.8807541039
H	1.0	-4.3648516101	9.7739035161	-2.4050034173
H	1.0	-2.4715217769	5.6036766894	-3.3947455857
H	1.0	-4.9252593412	1.6775854706	-2.6475422066
H	1.0	-11.1379456280	6.0971030780	0.1795428792
H	1.0	-8.6985076212	10.0334706275	-0.6430303368
H	1.0	-6.6483342991	-1.0490682305	0.5919075759
H	1.0	-3.0788155029	-5.7330076557	2.5981466554
H	1.0	-5.0618241791	-7.5732456334	4.6333382288
H	1.0	-4.5272414452	-8.5746927655	1.4672192686

Calculation of (*Z*)-2e in DCM (PBE0-d3 / 6-31G)**

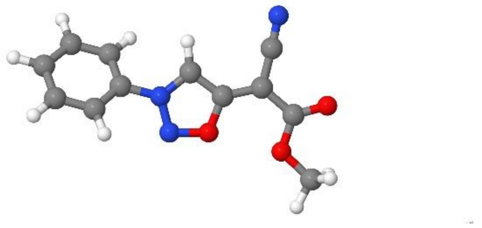

Charge: 0

Number of imaginary frequencies: 0

E: -851.6003601141 H

ATOM	ATOMIC CHARGE	COORDINATES (BOHR) X	Y	Z
C	6.0	-7.6206002804	9.2382360794	-1.0758834442
C	6.0	-7.5864793855	8.0637089315	1.2810094357
C	6.0	-7.8488243943	5.4561760064	1.4627273896
C	6.0	-8.1545423971	4.0769631732	-0.7545638624
C	6.0	-8.1965906931	5.2051542365	-3.1299061384
C	6.0	-7.9188368575	7.8131406958	-3.2710308855
N	7.0	-8.4503015433	1.3952396005	-0.5783128862
N	7.0	-7.4196505836	-0.0329813901	-2.2892879277
O	8.0	-8.0819806934	-2.4166214535	-1.6119269364
C	6.0	-9.4904805021	-2.4124829533	0.5364630114
C	6.0	-9.7288864603	0.1267987333	1.2116564868
C	6.0	-10.4368534560	-4.6120580229	1.6550108023
C	6.0	-11.8812051487	-4.2739331067	3.8712079029
C	6.0	-10.1033867147	-7.1890454148	0.7624988224
O	8.0	-11.0108993405	-9.0384107697	1.8005159345
O	8.0	-8.6837035068	-7.3299169386	-1.3383304982
C	6.0	-8.3094829319	-9.8255572900	-2.2978502768
N	7.0	-13.0606285770	-3.9098565817	5.6986921190
H	1.0	-7.4158125499	11.2732613569	-1.2024459617
H	1.0	-7.3391104562	9.1711867067	2.9863171885
H	1.0	-7.7735112493	4.5117608088	3.2798445681
H	1.0	-8.4696088751	4.0660235487	-4.8106909154
H	1.0	-7.9579541883	8.7316912120	-5.1017918867
H	1.0	-10.7485411036	1.0375465703	2.7173449102
H	1.0	-7.1092366114	-9.6089228664	-3.9558203902
H	1.0	-7.3829029694	-11.0177722745	-0.8892068489
H	1.0	-10.1141014618	-10.6805827727	-2.8243166370

Calculation of (*E*)-2e in DMSO (PBE0-d3 / 6-31G)**

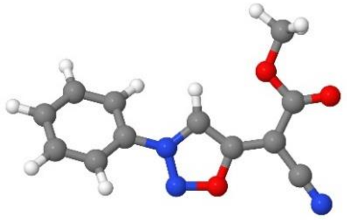

Charge: 0

Number of imaginary frequencies: 0

E: -851.6082282003 H

ATOM	ATOMIC CHARGE	COORDINATES (BOHR) X	Y	Z
C	6.0	-5.4297614146	8.0610557560	-2.0560617087
C	6.0	-4.3493369586	5.7184019625	-2.5868064089
C	6.0	-5.7066062920	3.5099999821	-2.1632205184
C	6.0	-8.1424689369	3.7127883824	-1.1943900592
C	6.0	-9.2632182588	6.0283038190	-0.6458876027
C	6.0	-7.8771003663	8.2139402687	-1.0937300177
N	7.0	-9.5409380793	1.4448600291	-0.7378038814
N	7.0	-11.9588086418	1.4306889729	-1.1520620838
O	8.0	-12.6918730901	-0.9364046486	-0.5001046807
C	6.0	-10.6891451218	-2.3566302079	0.2726213395
C	6.0	-8.6004270559	-0.7608755477	0.1150767621
C	6.0	-11.0890621926	-4.8637109613	1.0111452066
C	6.0	-13.5570161665	-5.8593075013	0.9389916837
C	6.0	-9.0852381831	-6.5332008452	1.8612876370
O	8.0	-9.4014139303	-8.7240661671	2.5118201865
O	8.0	-6.8095902986	-5.3977022109	1.8510037474
C	6.0	-4.7390230521	-6.9309881946	2.6712223646
N	7.0	-15.6081022264	-6.6689587700	0.8727114295
H	1.0	-4.3575667158	9.7747425545	-2.3891089308
H	1.0	-2.4526963252	5.6031702428	-3.3498853773
H	1.0	-4.9094253260	1.6773379165	-2.6114087533
H	1.0	-11.1520221979	6.1030405975	0.1441596472
H	1.0	-8.7075745272	10.0373559044	-0.6681466867
H	1.0	-6.6502504814	-1.0262384491	0.6084067747
H	1.0	-3.0739626862	-5.7340299975	2.5277638058
H	1.0	-5.0193299077	-7.5426755339	4.6219753056
H	1.0	-4.5408550322	-8.5882439915	1.4577649688

Calculation of (Z)-2e in DMSO (PBE0-d3 / 6-31G)**

Charge: 0

Number of imaginary frequencies: 0

E: -851.6044626098 H

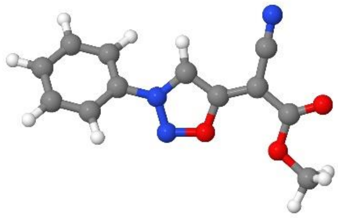

ATOM	ATOMIC CHARGE	COORDINATES (BOHR) X	Y	Z
C	6.0	-7.7124107345	9.2500052937	-1.0920330437
C	6.0	-7.6153789671	8.0665680871	1.2586388578
C	6.0	-7.8679370843	5.4578635318	1.4368853848
C	6.0	-8.2287746188	4.0864344805	-0.7770081396
C	6.0	-8.3364871182	5.2238247307	-3.1458119632
C	6.0	-8.0662638412	7.8327825092	-3.2840000759
N	7.0	-8.5105384533	1.4030611769	-0.6034405745
N	7.0	-7.5234003274	-0.0146982898	-2.3486234383
O	8.0	-8.1517285949	-2.4039319426	-1.6583008155
C	6.0	-9.4939594879	-2.4136583629	0.5317802701
C	6.0	-9.7283592267	0.1225241728	1.2198767955
C	6.0	-10.3856380985	-4.6217882227	1.6777933405
C	6.0	-11.7558161511	-4.2971824072	3.9427415957
C	6.0	-10.0543464320	-7.1965192817	0.7775145862
O	8.0	-10.8861604087	-9.0549137480	1.8620624247
O	8.0	-8.7279816796	-7.3242024068	-1.3840524218
C	6.0	-8.3519337396	-9.8183801102	-2.3469737074
N	7.0	-12.8758020236	-3.9445670711	5.8093639295
H	1.0	-7.5143825543	11.2858488226	-1.2159688419
H	1.0	-7.3262414213	9.1680119668	2.9612707585
H	1.0	-7.7439124690	4.5071177517	3.2480215802

H	1.0	-8.6542596840	4.0914479240	-4.8231290927
H	1.0	-8.1553946636	8.7581984004	-5.1095397638
H	1.0	-10.7048903199	1.0220753825	2.7604779090
H	1.0	-7.2730380719	-9.5858341926	-4.0842518469
H	1.0	-7.2984813445	-10.9701549556	-0.9950031660
H	1.0	-10.1630359198	-10.7286347286	-2.7410609731

Calculated HOMOs and LUMOs and their energies

HOMO-LUMO DFT calculations were performed using the Spartan Software (***Spartan'18*** or ***Spartan'20***, Wavefunction, Inc., Irvine, CA. Available from: http://www.wavefun.com) running on a MS Windows 10 Pro PC system with an AMD Ryzen Threadripper 3970X 32-Core and 128 GB RAM or with an Intel Core i7-6950X (decacore) and 64 GB RAM in both cases utilizing the appropriate message passing interface MS-MPI 10.0.12498.5. MMFF optimized structures were used as starting geometries for the geometry optimizations with the B3LYP density functional and the 6-311++G** basis set carried out as vacuum calculations. Subsequent frequency calculations of all final structures evidenced the absence of imaginary frequencies and thus the presence of true minima on the potential energy surface. In case of anions additional calculations were performed taking into account THF as the solvent. This was accomplished using ORCA 4.2.1 (a) F. Neese, The ORCA Program System, *WIREs Comput. Mol. Sci.* **2012**, 2: 73-78; doi: 10.1002/wcms.81), b) F. Neese, Software Update: The ORCA Program System, Version 4.0. *Wiley Interdiscip. Rev. Comput. Mol. Sci.* **2018**, *8*, e1327, doi: 10.1002/wcms.1327) with the abovementioned functional and diffuse basis set and the implemented C-PCM solvent model.

Geometry optimization and energy of 2e (B3LYP / 6-311++G)**

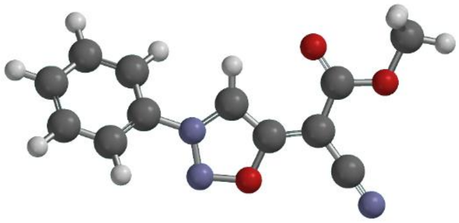

Charge: 0

Number of imaginary frequencies: 0

E: -852.741303666602 H

Atom	Coordinates (Angstroms)		
C	0.4433932961	1.3040246850	-4.7408191137
C	0.0686751833	-0.0373473437	-4.6739660228
C	0.0450715972	-0.6998471655	-3.4505815418
C	0.4077663591	0.0031553772	-2.3045228151
C	0.7937086235	1.3400587724	-2.3508205719
C	0.8041057361	1.9895318004	-3.5817919203
N	0.3870481962	-0.6739391257	-1.0334206365
N	0.7404991485	-1.9345922376	-0.9892074807
O	0.5901042387	-2.2741680032	0.3344637818
C	0.1623742224	-1.2000448066	1.0725373209
C	0.0276635826	-0.1484529343	0.1548933430
C	-0.0587642955	-1.3024530356	2.4395210180
H	0.4566489087	1.8150903396	-5.6962920367
H	-0.2125739644	-0.5704581234	-5.5741026806
H	-0.2499691944	-1.7381885223	-3.3768591367
H	1.0973069958	1.8569936155	-1.4492650290
H	1.1037006163	3.0293073713	-3.6337045704
H	-0.3220175406	0.8545444567	0.3013791301
C	0.1494603728	-2.5348317552	3.1018277150
N	0.3256657603	-3.5525593197	3.6266691846
C	-0.5052931604	-0.1088008884	3.1475965357
O	-0.6921186187	0.9810819264	2.6258784735
O	-0.6947855070	-0.3360717474	4.4628229036
C	-1.1358836295	0.7851107434	5.2438176532
H	-2.0850680134	1.1681540070	4.8657980335
H	-0.3928016295	1.5842853851	5.2236548104
H	-1.2539172842	0.4004165289	6.2544936529

Geometry optimization and energy of 2i (B3LYP / 6-311++G)**

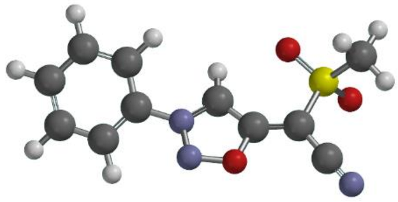

Charge: 0

Number of imaginary frequencies: 0

E: -1212.75852371011 H

Atom	Coordinates (Angstroms)		
C	0.2447903020	4.3877587839	-2.0700457648
C	-0.2844072404	4.5144645197	-0.7861364752
C	-0.3644736499	3.4084797812	0.0543930206
C	0.1004400303	2.1819328623	-0.4132554551
C	0.6404130675	2.0357374213	-1.6881359332
C	0.7048390717	3.1507794386	-2.5186871961
N	0.0237648406	1.0296847422	0.4484156525
N	0.1963228035	1.1954569990	1.7349370082
O	0.0365734973	-0.0696038573	2.2539216007
C	-0.2114660737	-0.9840331335	1.2591909467
C	-0.2252866332	-0.2393422326	0.0696725999
C	-0.4046633270	-2.3178786422	1.5796238577
C	-0.3788918095	-2.7849236577	2.9043025945
N	-0.3365043160	-3.2069413160	3.9845013926
S	-0.7495062634	-3.5053715045	0.2957837648
O	-0.5651657290	-2.8027350603	-0.9890773680
O	-2.0093369893	-4.1965776561	0.5673354102
C	0.5731509904	-4.7234264413	0.4574986814
H	0.2992264530	5.2529062495	-2.7202367620
H	-0.6443224448	5.4749669502	-0.4374014126
H	-0.7819113307	3.4858790479	1.0495253929
H	1.0186080020	1.0771499930	-2.0206059018
H	1.1238046387	3.0512143354	-3.5126547747
H	-0.4329823965	-0.5468889657	-0.9363149432
H	0.3664924971	-5.4887663973	-0.2908599511
H	0.5349337282	-5.1470174900	1.4601646371
H	1.5255582813	-4.2329047697	0.2641453786

Geometry optimization and energy of 3e (B3LYP / 6-311++G)**

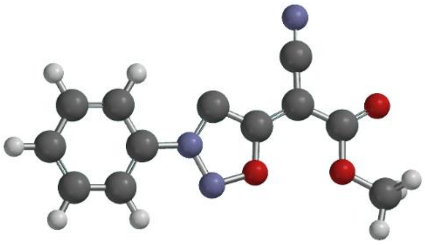

Charge: -1

Number of imaginary frequencies: 0

E: -852.175085564867 H

Atom	Coordinates (Angstroms)		
C	-2.5755541694	-0.0438847435	-4.3163242665
C	-1.1857598929	0.0579991107	-4.3487543036
C	-0.4484756731	0.0794019169	-3.1662713762
C	-1.1152689561	-0.0016524653	-1.9416789556
C	-2.5058913814	-0.1024095866	-1.8967278819
C	-3.2290825411	-0.1229194793	-3.0855655694
N	-0.3831778154	0.0151125042	-0.7104227776
N	0.9599512433	0.0319141970	-0.8067678454
O	1.3374301029	0.0433972669	0.5067419557
C	0.2309349383	0.0304678590	1.3150684675
C	-0.9454054885	0.0112634593	0.5241240599
C	0.3537616095	0.0399436760	2.7310927110
C	-0.8483452793	0.0560258581	3.4821033298
N	-1.8201093983	0.0705434011	4.1175988124
H	-3.1441568136	-0.0610738373	-5.2400896175
H	-0.6667846548	0.1217948584	-5.2993904612
H	0.6297367676	0.1579366699	-3.1807120904

H	-2.9769961897	-0.1591670548	-0.9238245924
H	-4.3103874202	-0.2021023037	-3.0479537098
C	1.5721404420	0.0303755164	3.5056023559
O	1.6529507508	0.0422469469	4.7268812632
O	2.7113149785	0.0011756968	2.7303331960
C	3.9365102869	-0.0197873777	3.4529810752
H	4.0434276472	0.8686384790	4.0815867585
H	4.7217162146	-0.0421657596	2.6962337133
H	4.0055206925	-0.9030748087	4.0941357491

Geometry optimization and energy of 3i (B3LYP / 6-311++G)**

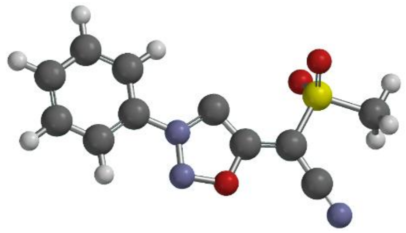

Charge: -1

Number of imaginary frequencies: 0

E: -1212.19654021879 H

Atom	Coordinates (Angstroms)		
C	0.5356816440	-0.0000004782	4.9239679430
C	-0.8205697760	-0.0000008928	4.6018515551
C	-1.2323926150	-0.0000006060	3.2706980253
C	-0.2716272056	0.0000000932	2.2575241340
C	1.0885234979	0.0000005822	2.5669433895
C	1.4847011678	0.0000002477	3.9008130157
N	-0.6641262608	0.0000003303	0.8783912250
N	-1.9863798590	0.0000001673	0.6269063087
O	-2.0109578410	0.0000002729	-0.7416820172
C	-0.7269802179	0.0000004843	-1.2284040051
C	0.2027763442	0.0000005258	-0.1666654264
C	-0.5578646391	0.0000006325	-2.6294448901
C	-1.6277020611	0.0000016259	-3.5227105139
N	-2.5066554203	0.0000022534	-4.2908857445
S	1.0899966838	0.0000006907	-3.3030823032
O	1.7996622575	-1.2592154893	-3.0082841110
O	1.7996613711	1.2592180381	-3.0082868676
C	0.7863453881	-0.0000014228	-5.0940756814
H	0.8510654001	-0.0000006805	5.9620458217
H	-1.5671662868	-0.0000014644	5.3890121437
H	-2.2815954135	-0.0000009682	3.0100178553
H	1.7944790434	0.0000011562	1.7461927842
H	2.5426827289	0.0000005943	4.1404978405
H	1.7902688763	-0.0000020991	-5.5189185500
H	0.2440866386	0.8983768422	-5.3812121635
H	0.2440865542	-0.8983804356	-5.3812097681

Calculated HOMOs and LUMOs of 2a

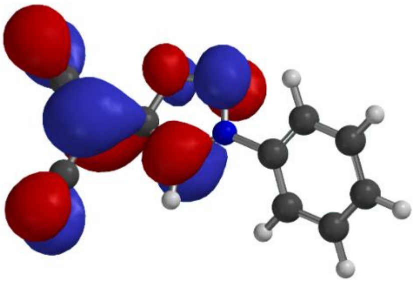

HOMO (-6.09 eV)

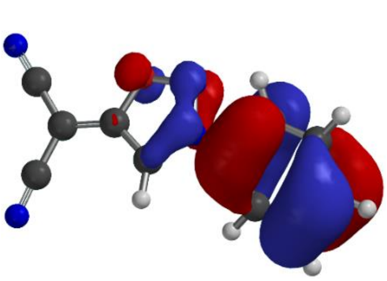

HOMO-1 (-8.51 eV)

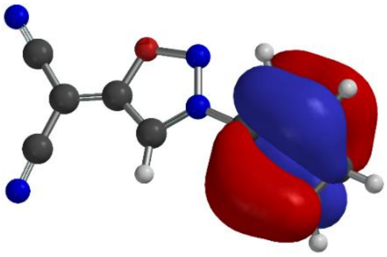

HOMO-2 (-8.56 eV)

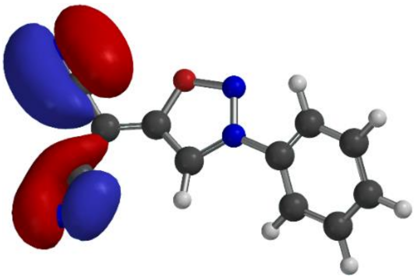

HOMO-3 (-8.94 eV)

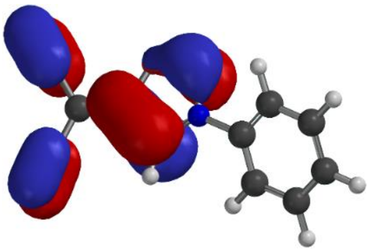

HOMO-4 (-9.04 eV)

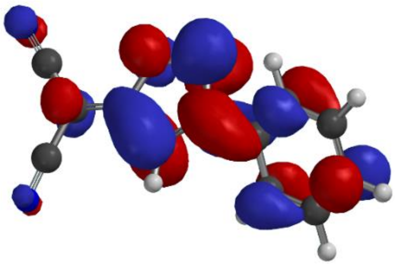

LUMO (-3.14 eV)

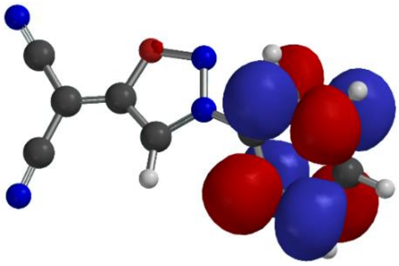

LUMO+1 (-1.84 eV)

Calculated HOMOs and LUMOs of 2e

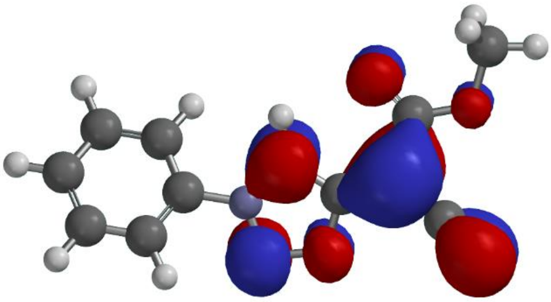

HOMO (-5.99 eV)

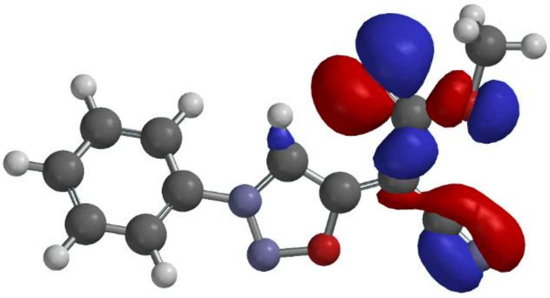

HOMO-1 (-7.72 eV)

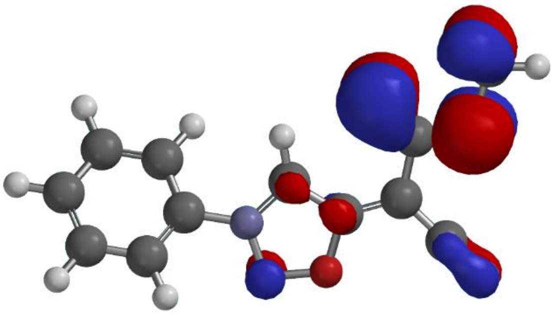

HOMO-2 (-8.32 eV)

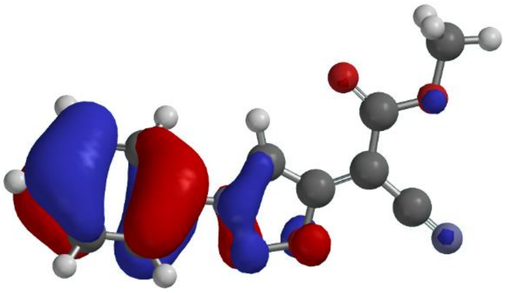

HOMO-3 (-8.34 eV)

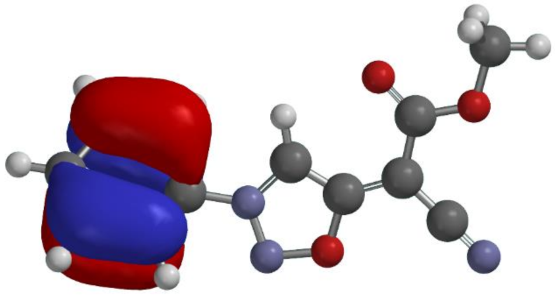

HOMO-4 (-8.41 eV)

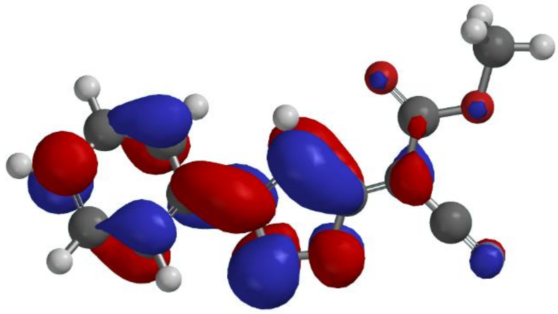

LUMO (-2.99 eV)

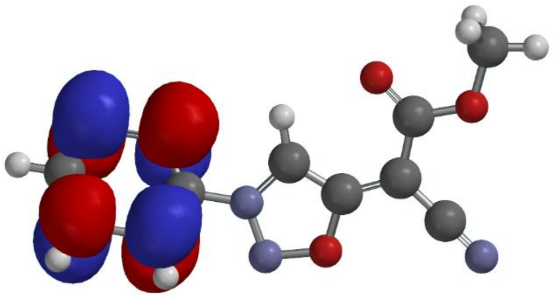

LUMO+1 (-1.77 eV)

Calculated HOMOs and LUMOs of 2i

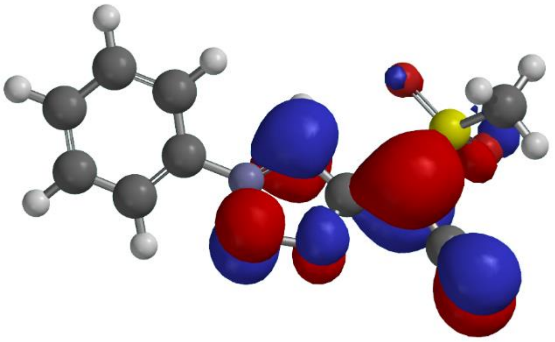

HOMO (-6.27 eV)

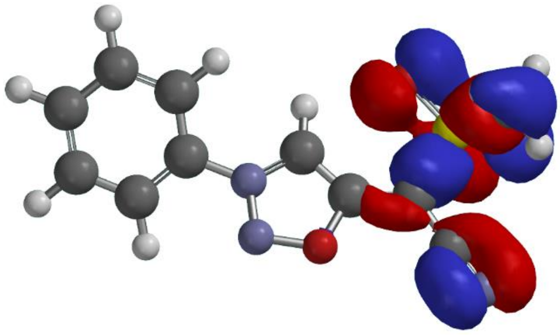

HOMO-1 (-8.19 eV)

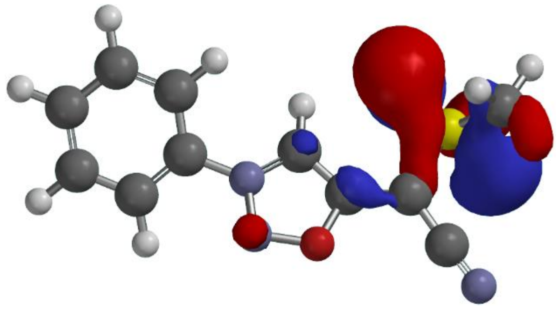

HOMO-2 (-8.41 eV)

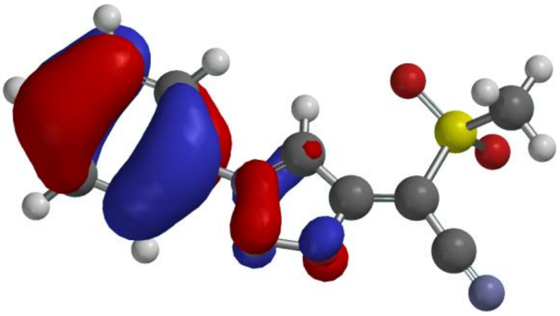

HOMO-3 (-8.44 eV)

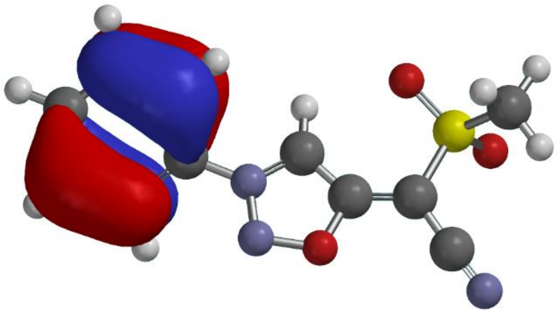

HOMO-4 (-8.47 eV)

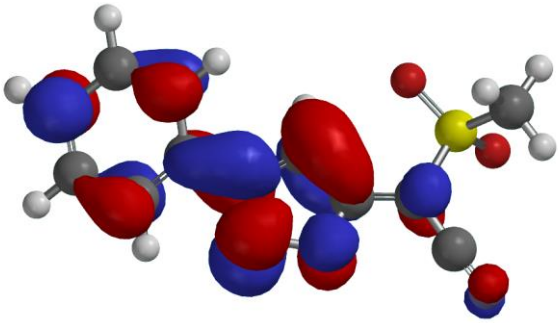

LUMO (-3.11 eV)

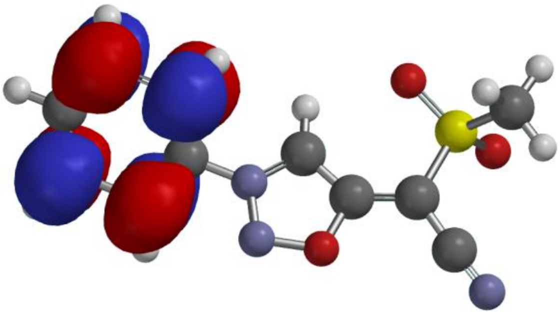

LUMO+1 (-1.83 eV)

Calculated HOMOs and LUMOs of 3a

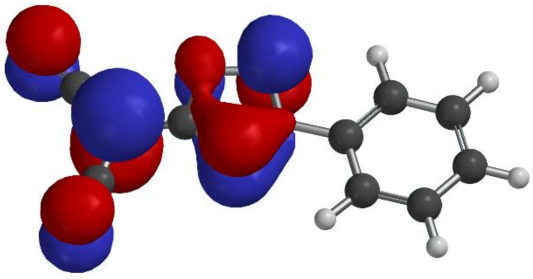

HOMO (-1.84 eV)

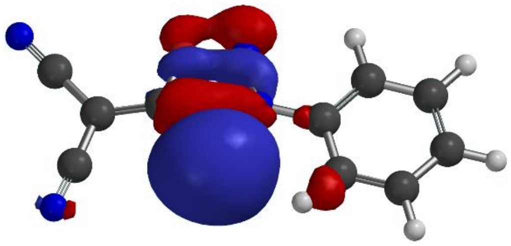

HOMO-1 (-2.99 eV)

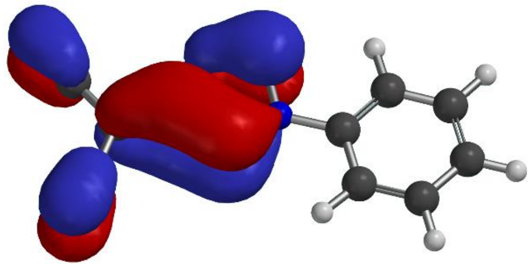

HOMO-2 (-4.39 eV)

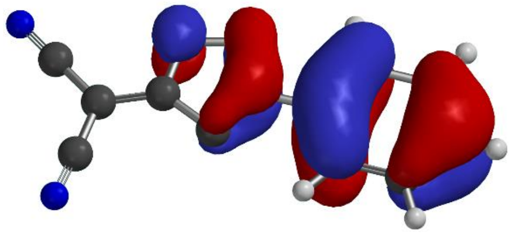

HOMO-3 (-4.58 eV)

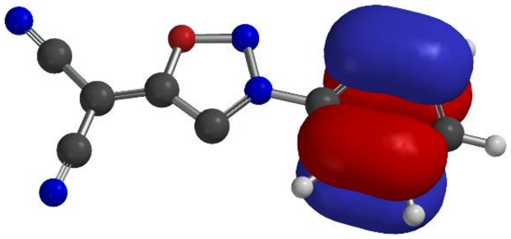

HOMO-4 (-5.03 eV)

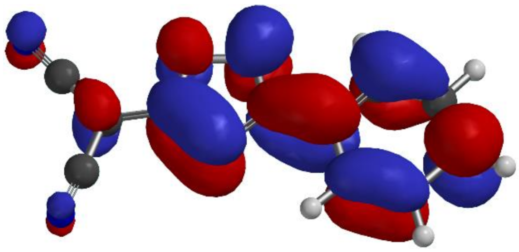

LUMO (+0.88 eV)

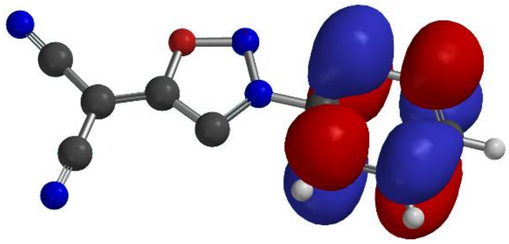

LUMO+1 (+1.55 eV)

Calculated HOMOs and LUMOs of 3e

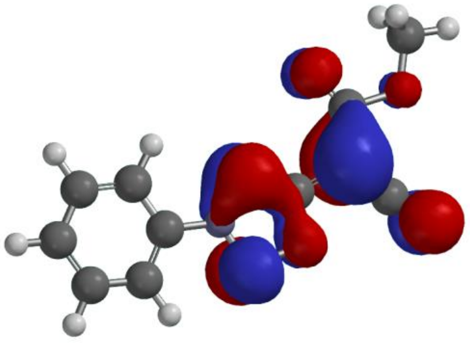

HOMO (-1.70 eV)

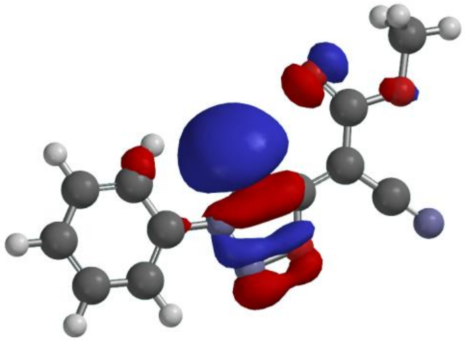

HOMO-1 (-2.74 eV)

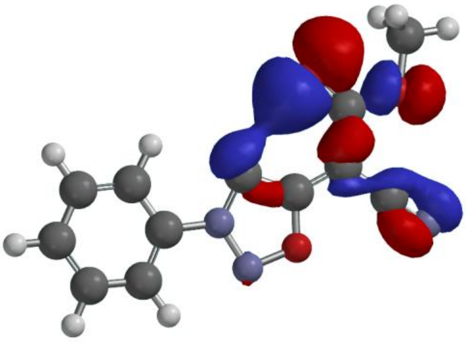

HOMO-2 (-4.03 eV)

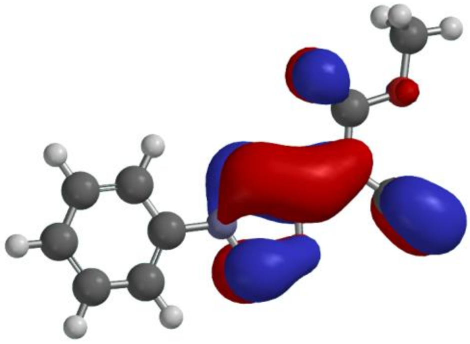

HOMO-3 (-4.20 eV)

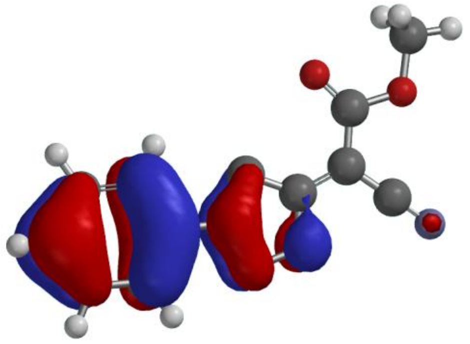

HOMO-4 (-4.44 eV)

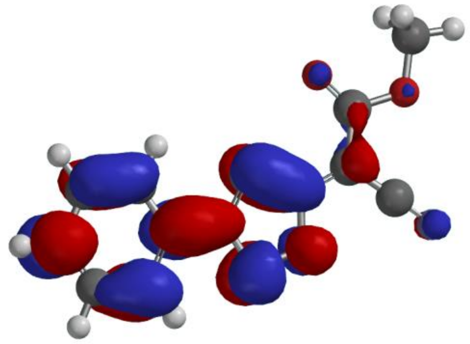

LUMO (+1.04 eV)

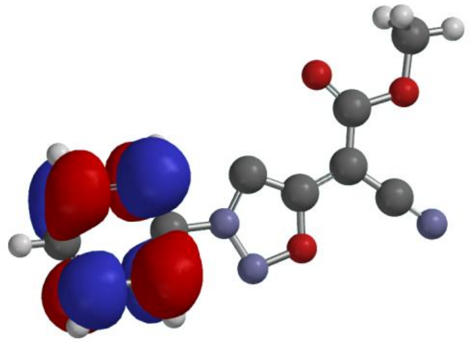

LUMO+1 (+1.61 eV)

Calculated HOMOs and LUMOs of 3i.

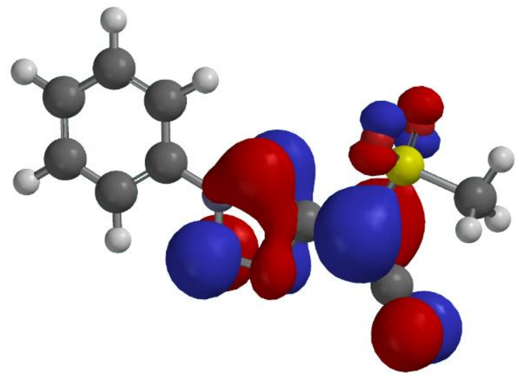

HOMO (-1.88 eV)

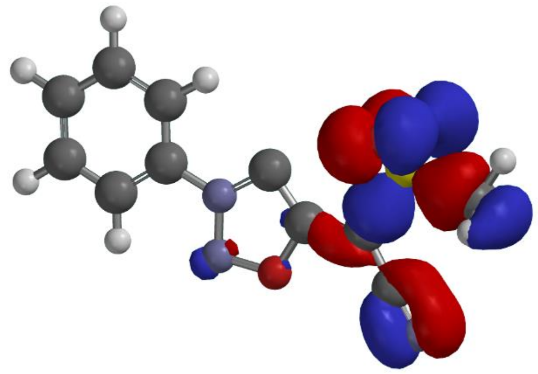

HOMO-4 (-4.60 eV)

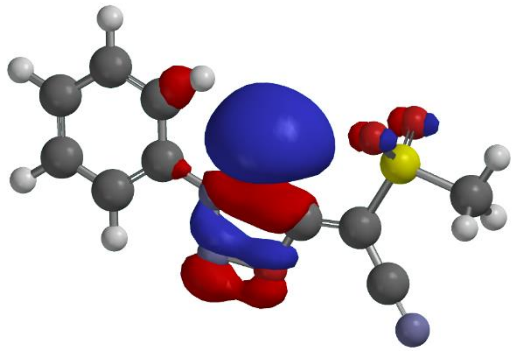

HOMO-1 (-2.84 eV)

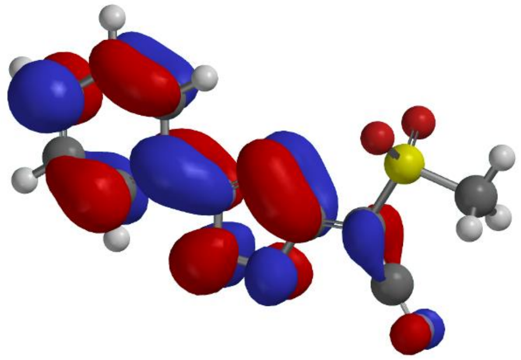

LUMO (+0.96 eV)

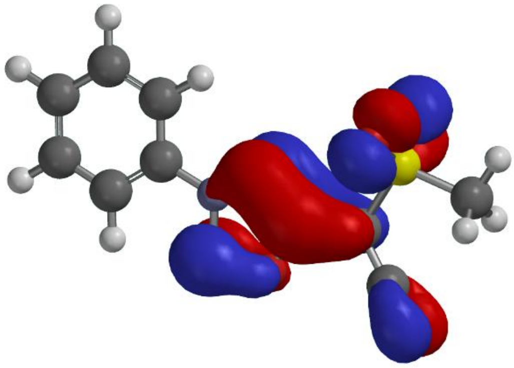

HOMO-2 (-4.32 eV)

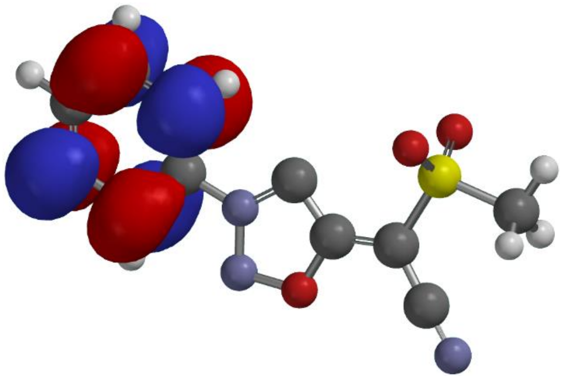

LUMO+1 (+1.61 eV)

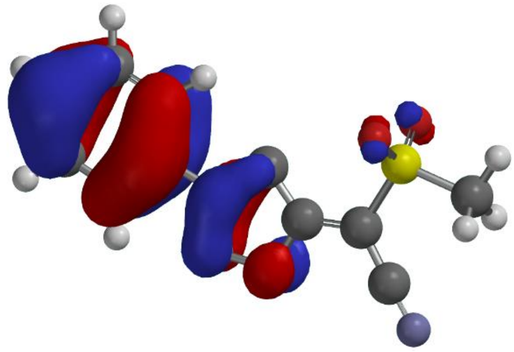

HOMO-3 (-4.49 eV)

Table S1: HOMO-LUMO values from DFT
taking the solvent THF into account (C-PCM model)

carbene	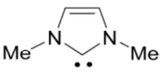	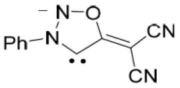	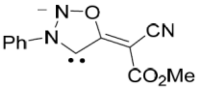	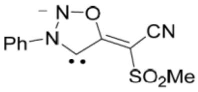	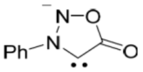
		**3a**	**3e**	**3i**	
**charge**	**0**	**-1**	**-1**	**-1**	**-1**
**MO**	**MO energy of given species with THF as solvent (C-PCM model, B3LYP / 6-311++G**)**				
**LUMO+1**	+0.27	-0.43	-0.39	-0.43	-0.26
**LUMO**	0.00	-1.58	-1.46	-1.55	-0.96
**HOMO**	-6.27	-4.91	-4.81	-5.01	-4.74
**HOMO-1**	-6.38	-5.97	-5.83	-5.96	-5.46
**HOMO-2**	-7.49	-6.90	-6.84	-6.90	-6.60
**HOMO-3**	-10.25	-7.05	-7.02	-7.05	-6.70
**HOMO-4**	-10.42	-7.43	-7.14	-7.52	-6.90

Calculated CREF value for compound 2a

Reactand **2a**:

Electronic energy: -717.06142472 H

Zero point energy: 0.15135079 H

Sum of electronic and ZPE: -716.91007393 H

Carbene **3a** from **2a**:

Electronic energy: -716.51416796 H

Zero point energy: 0.13796305 H

Sum of electronic and ZPE: -716.37620491 H

Resulting CREF value from energy sum: **0.534**

S4. NMR spectra: 2a ^{1}H-NMR (400 MHz)

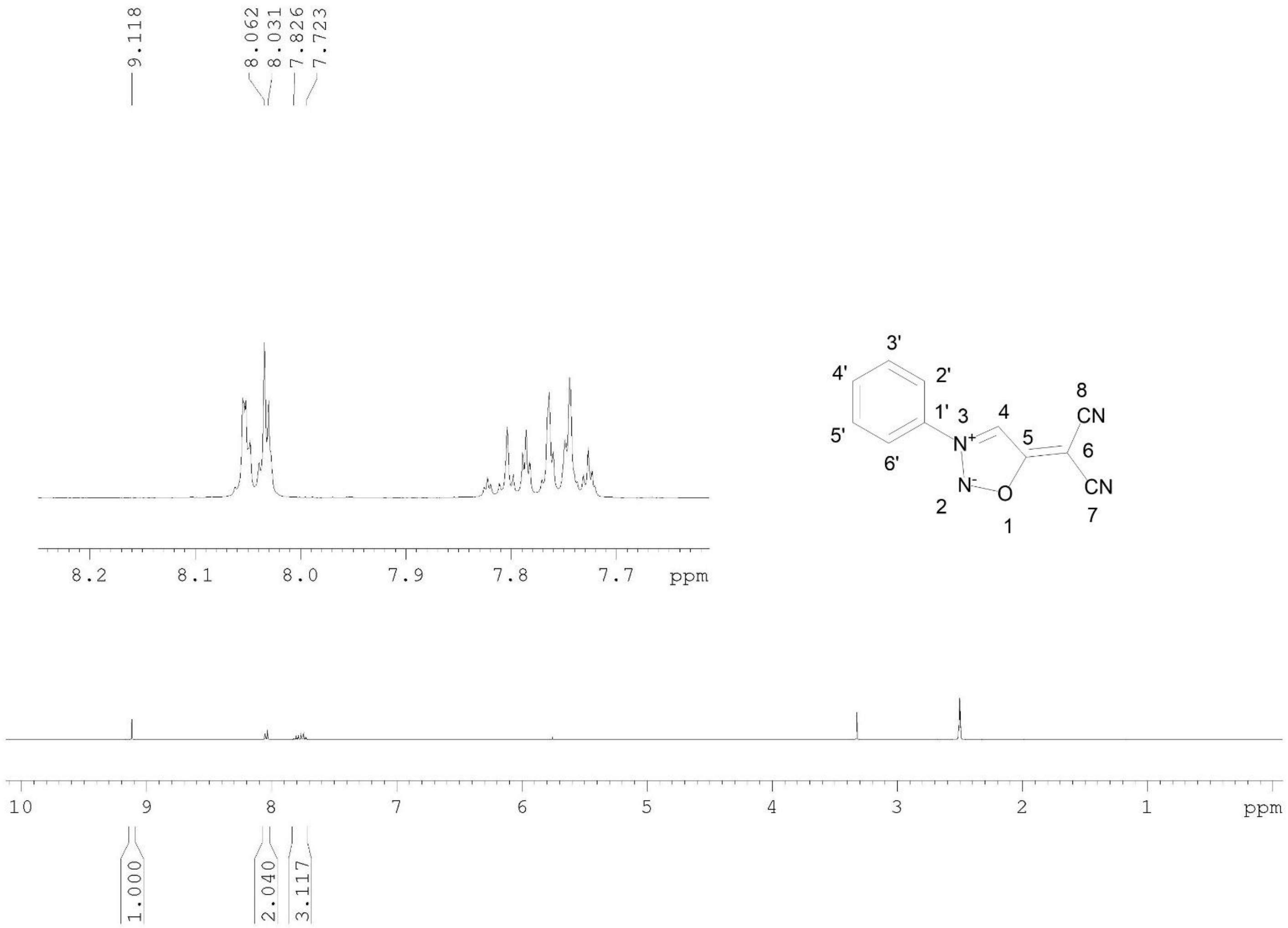

2a ^{15}N-NMR (zgig30, 61 MHz)

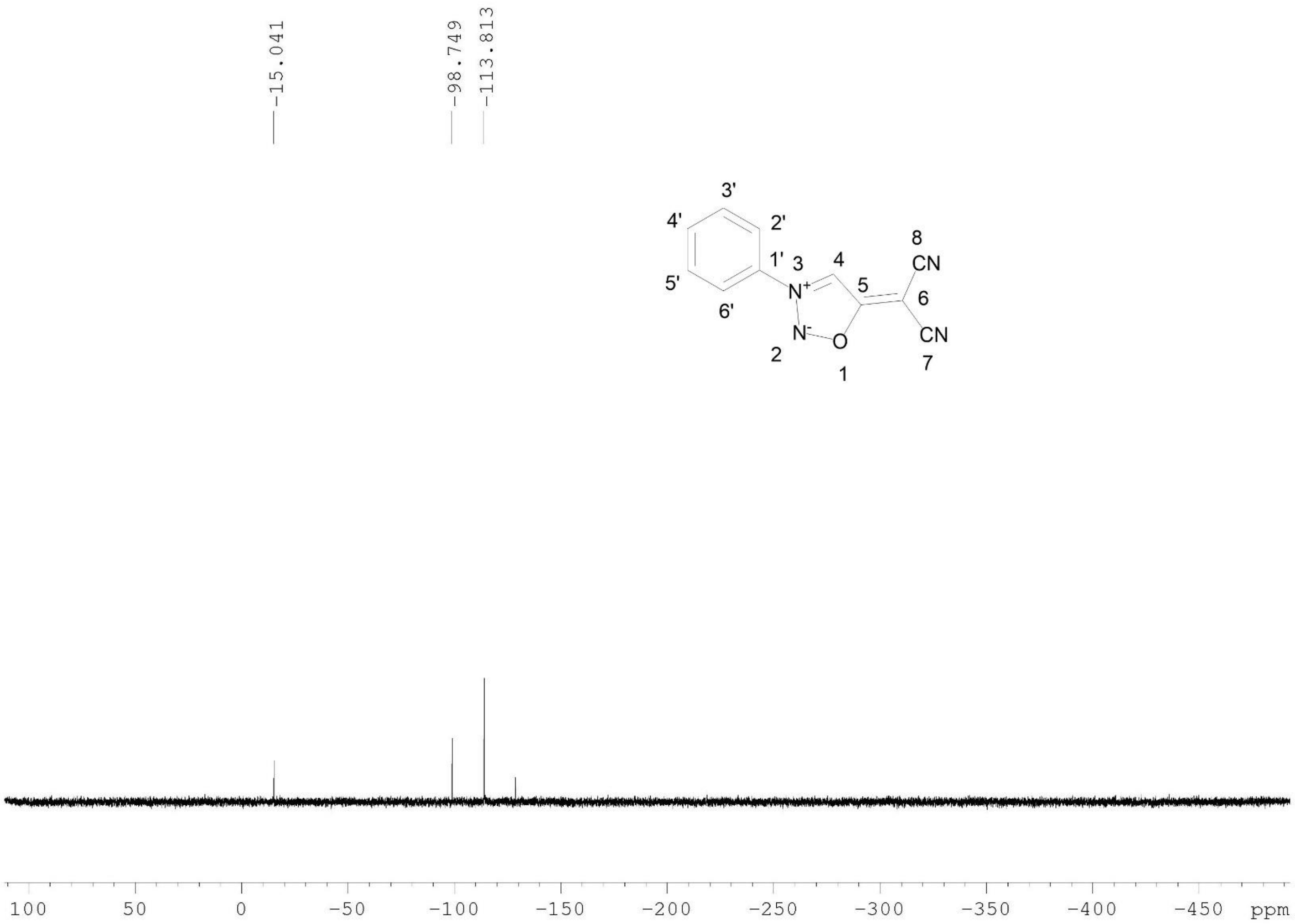

2b ^{1}H-NMR (600 MHz)

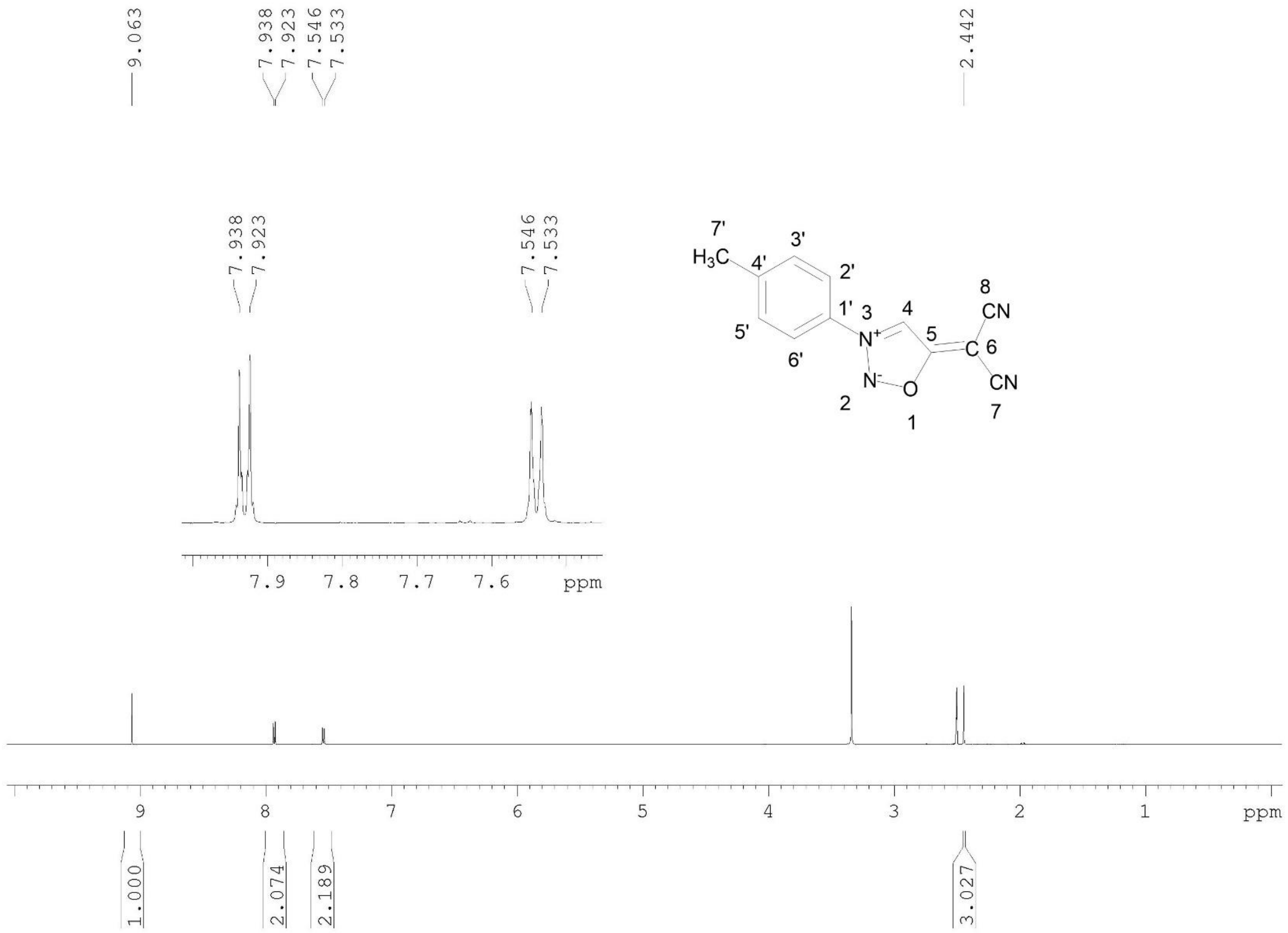

2b ^{13}C-NMR (150 MHz)

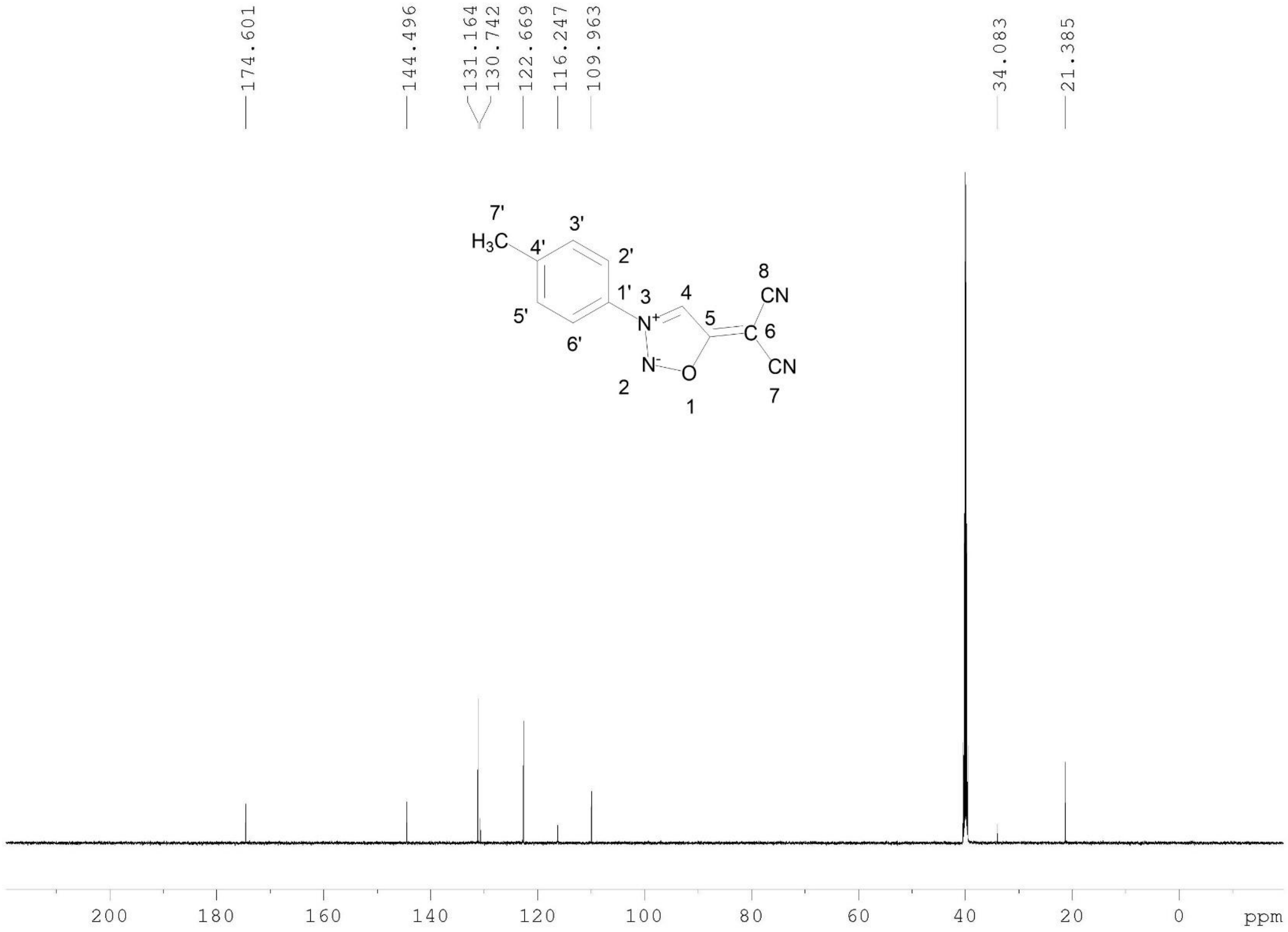

2b ^{13}C-NMR DEPT (150 MHz)

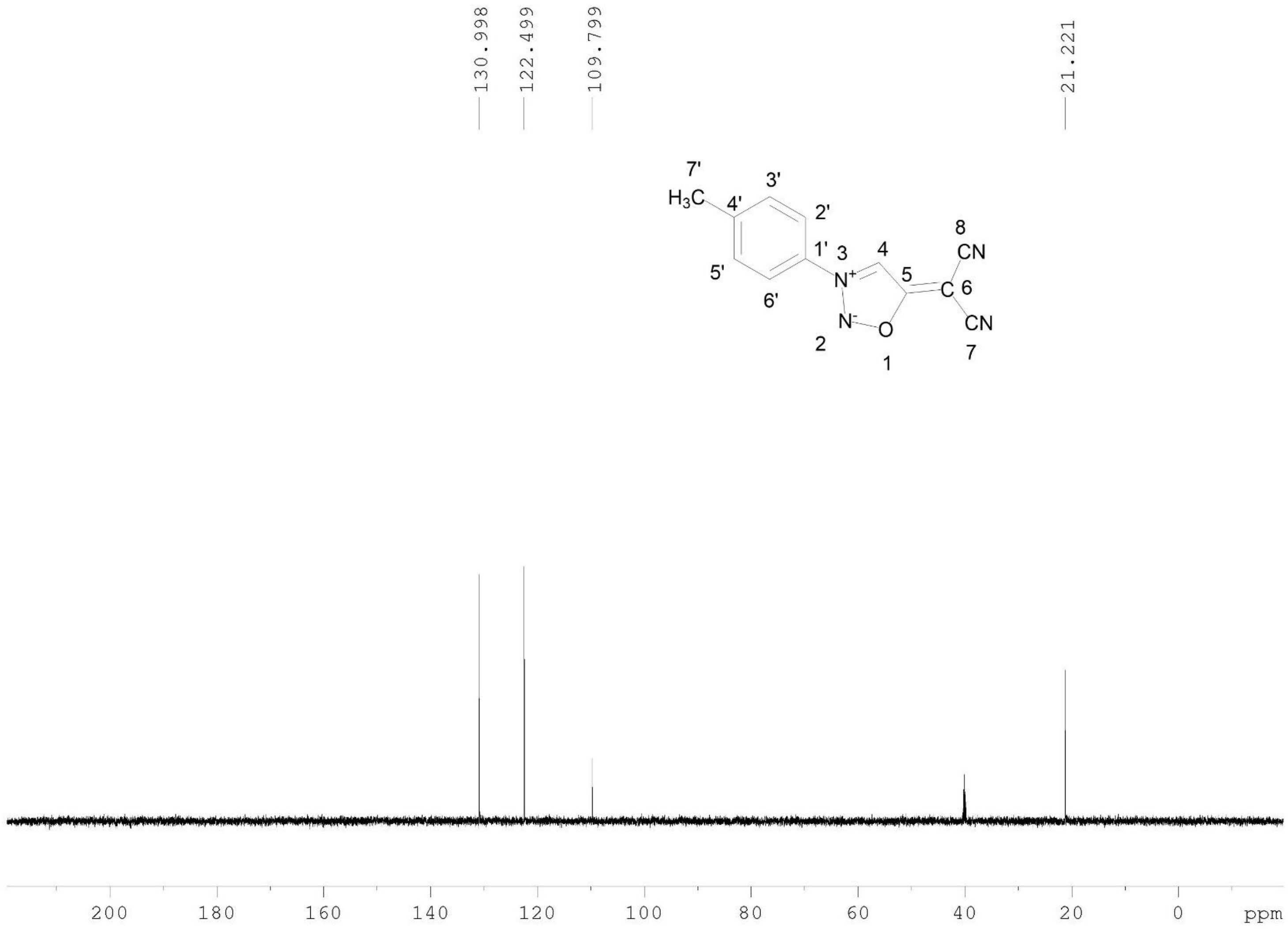

2c ^{1}H-NMR (600 MHz)

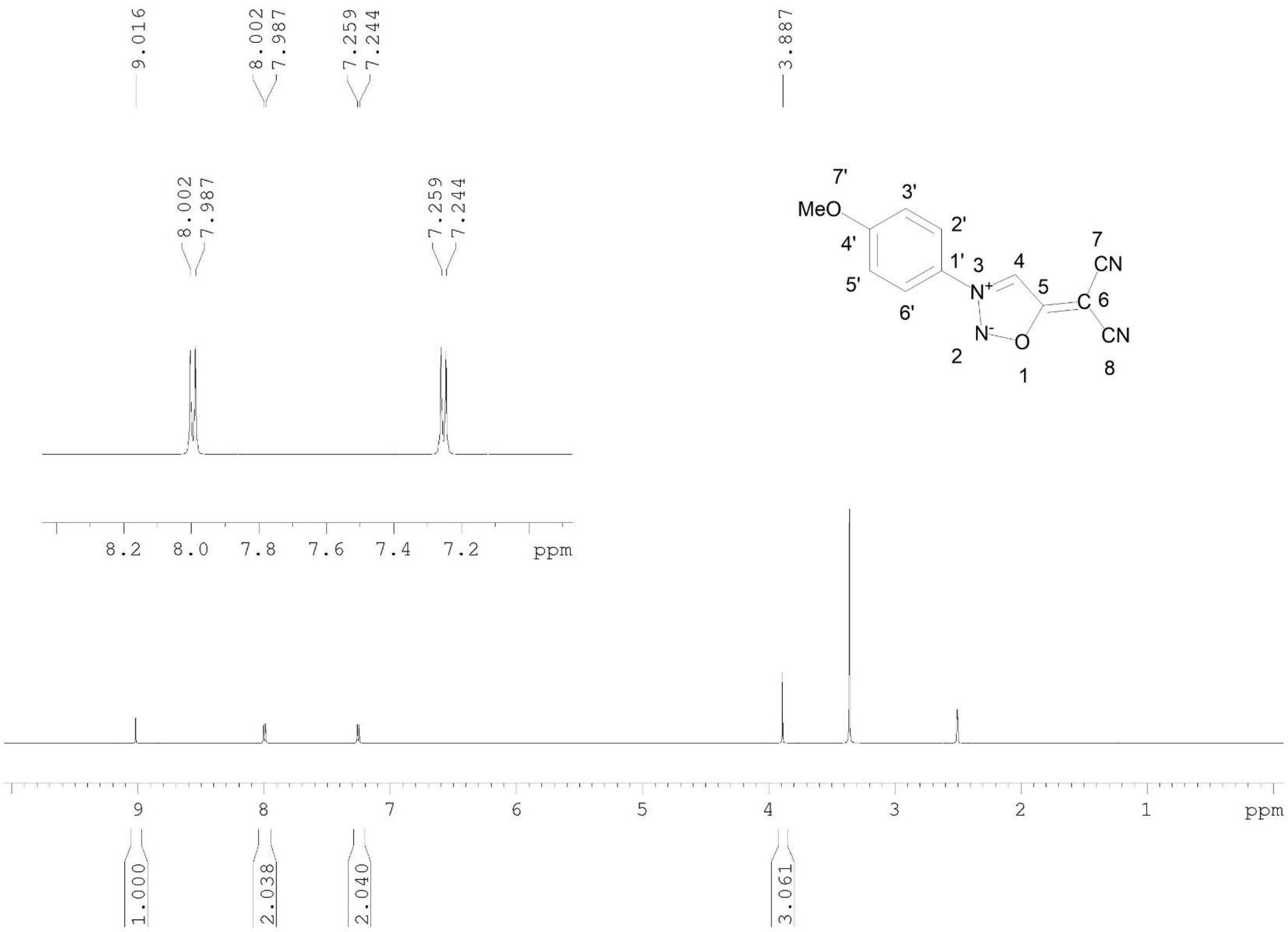

2c ^{13}C-NMR (150 MHz)

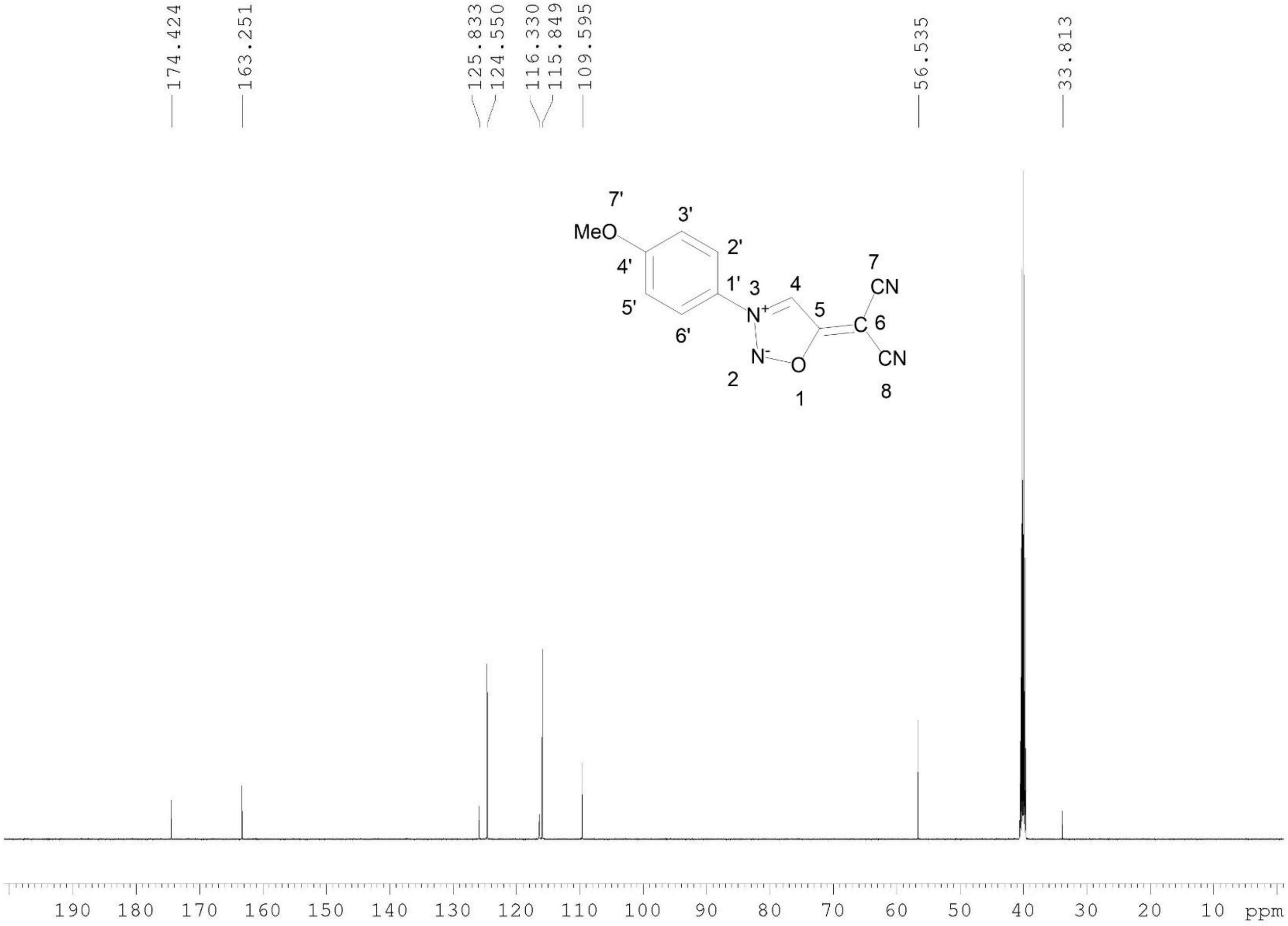

2c ^{13}C-NMR DEPT (150 MHz)

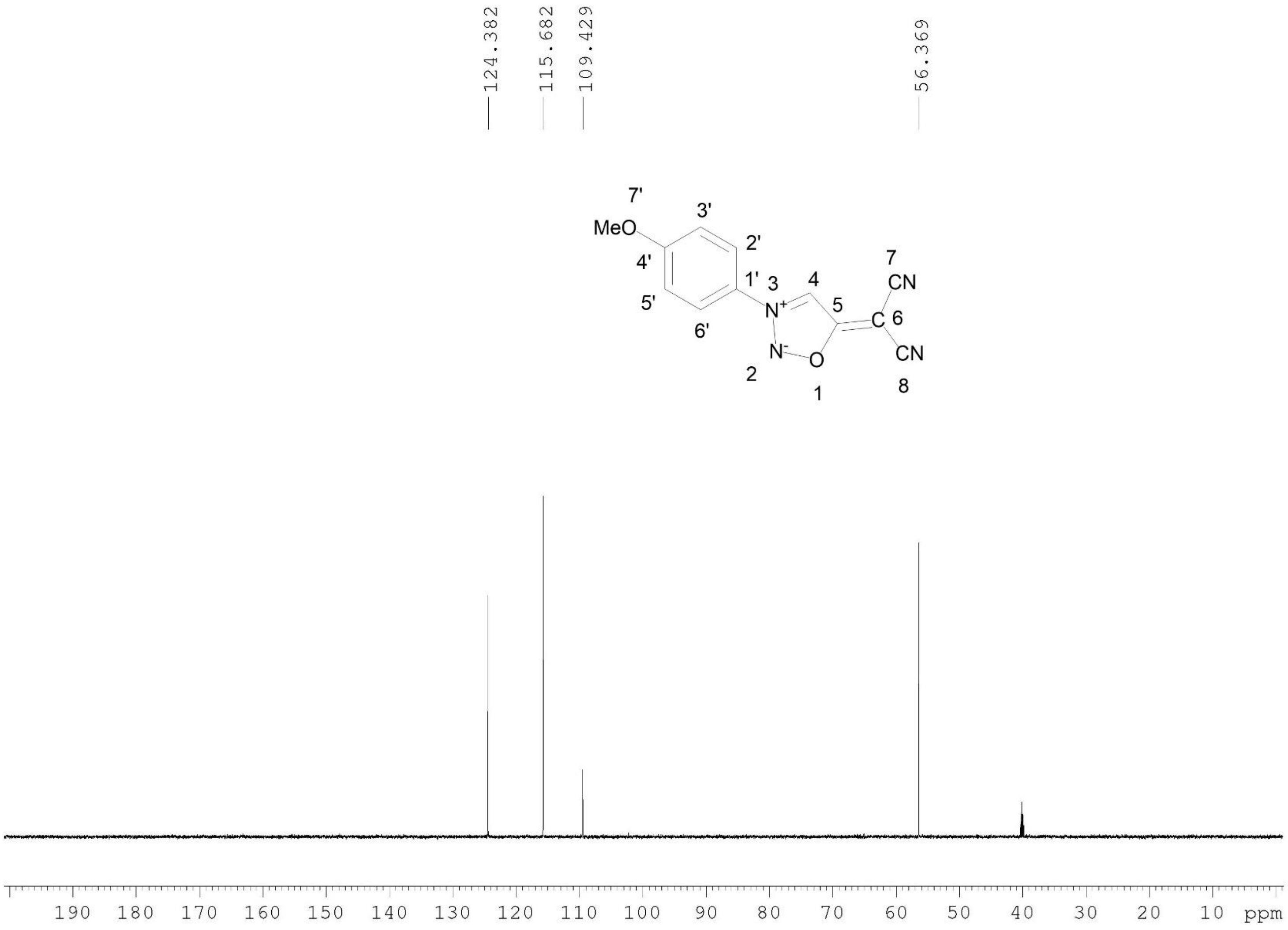

2d ^{1}H-NMR (600 MHz)

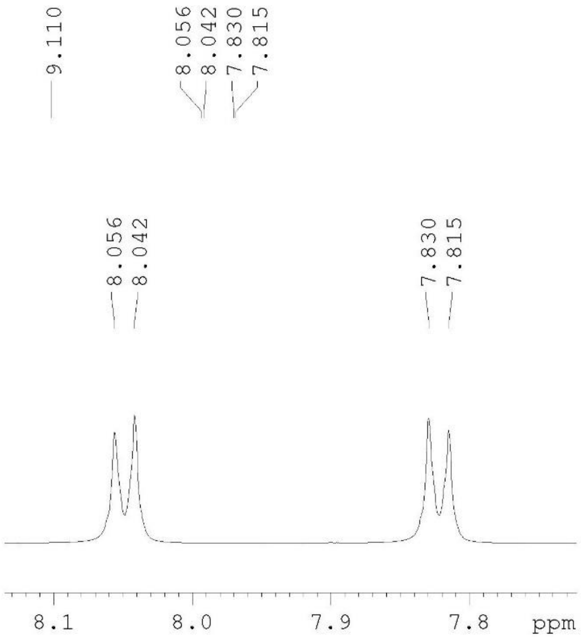

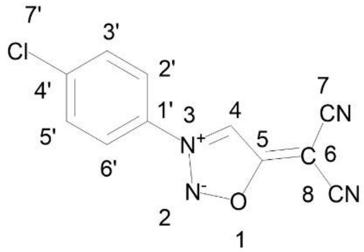

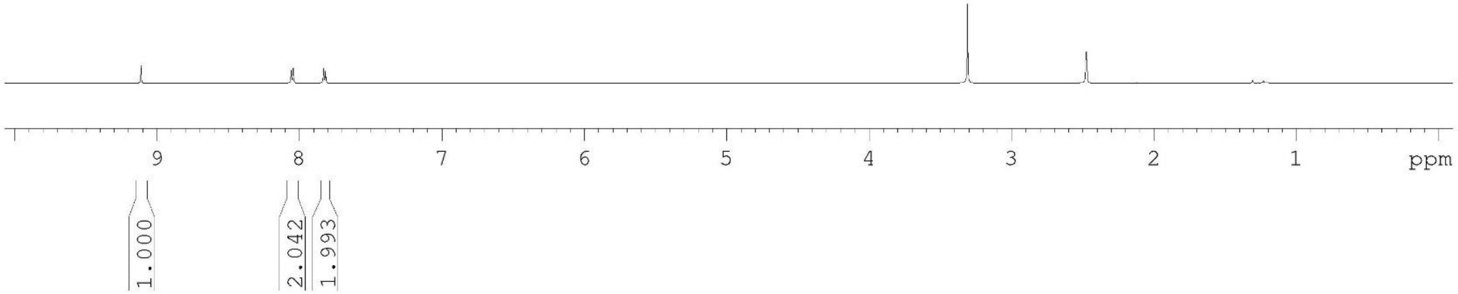

2d ^{13}C-NMR (150 MHz)

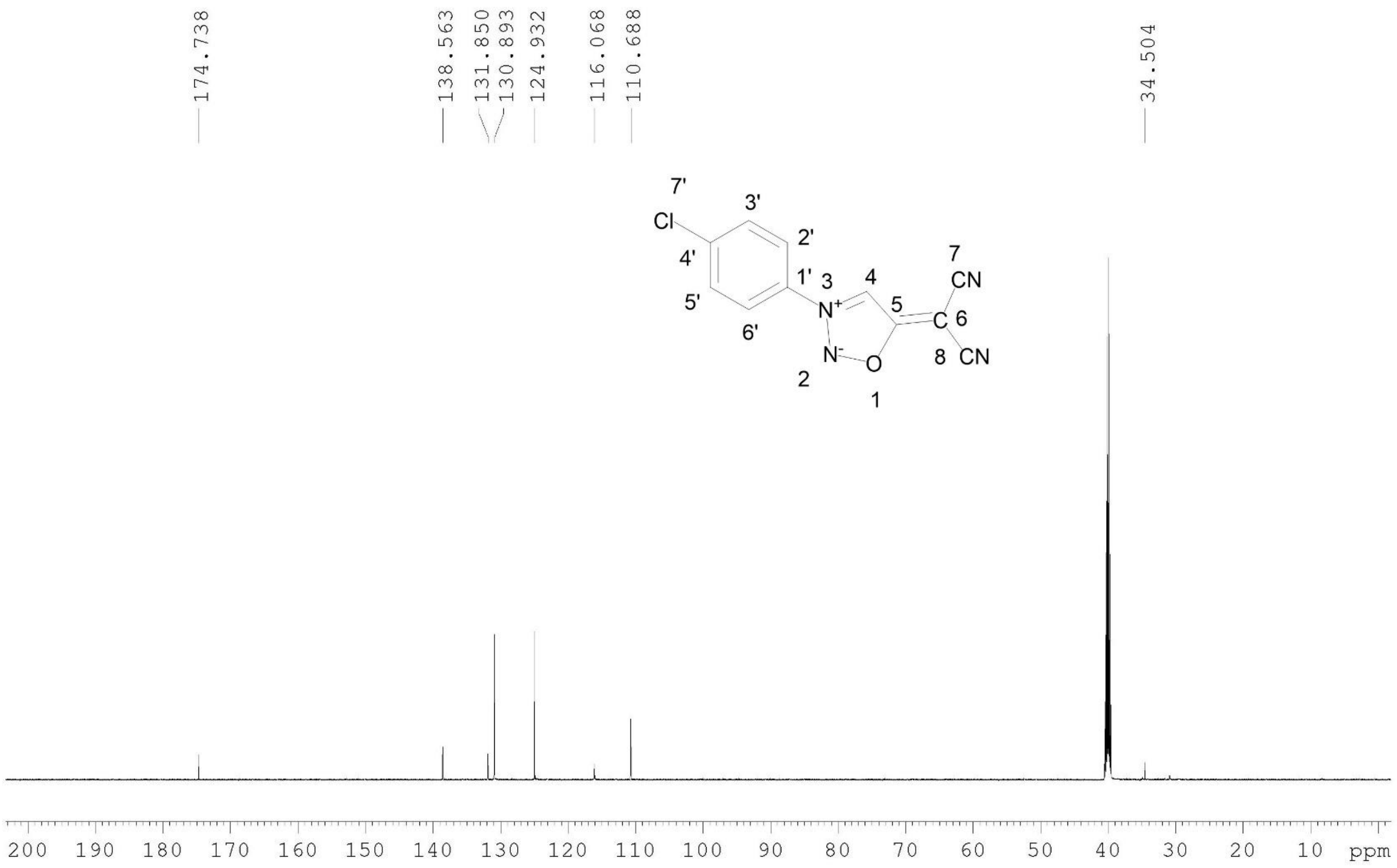

2d ^{13}C-NMR DEPT (150 MHz)

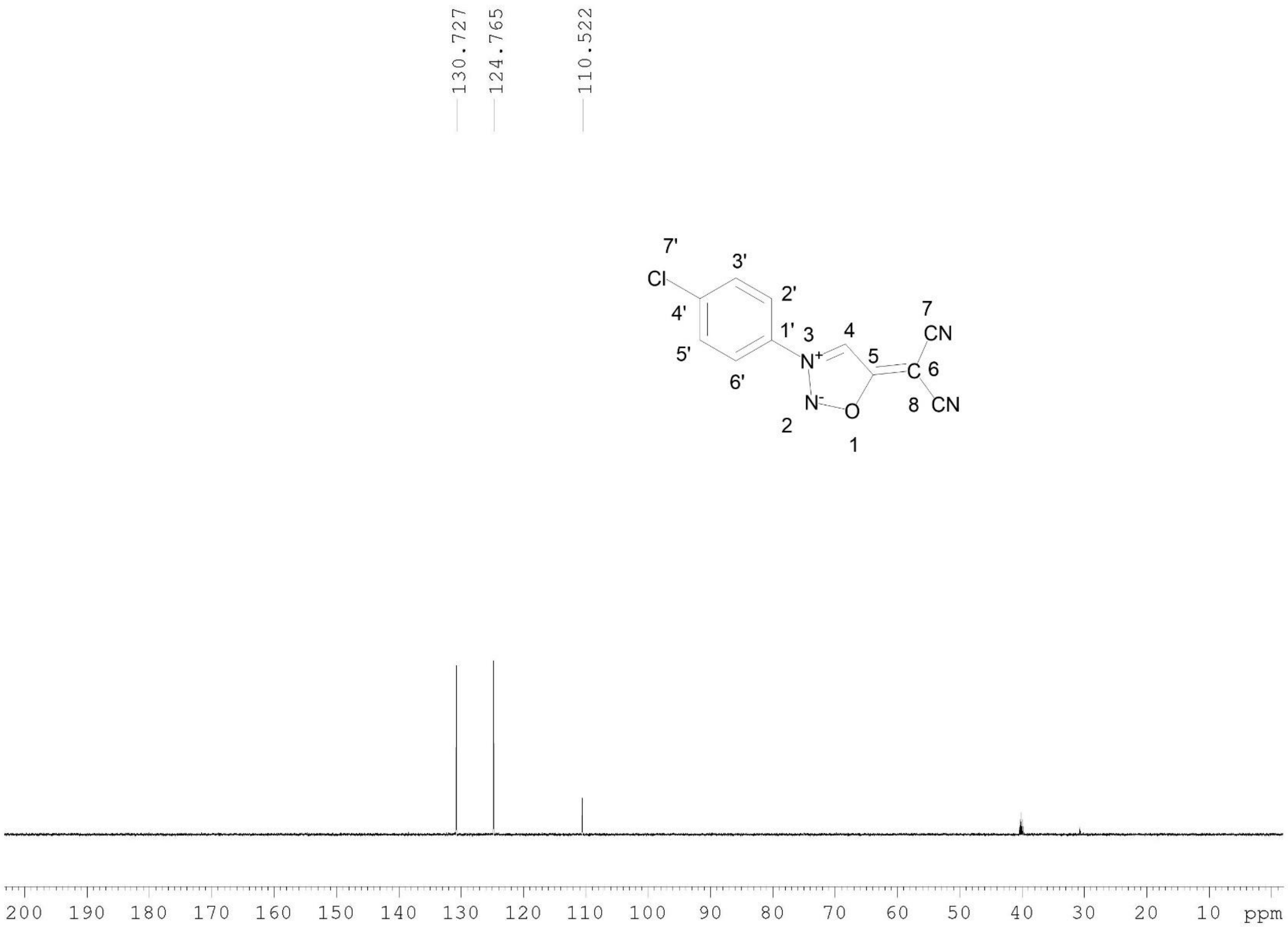

2e ^{1}H-NMR (600 MHz)

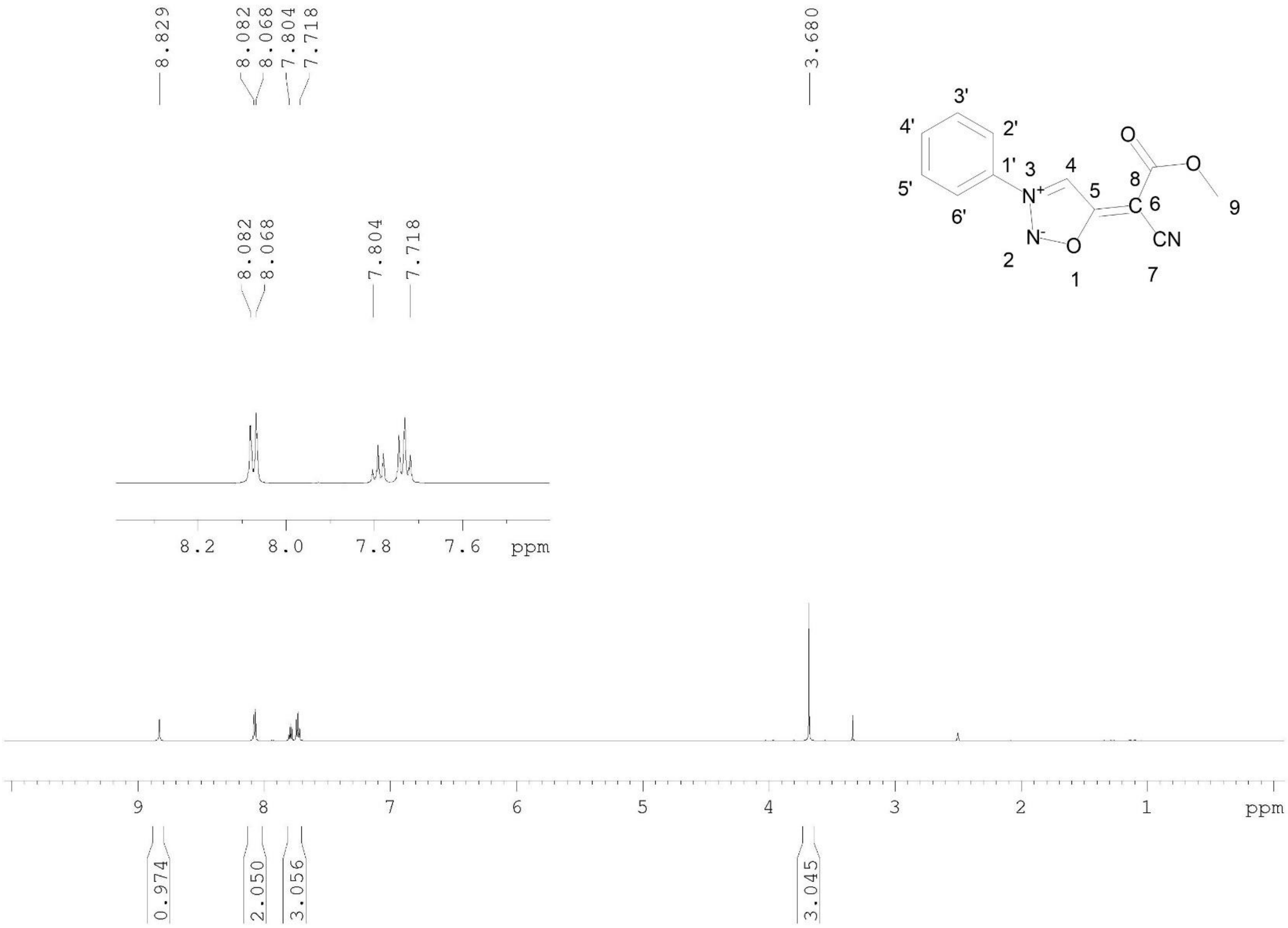

2e ^{13}C-NMR (150 MHz)

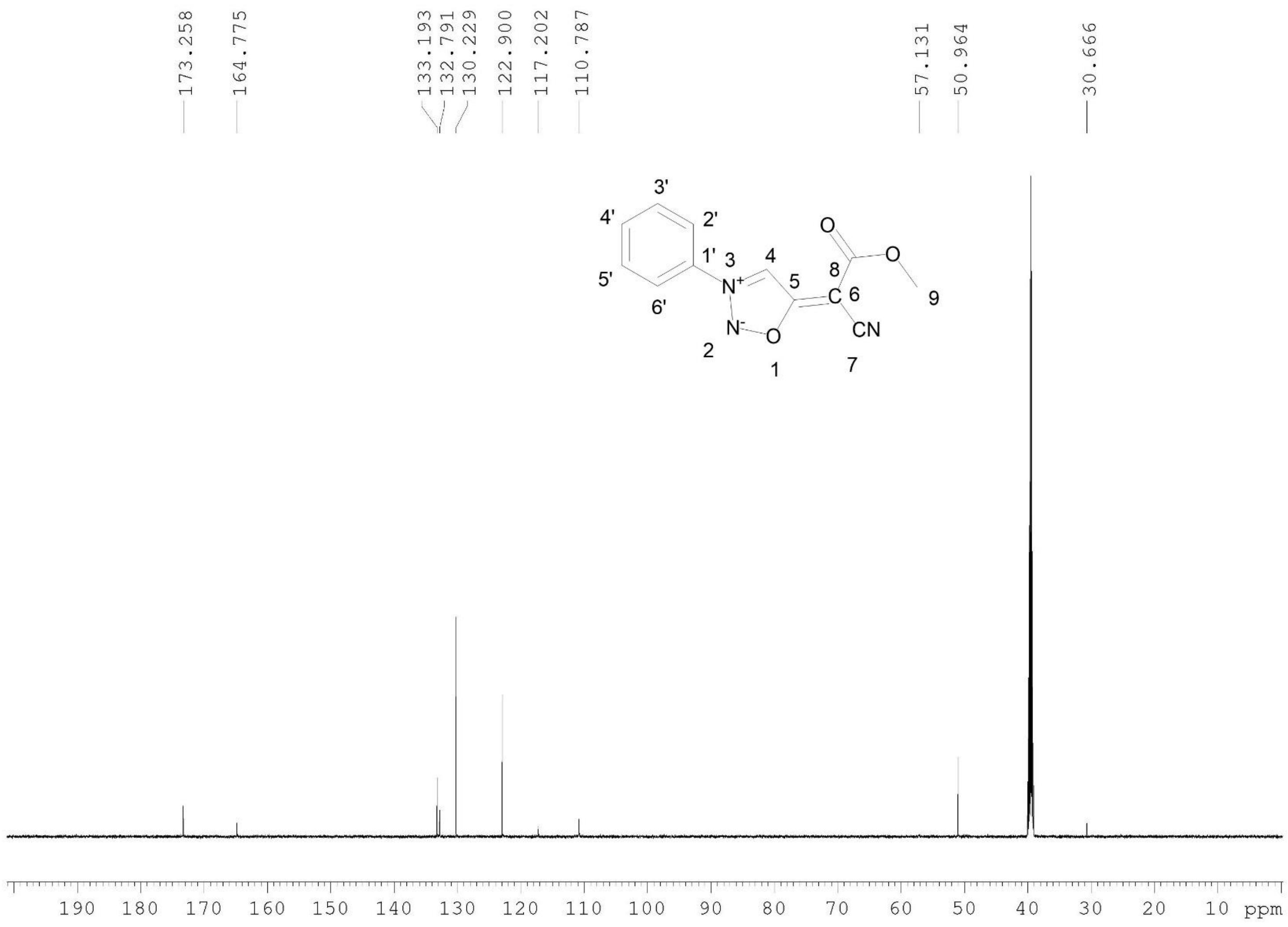

2e ^{15}N-NMR (zgig30, 61 MHz)

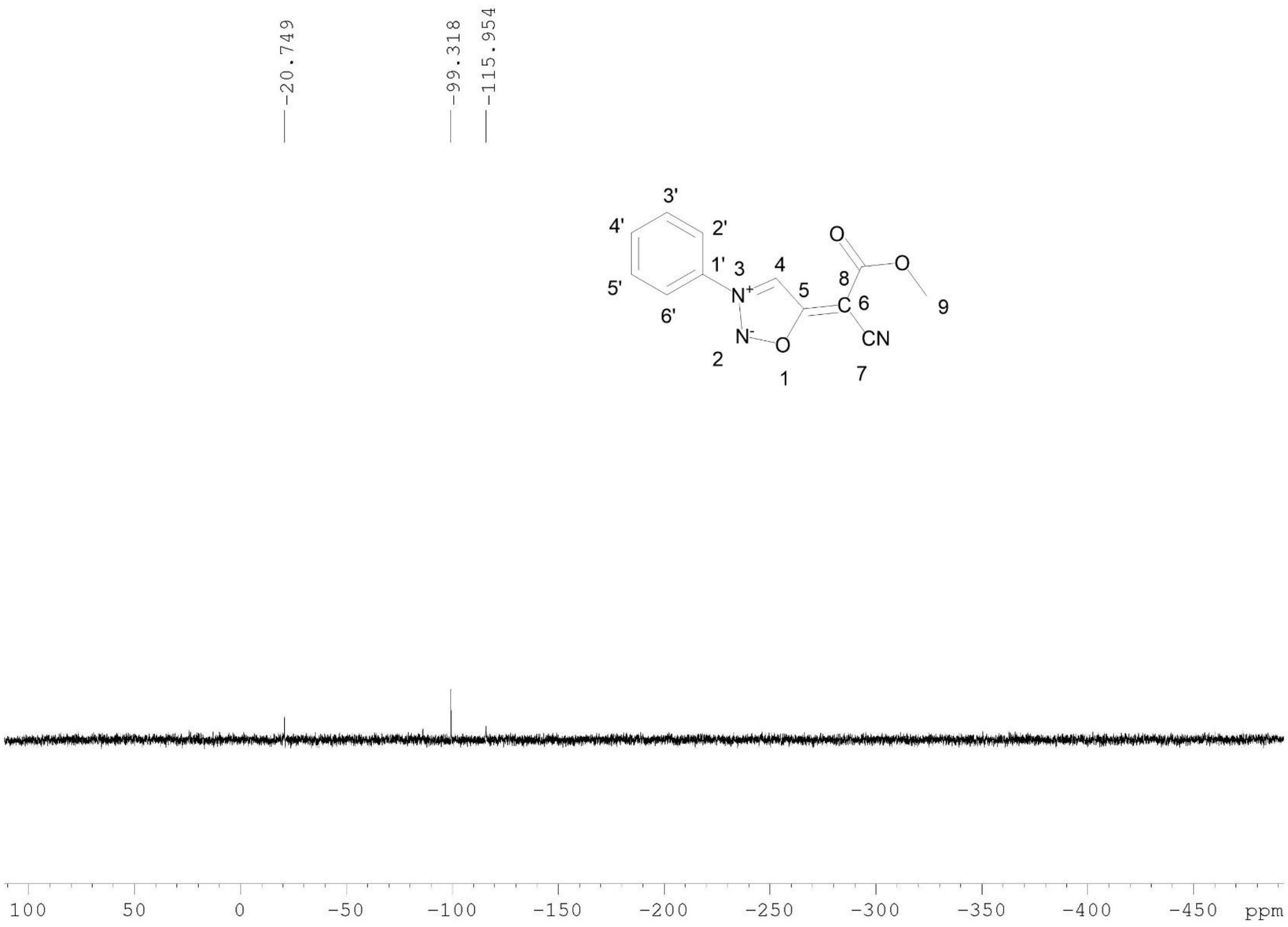

2e ^{13}C-NMR DEPT (150 MHz)

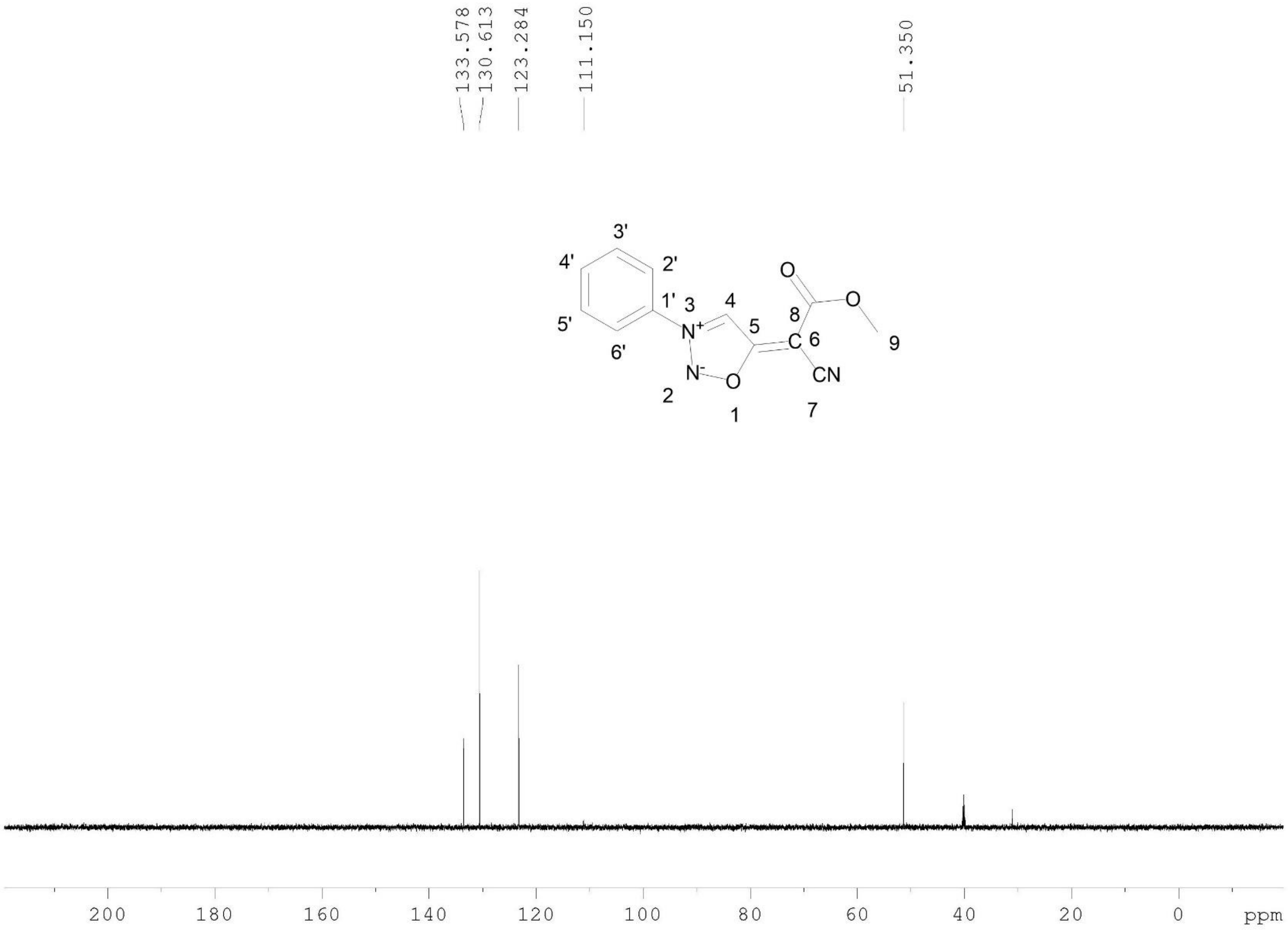

2f ^{1}H-NMR (600 MHz)

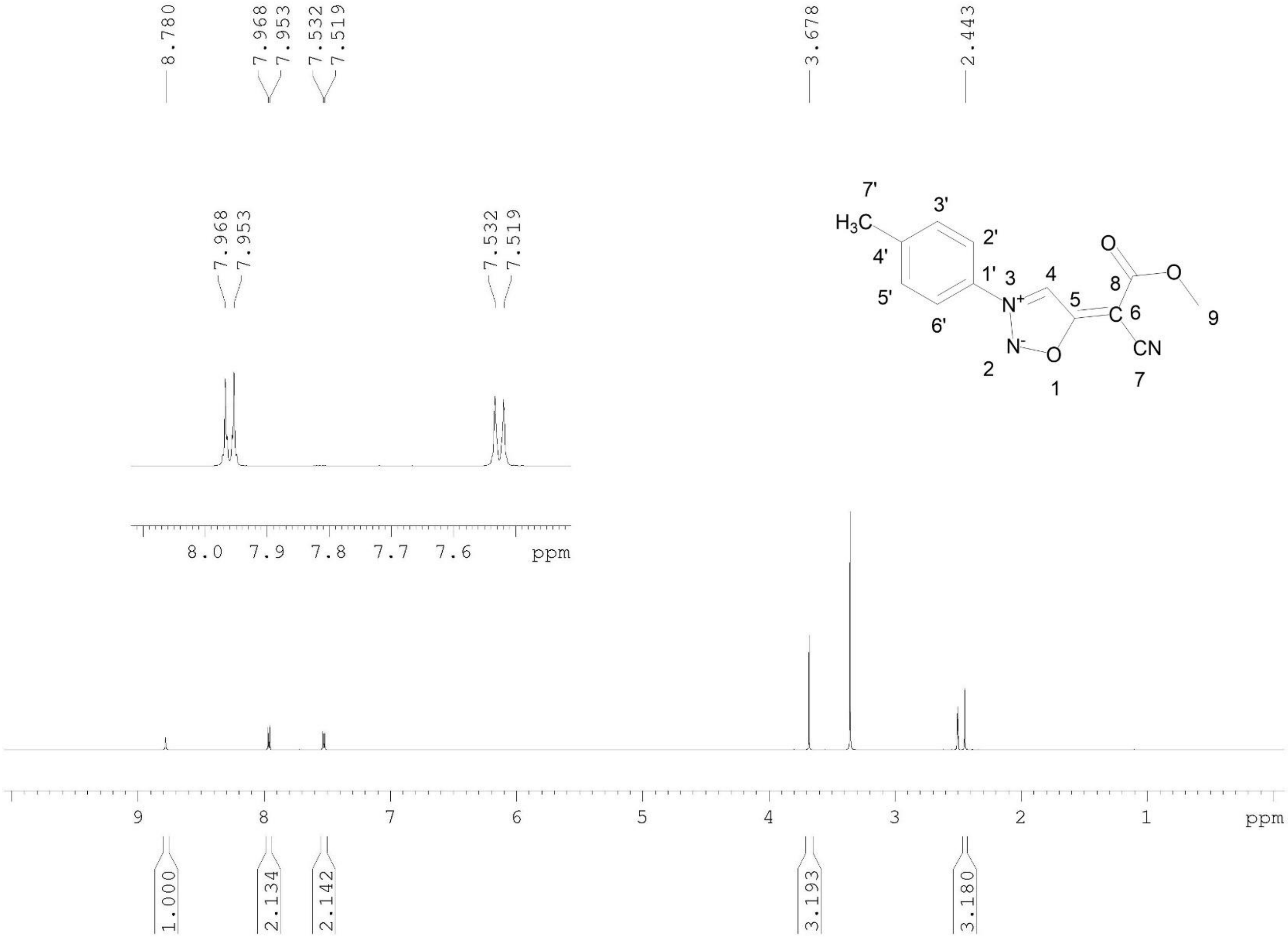

2f ^{13}C-NMR (150 MHz)

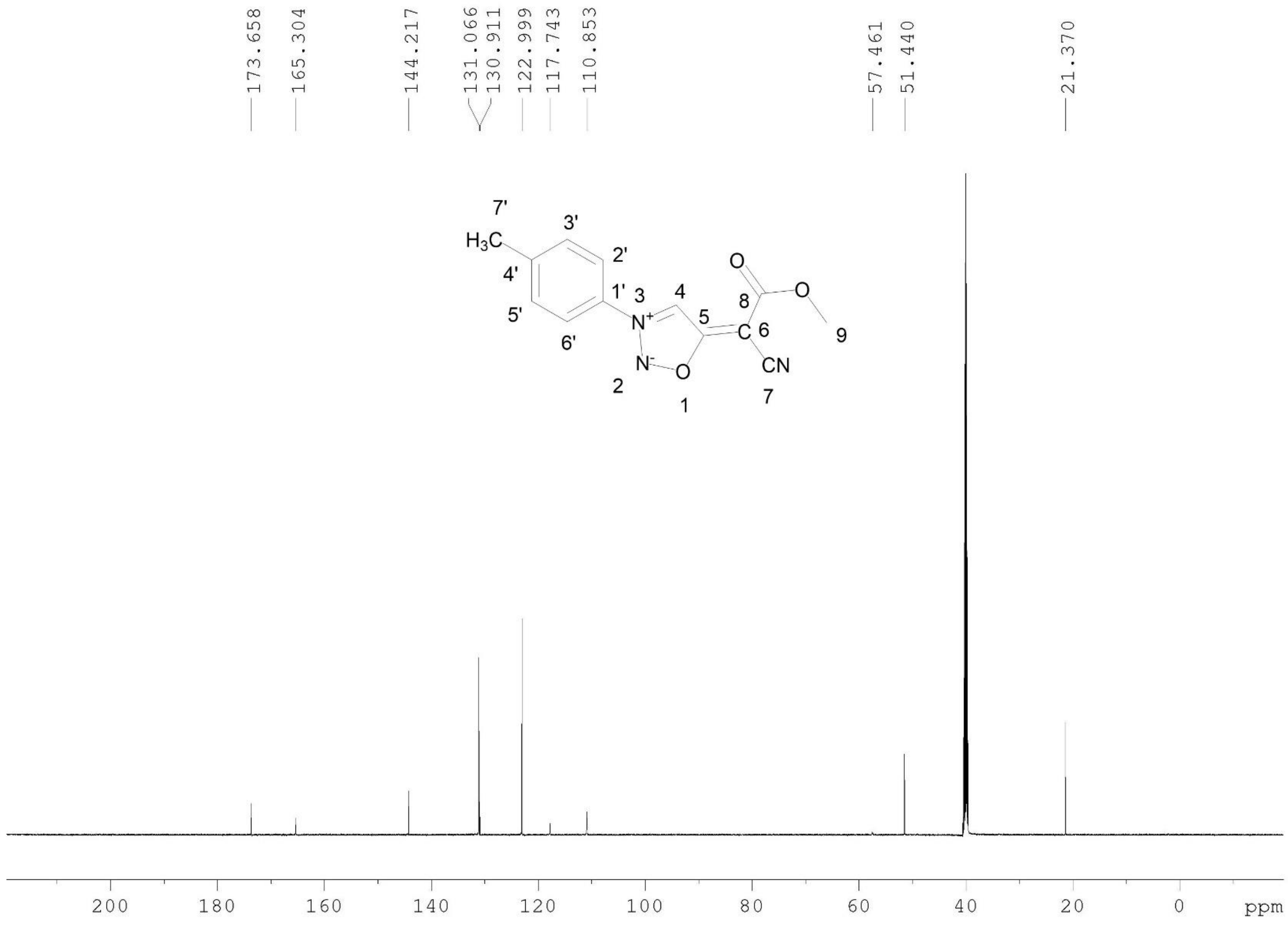

2f ^{13}C-NMR DEPT (150 MHz)

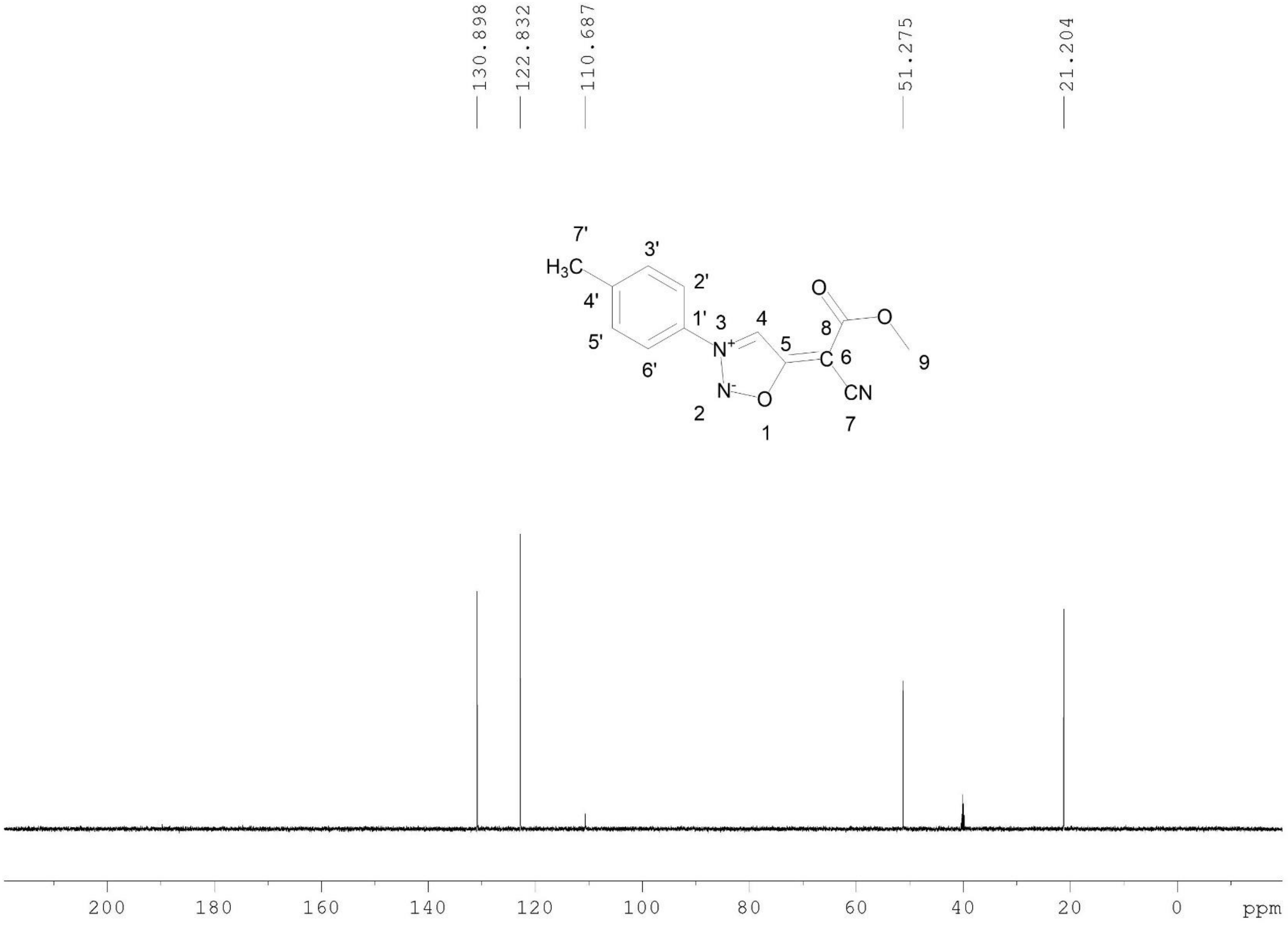

2g ^{1}H-NMR (600 MHz)

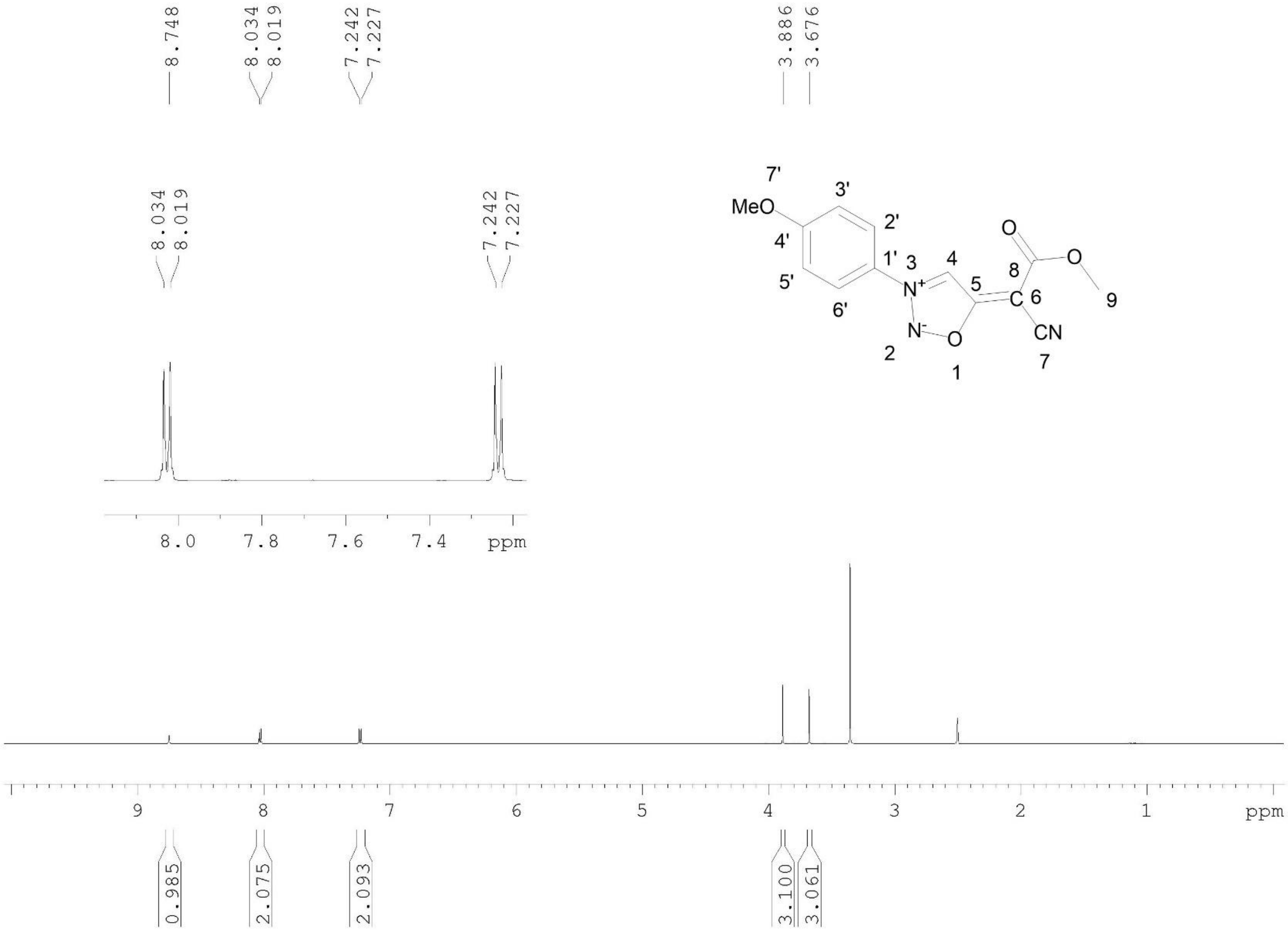

2g ^{13}C-NMR (150 MHz)

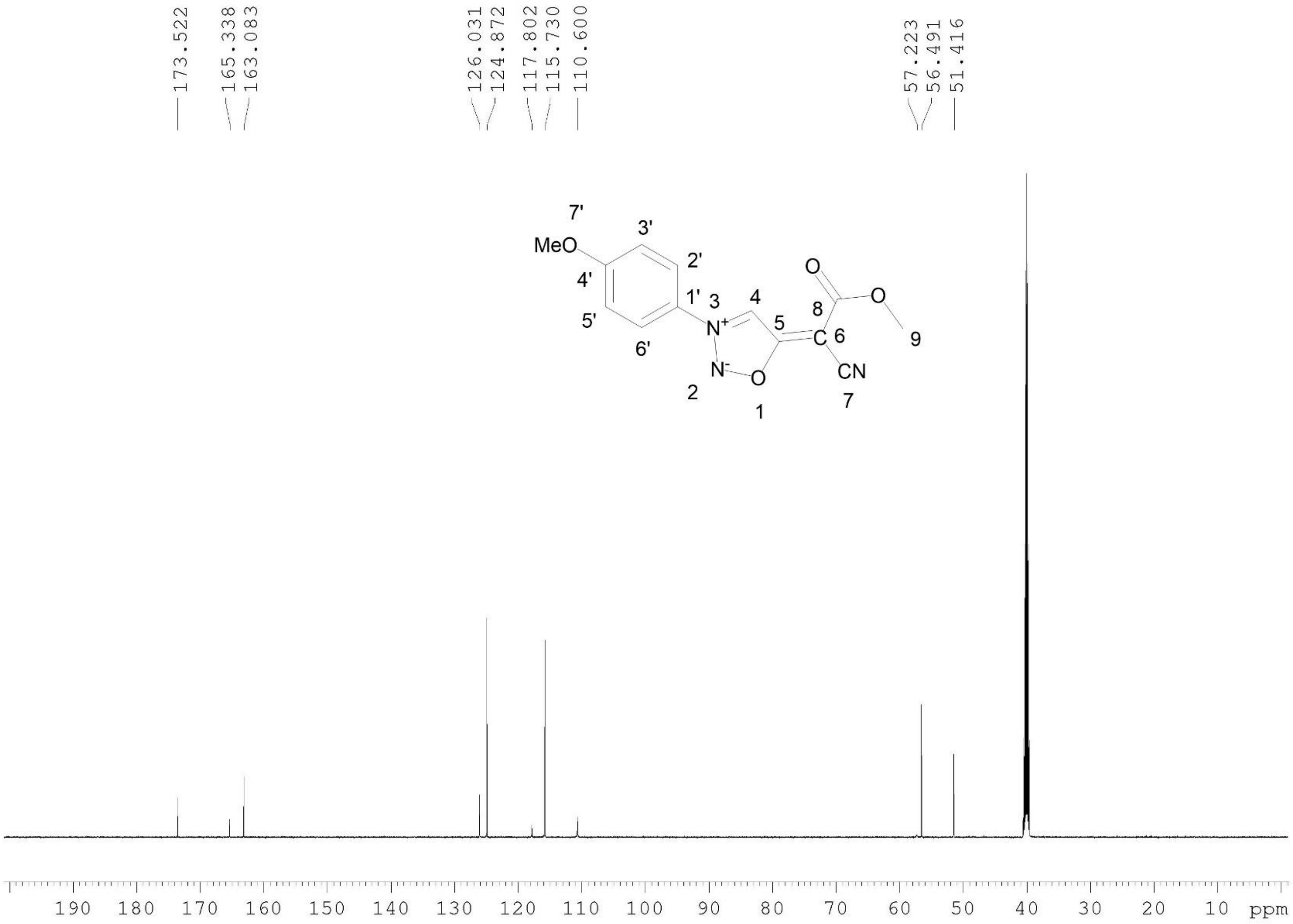

2g ^{13}C-NMR DEPT (150 MHz)

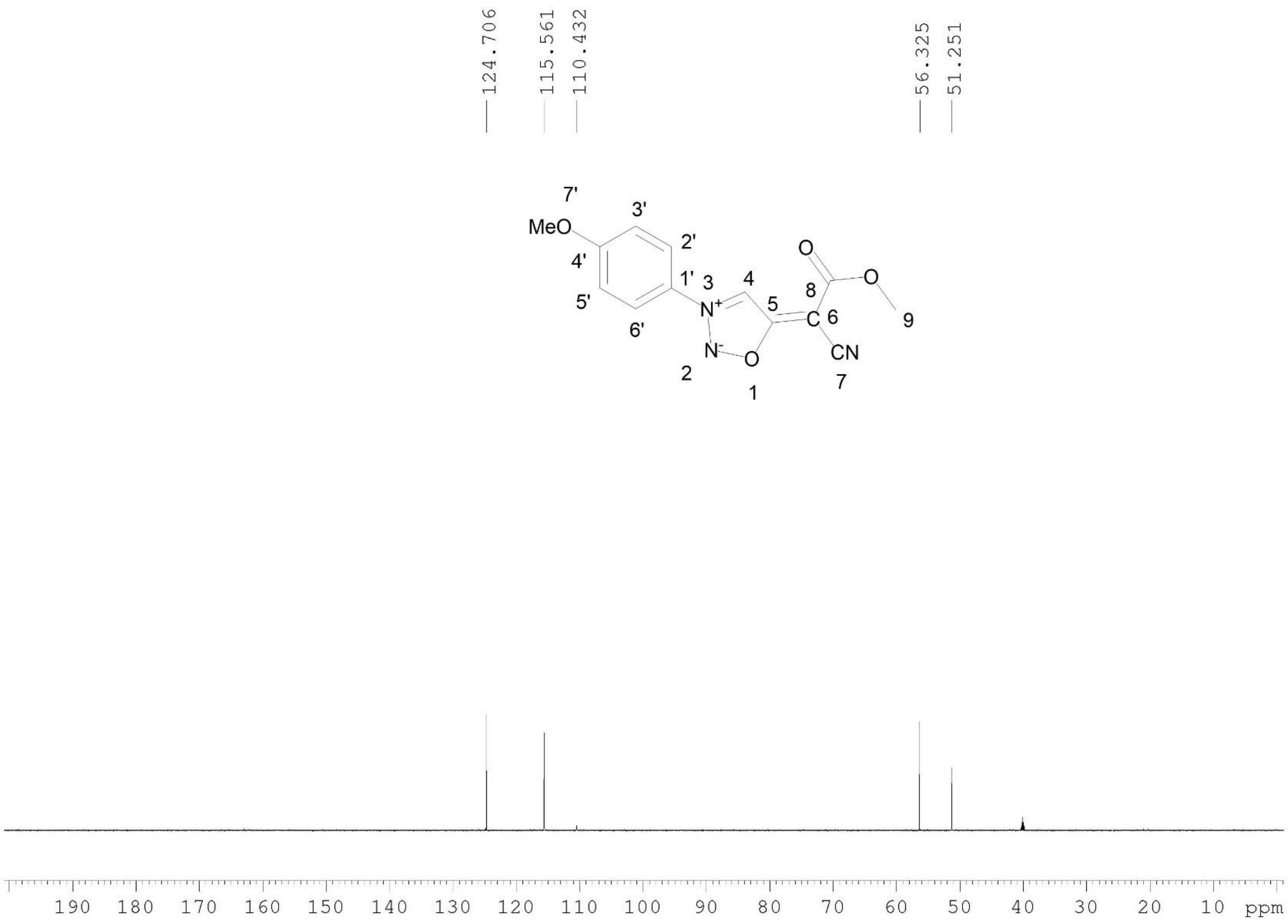

2h ^{1}H-NMR (600 MHz)

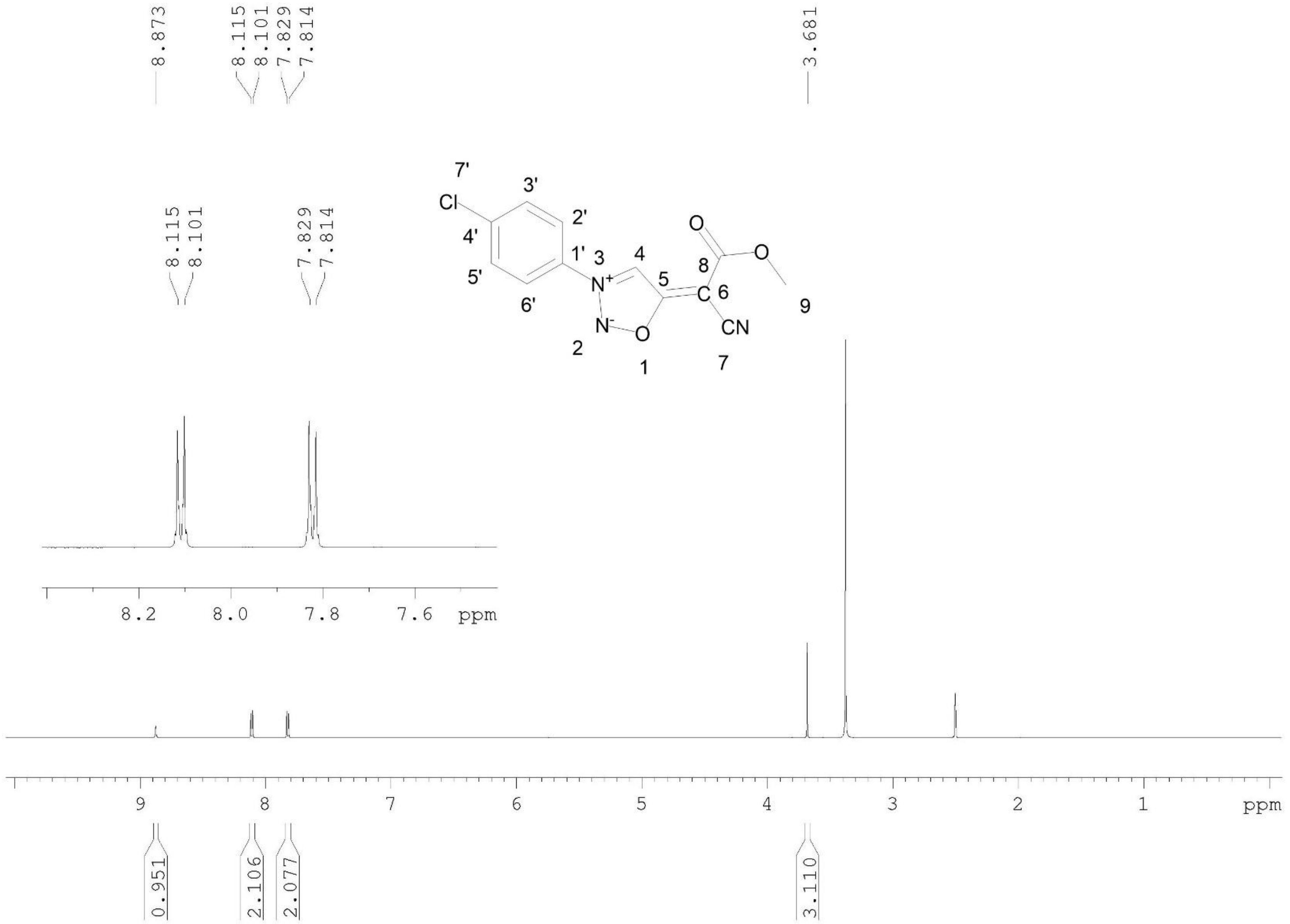

2h ^{13}C-NMR (150 MHz)

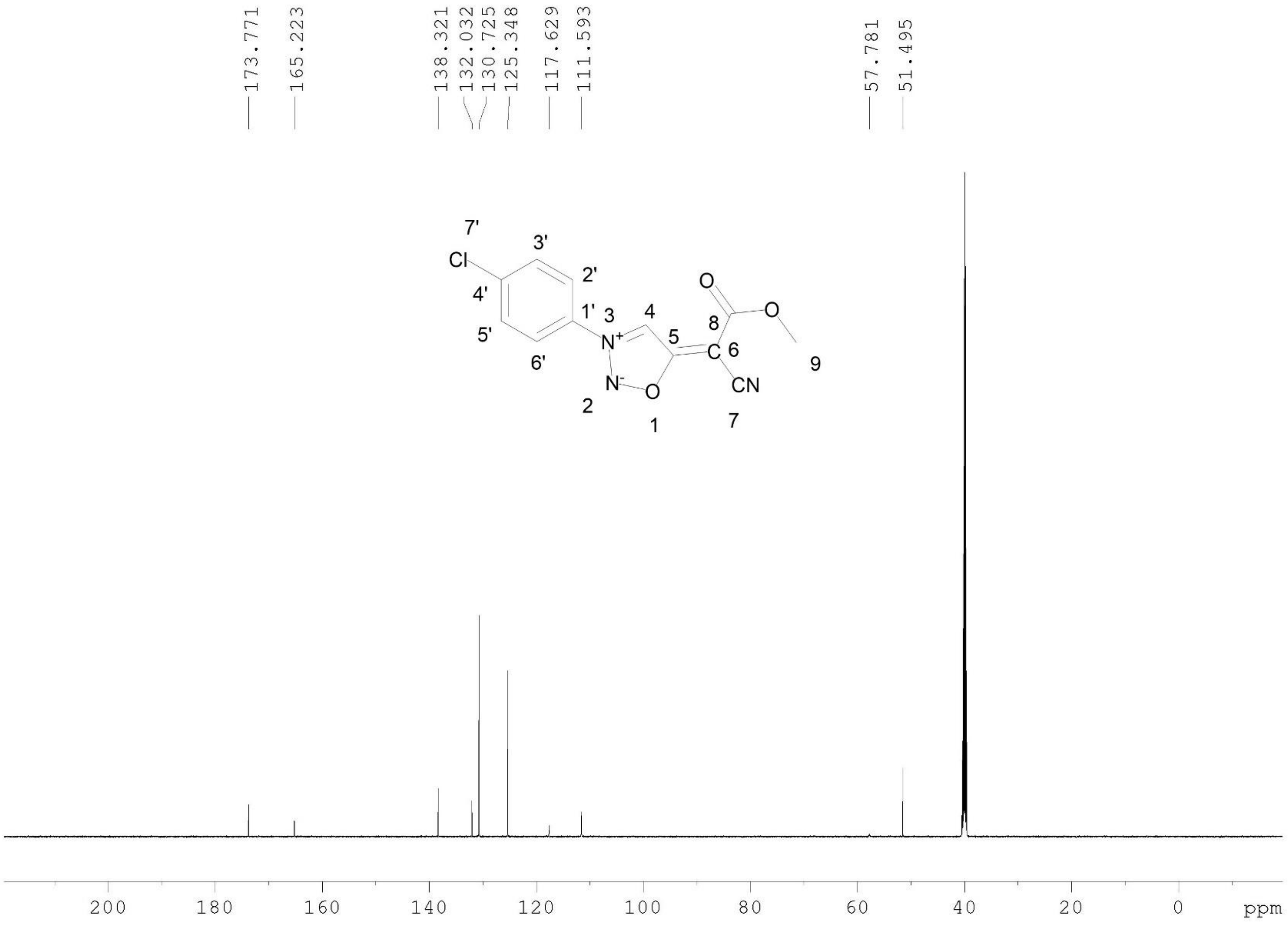

2h ^{13}C-NMR DEPT (150 MHz)

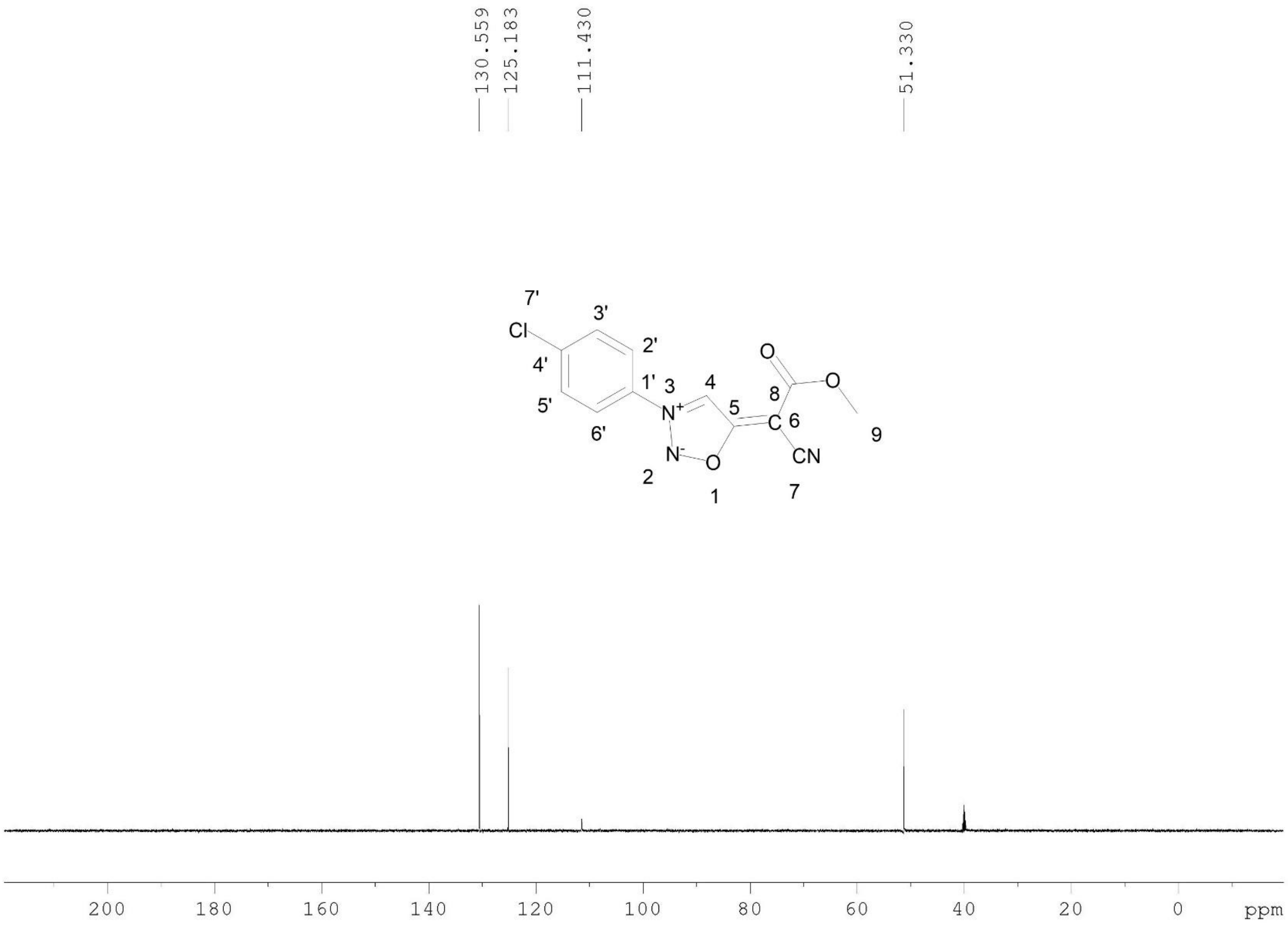

2i ^{1}H-NMR (600 MHz)

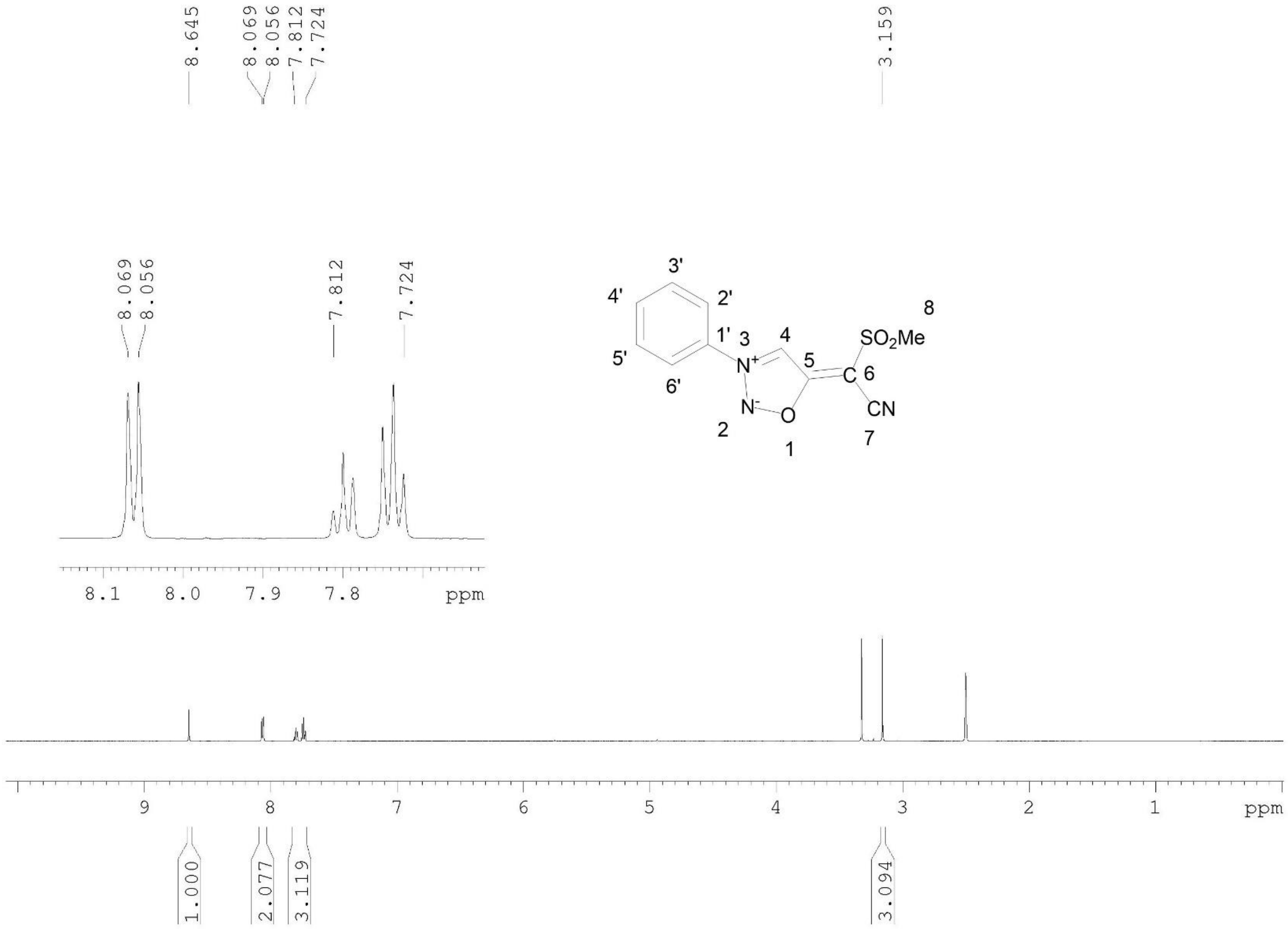

2i ^{13}C-NMR (150 MHz)

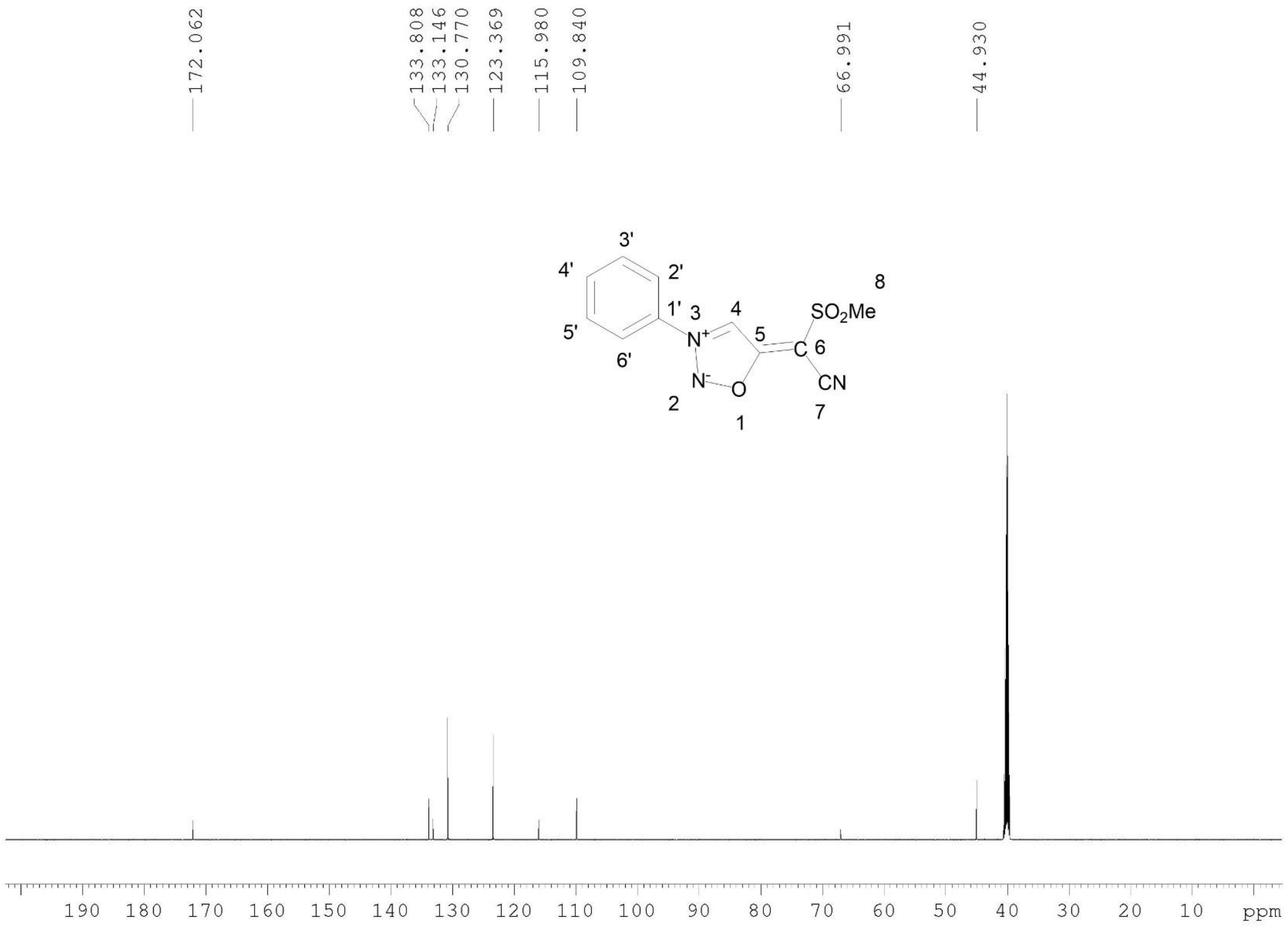

2i ^{15}N-NMR (zgig30, 61 MHz)

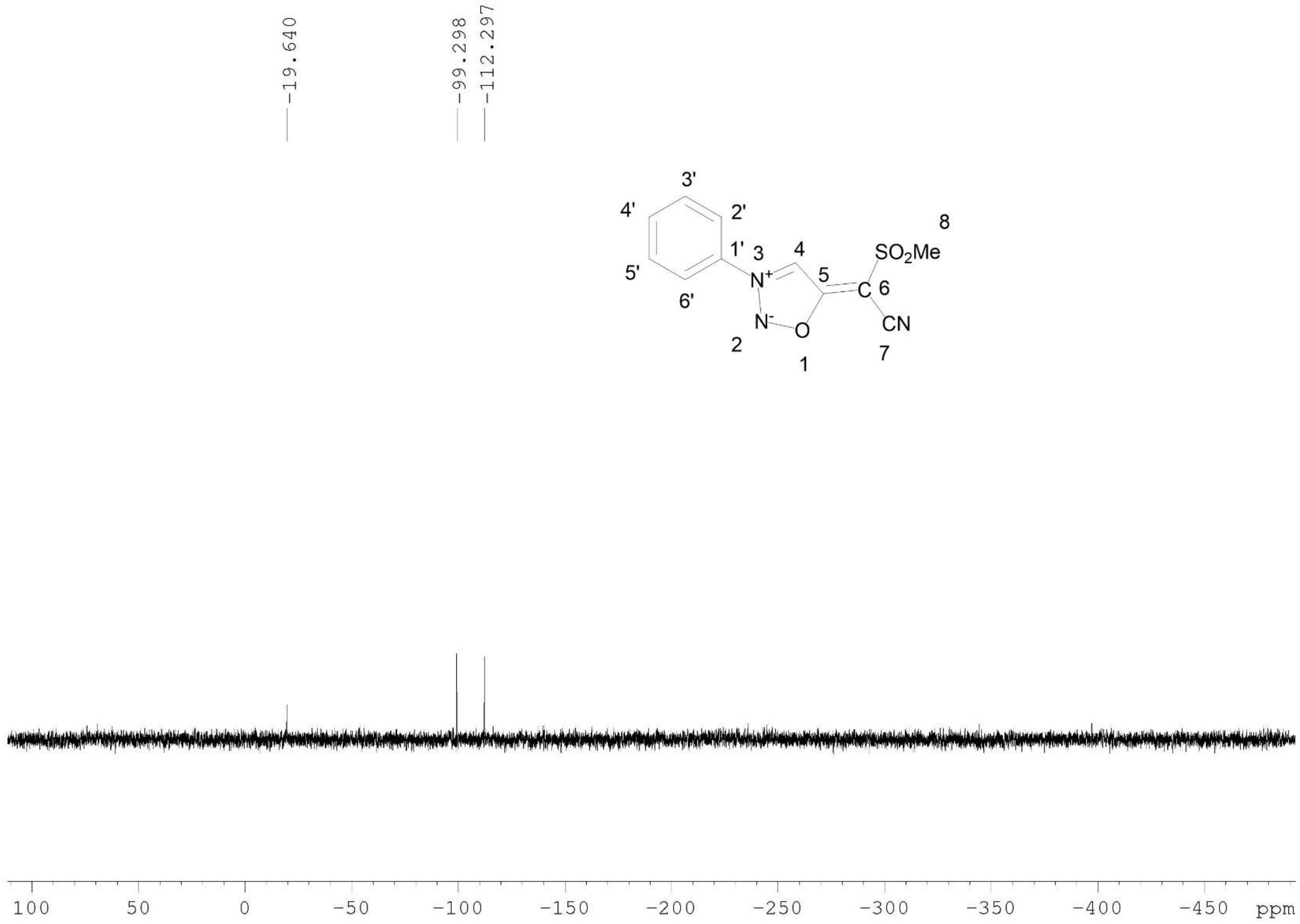

2i ^{13}C-NMR DEPT (150 MHz)

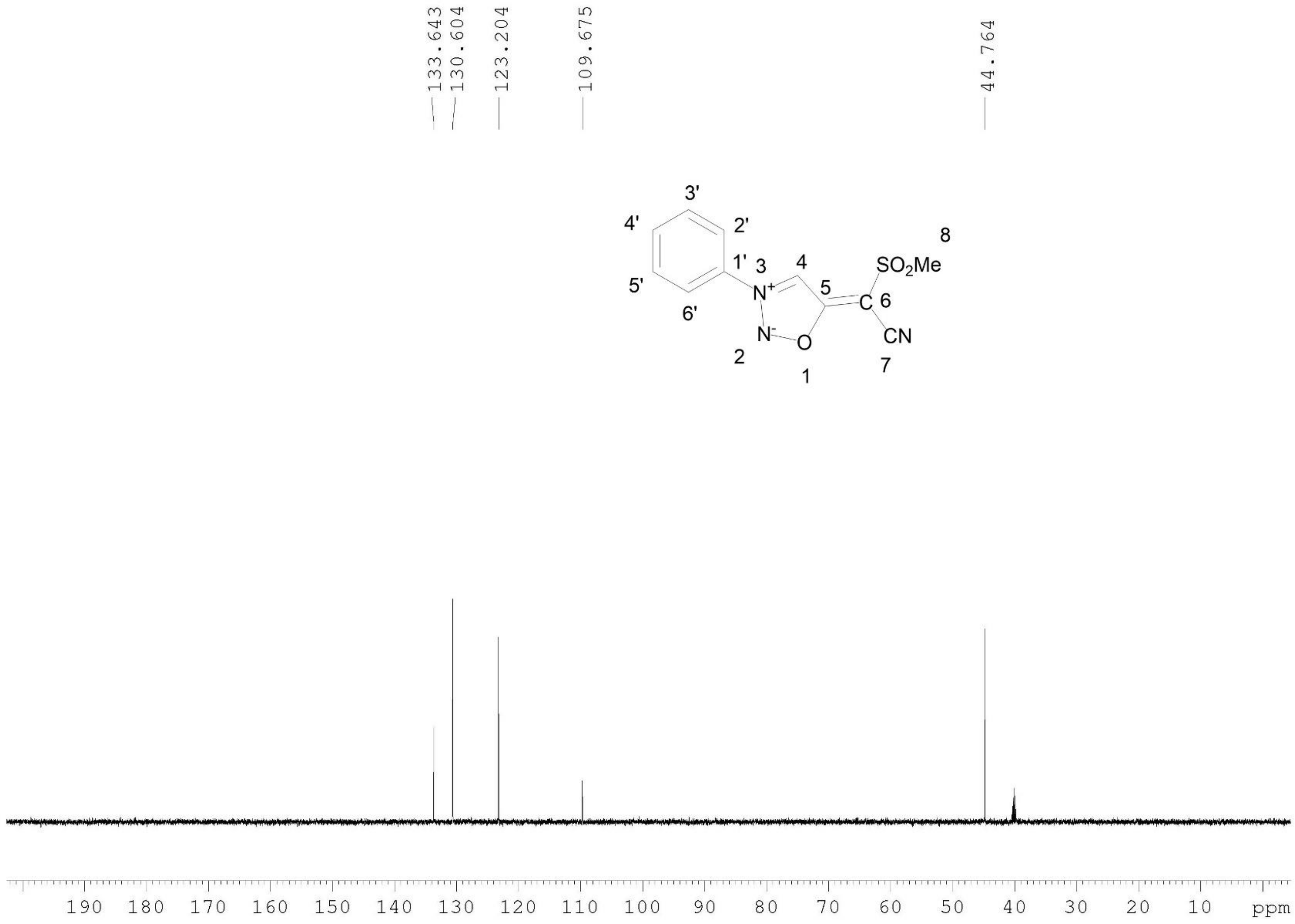

2j ^{1}H-NMR (600 MHz)

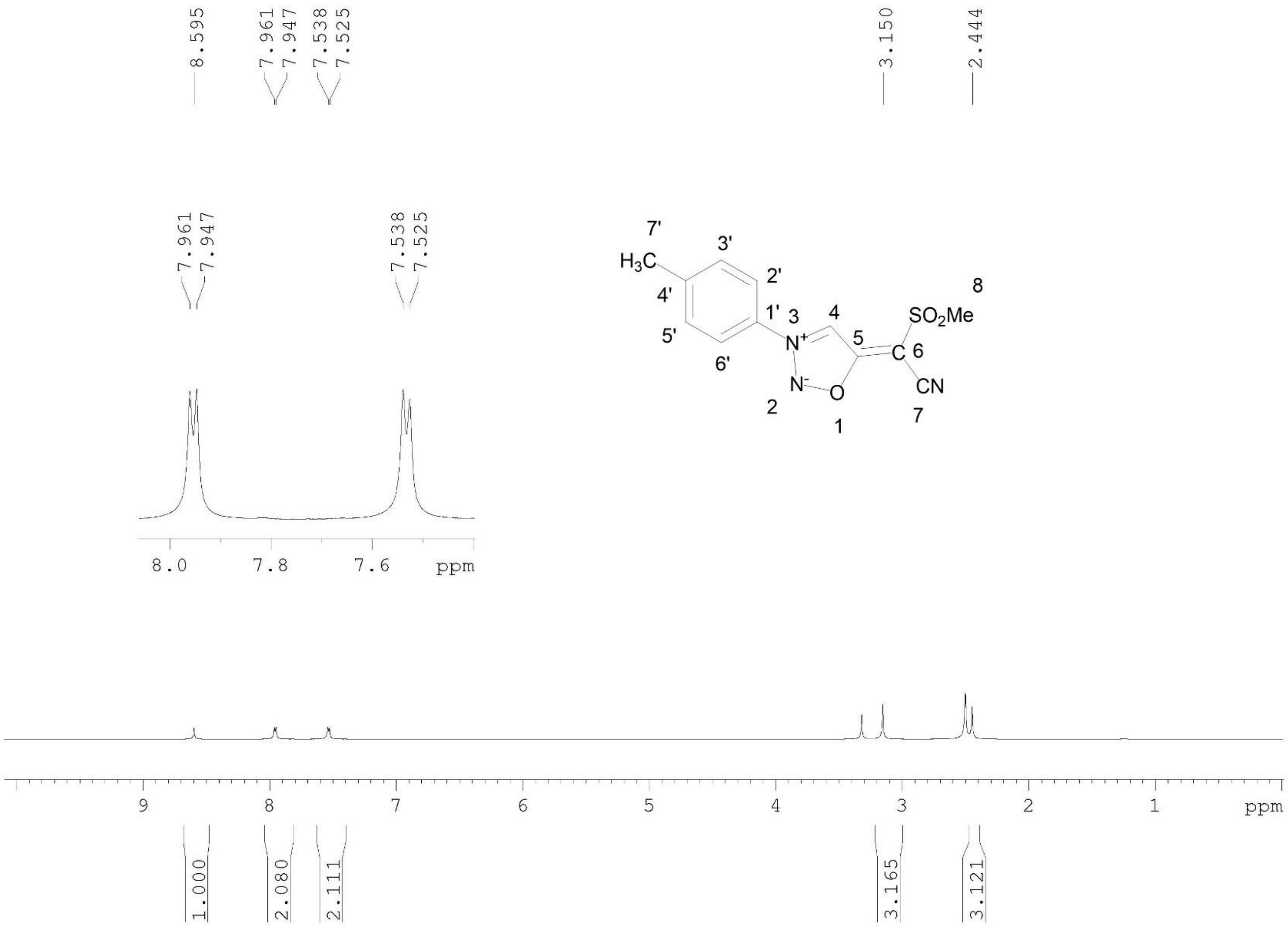

2j ^{13}C-NMR (150 MHz)

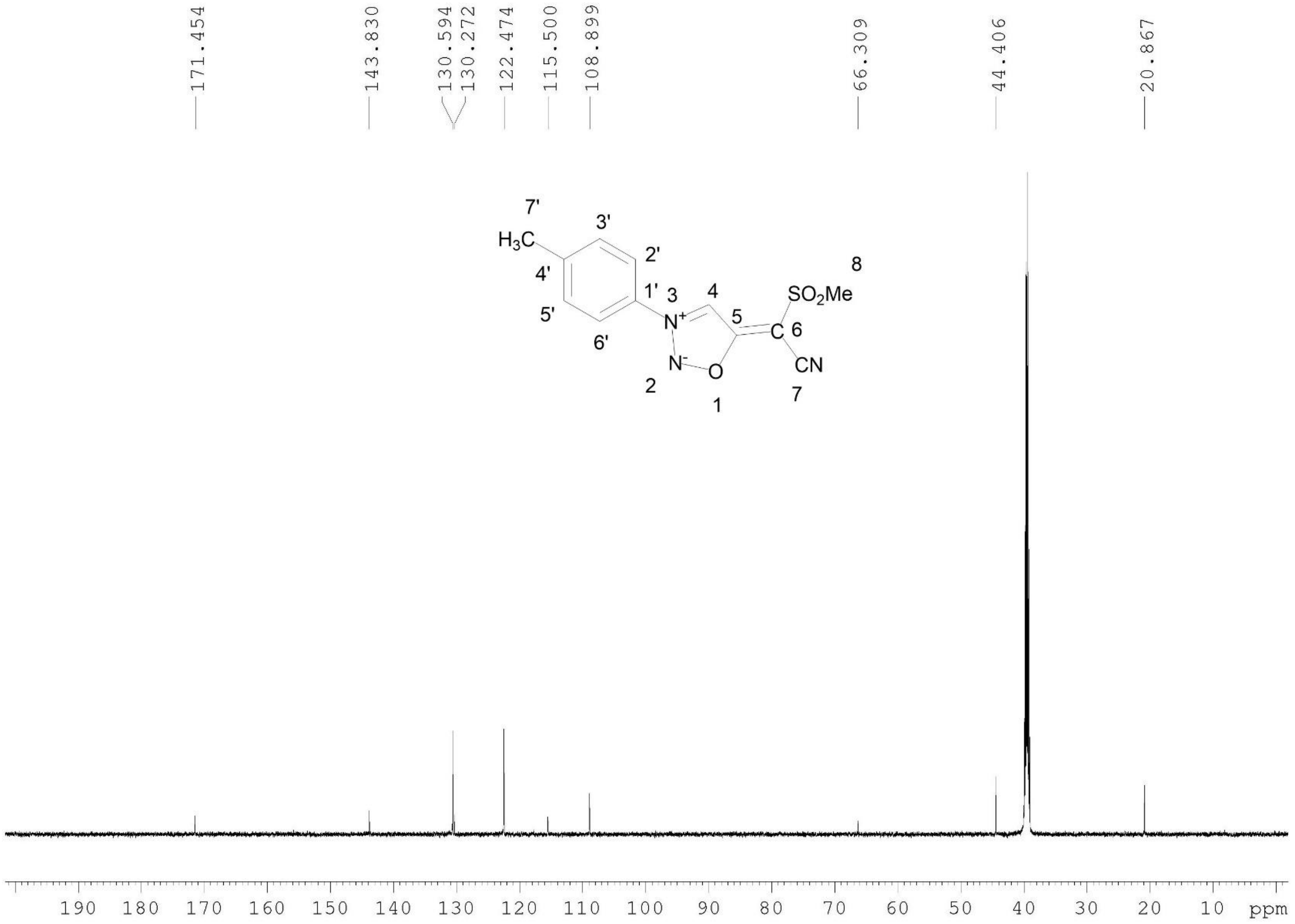

2j ^{13}C-NMR DEPT (150 MHz)

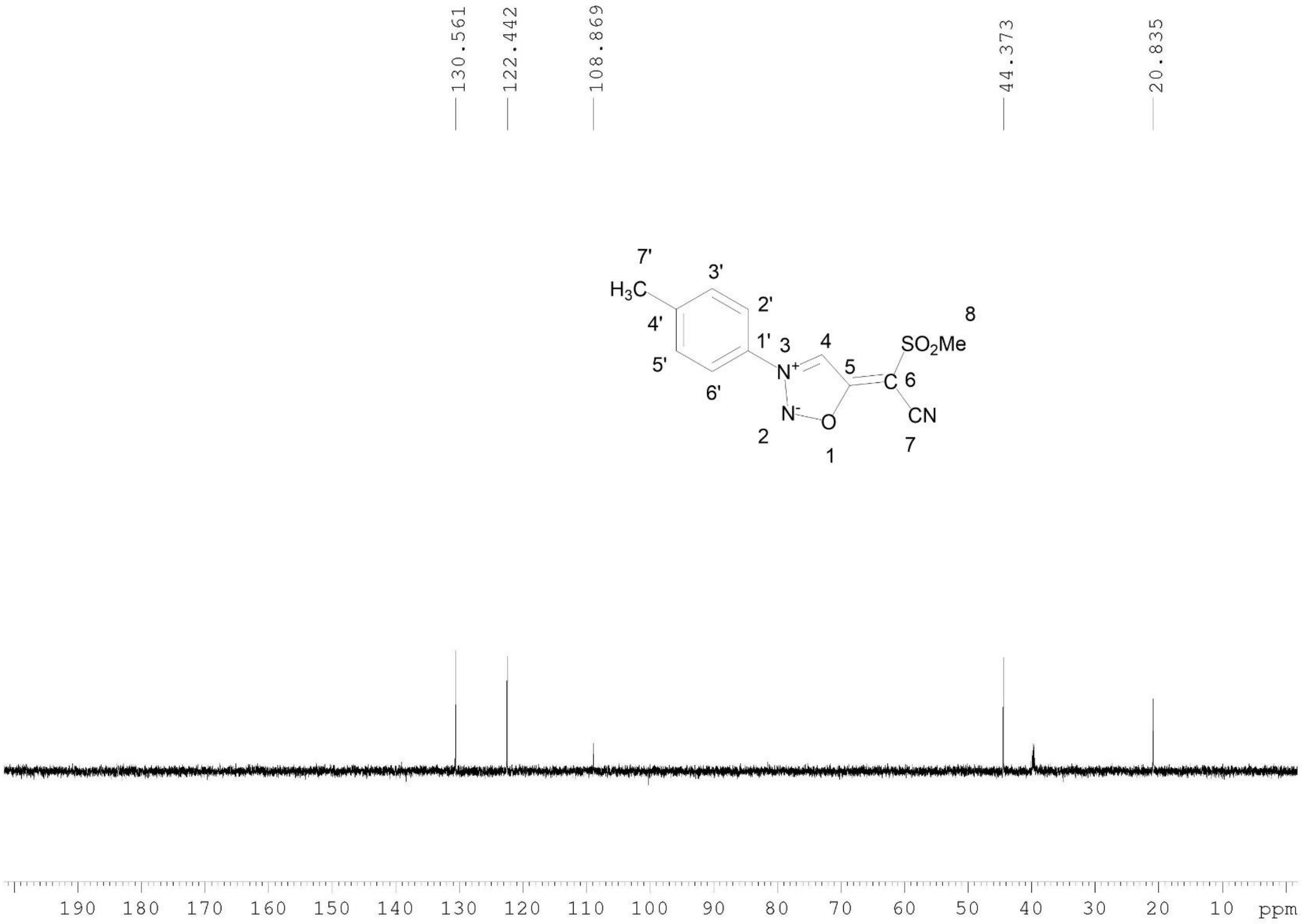

2k ^{1}H-NMR (600 MHz)

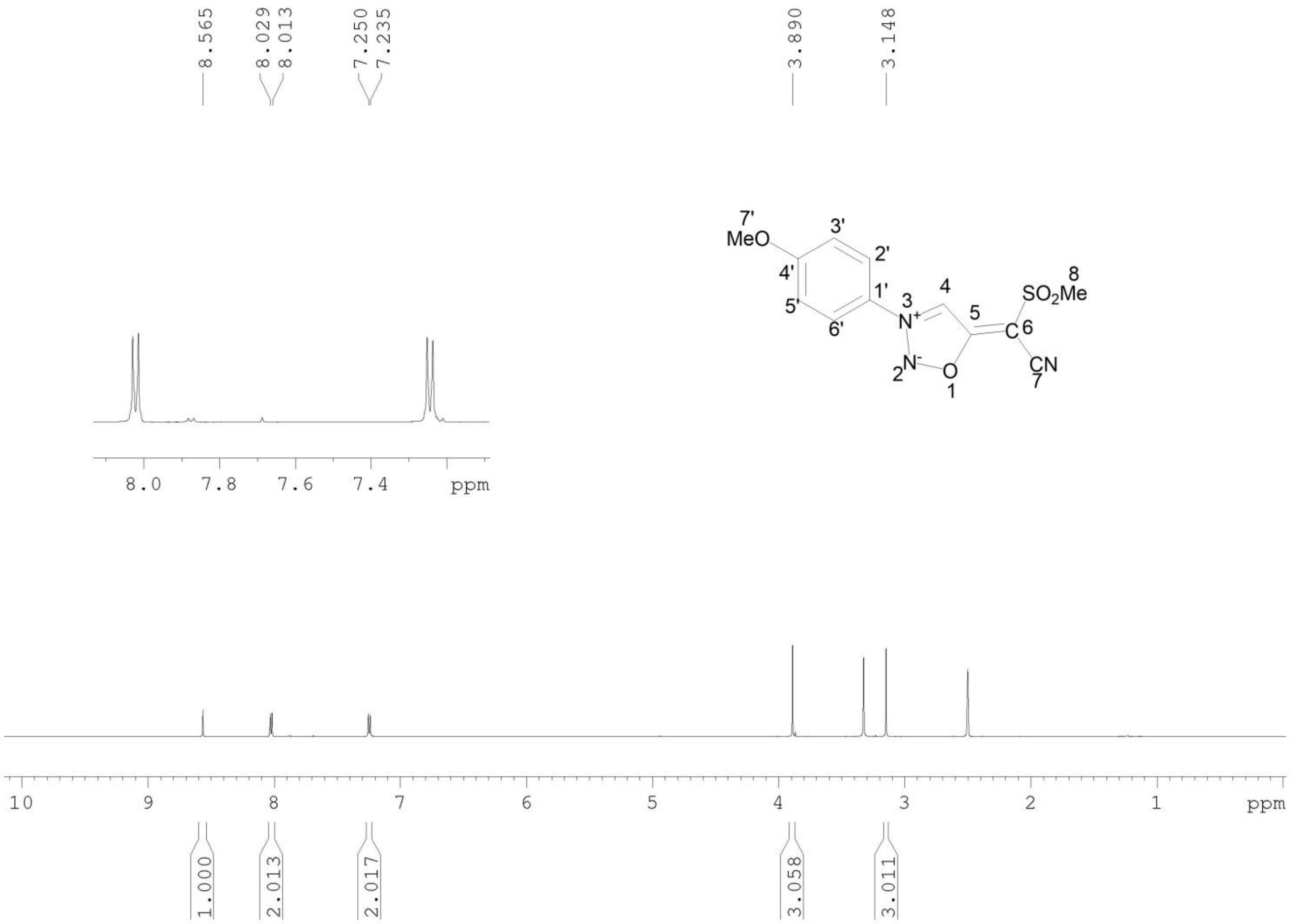

2k ^{13}C-NMR (150 MHz)

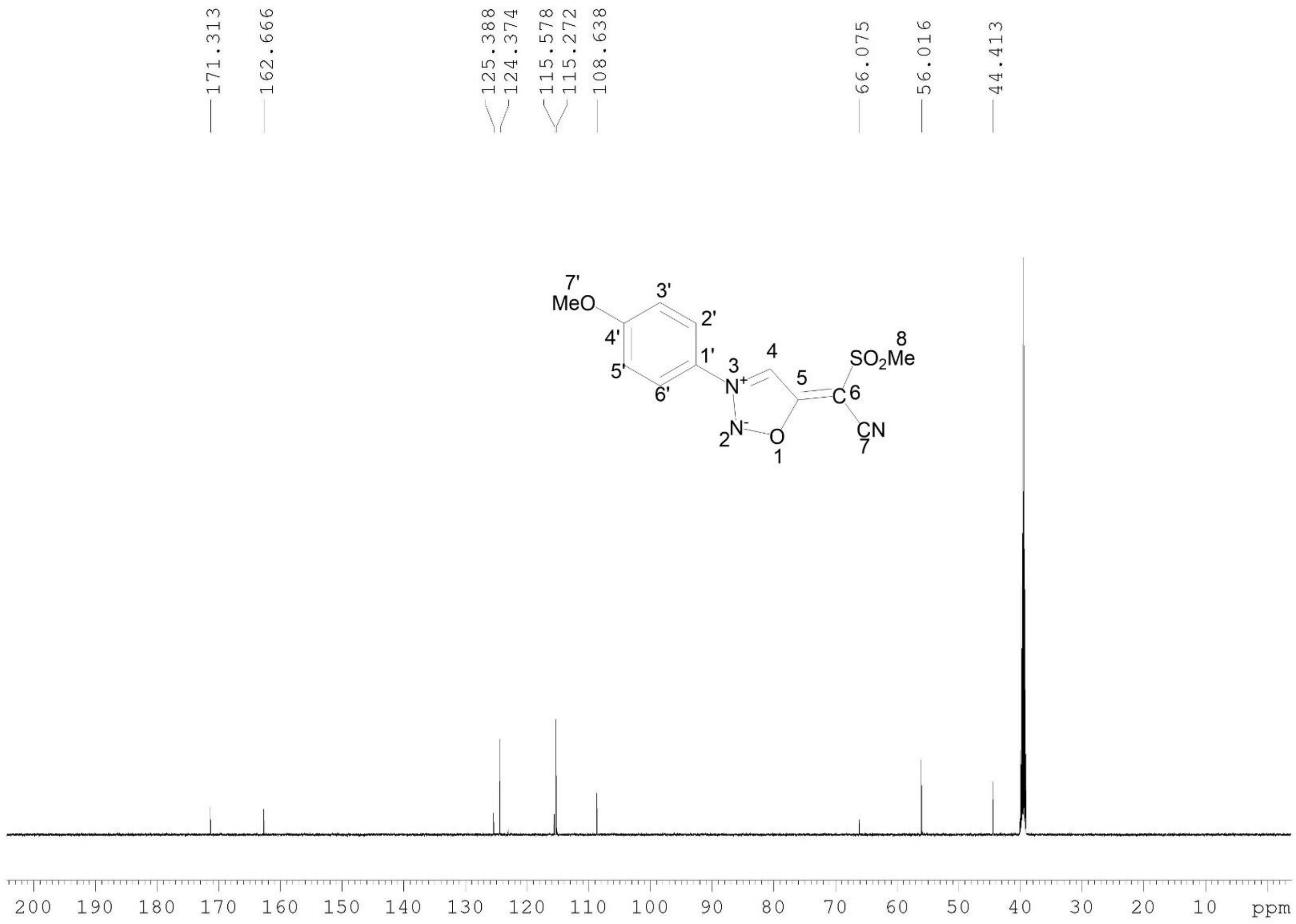

2k ^{13}C-NMR DEPT (150 MHz)

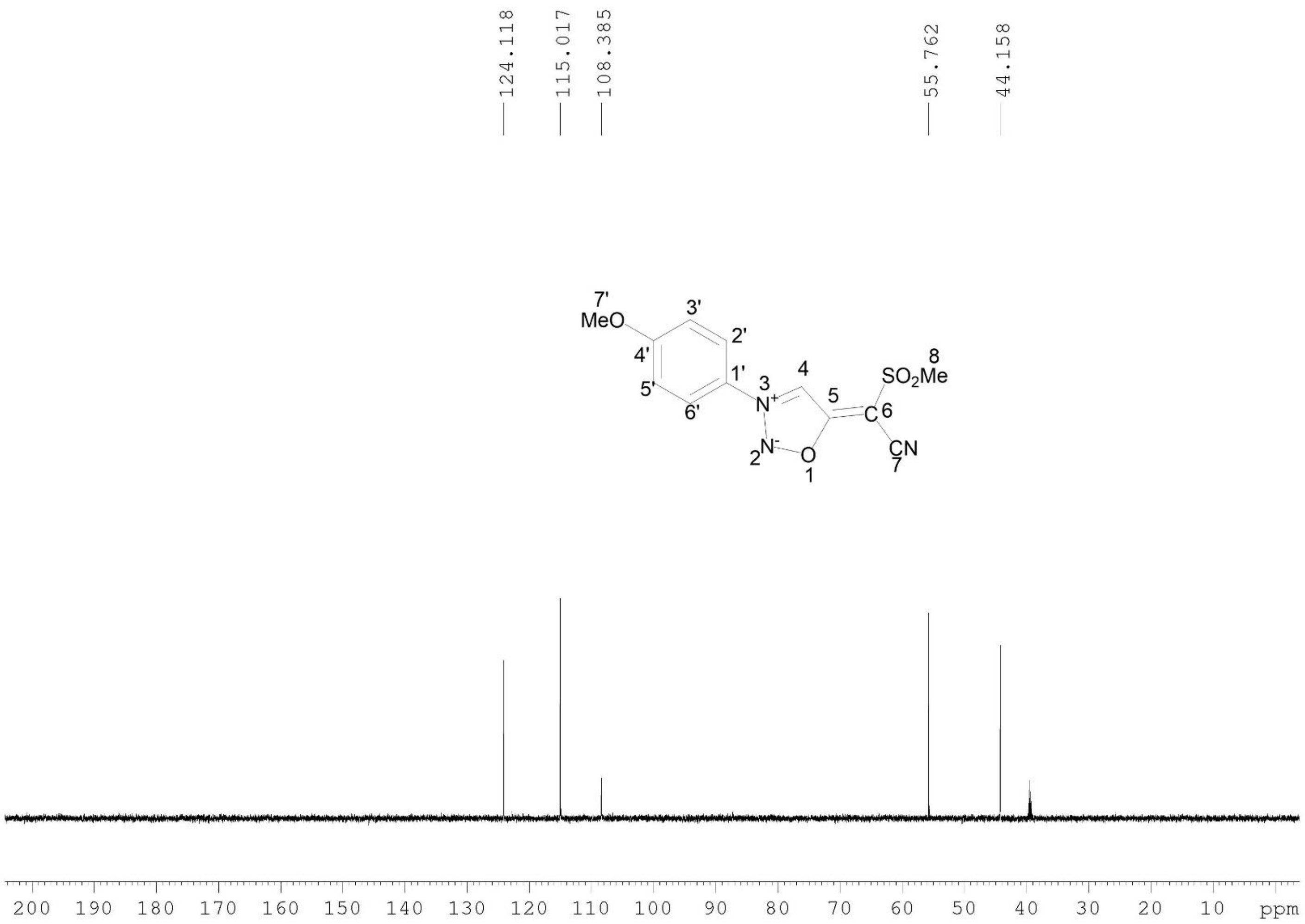

2l ^{1}H-NMR (600 MHz)

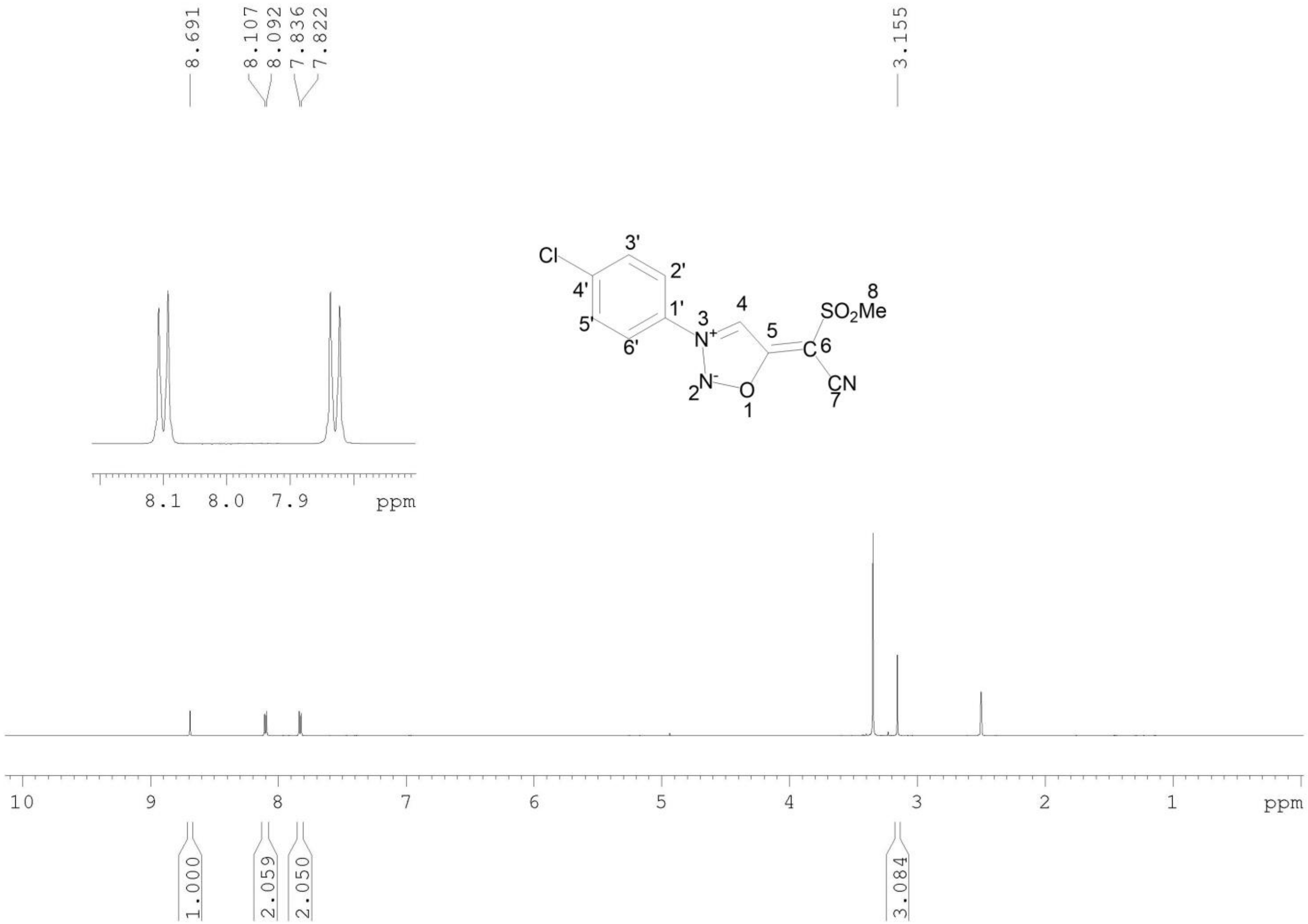

2l ^{13}C-NMR (150 MHz)

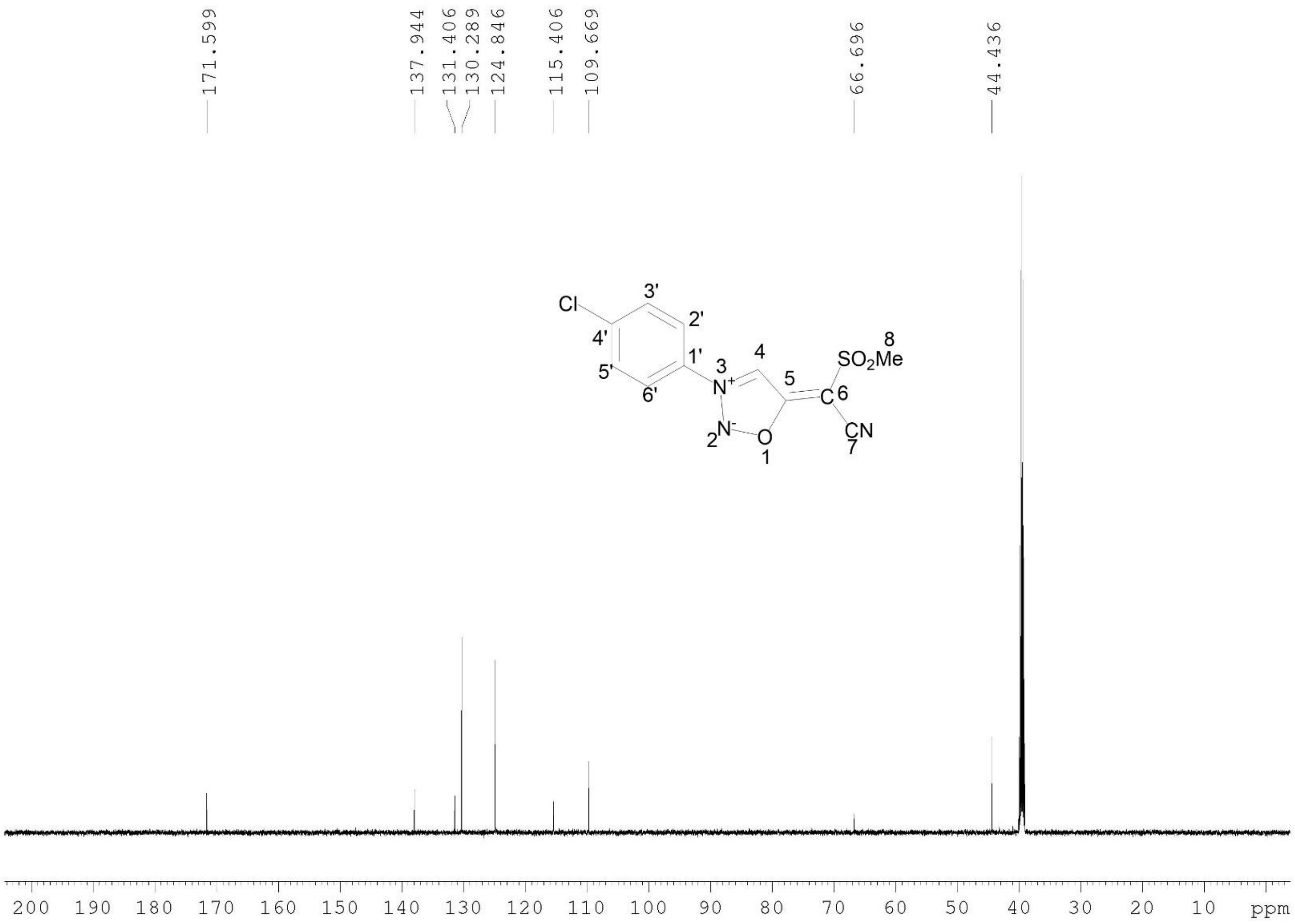

2l ^{13}C-NMR DEPT (150 MHz)

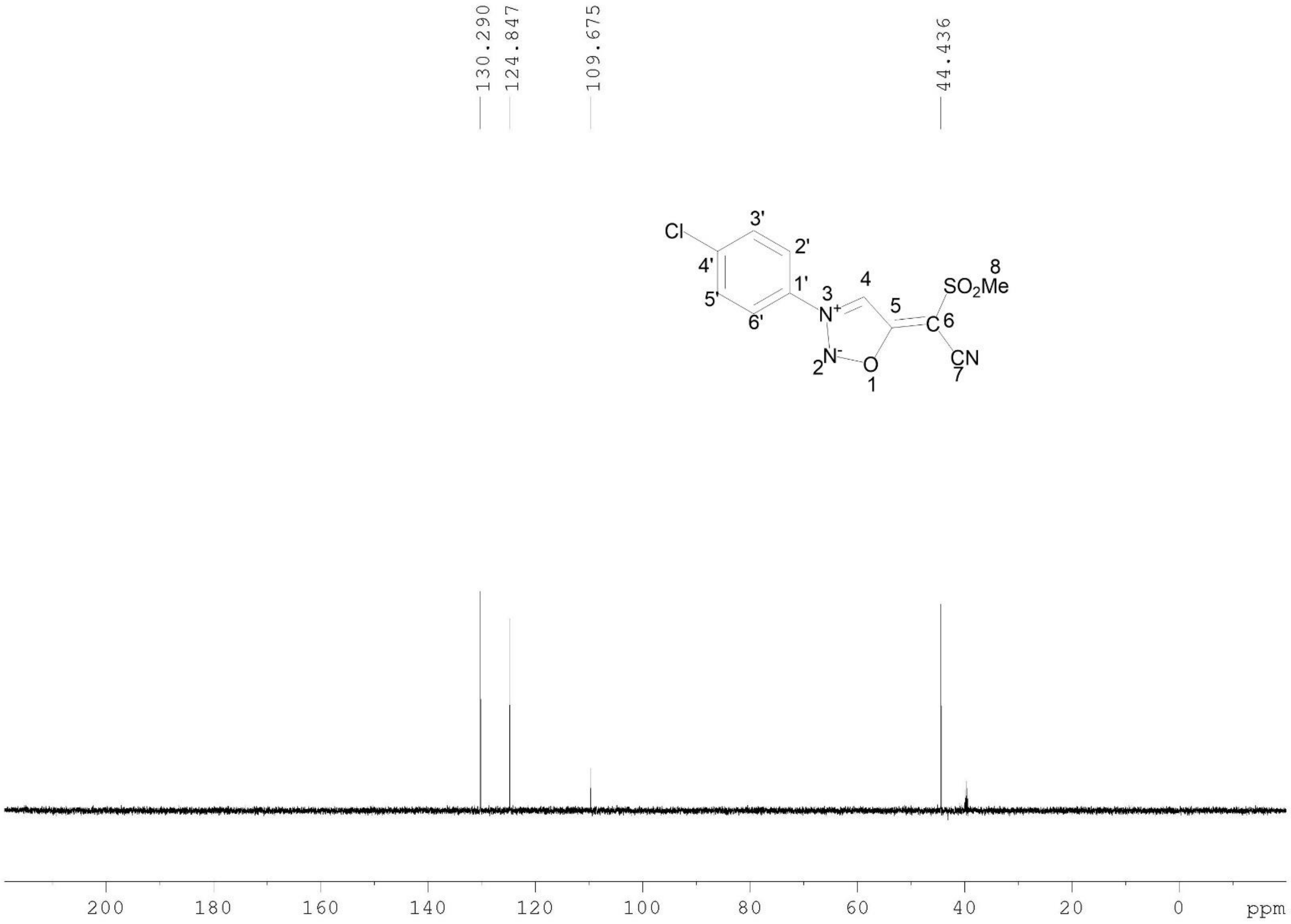

4a ^{1}H-NMR (600 MHz)

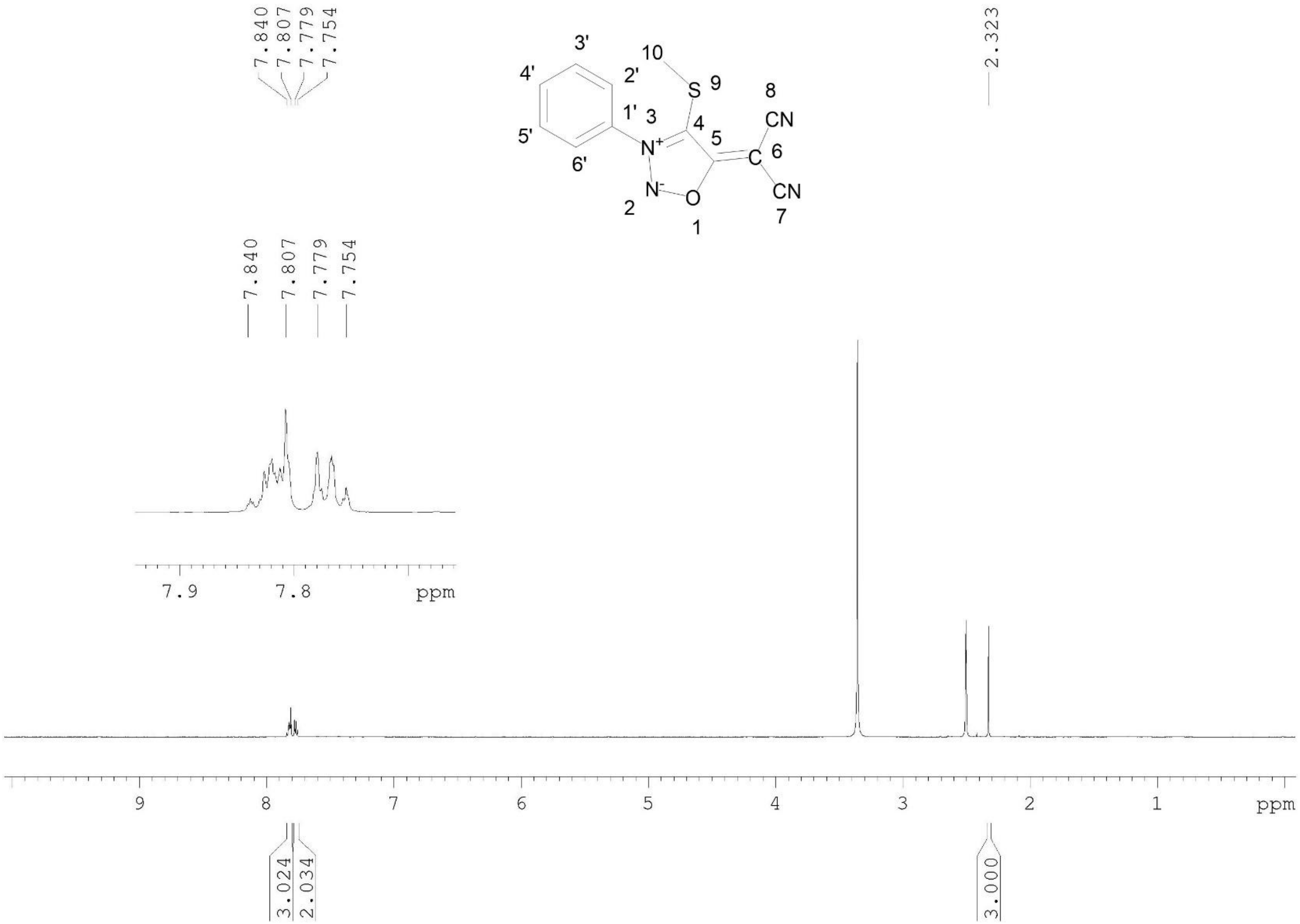

4a ^{13}C-NMR (150 MHz)

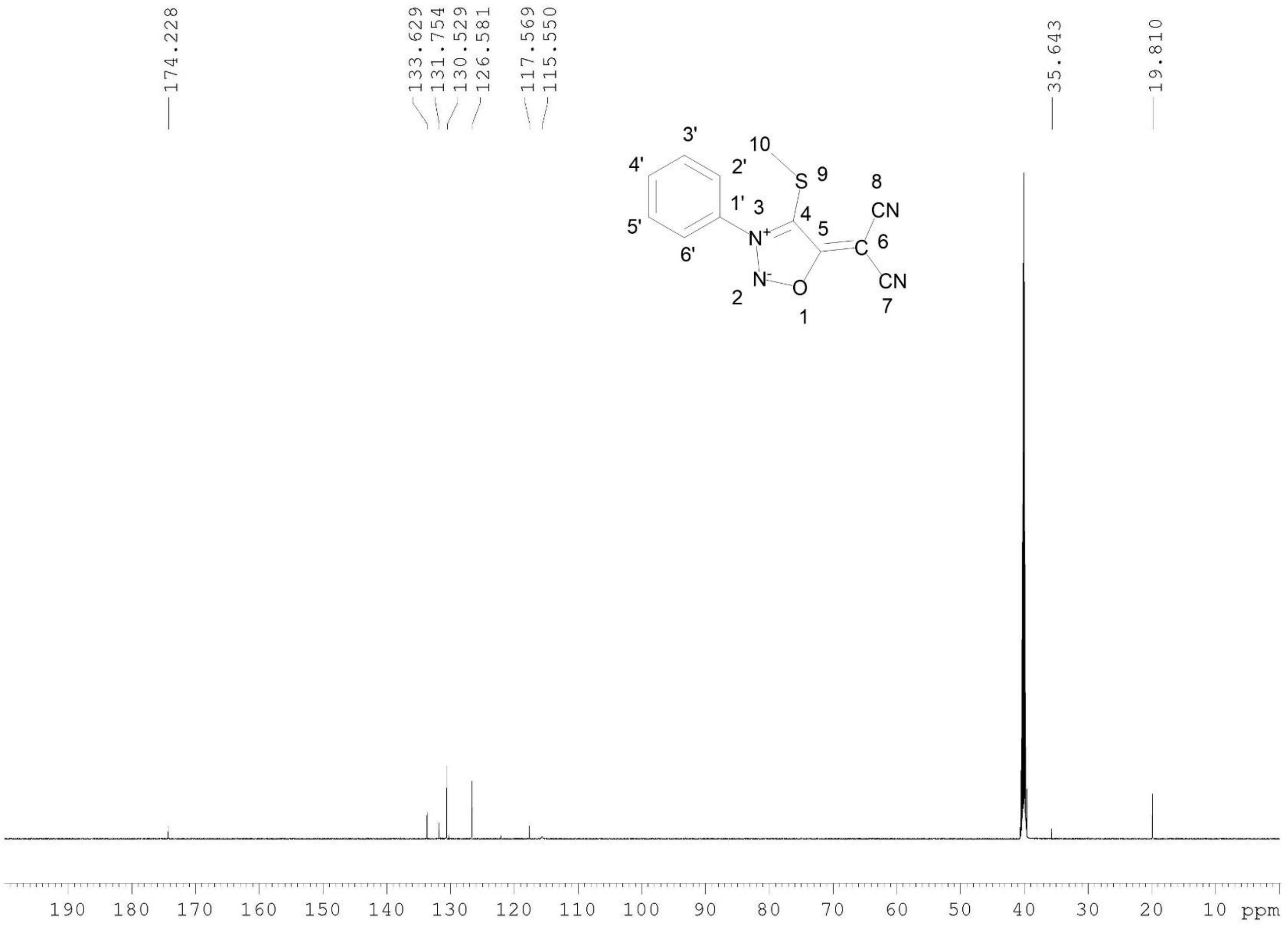

4a ^{13}C-NMR DEPT (150 MHz)

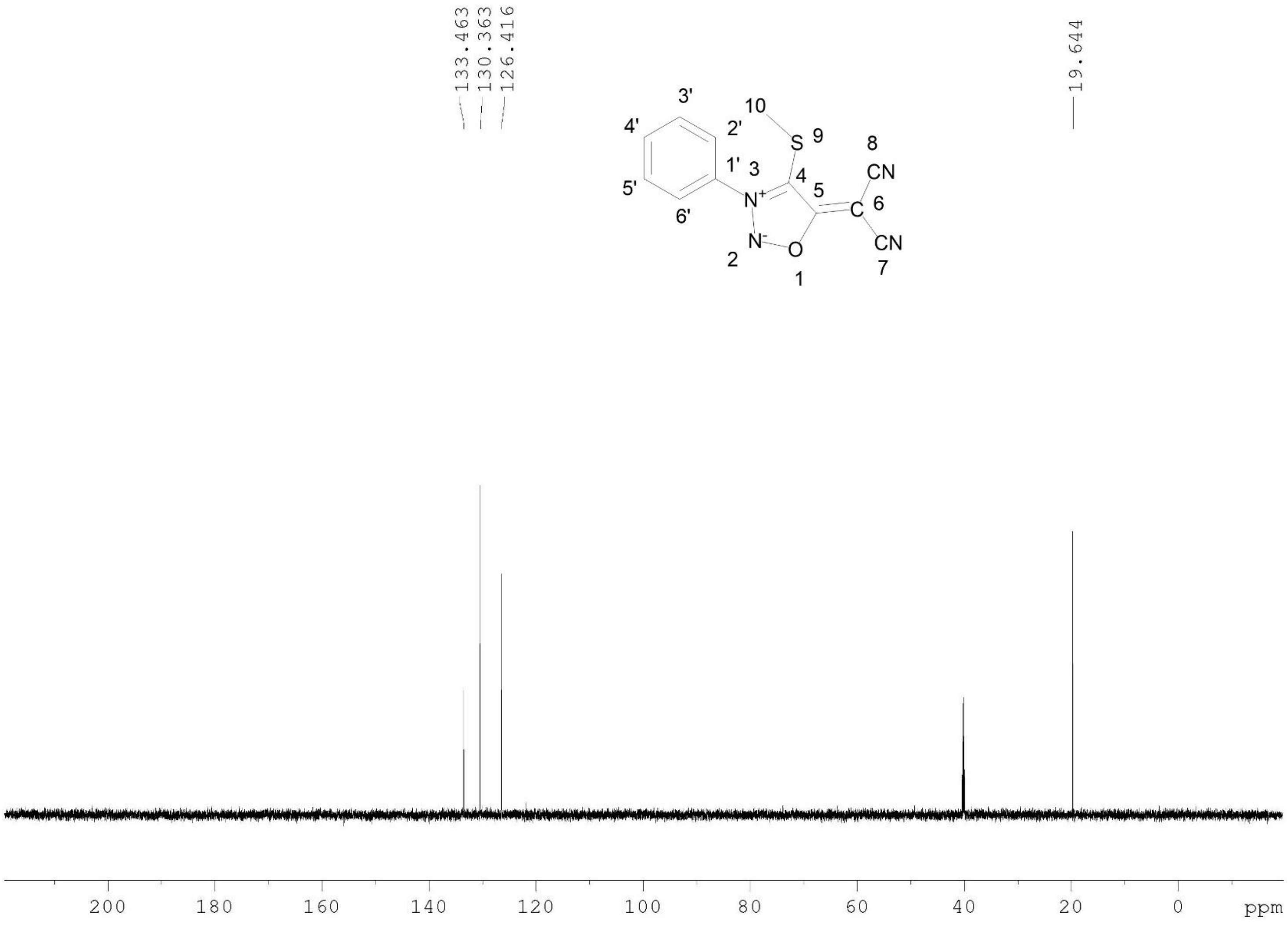

4b ^{1}H-NMR (600 MHz)

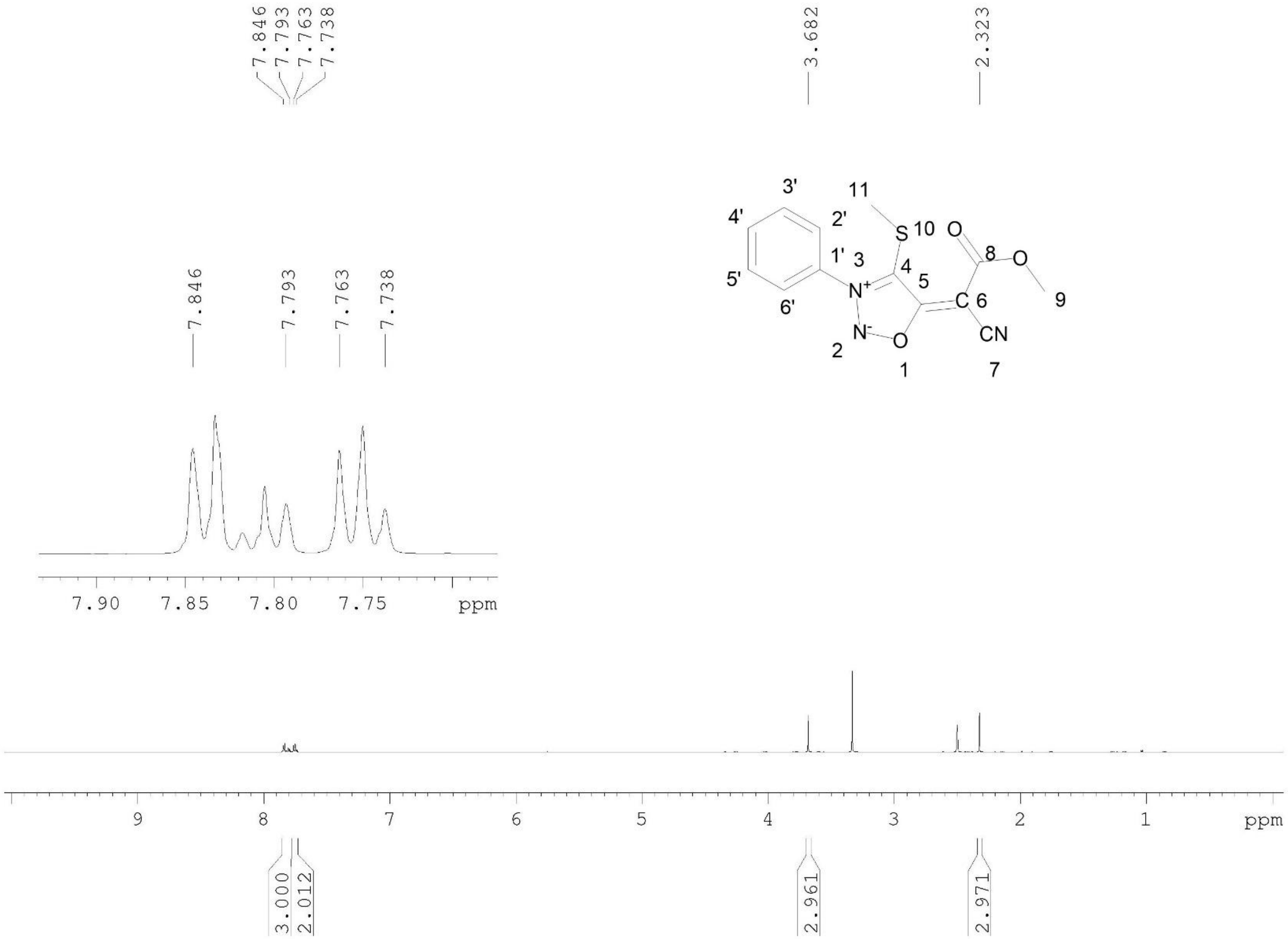

4b ^{13}C-NMR (150 MHz)

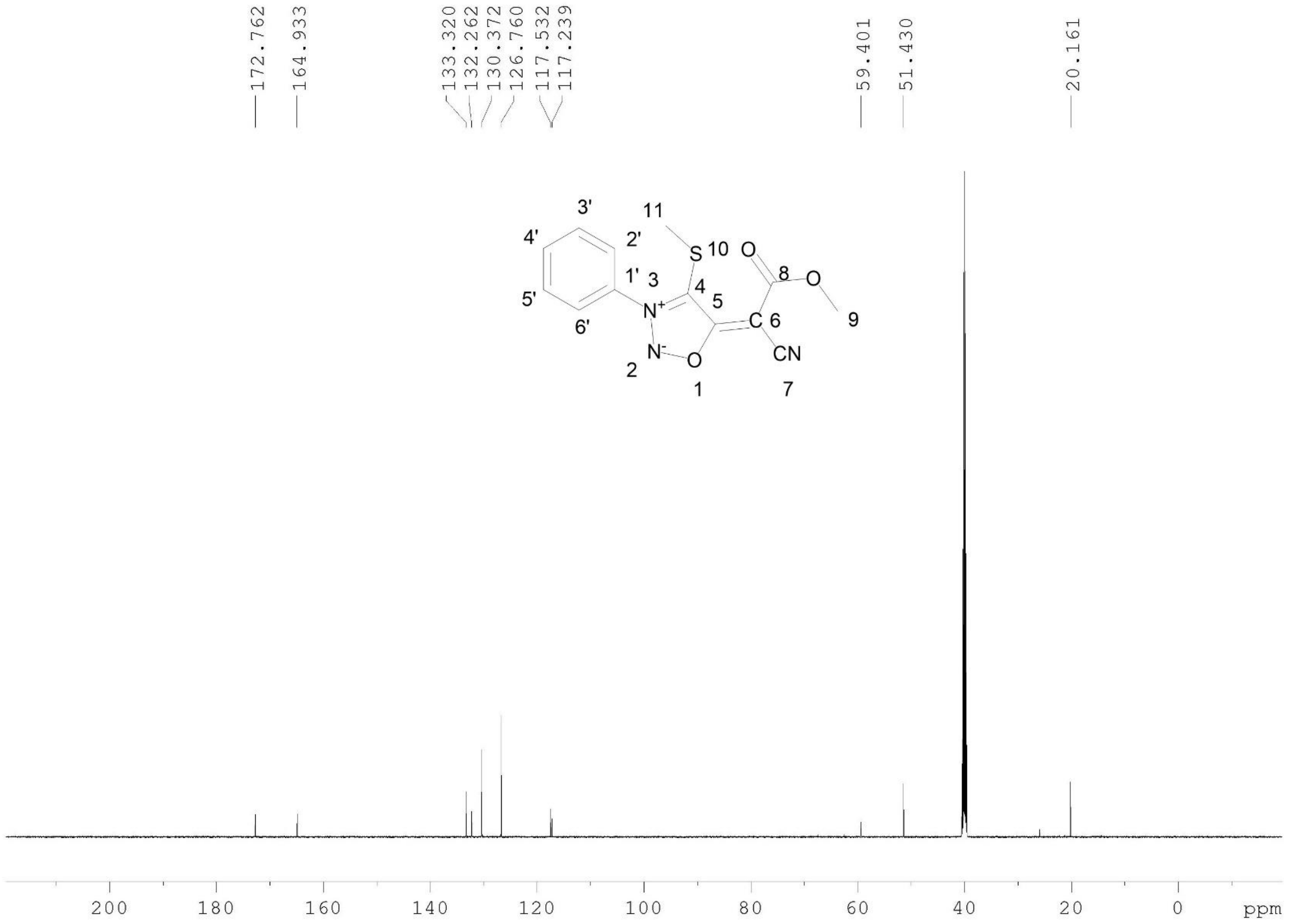

4b ^{13}C-NMR DEPT (150 MHz)

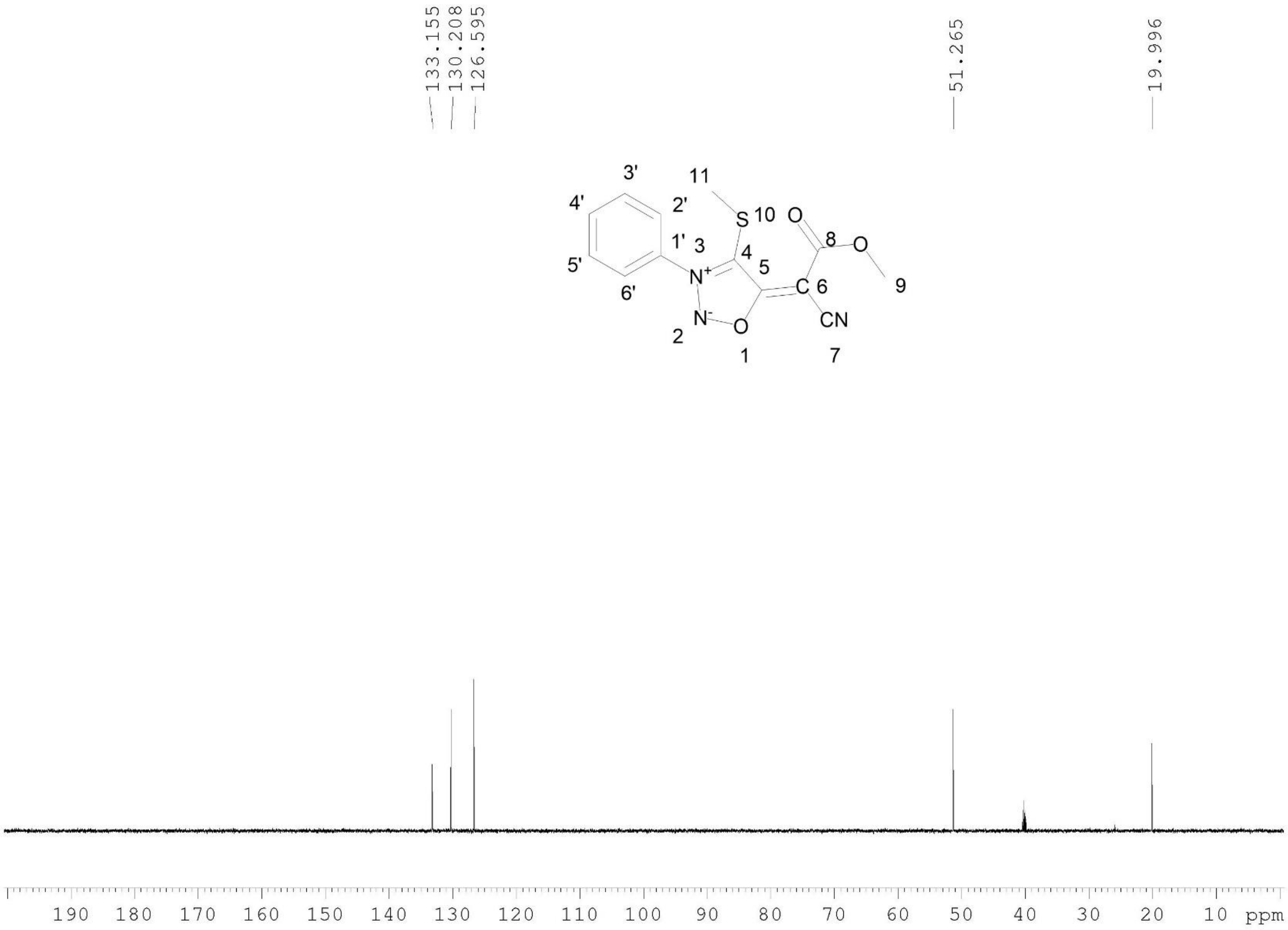

4c ^{1}H-NMR (600 MHz)

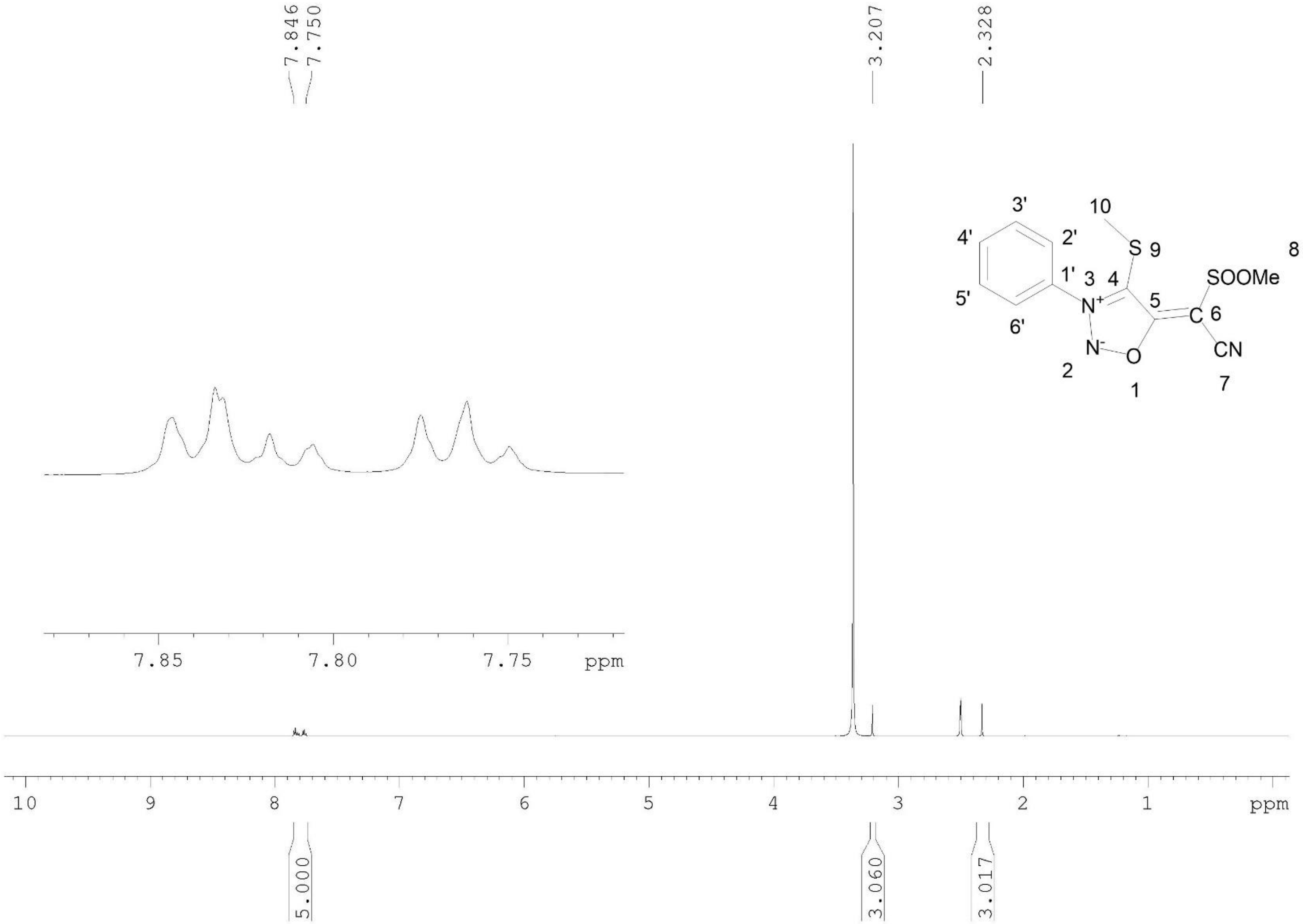

4c ^{13}C-NMR (150 MHz)

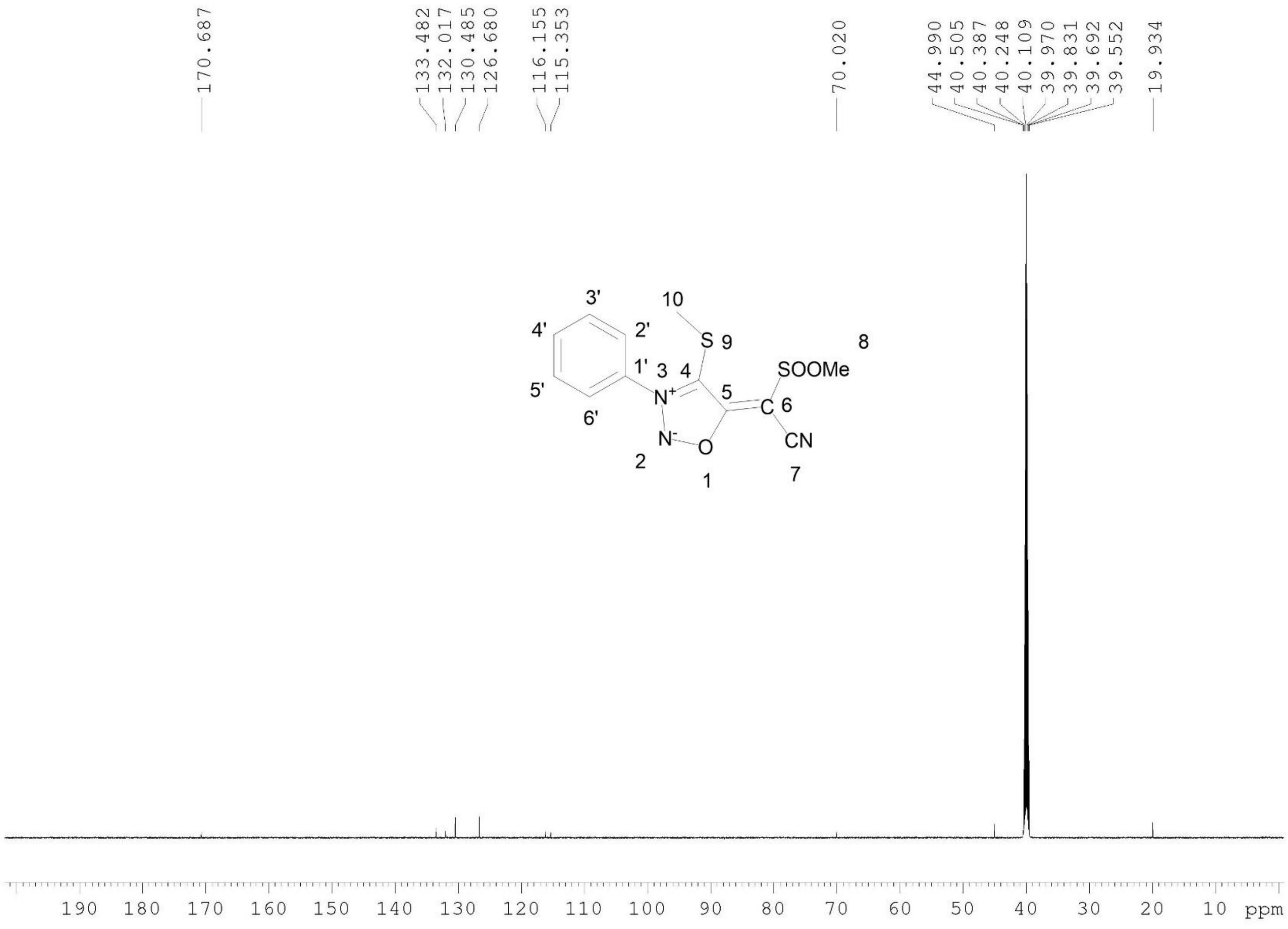

4c ^{13}C-NMR DEPT (150 MHz)

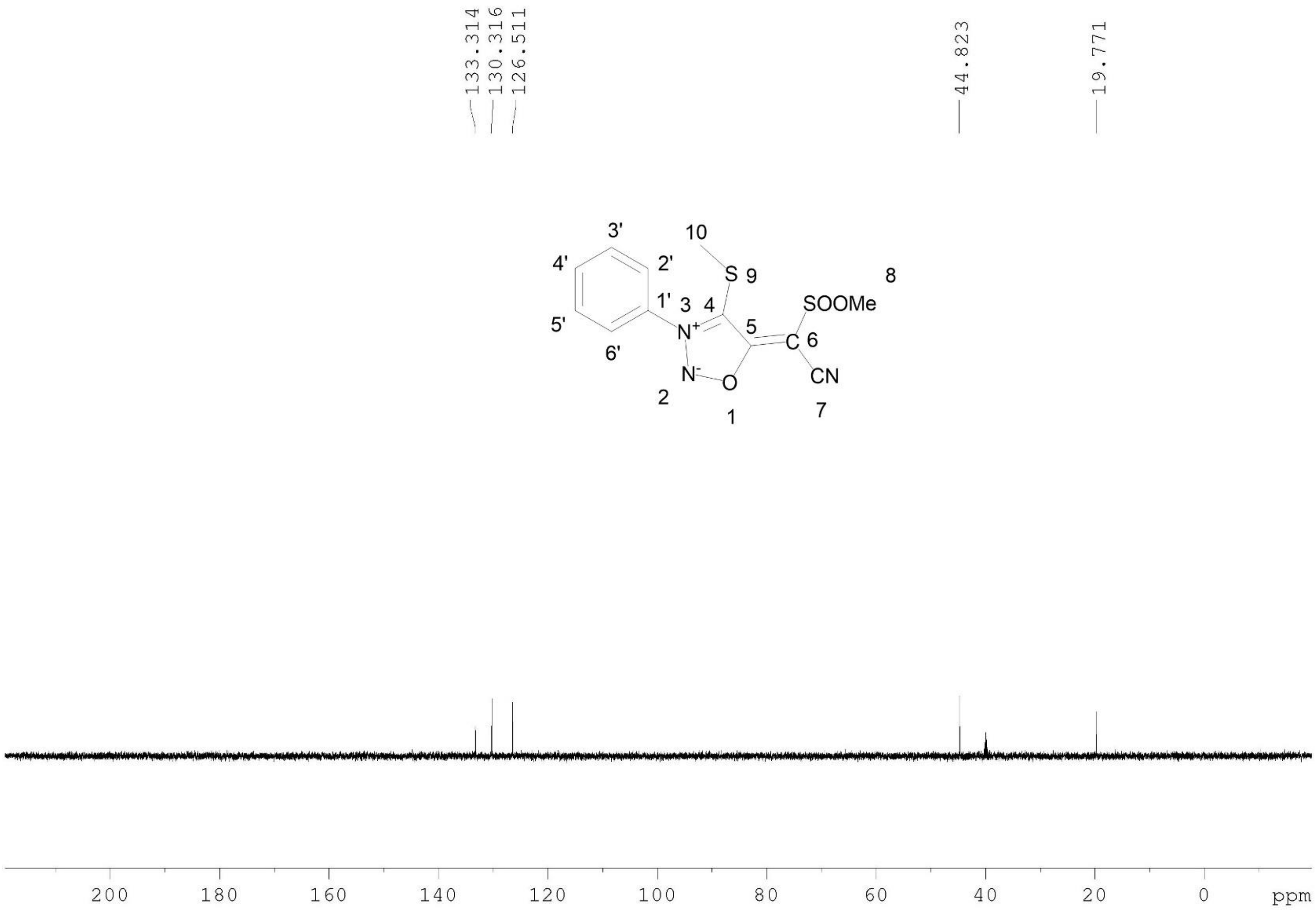

5a ^{1}H-NMR (600 MHz)

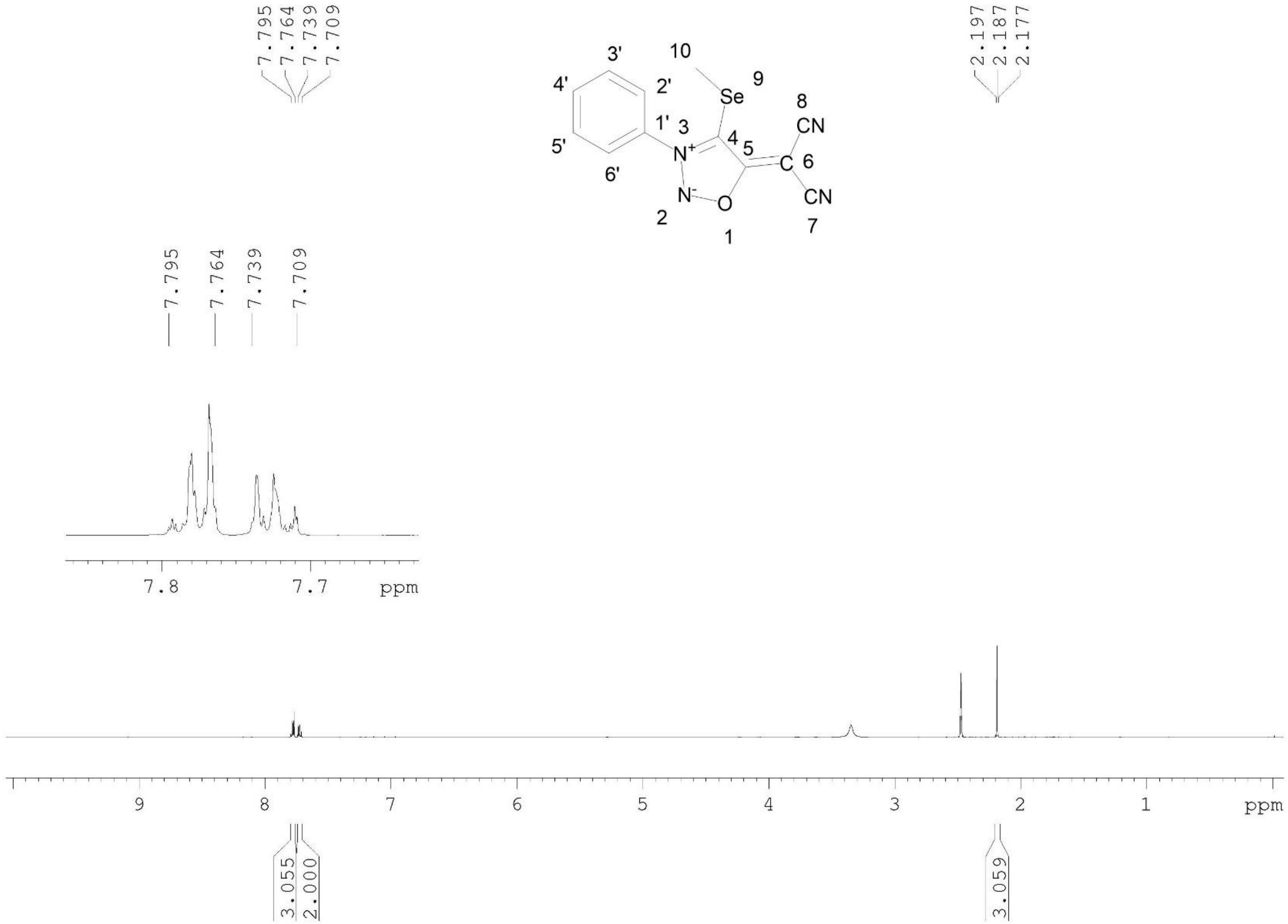

5a ^{13}C-NMR (150 MHz)

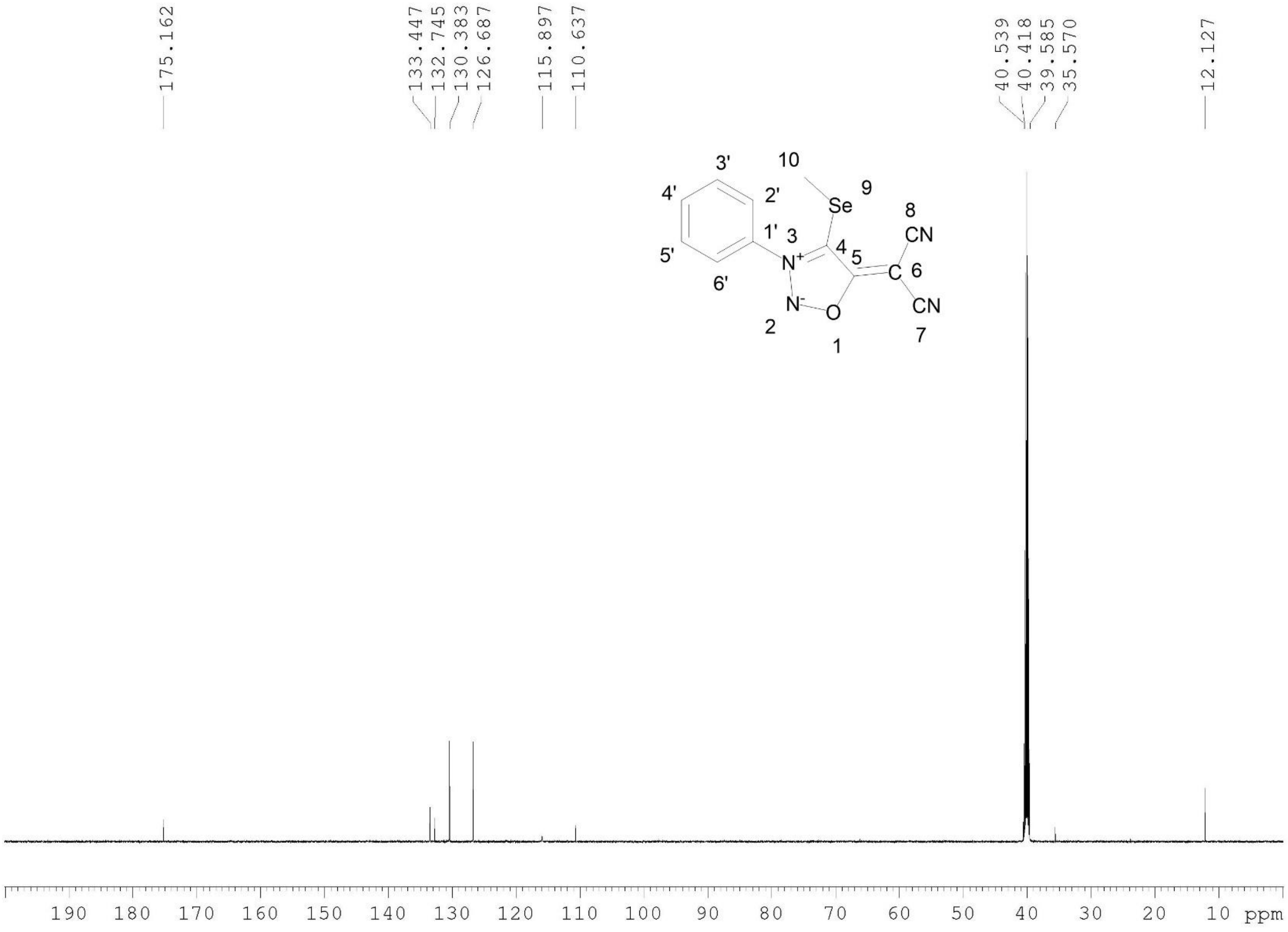

5a ^{77}Se-NMR (114 MHz)

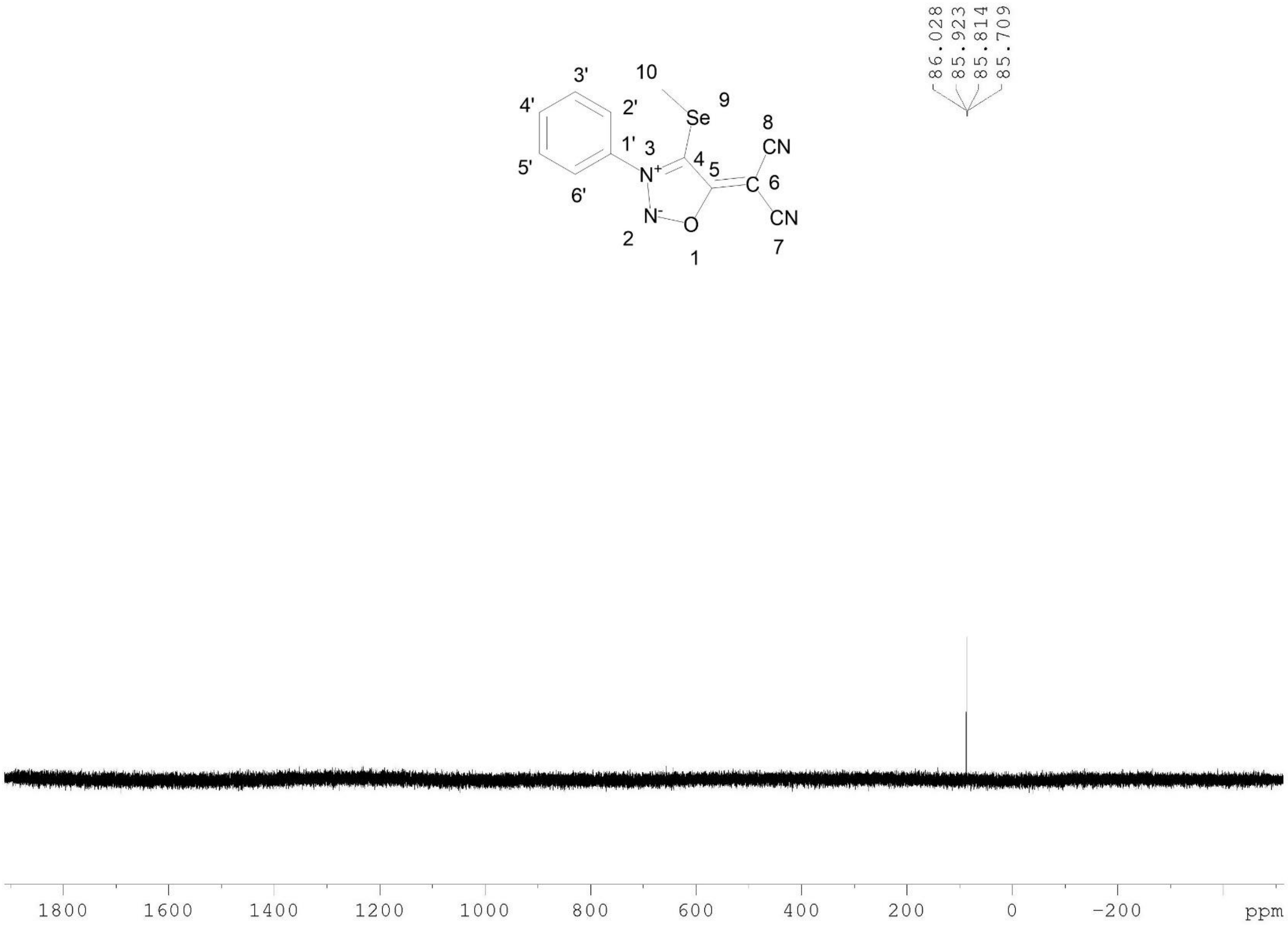

5a ^{13}C-NMR DEPT (150 MHz)

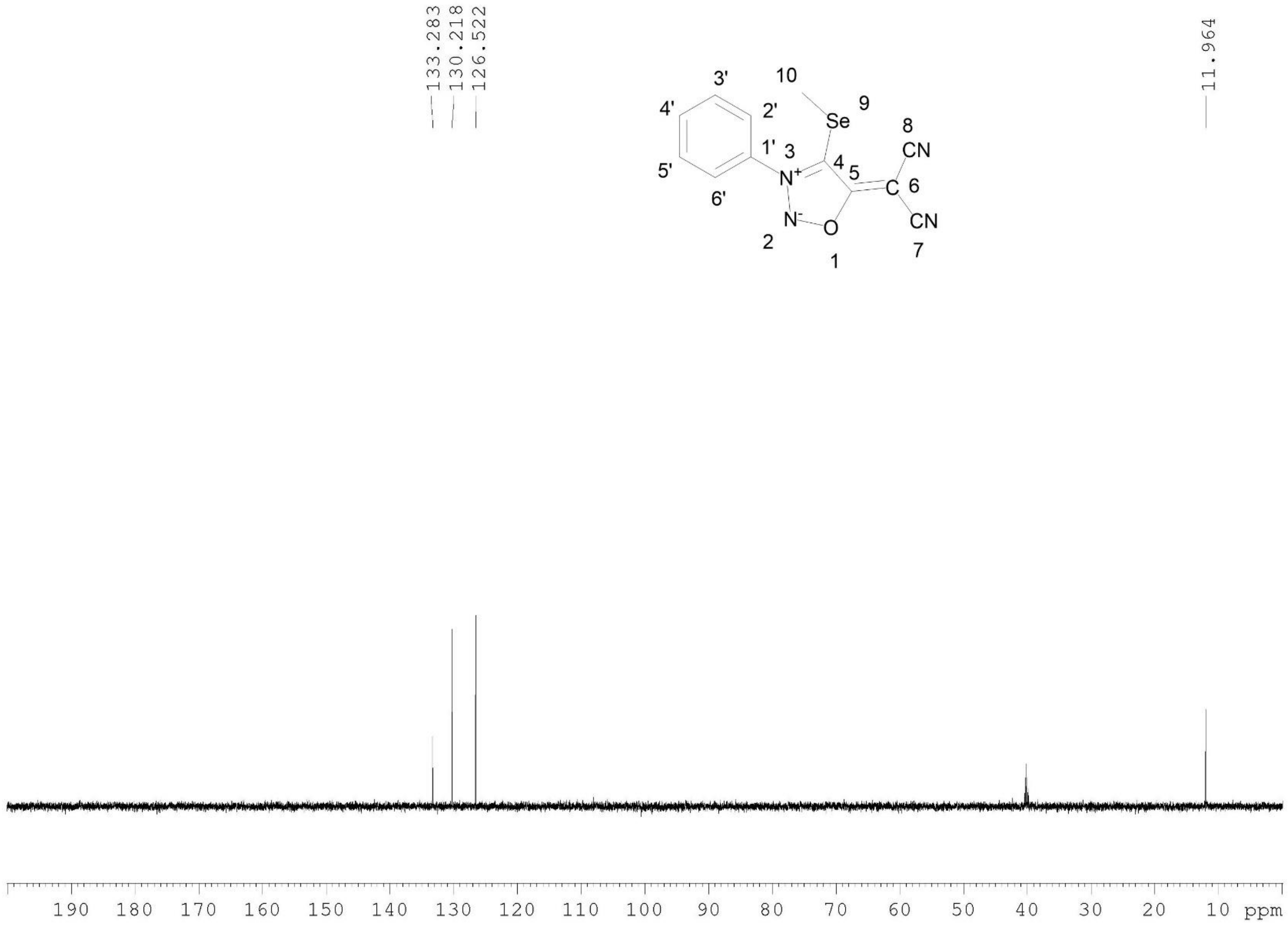

5b ^{1}H-NMR (600 MHz)

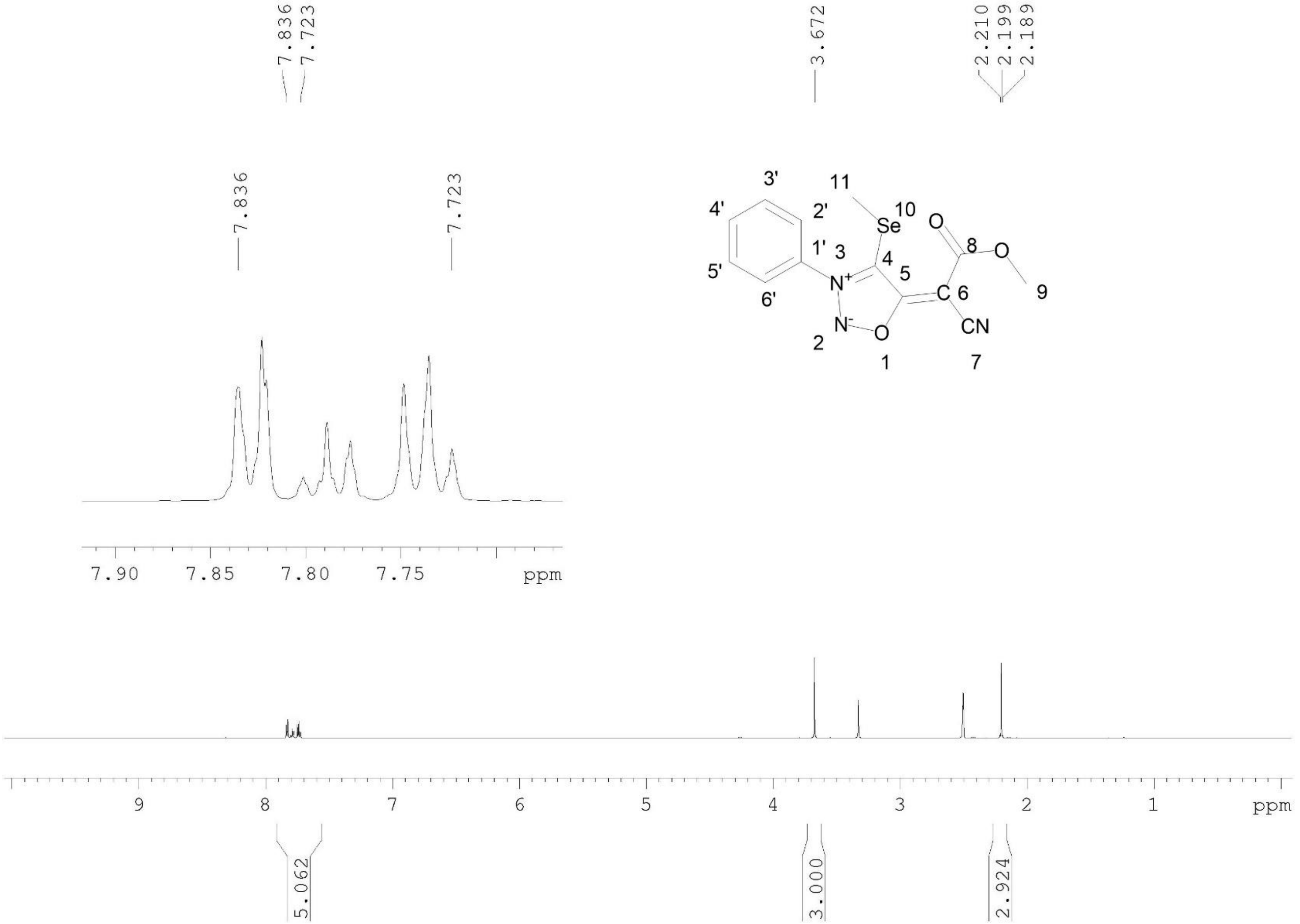

5b ^{13}C-NMR (150 MHz)

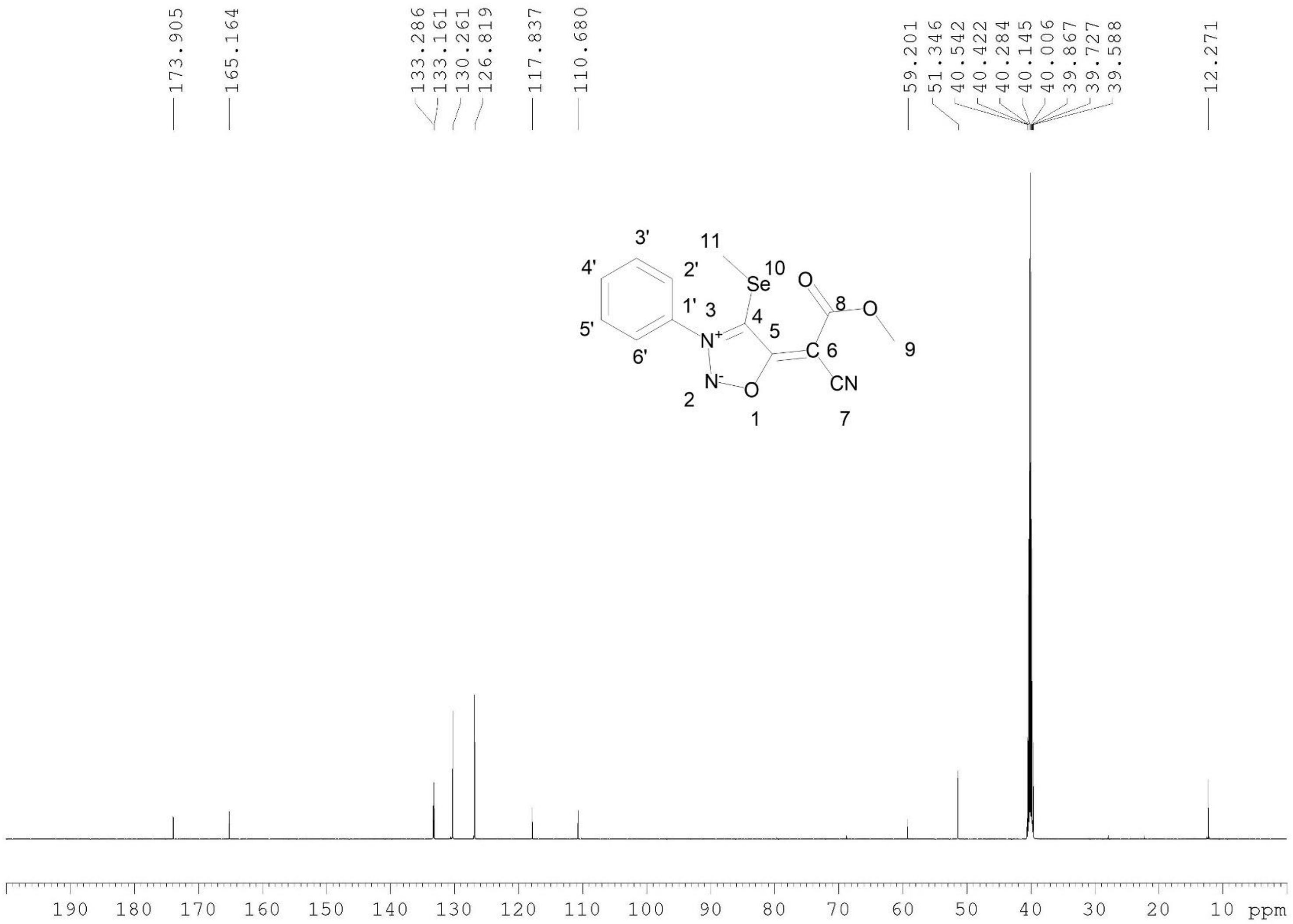

/ /

5b ^{77}Se-NMR (114 MHz)

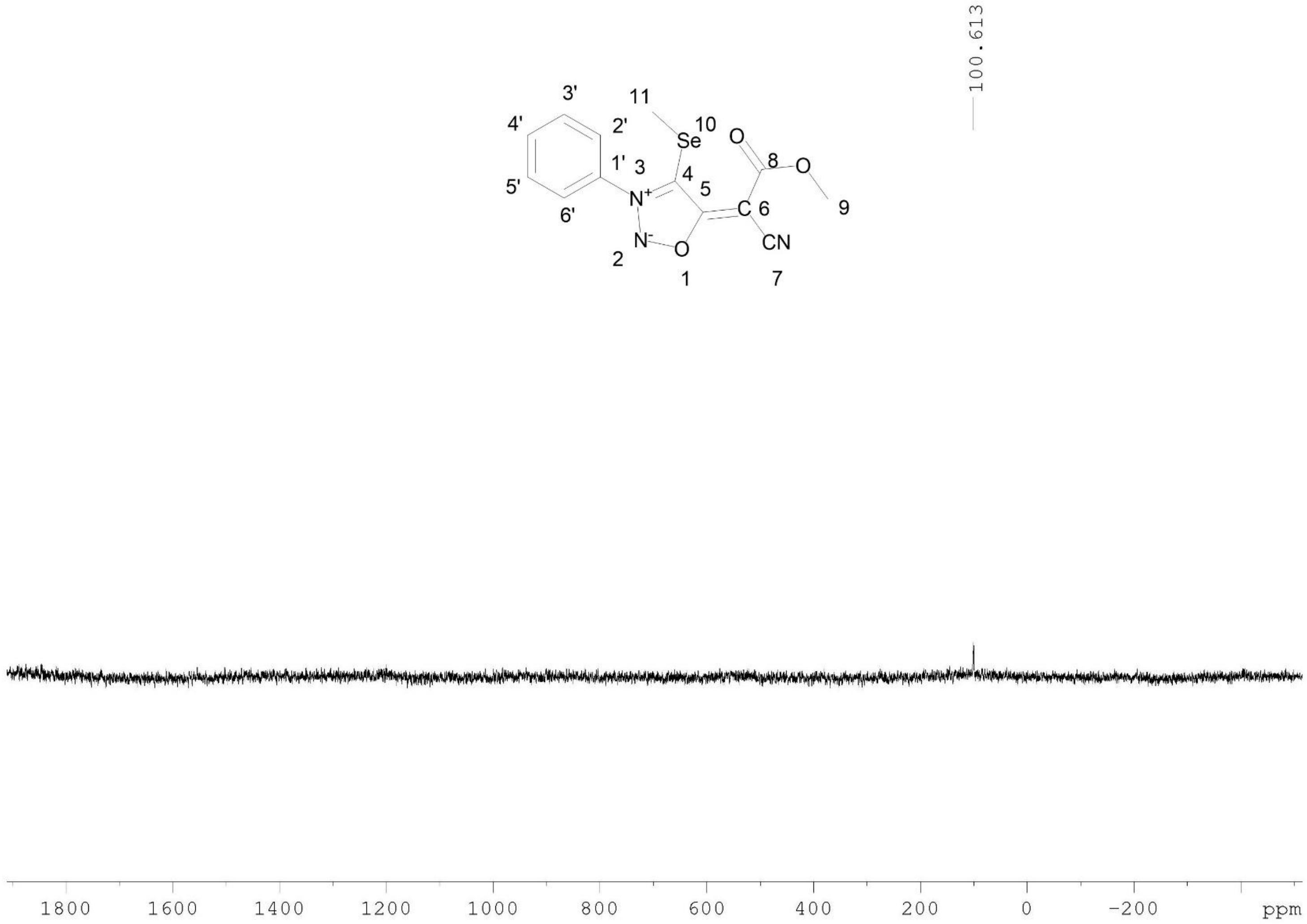

5b ^{13}C-NMR DEPT (150 MHz)

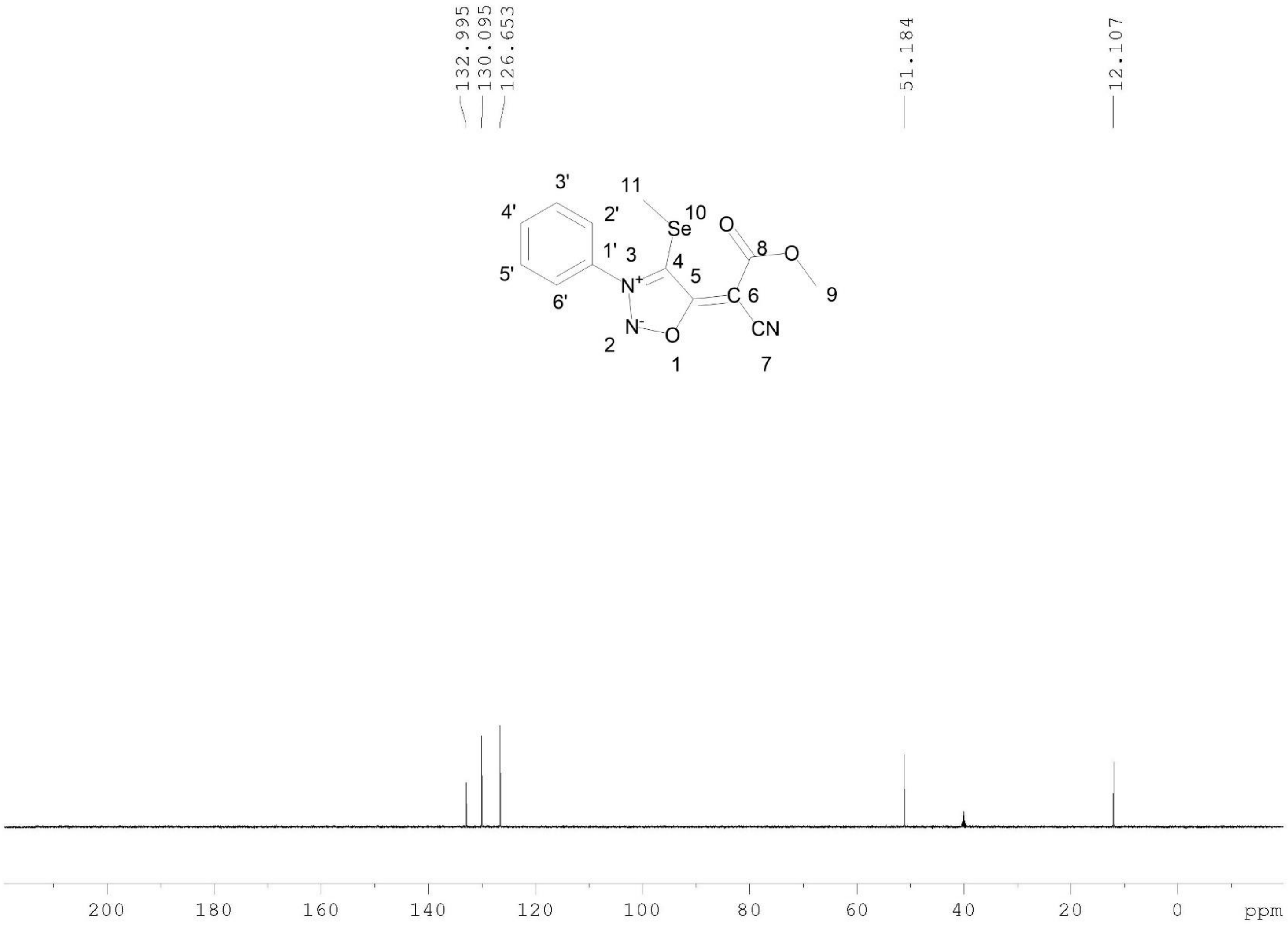

5c ¹H-NMR DEPT (600 MHz)

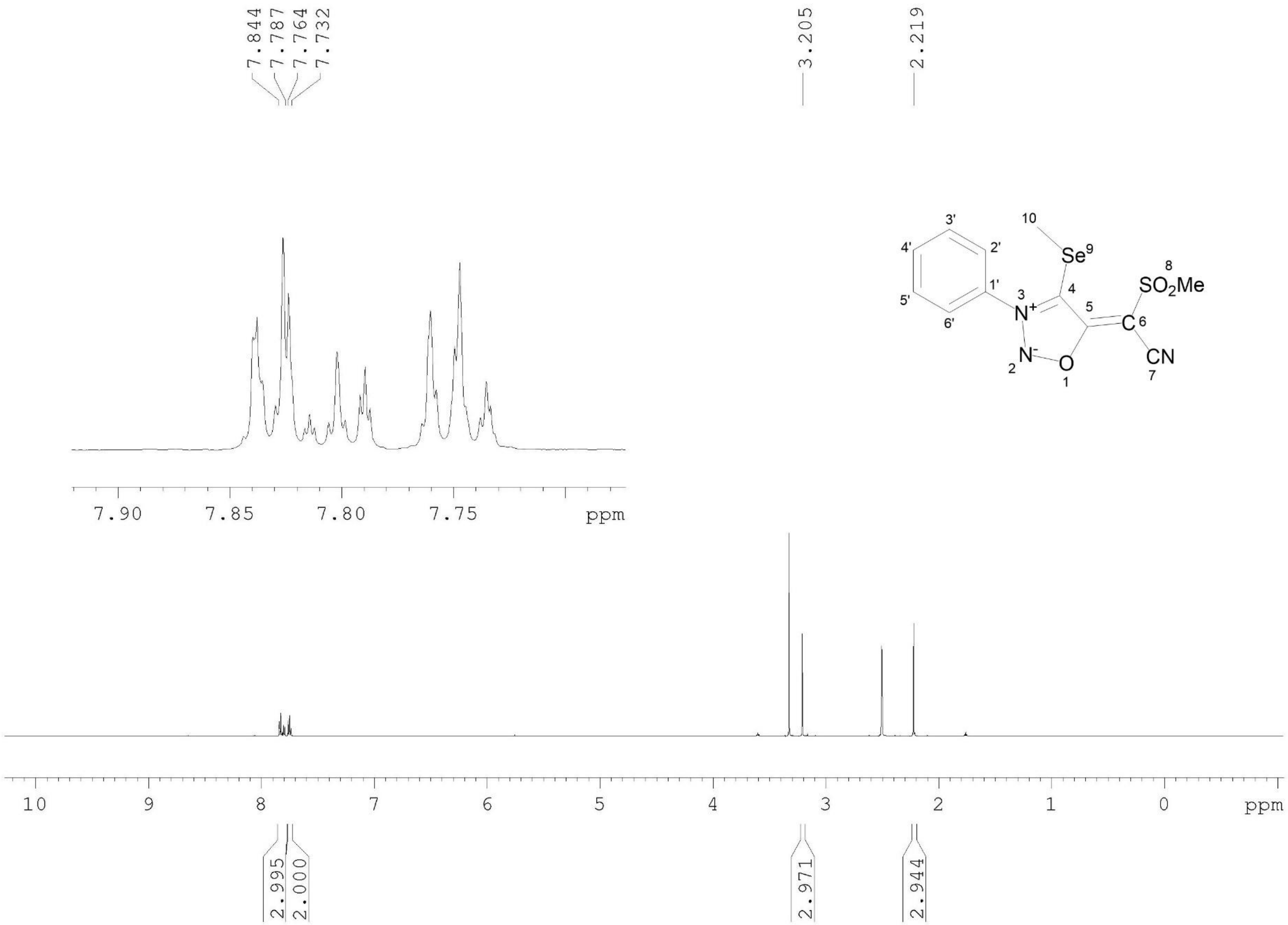

5c ^{13}C-NMR (150 MHz)

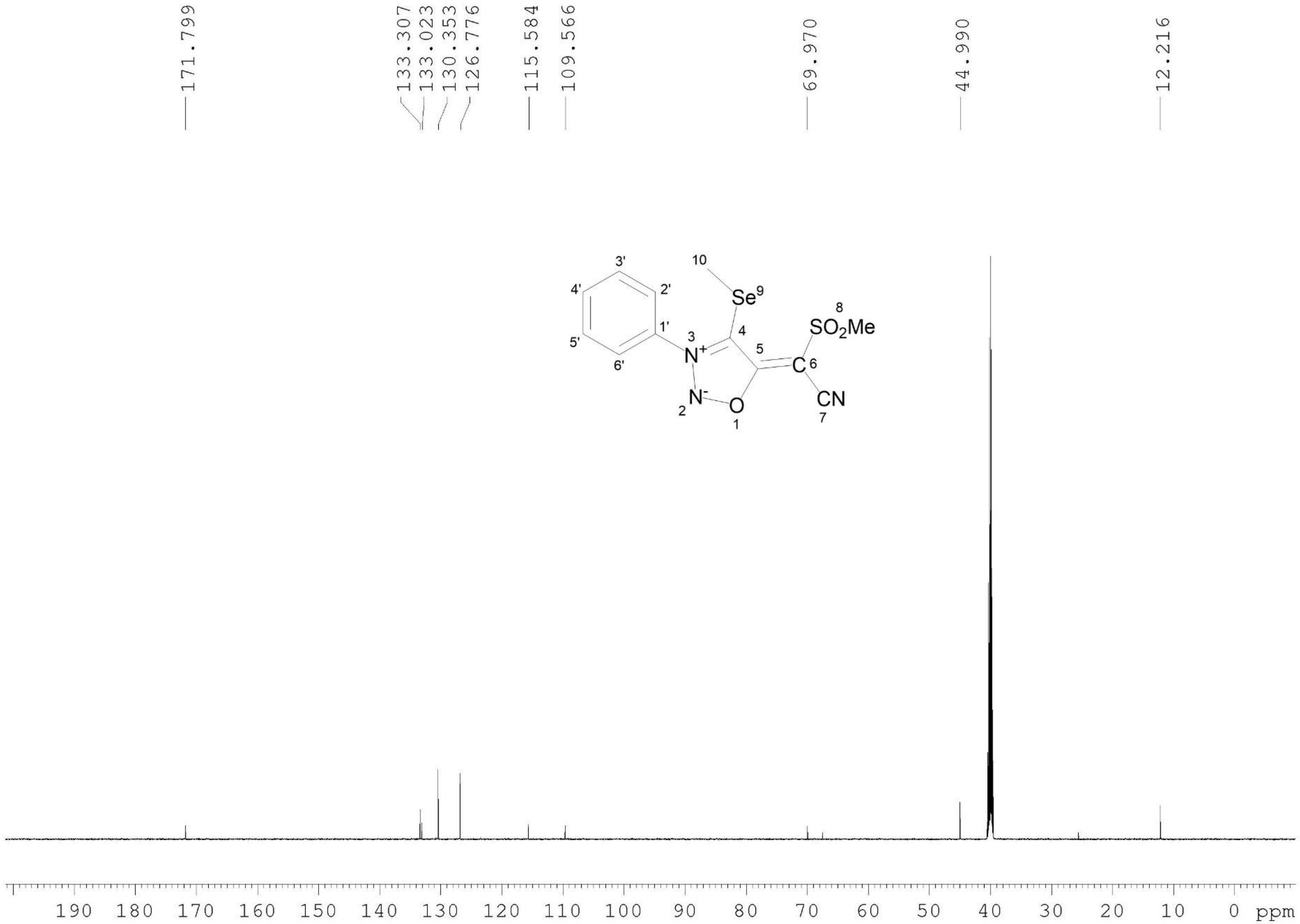

5c ^{77}Se-NMR (114 MHz)

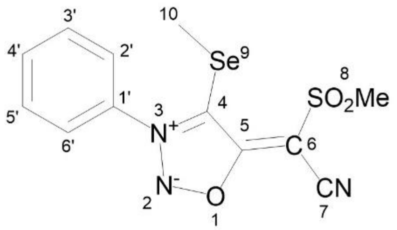

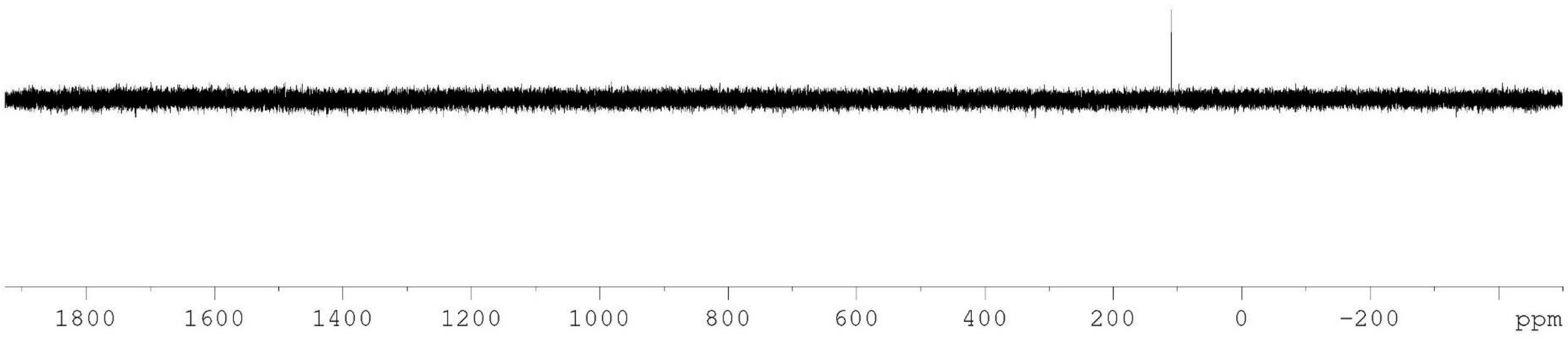

5c ^{13}C-NMR DEPT (150 MHz)

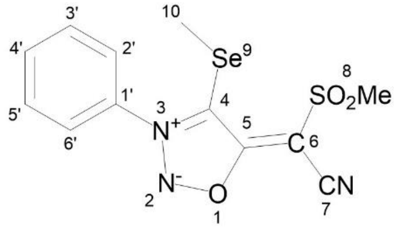

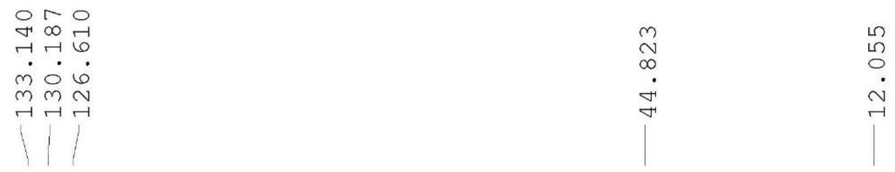

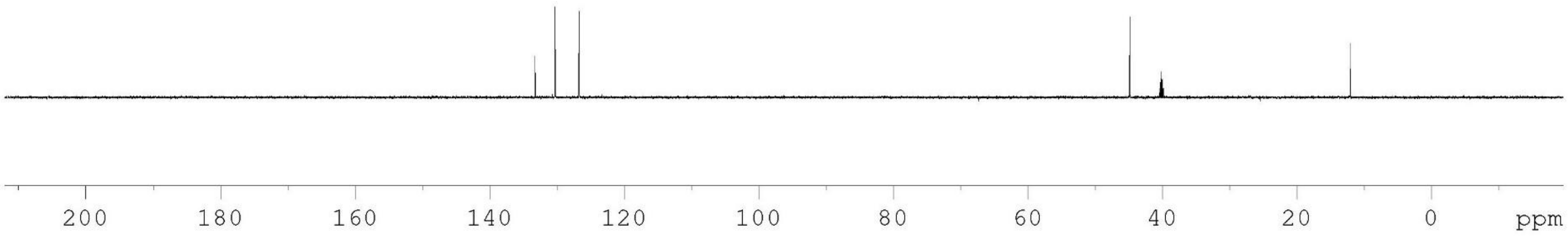

6a ^{1}H-NMR (400 MHz)

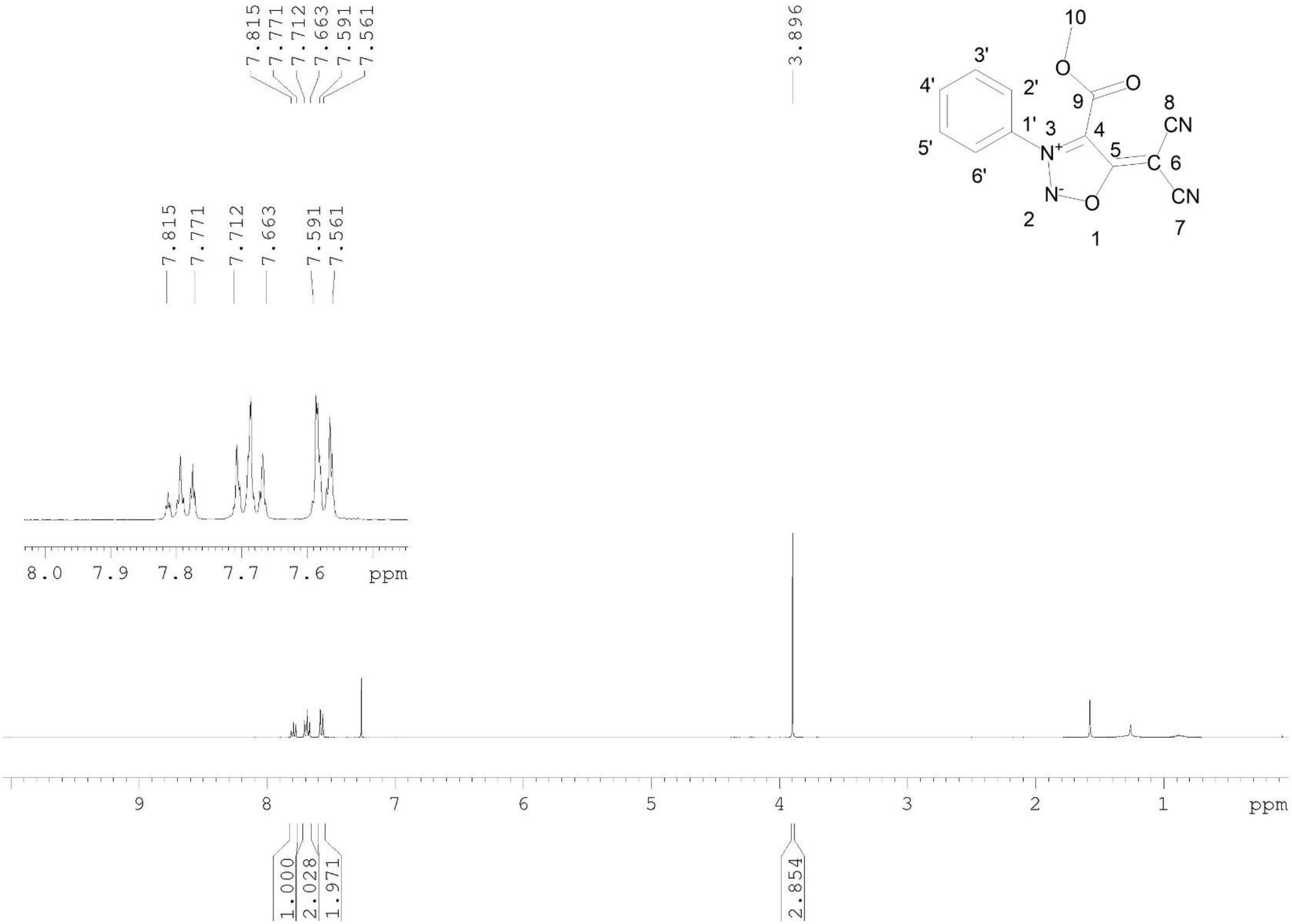

6a ^{13}C-NMR (100 MHz)

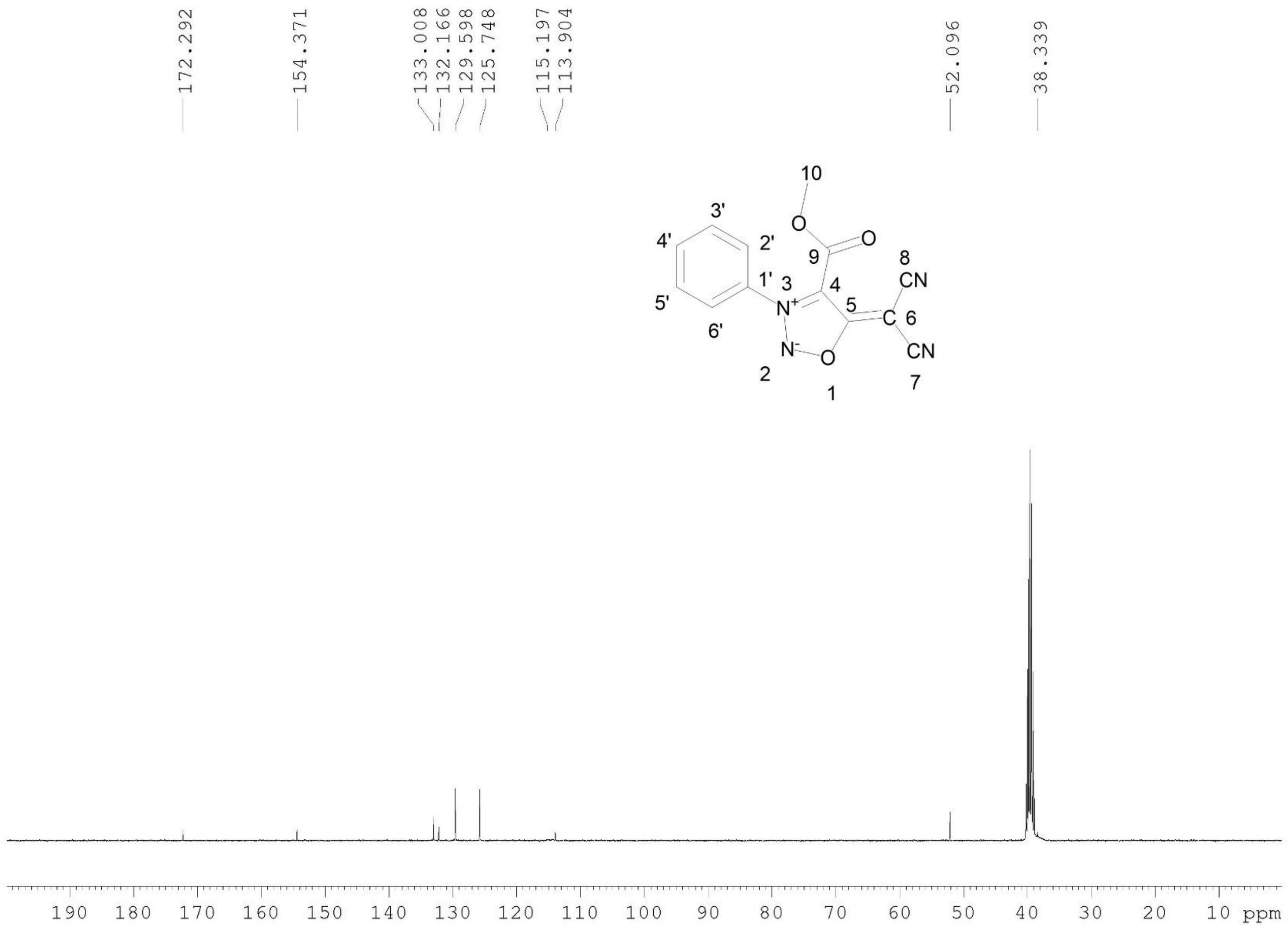

6a ^{13}C-NMR DEPT (100 MHz)

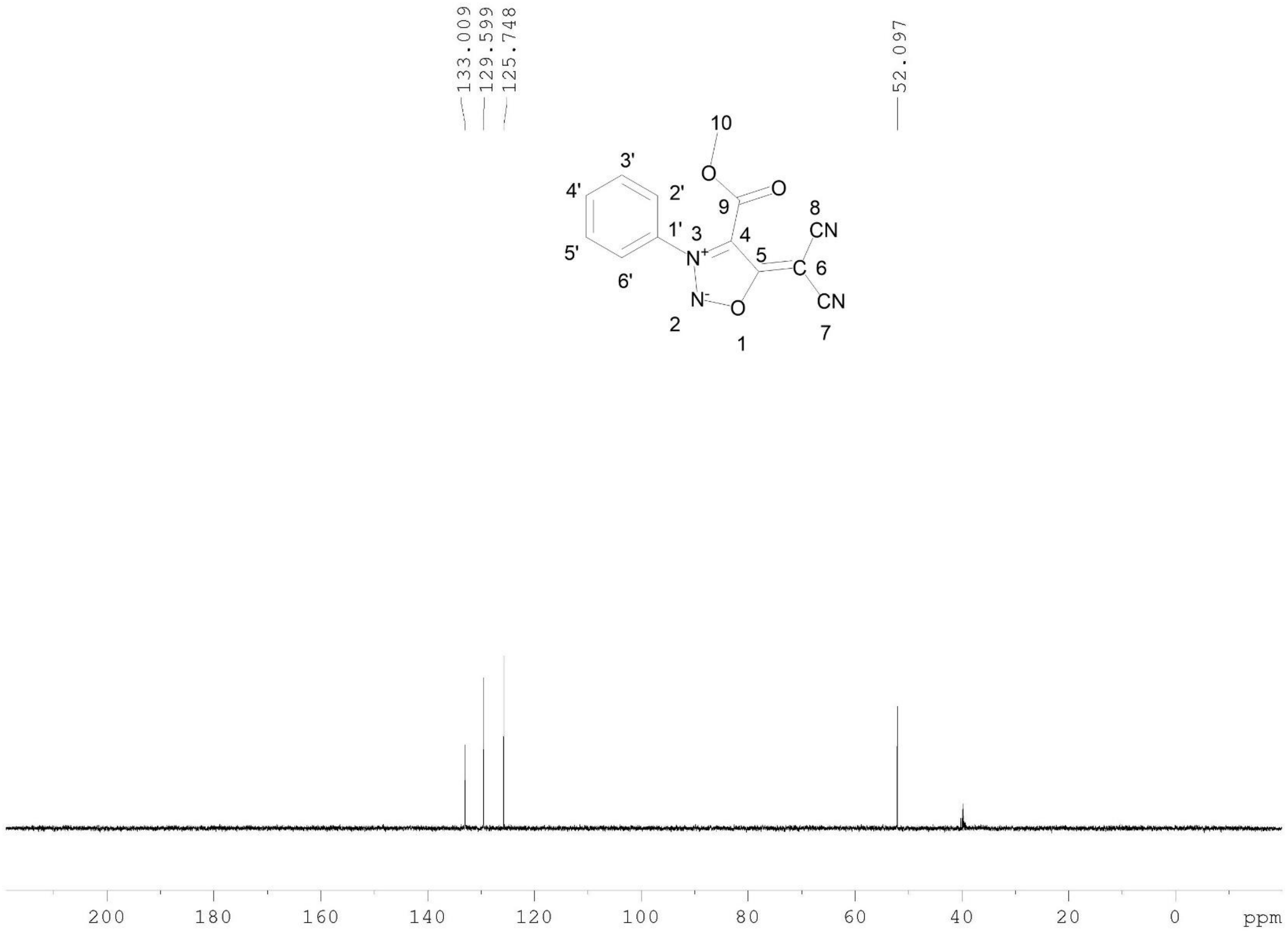

6b ^{1}H-NMR (600 MHz)

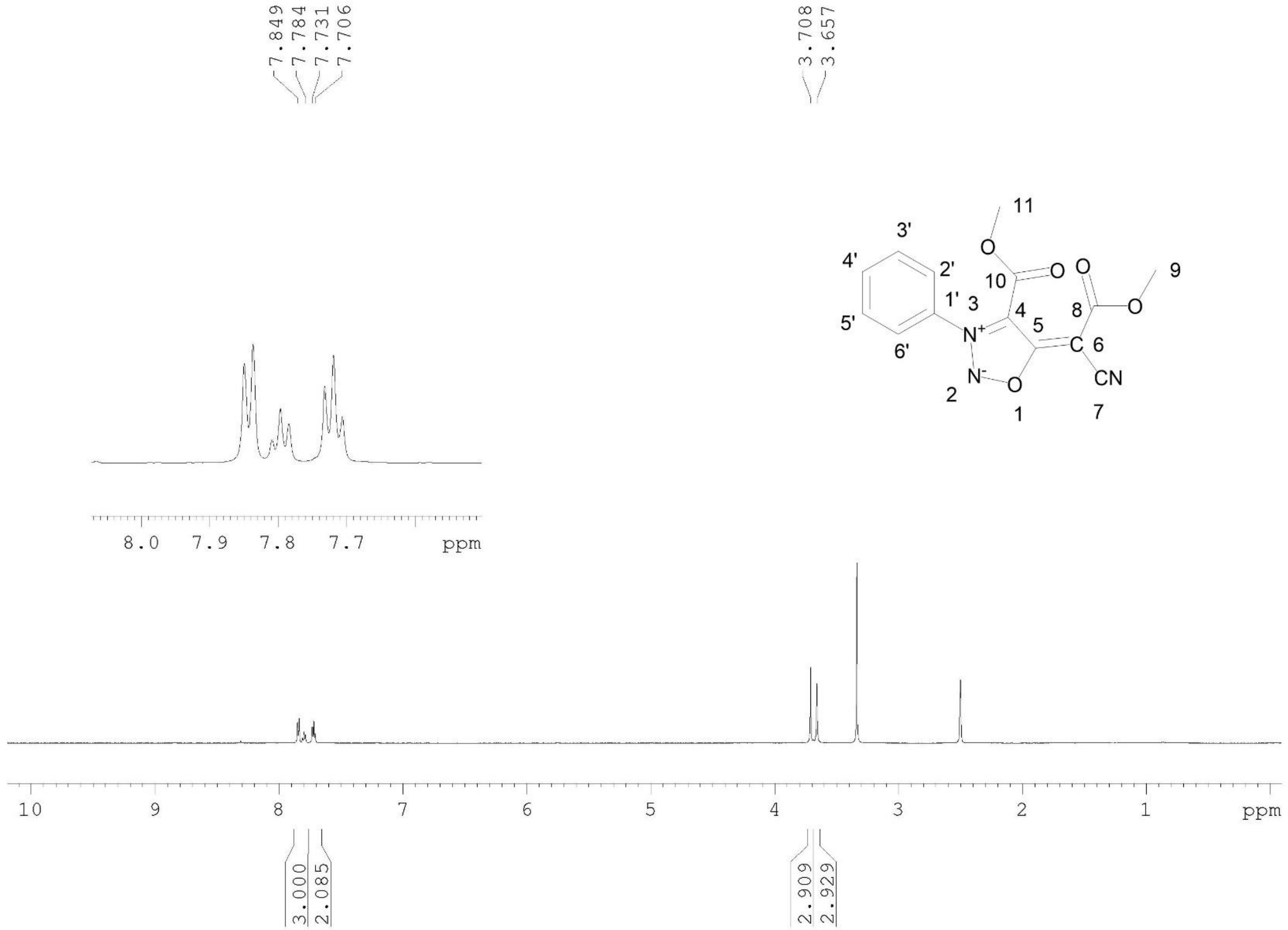

6b ^{13}C-NMR (150 MHz)

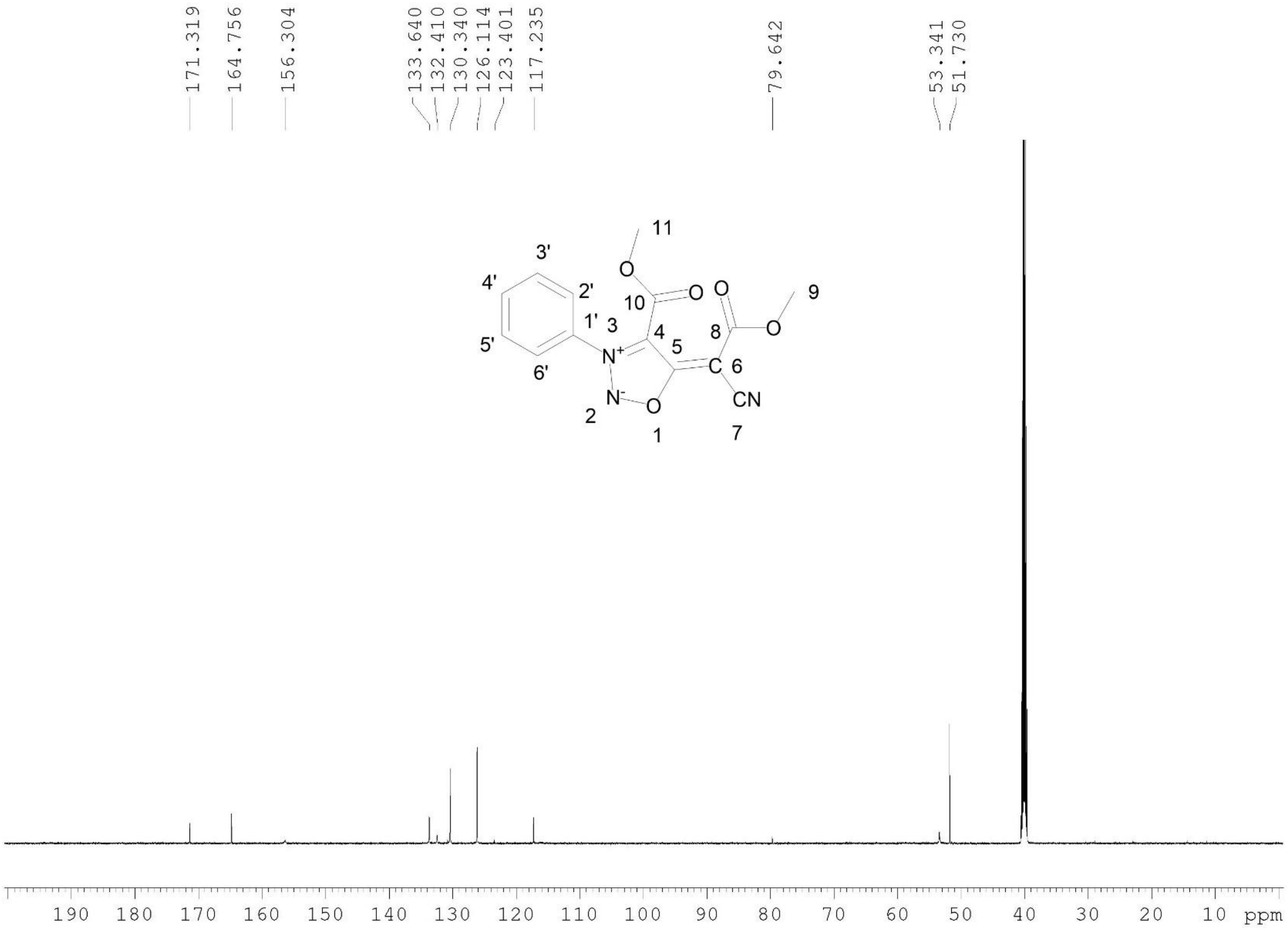

6b ^{13}C-NMR DEPT (150 MHz)

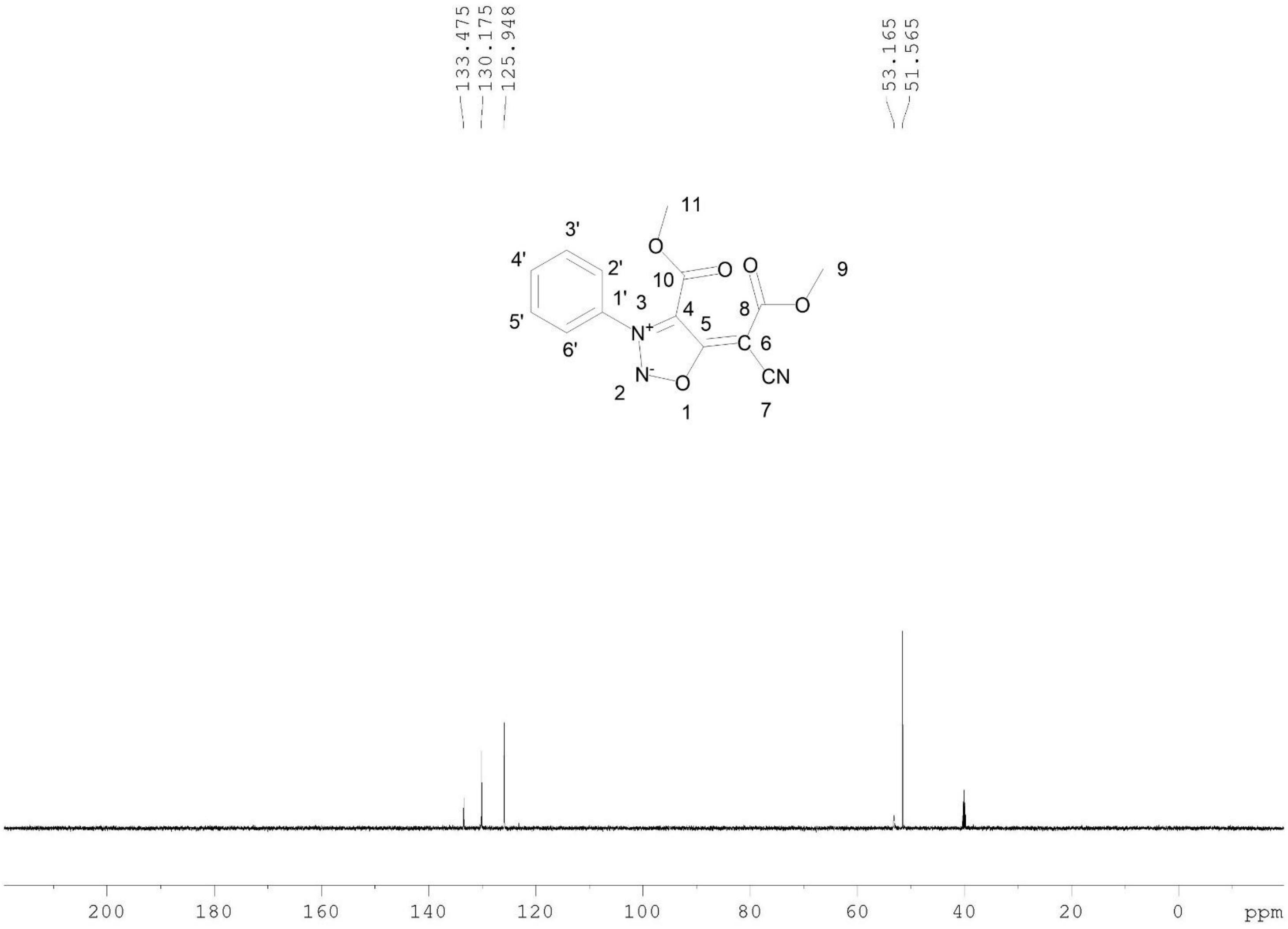

6c ^{1}H-NMR (600 MHz)

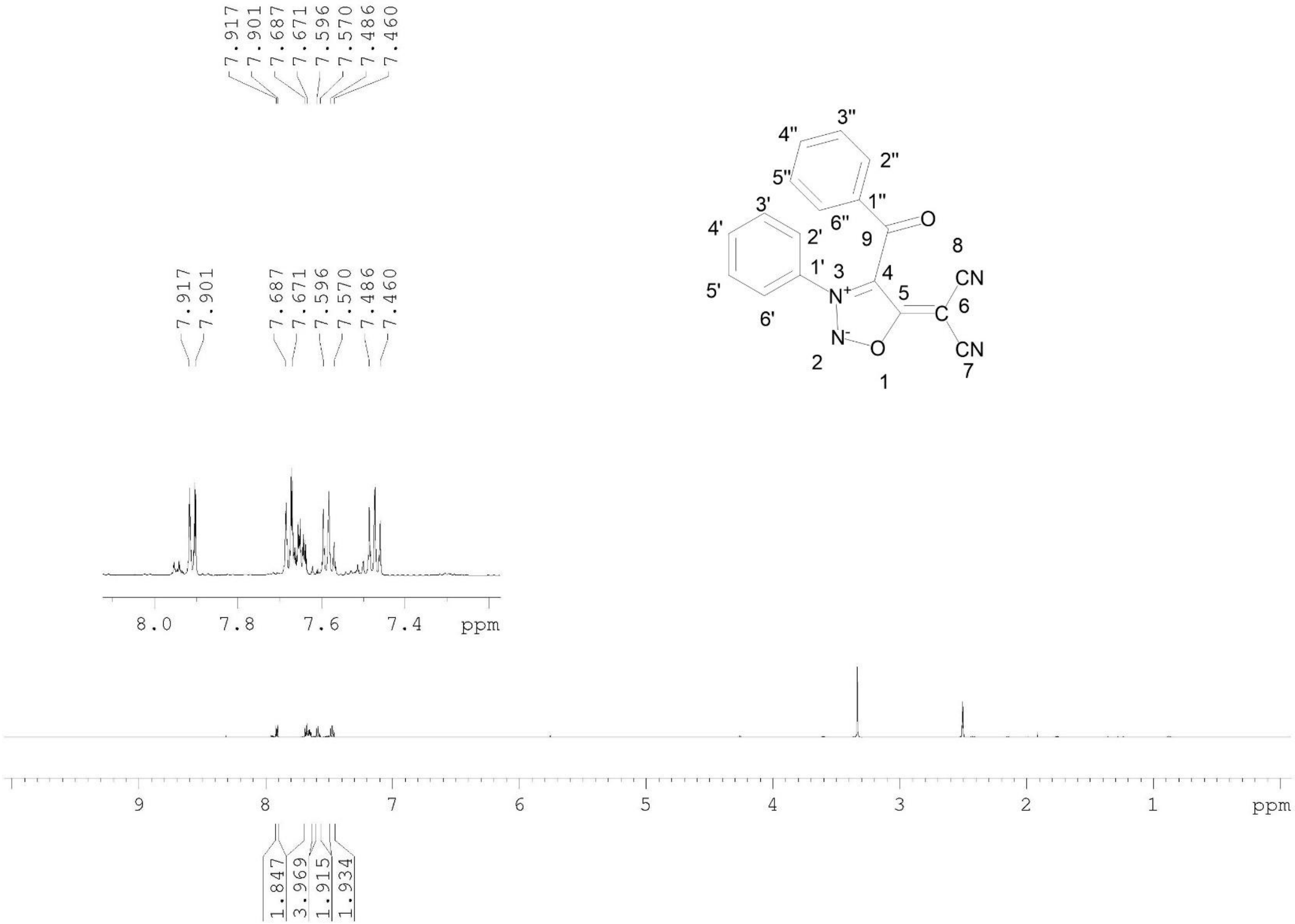

6c ^{13}C-NMR (150 MHz)

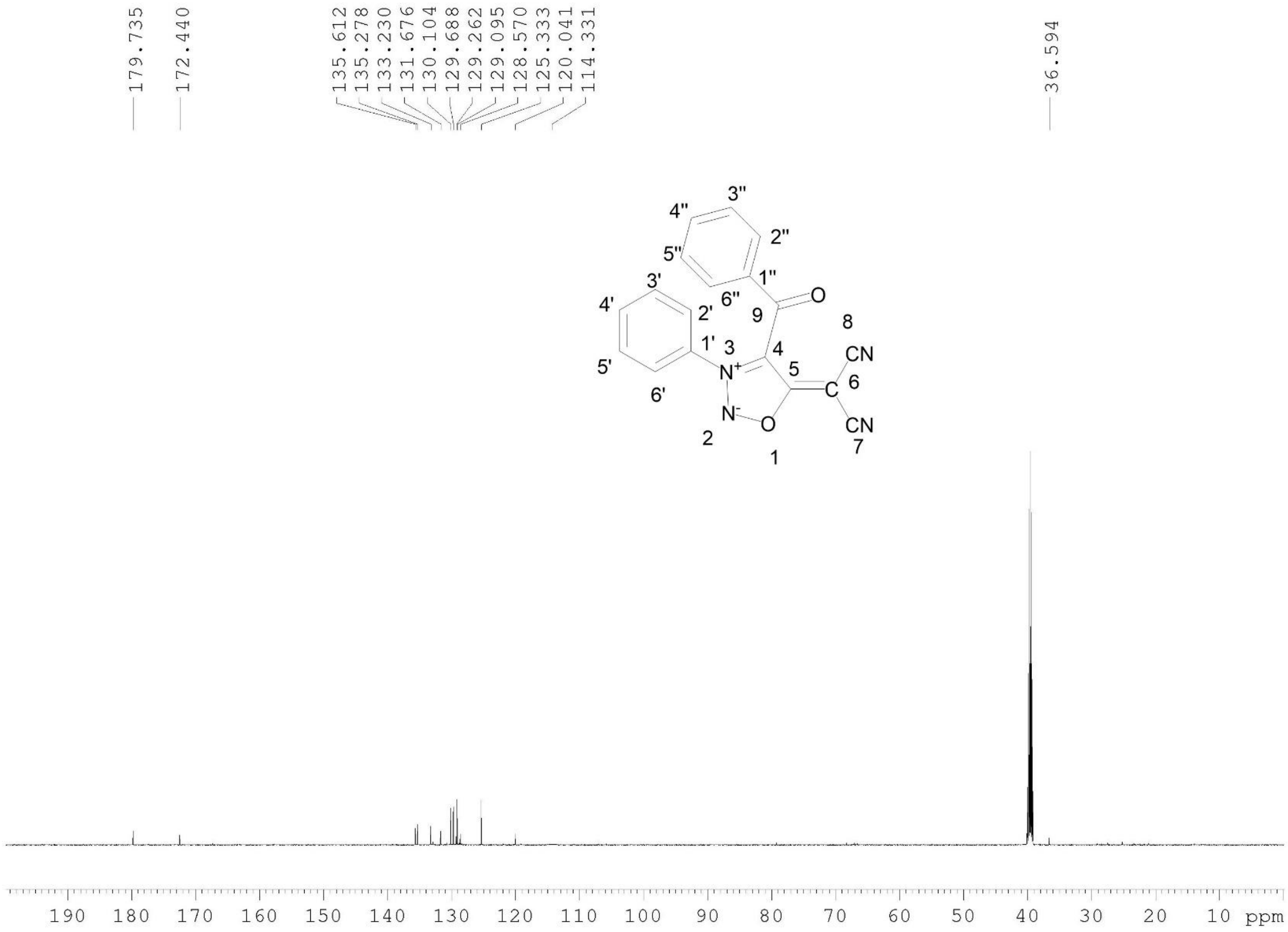

6c ^{13}C-NMR DEPT (150 MHz)

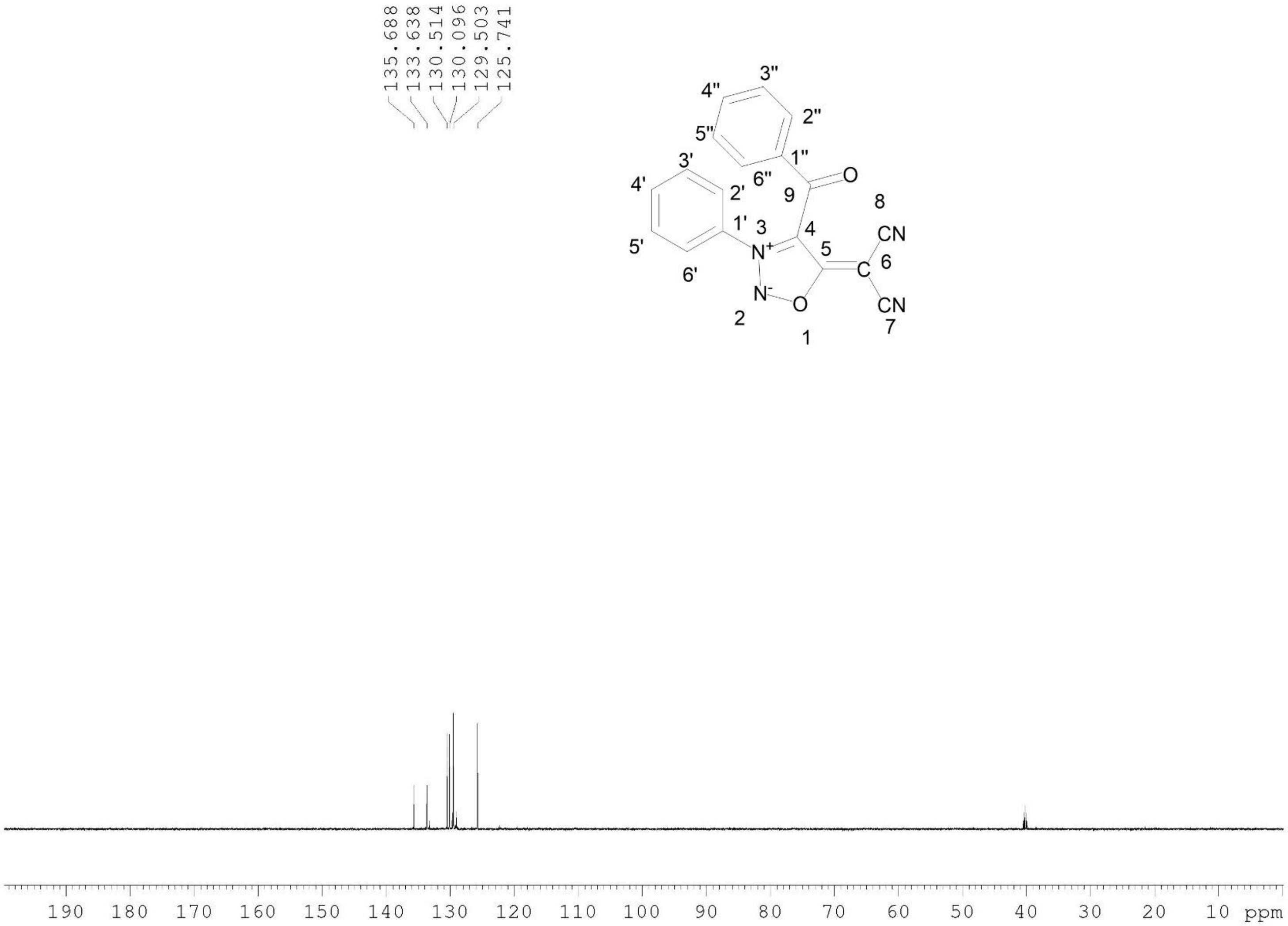

6d ^{1}H-NMR (600 MHz)

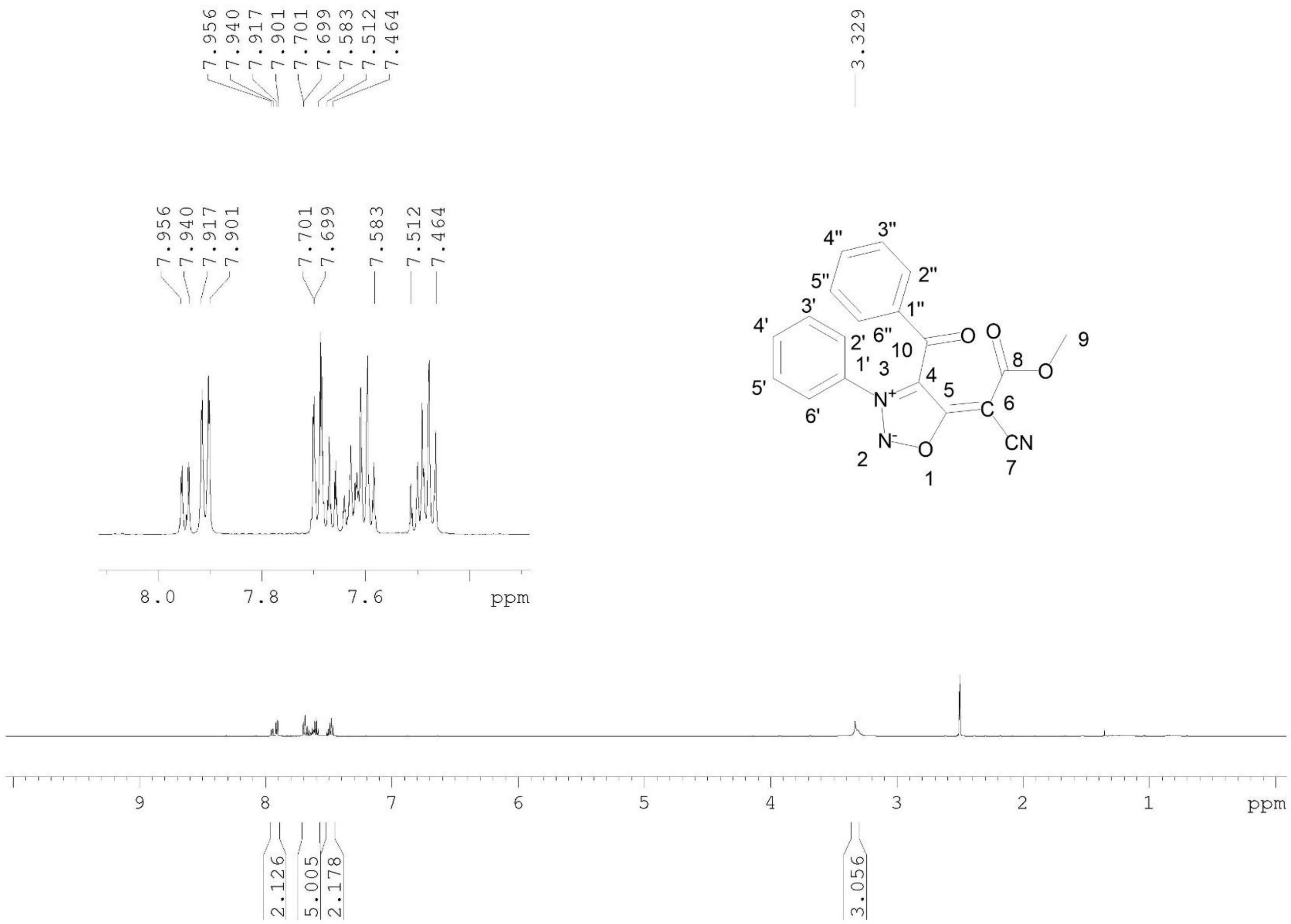

6d ^{13}C-NMR (150 MHz)

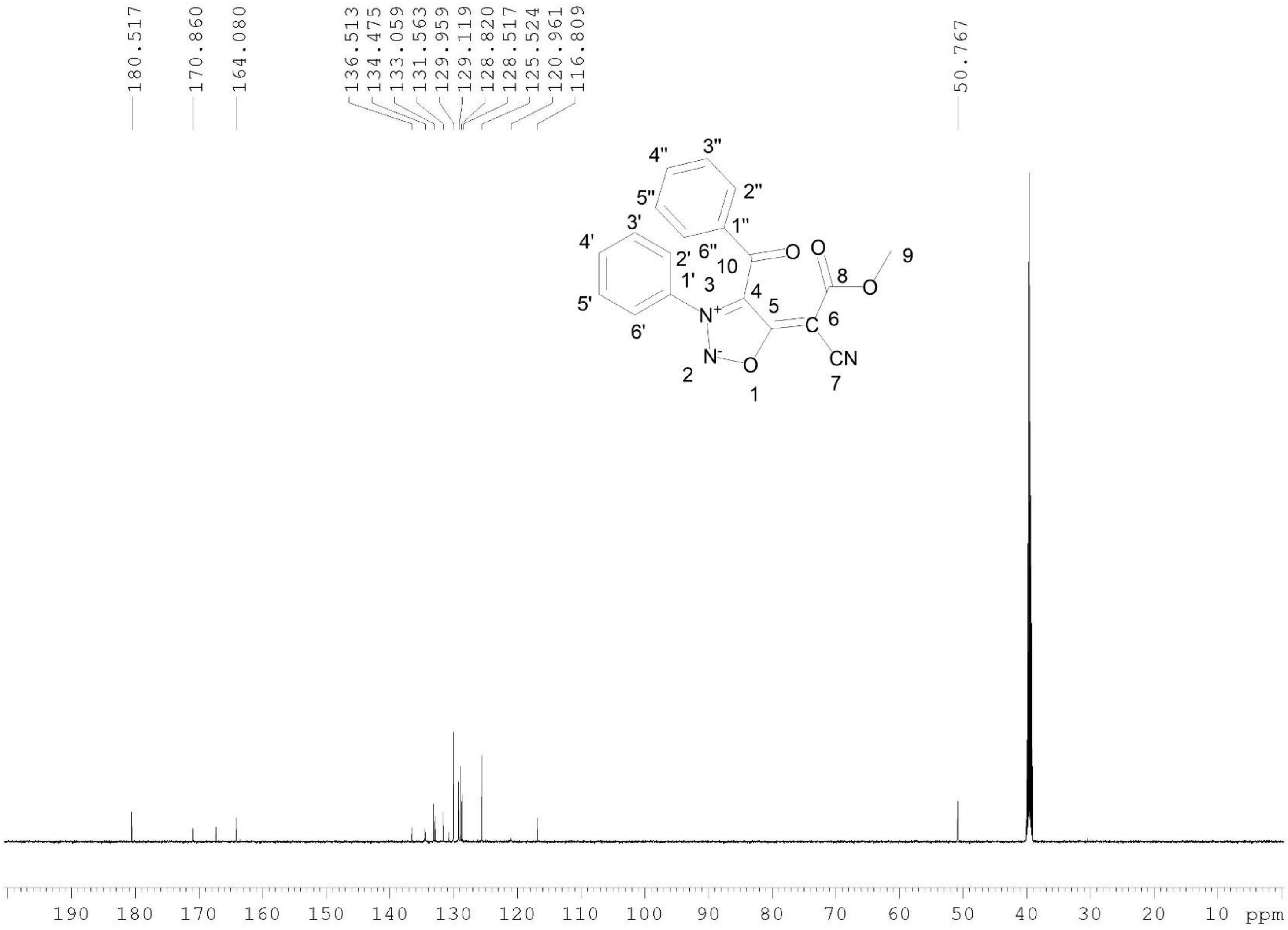

6d ^{13}C-NMR DEPT (150 MHz)

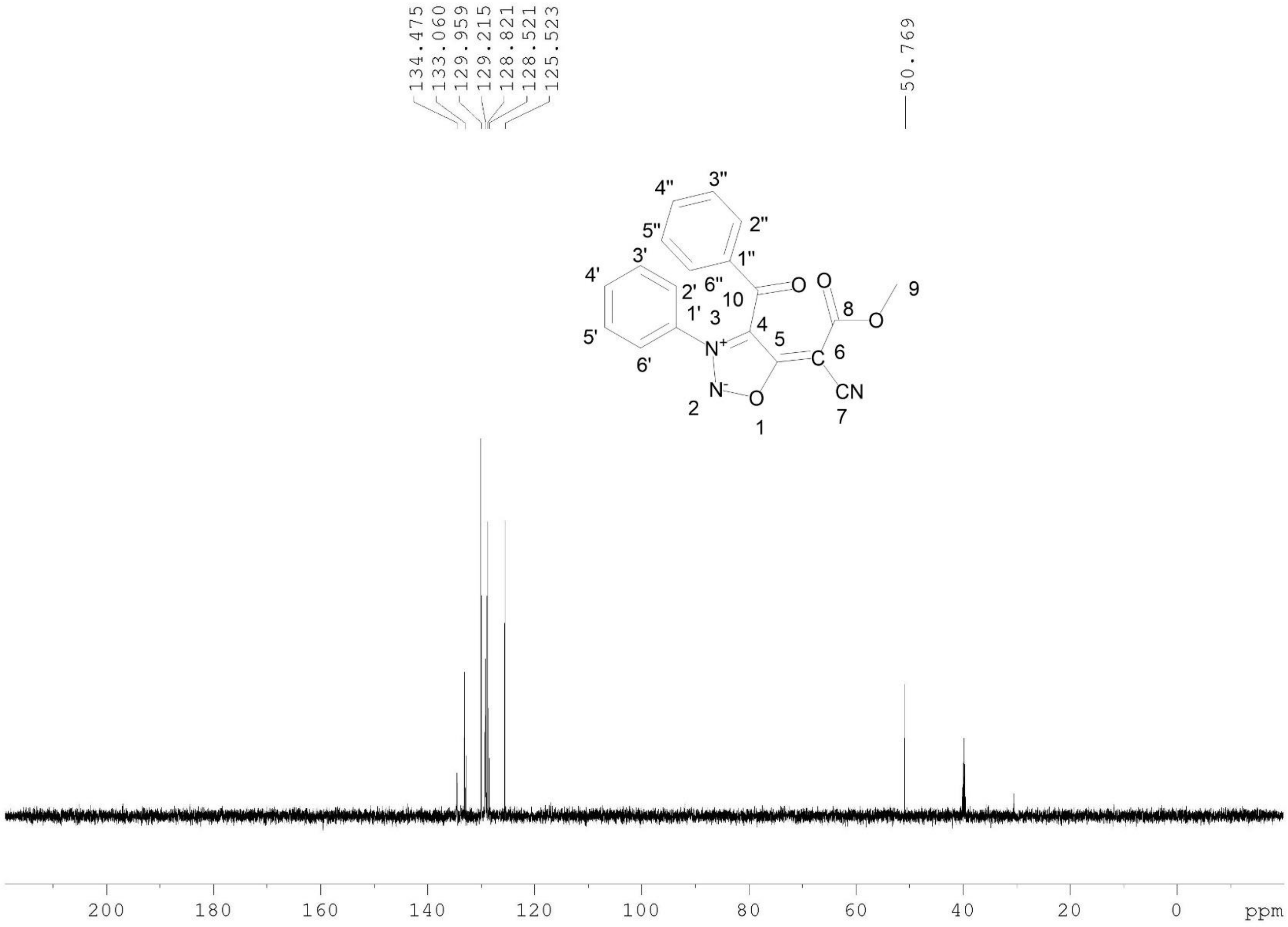

6e ^{1}H-NMR (600 MHz)

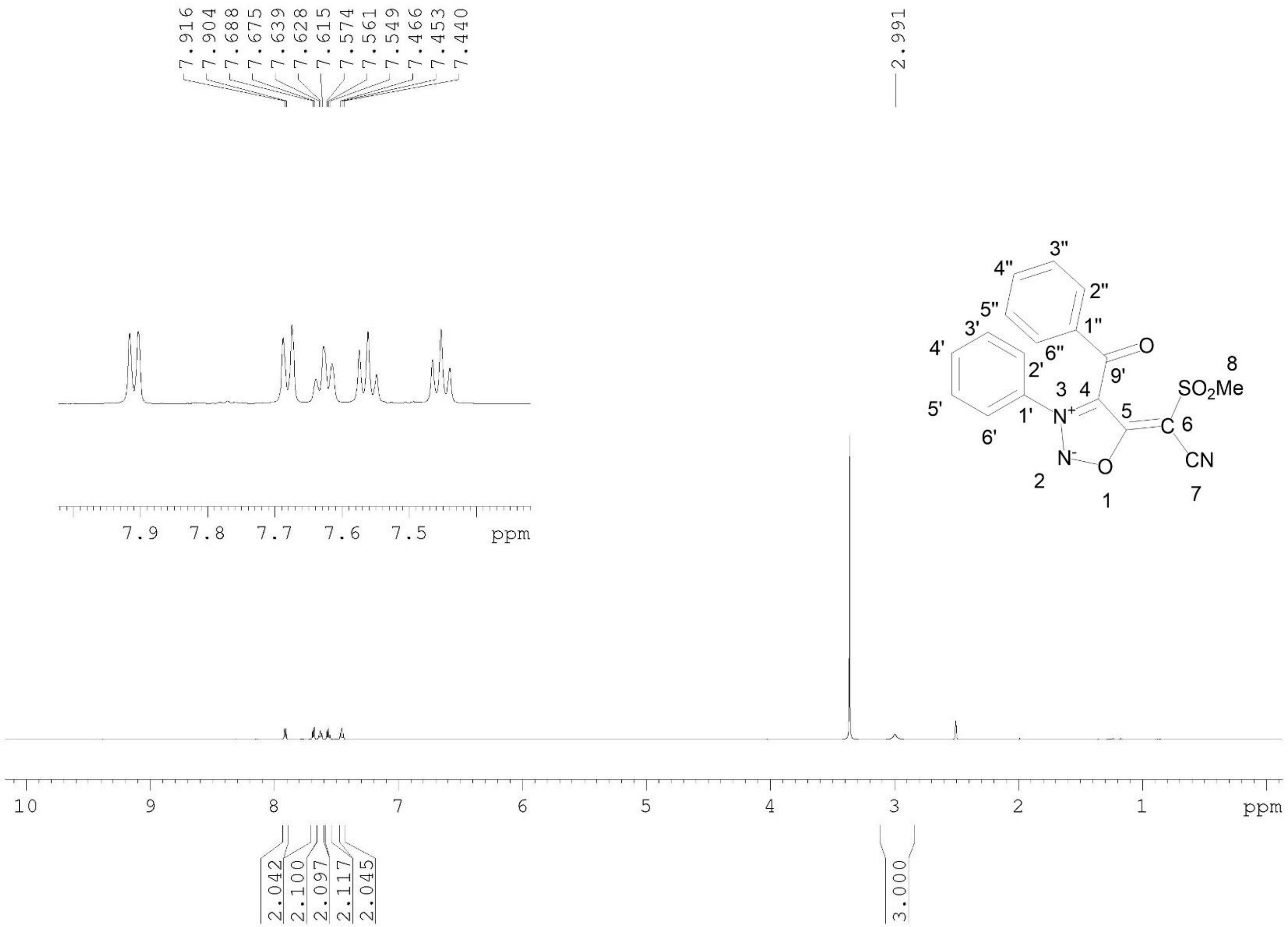

6e ^{13}C-NMR (150 MHz)

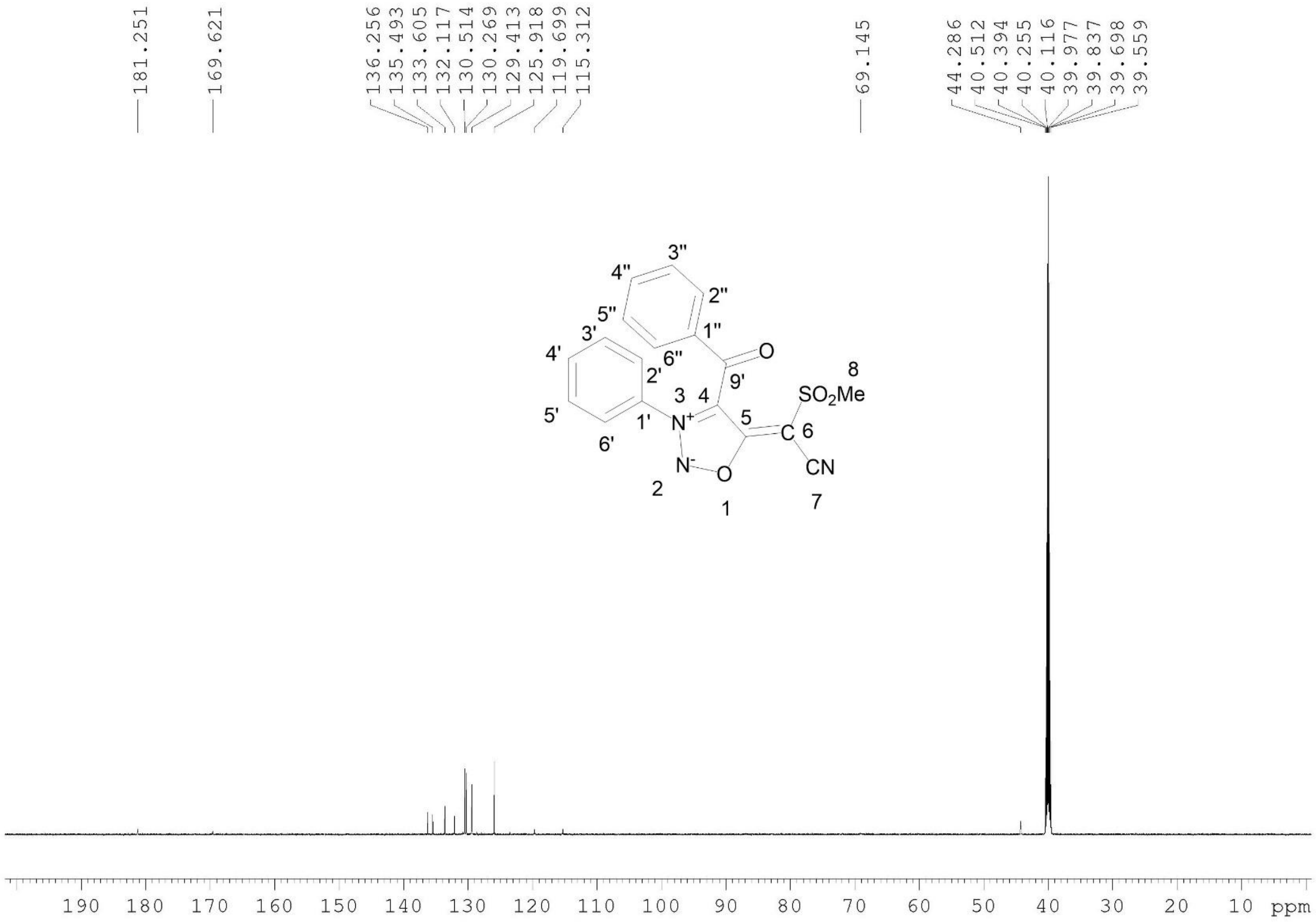

6e ^{13}C-NMR DEPT (150 MHz)

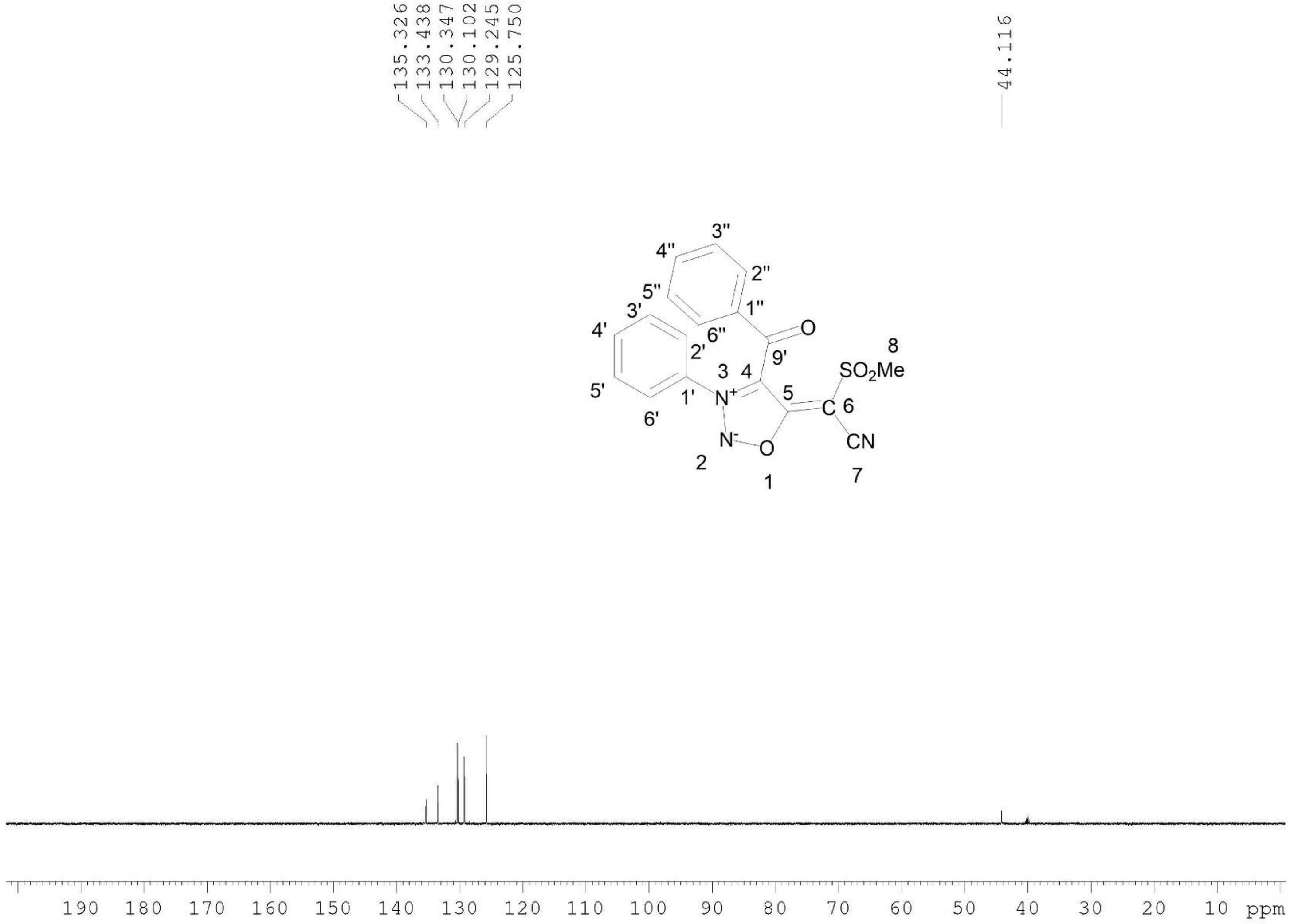

7a ^{1}H-NMR (600 MHz)

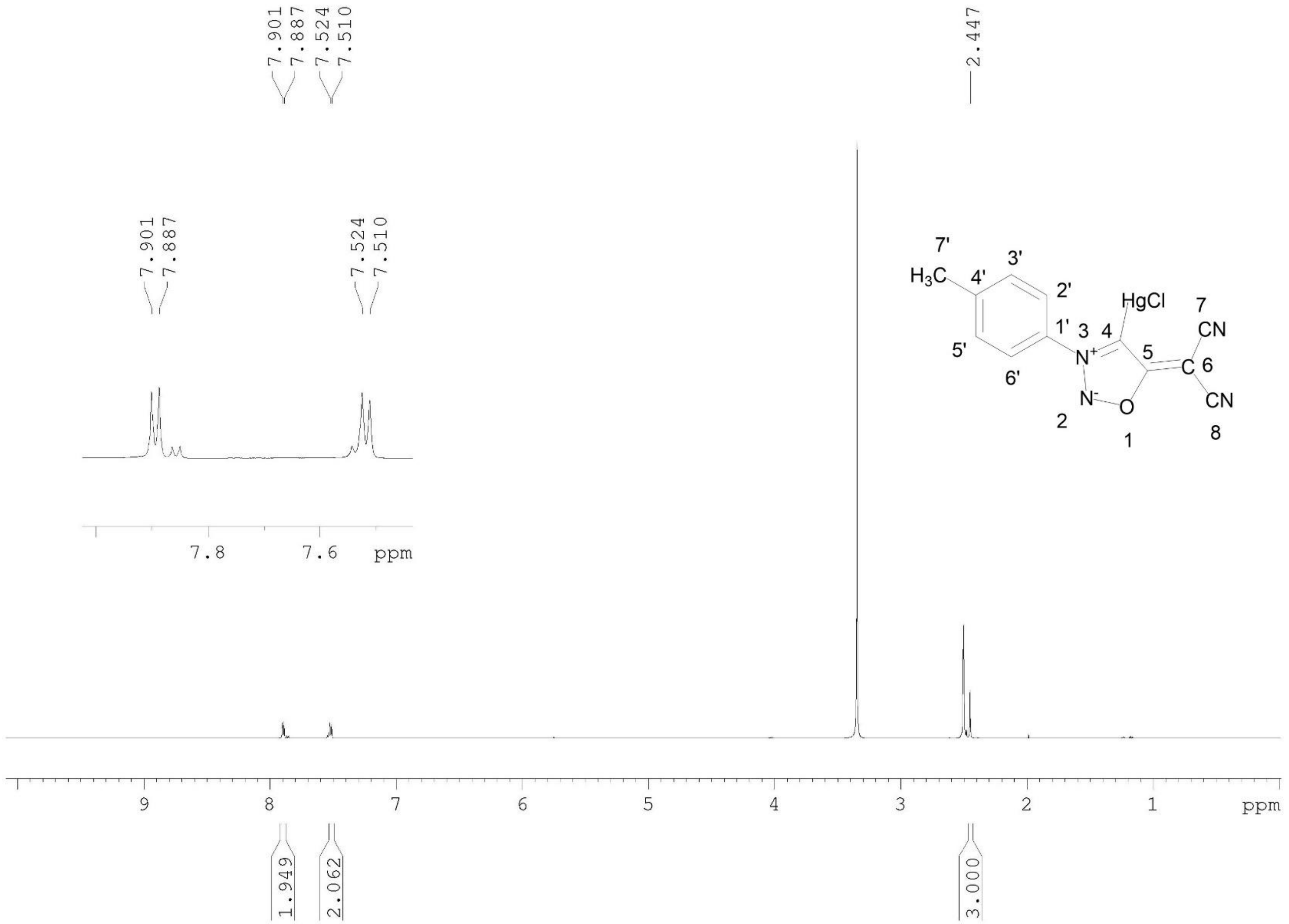

7a ^{13}C-NMR (150 MHz)

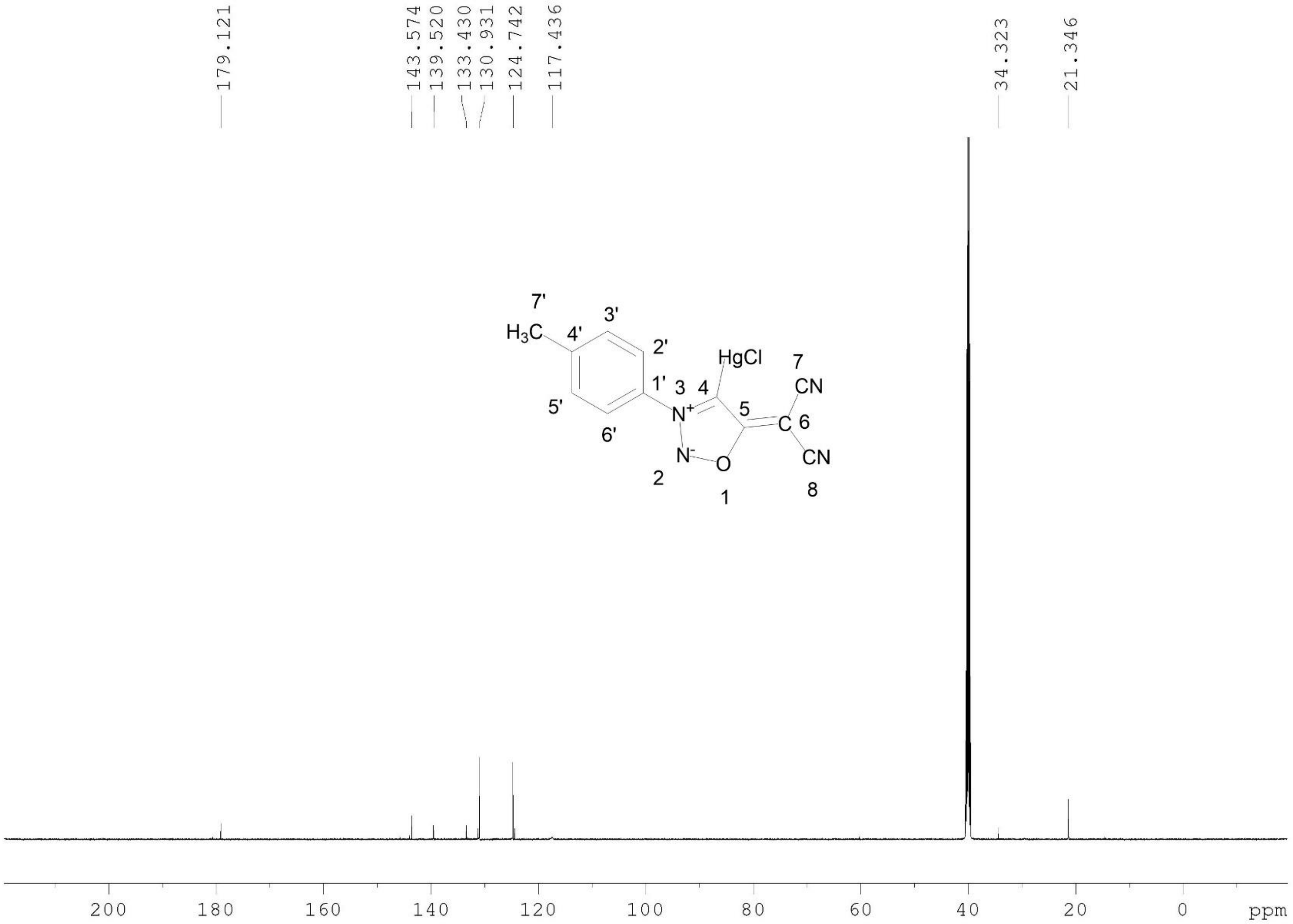

7a ^{13}C-NMR DEPT (150 MHz)

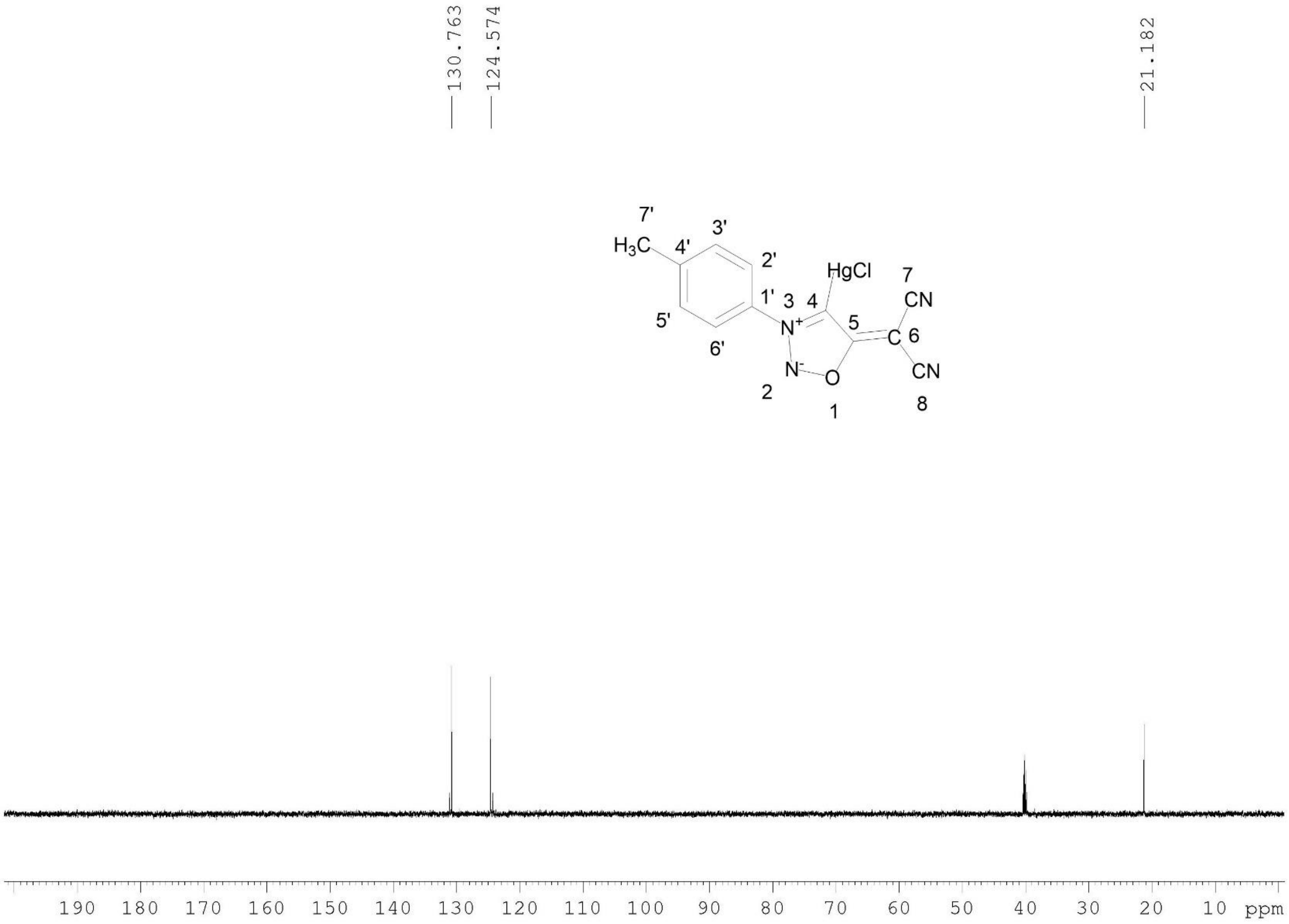

7b ^{1}H-NMR (600 MHz)

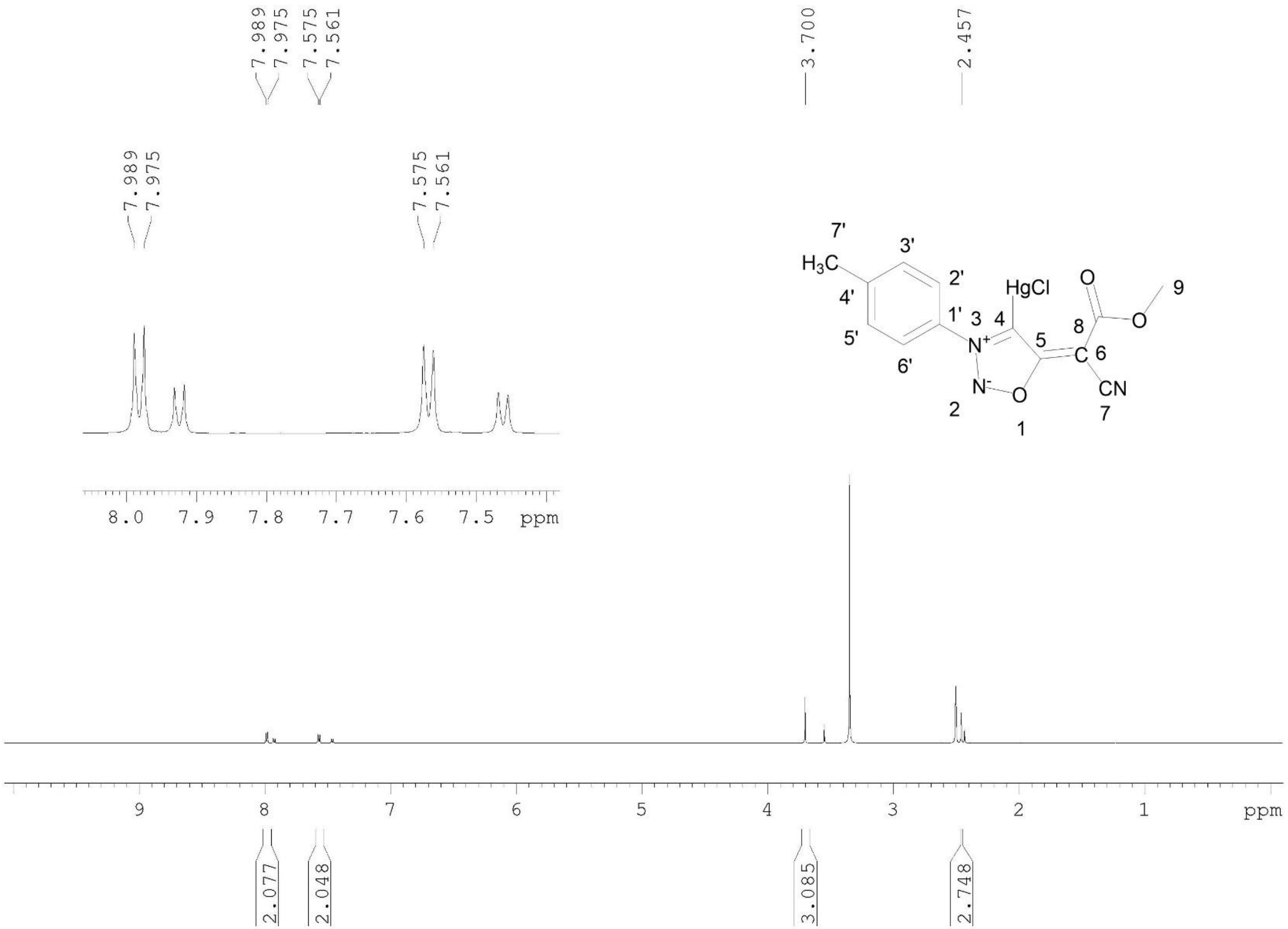

7b ^{13}C-NMR (150 MHz)

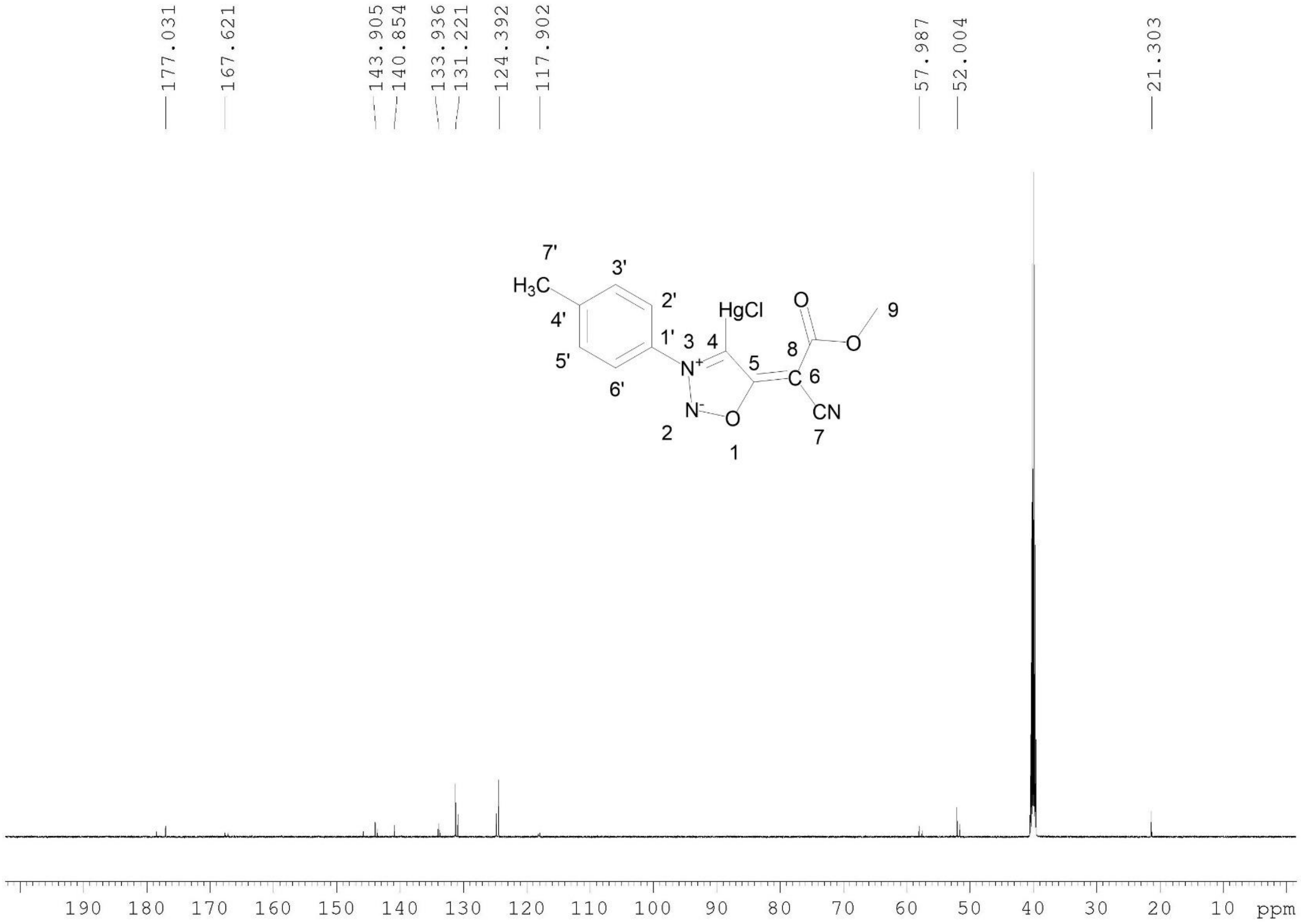

7b ^{13}C-NMR DEPT (150 MHz)

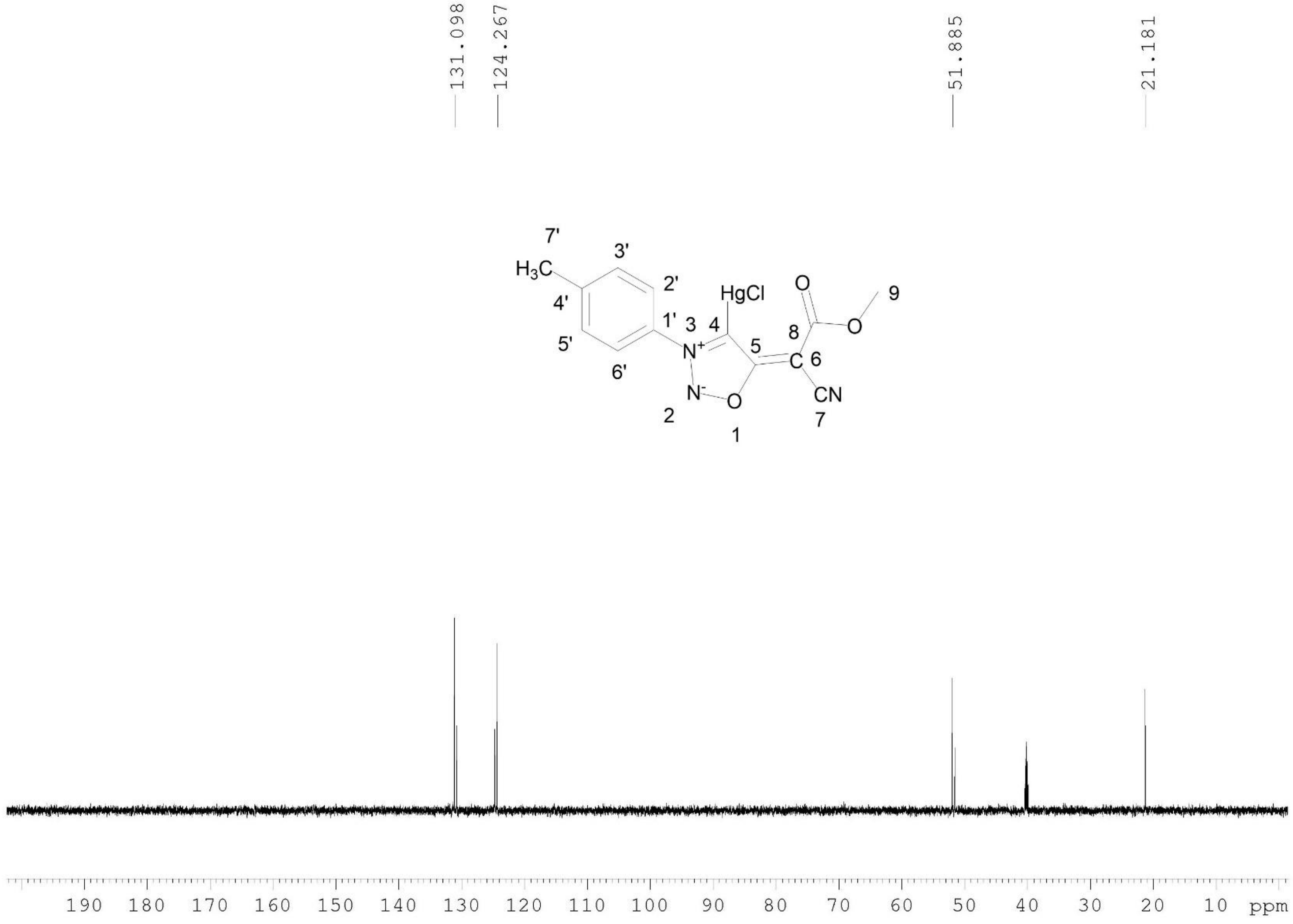

7_2a ^{1}H-NMR (600 MHz)

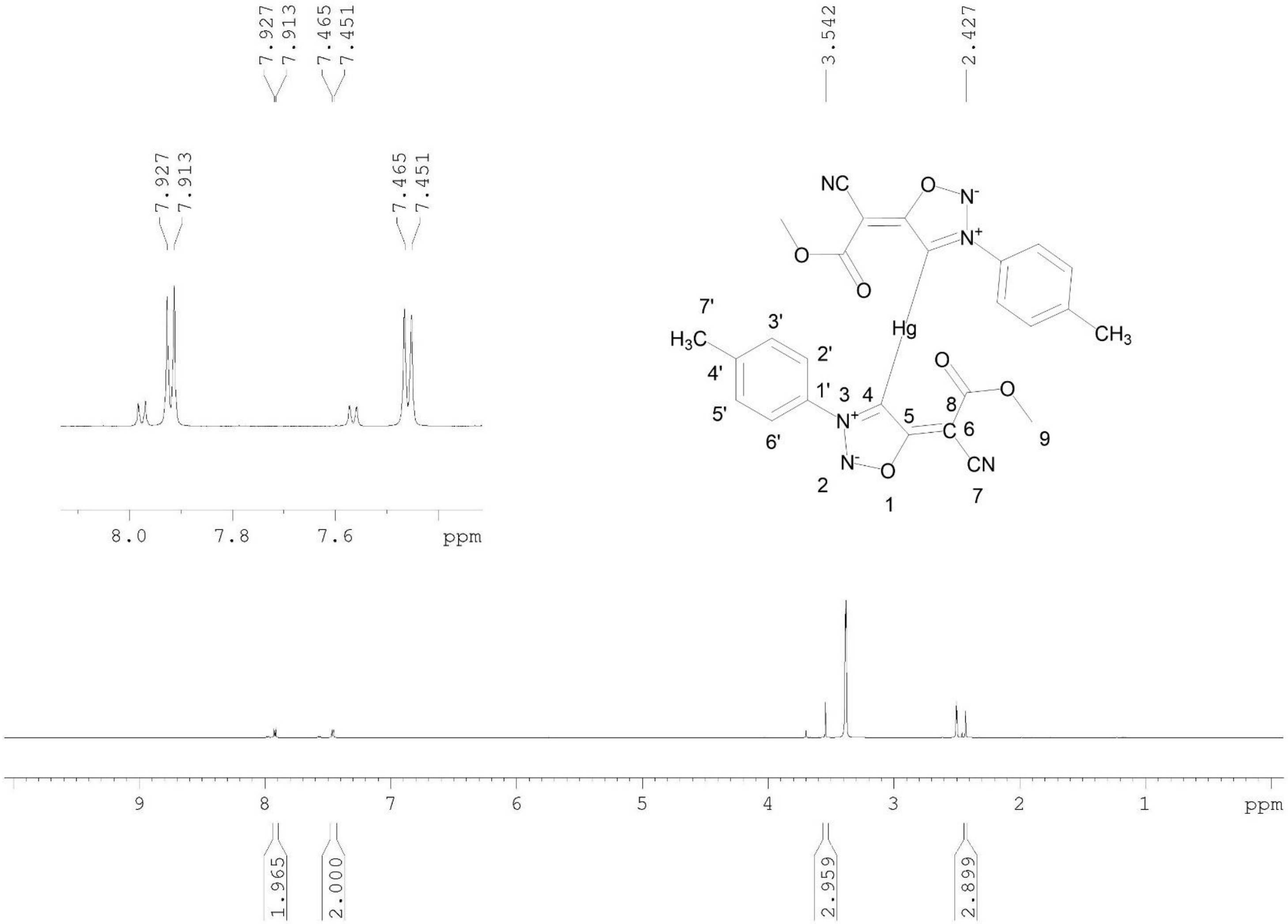

7_2a ^{13}C-NMR (150 MHz)

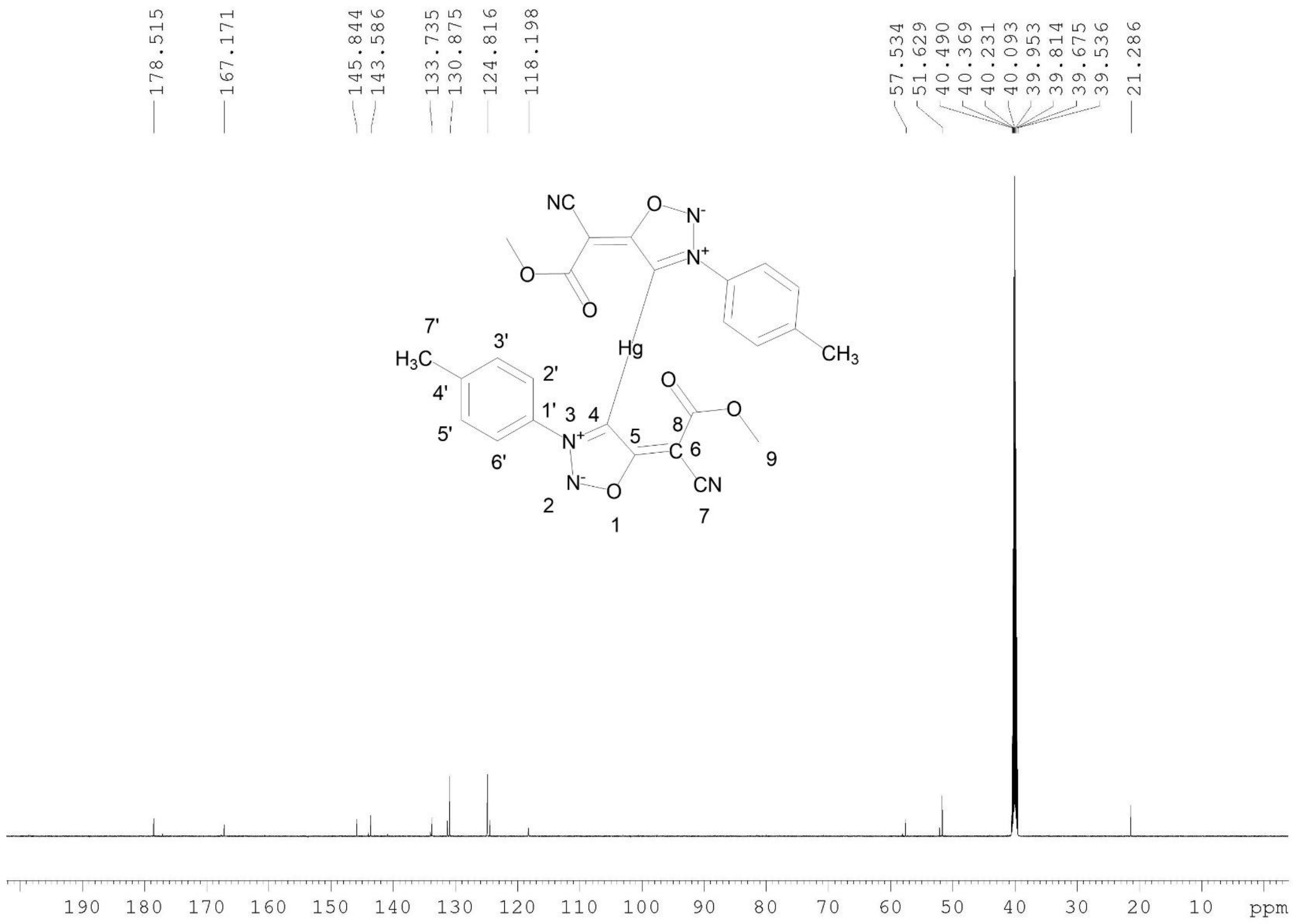

7_2a ^{13}C-NMR DEPT (150 MHz)

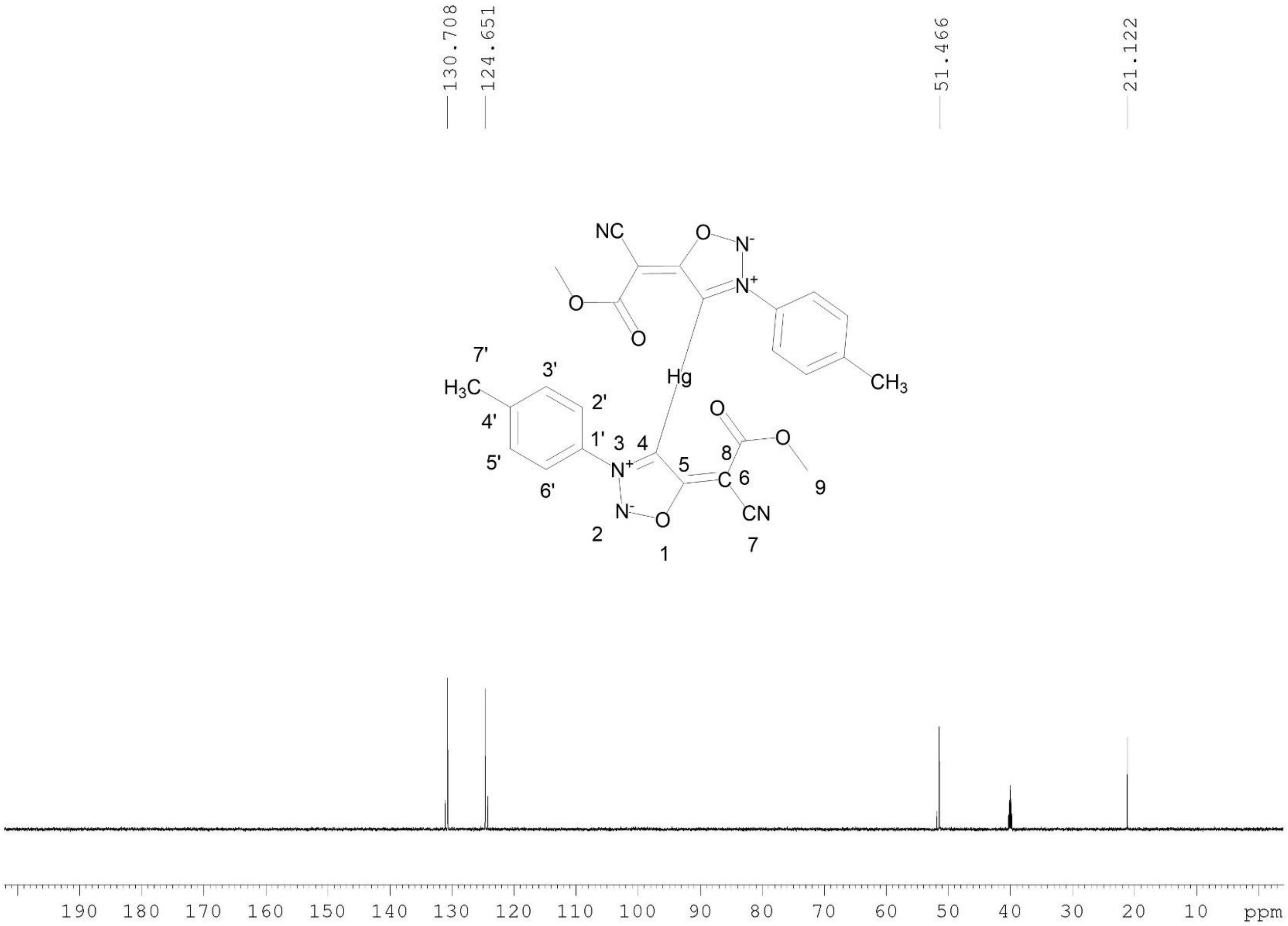

7_2b ^{1}H-NMR (600 MHz)

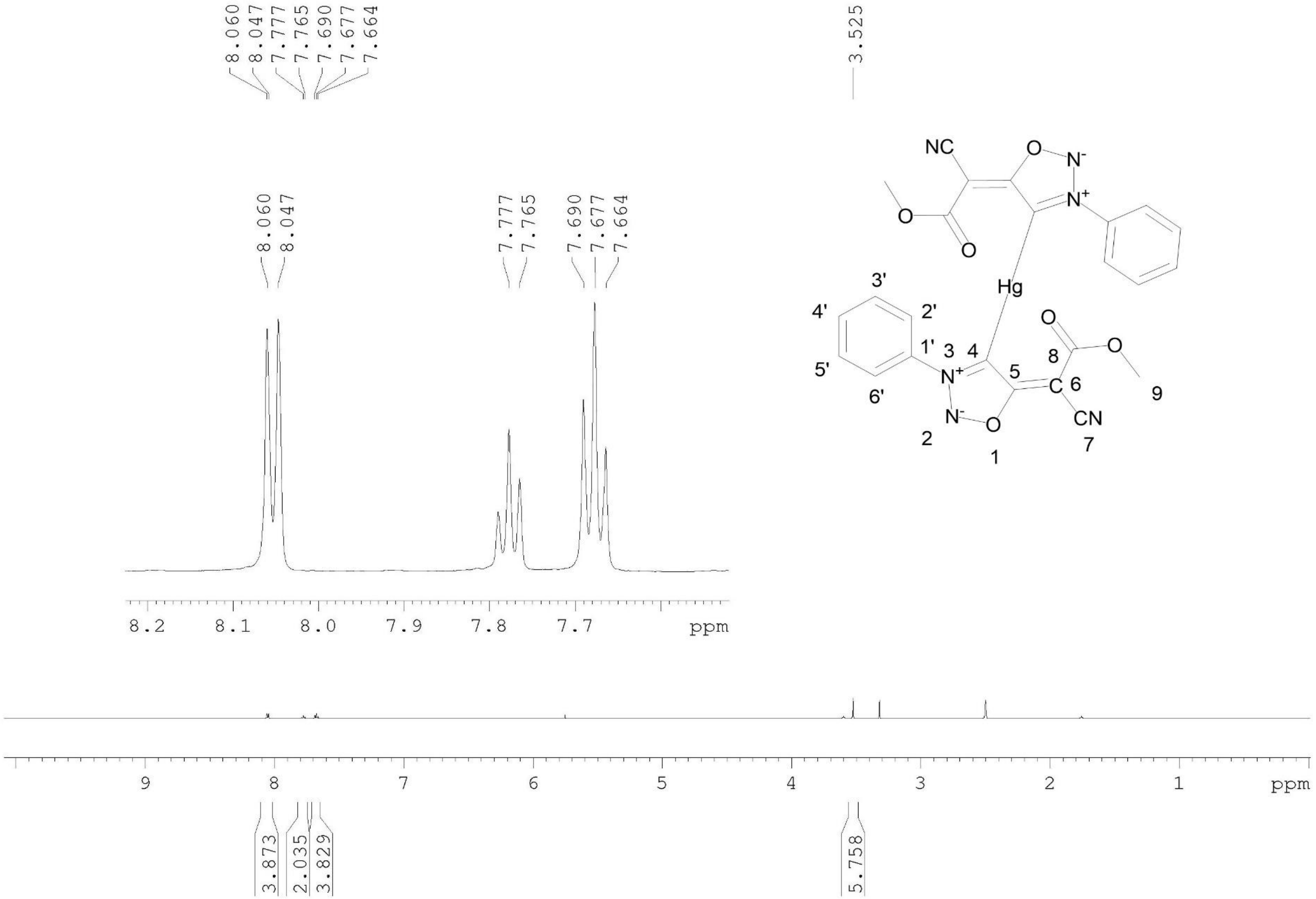

7_2b ^{13}C-NMR (150 MHz)

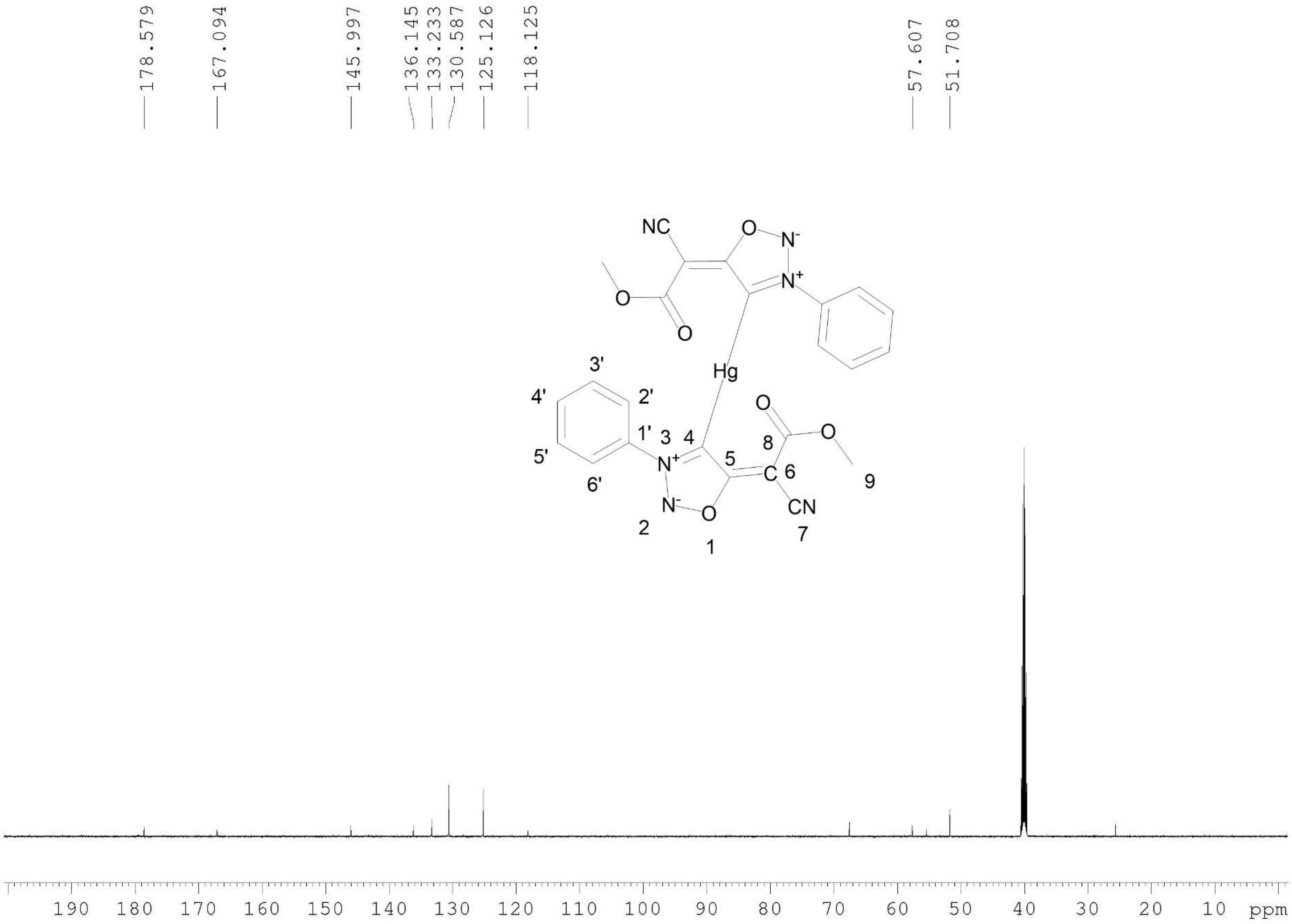

7_2b ^{13}C-NMR DEPT (150 MHz)

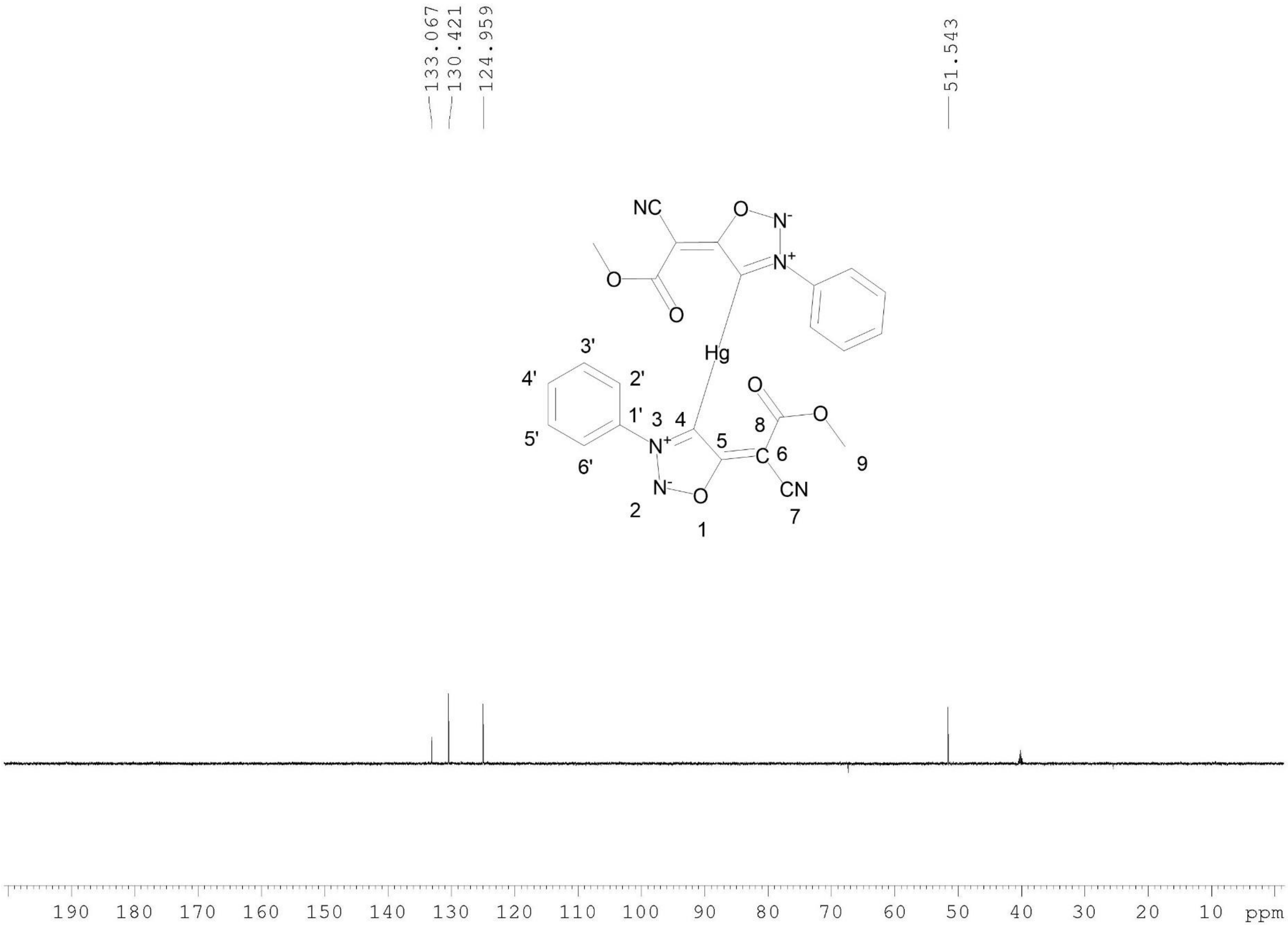

8a ^{1}H-NMR (600 MHz)

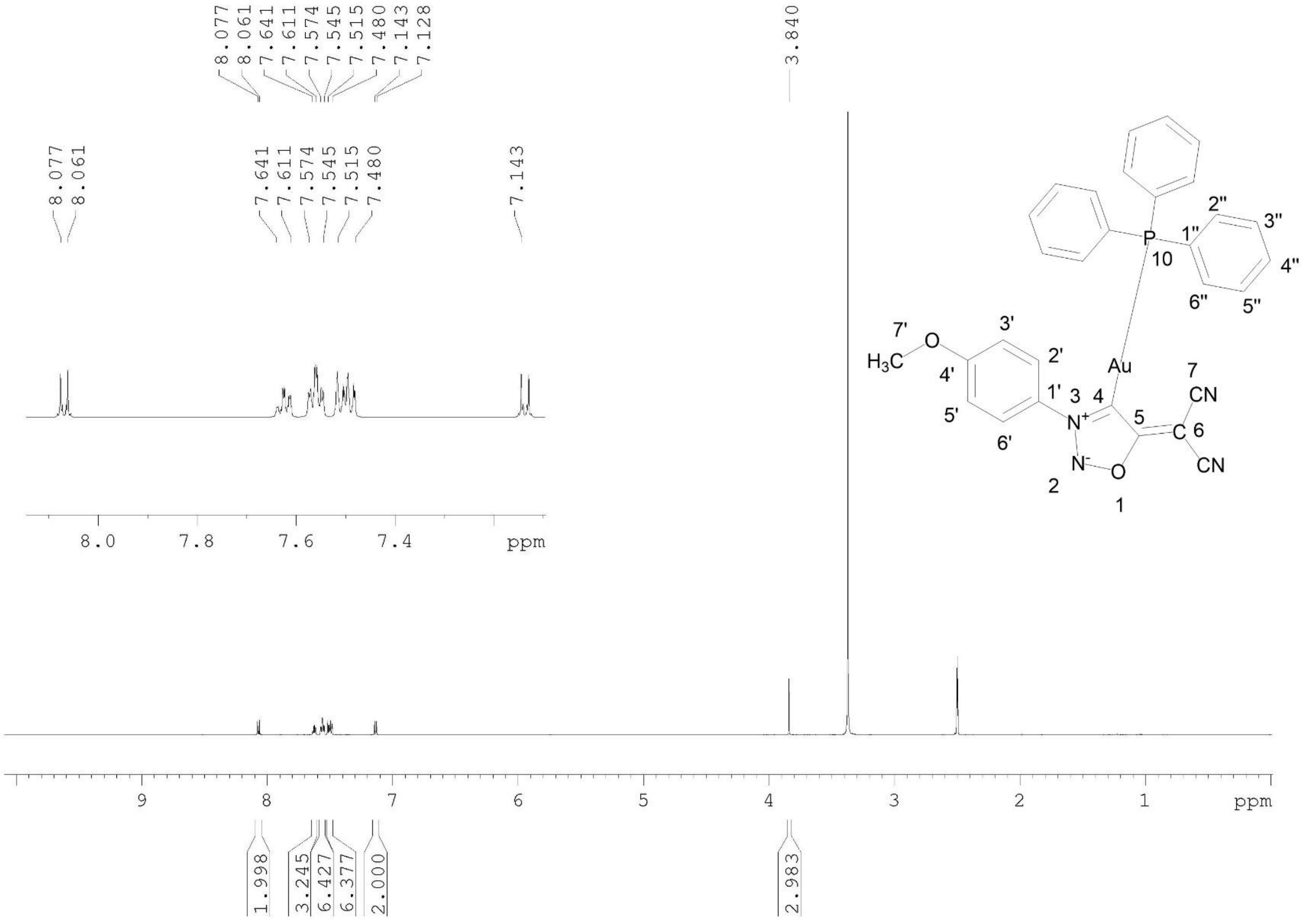

8a ^{13}C-NMR (150 MHz)

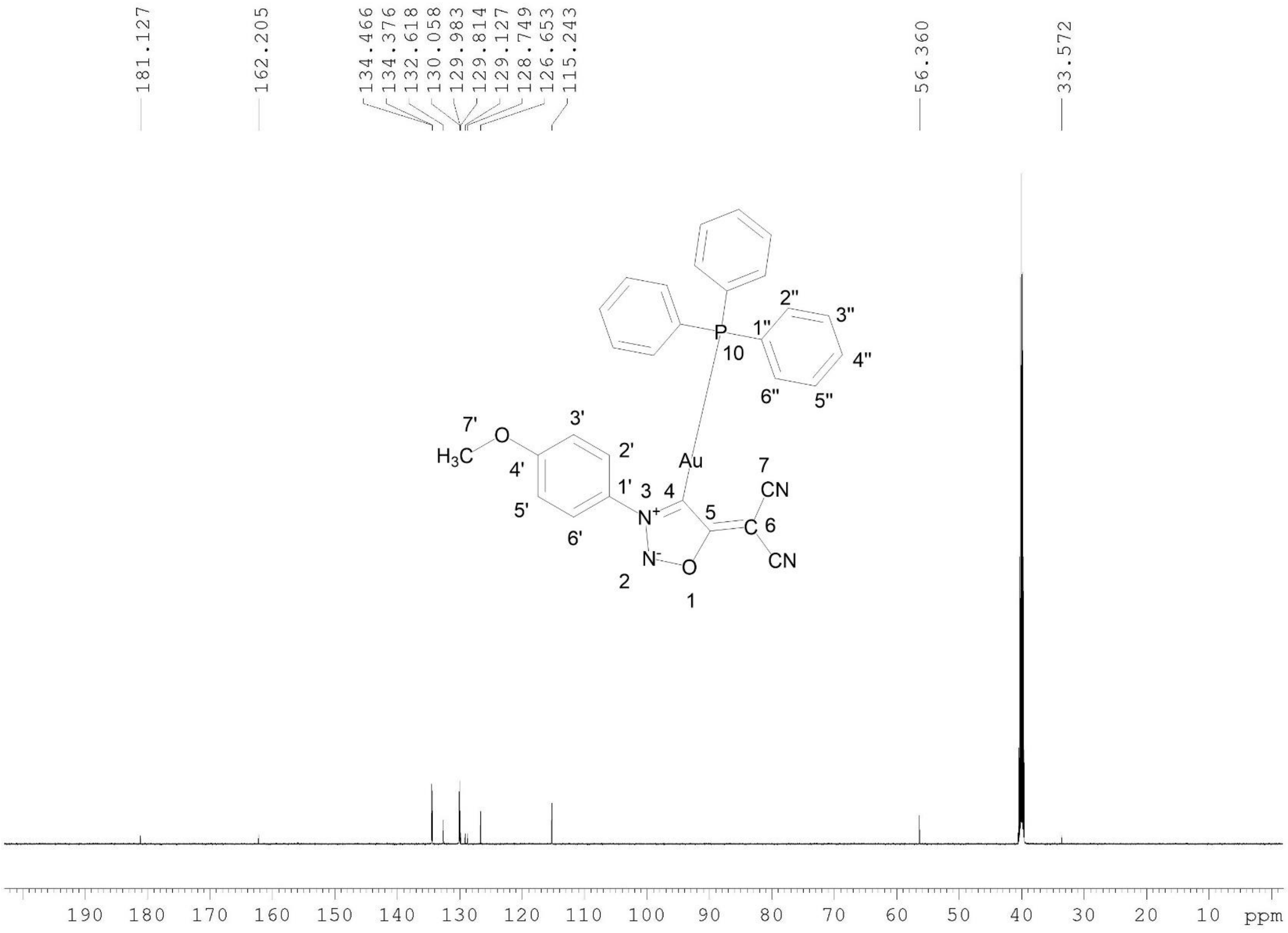

8a ^{13}C-NMR DEPT (150 MHz)

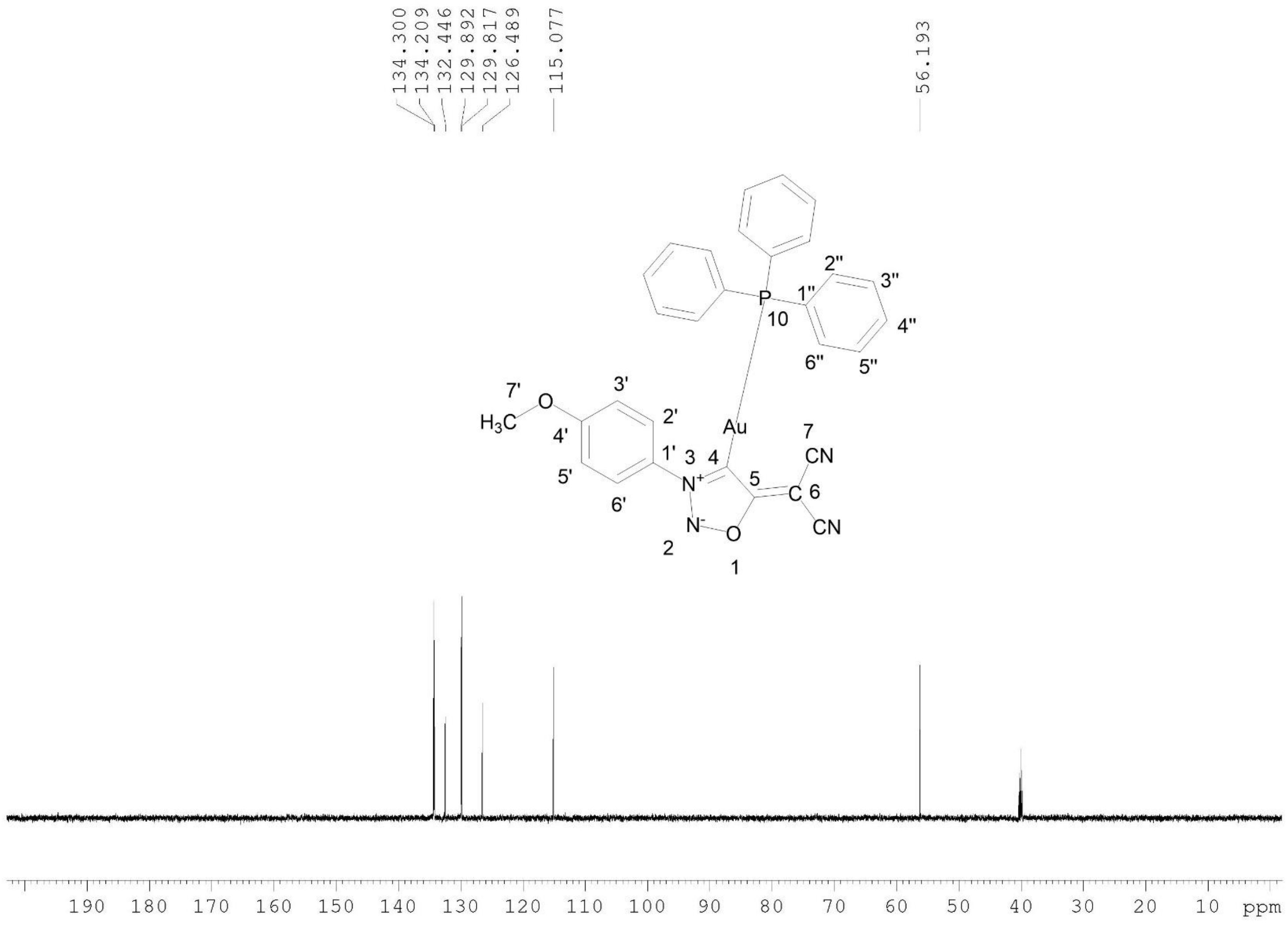

8b ^{1}H-NMR (600 MHz)

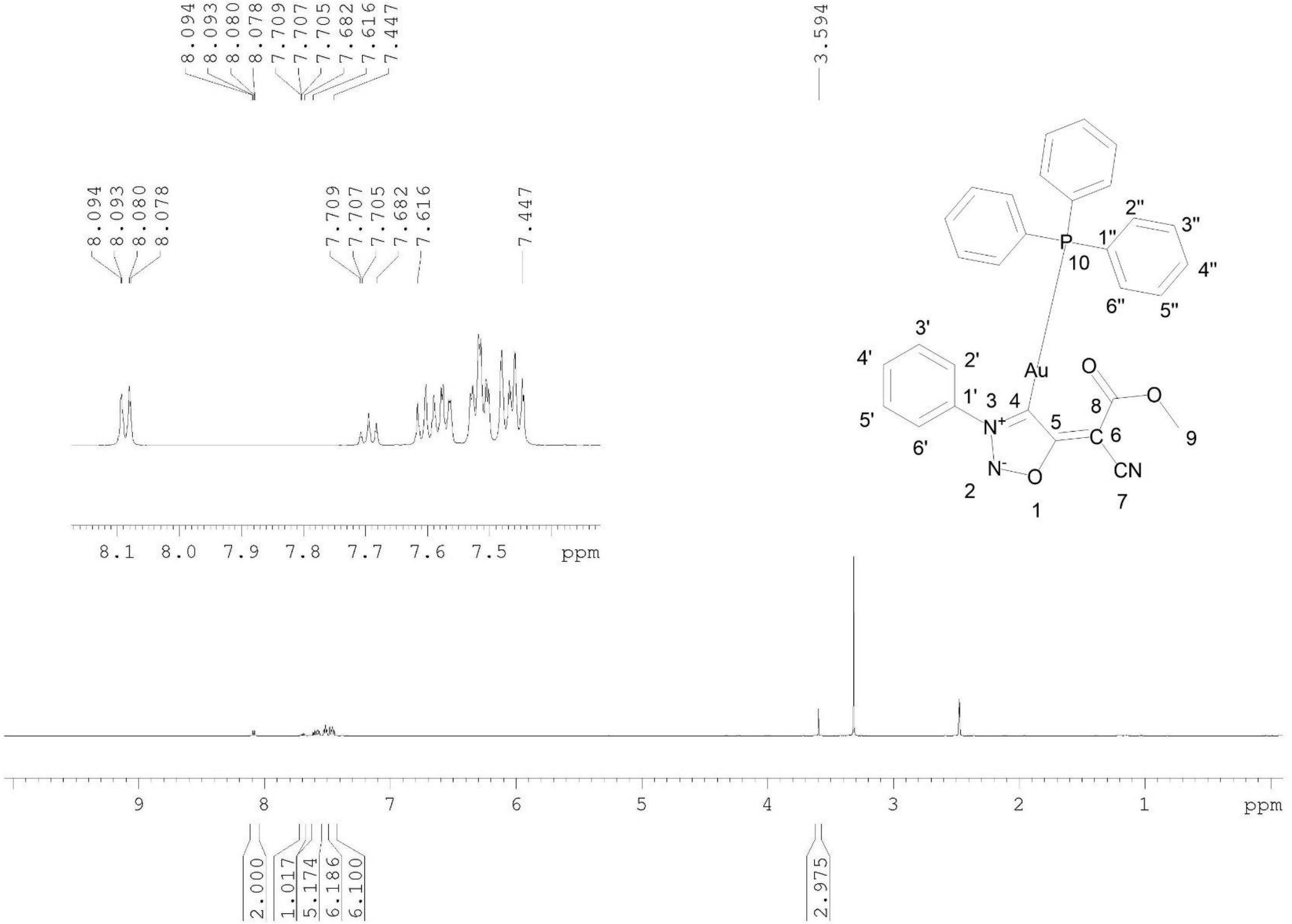

8b ^{13}C-NMR (150 MHz)

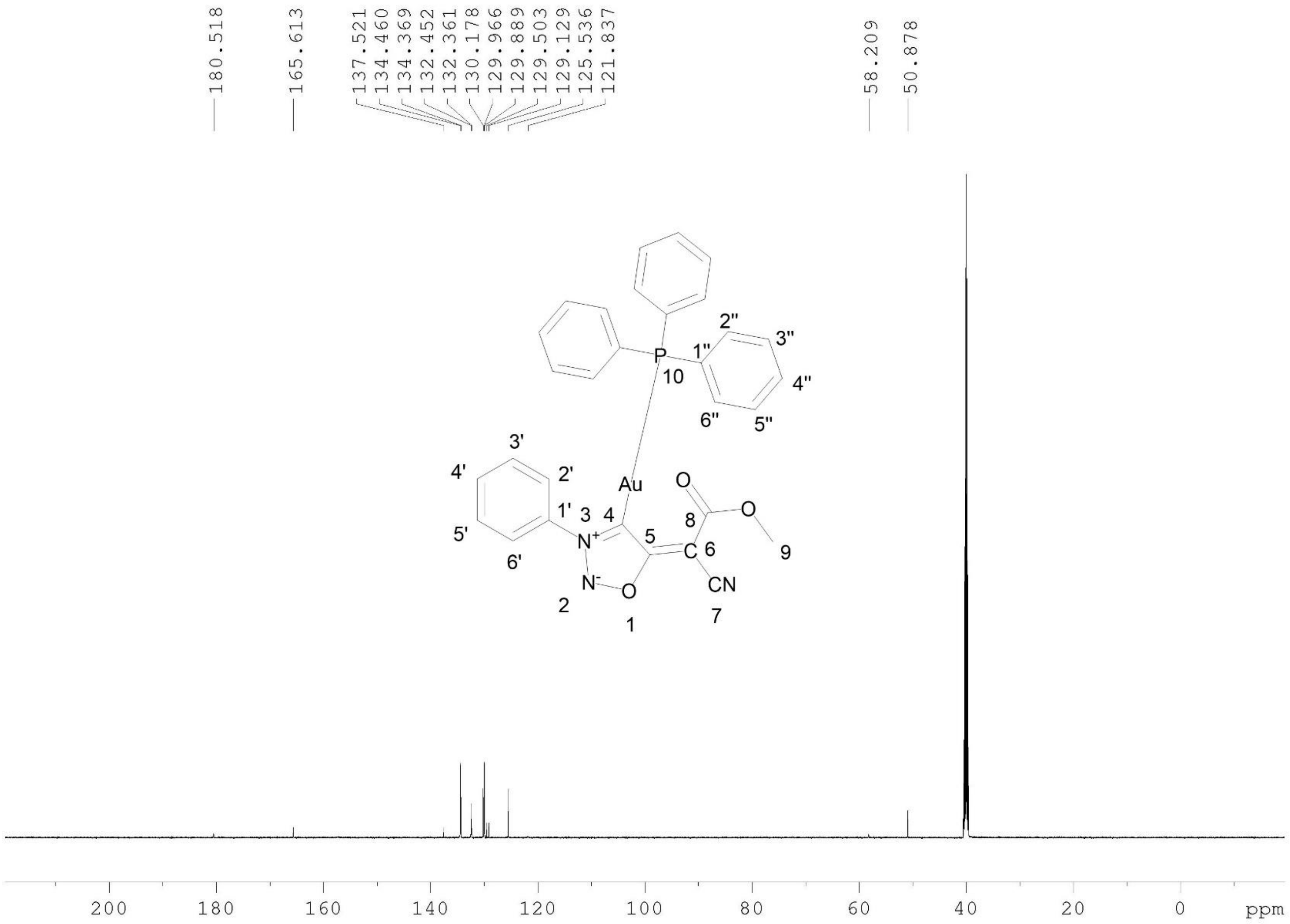

8b ^{13}C-NMR DEPT (150 MHz)

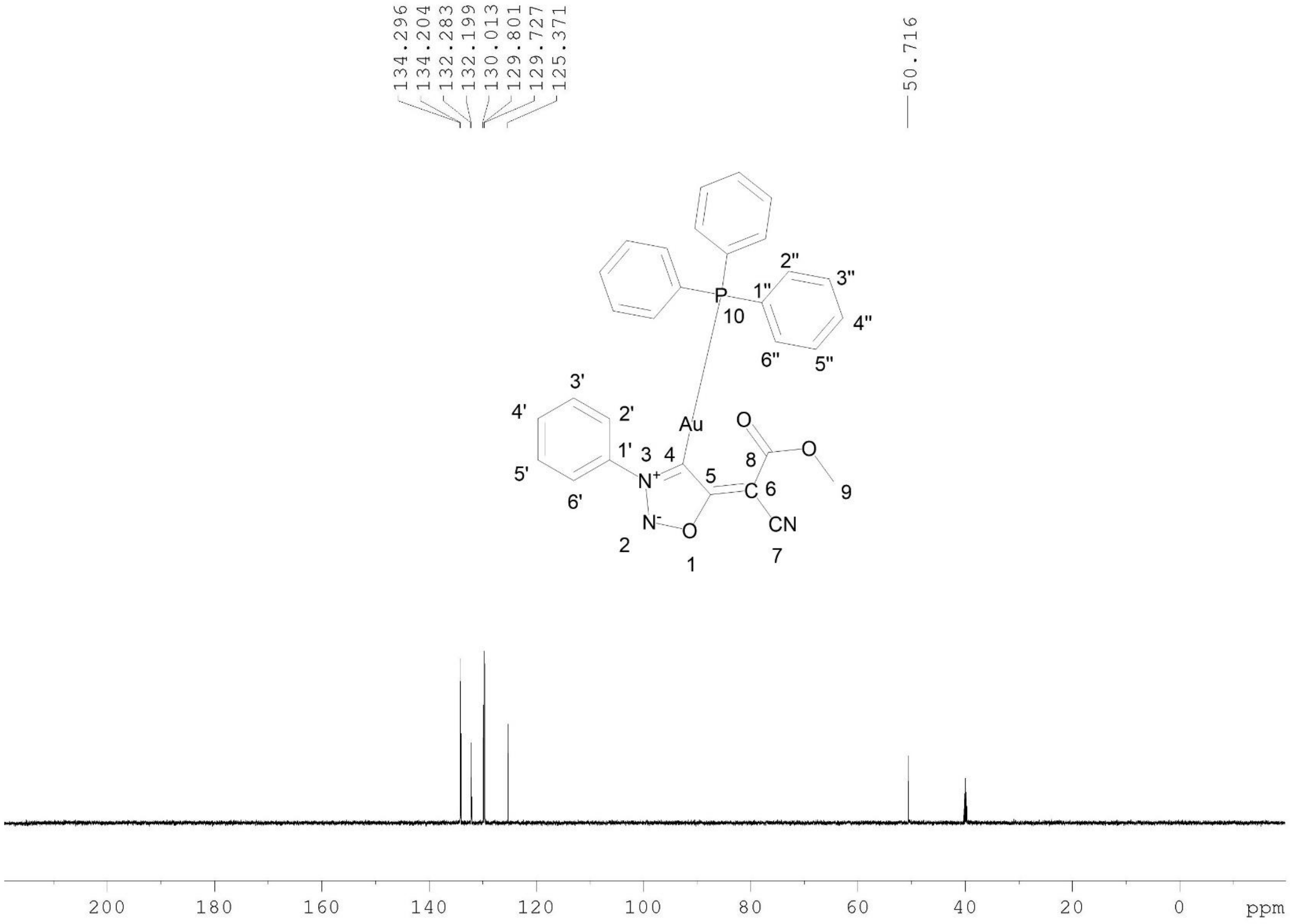

8c ^{1}H-NMR (600 MHz)

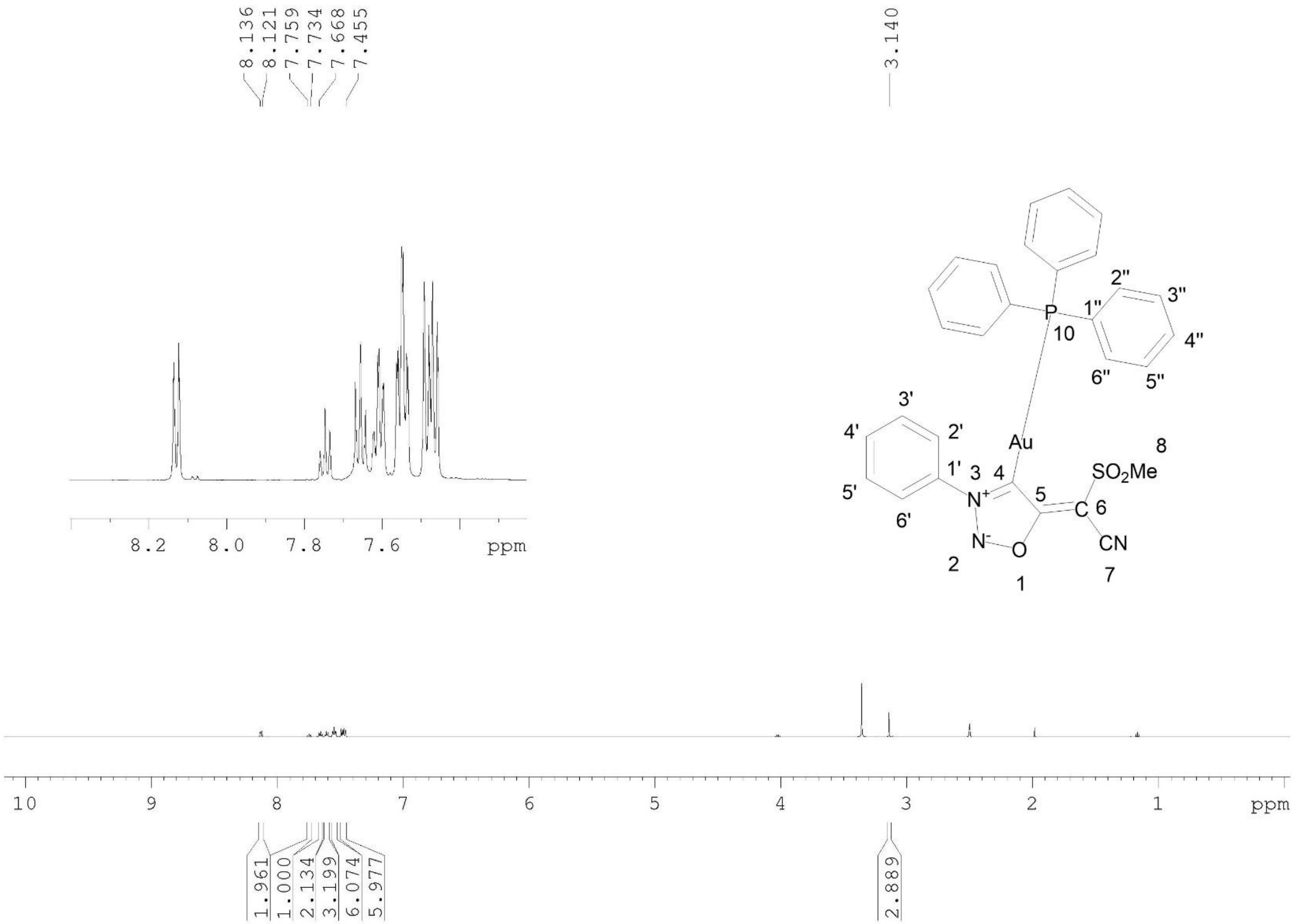

8c ^{13}C-NMR (150 MHz)

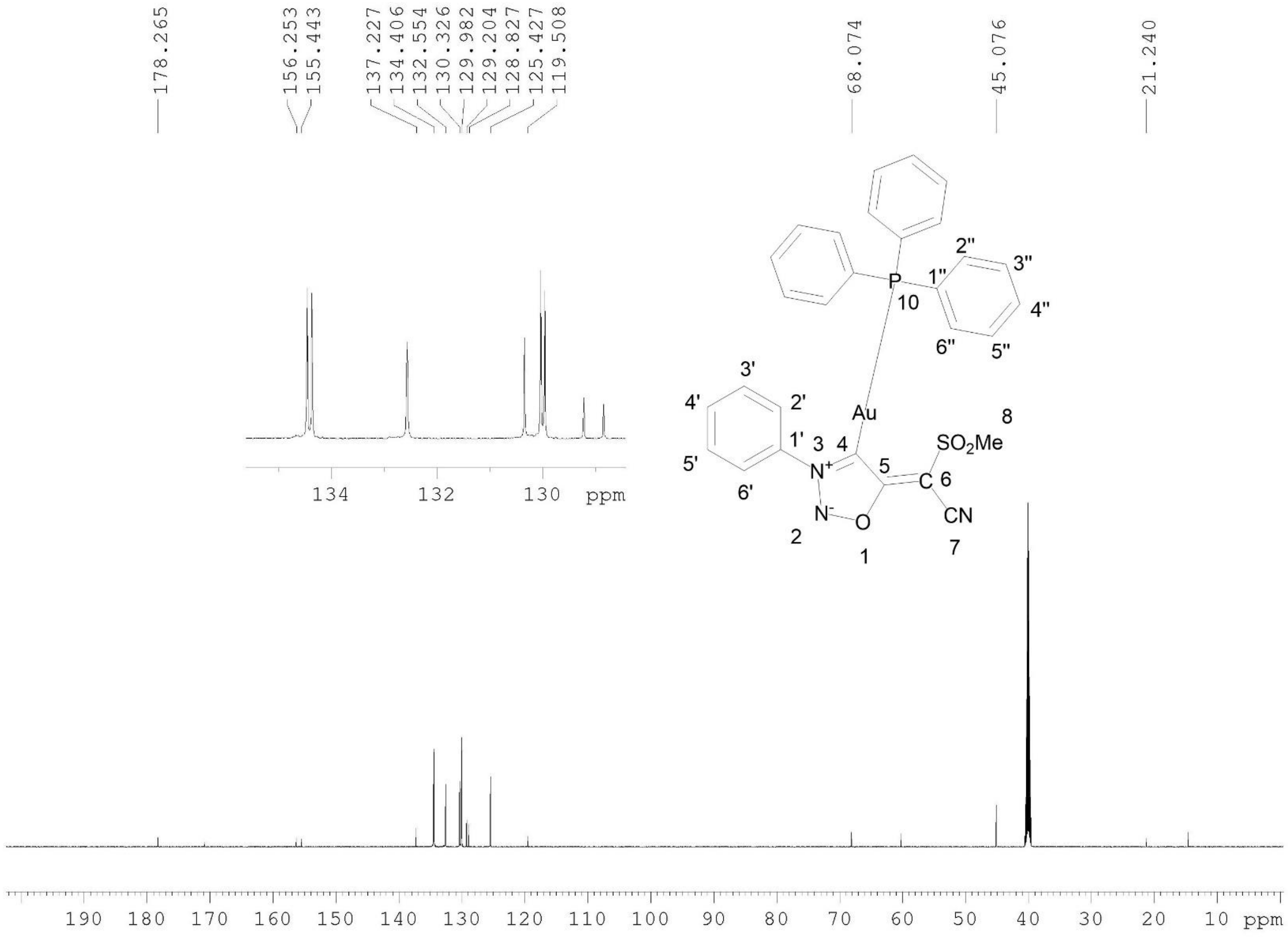

8c ^{13}C-NMR DEPT (150 MHz)

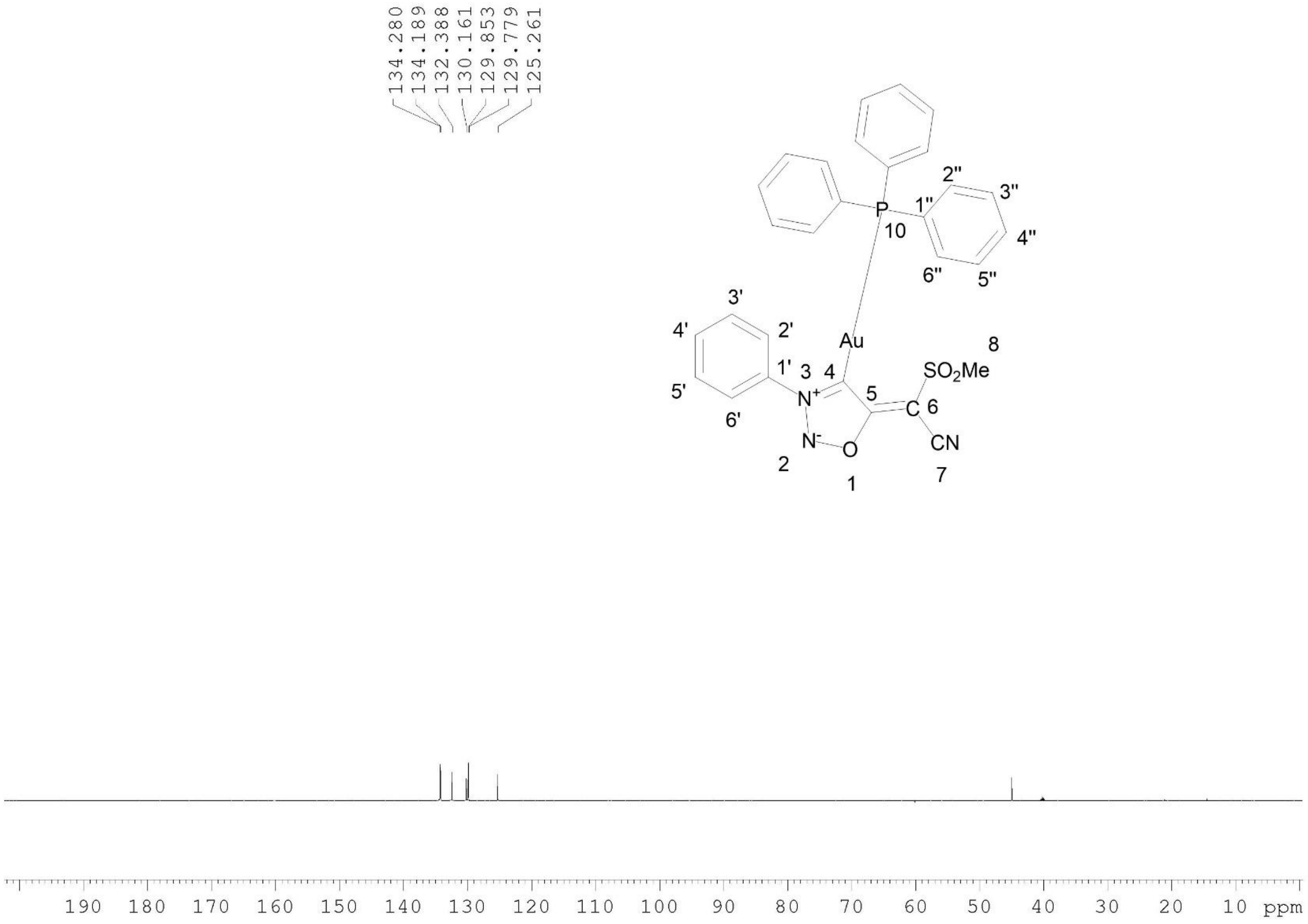

9a ^{1}H-NMR (600 MHz)

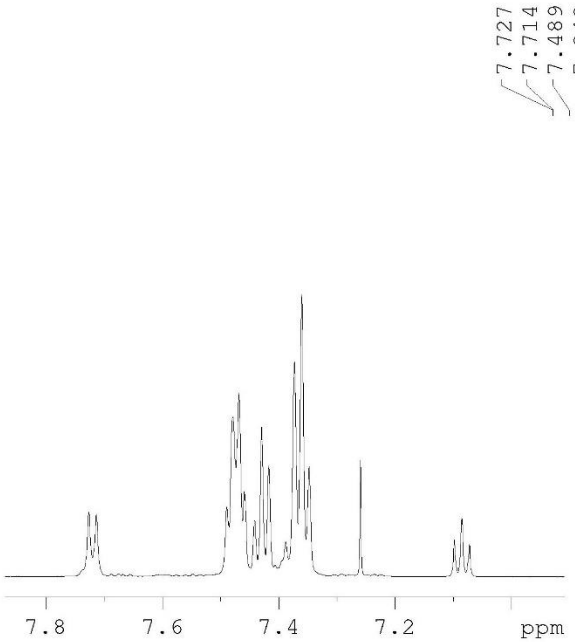

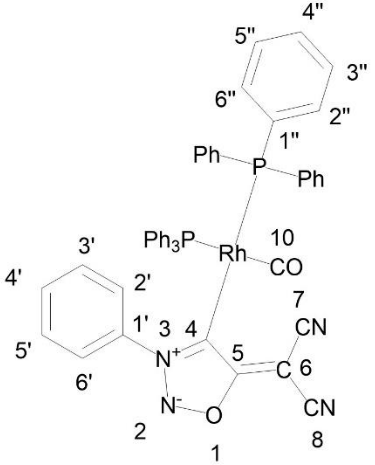

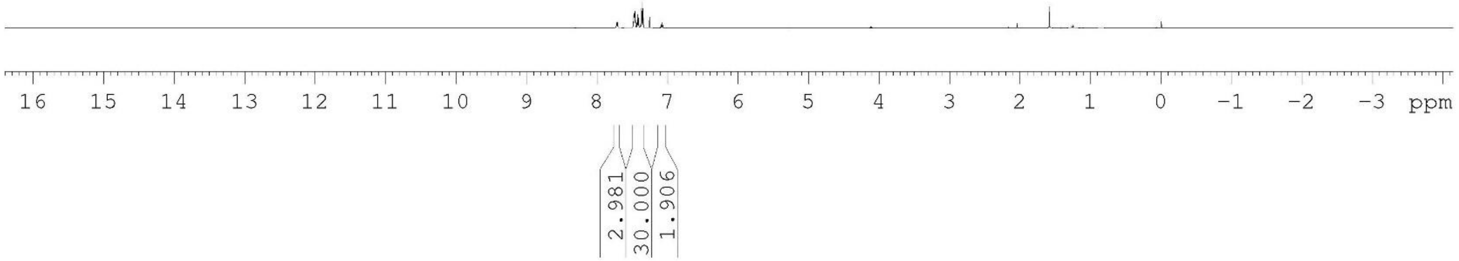

9a ^{13}C-NMR (150 MHz)

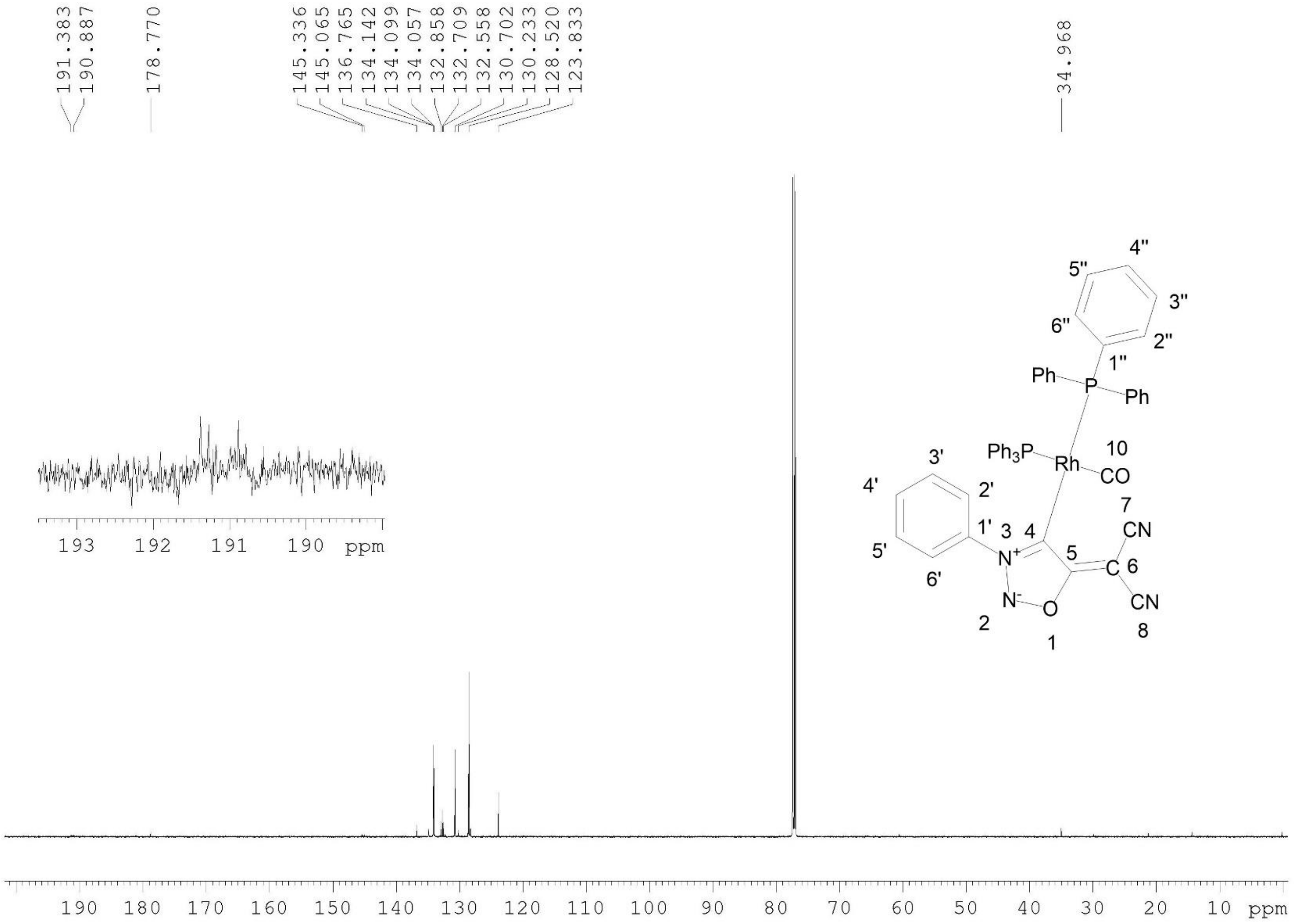

9a ^{13}C-NMR DEPT (150 MHz)

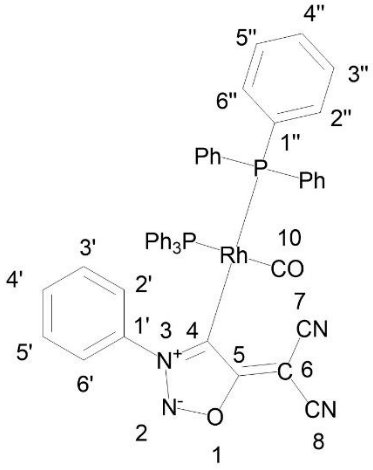

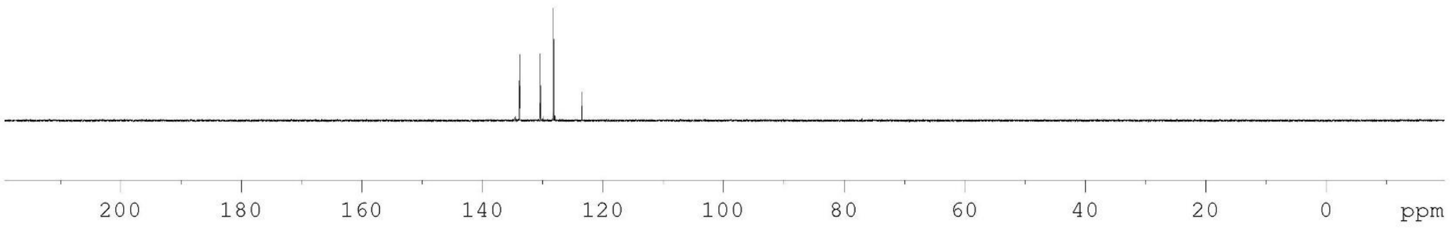

9b ^{1}H-NMR (600 MHz)

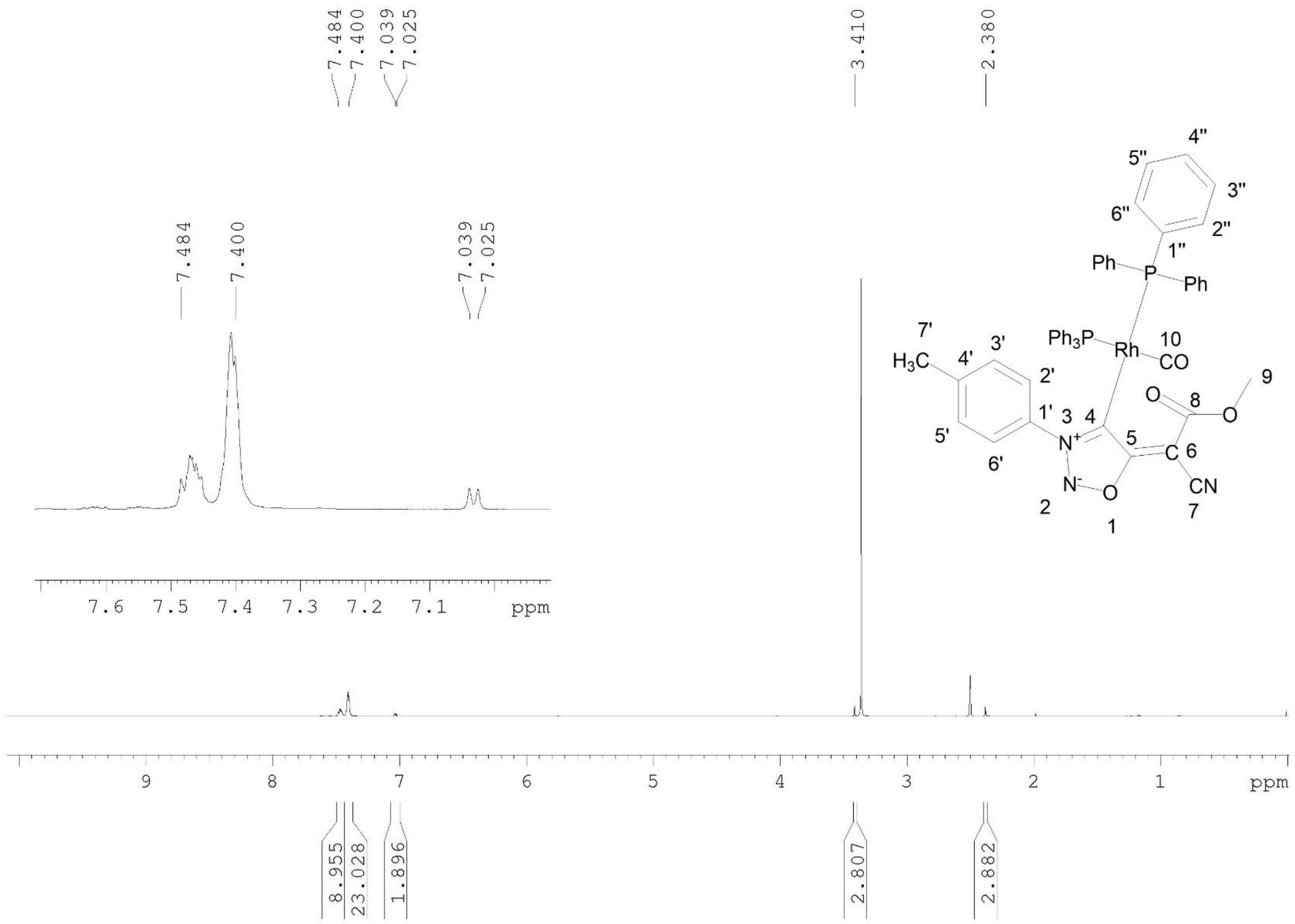

9b ^{13}C-NMR (150 MHz)

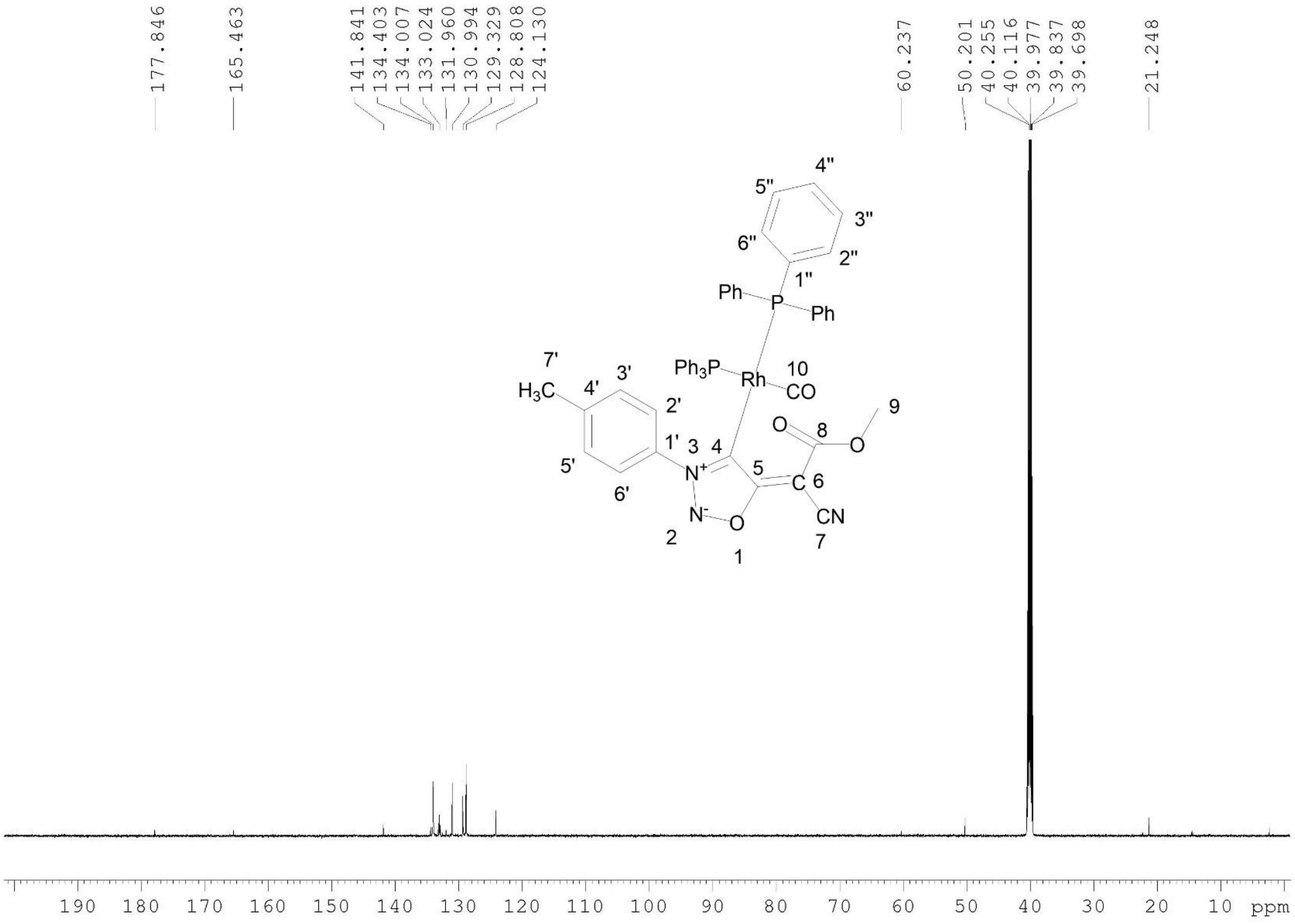

9b ^{13}C-NMR DEPT (150 MHz)

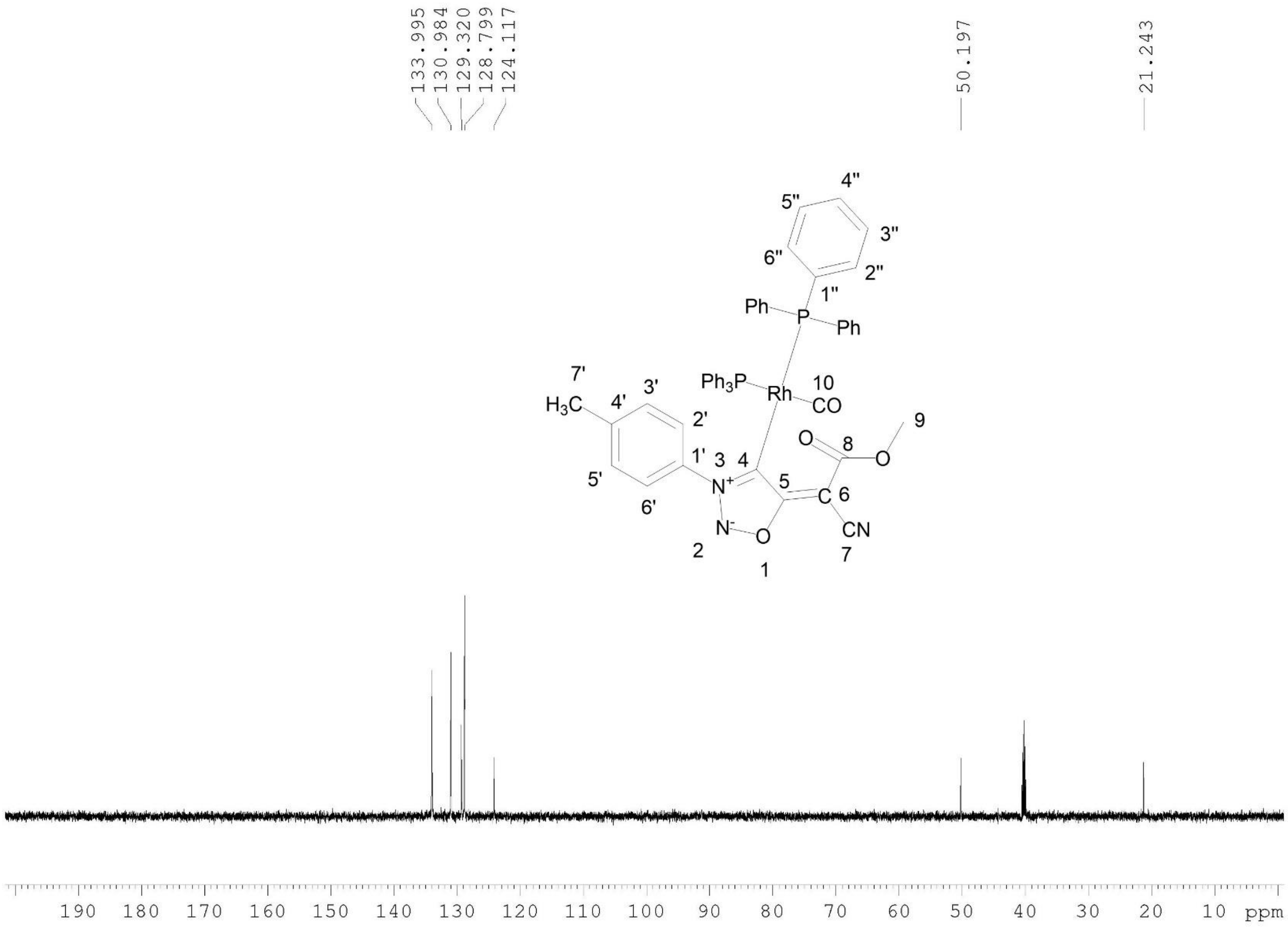

9c ^{1}H-NMR (600 MHz)

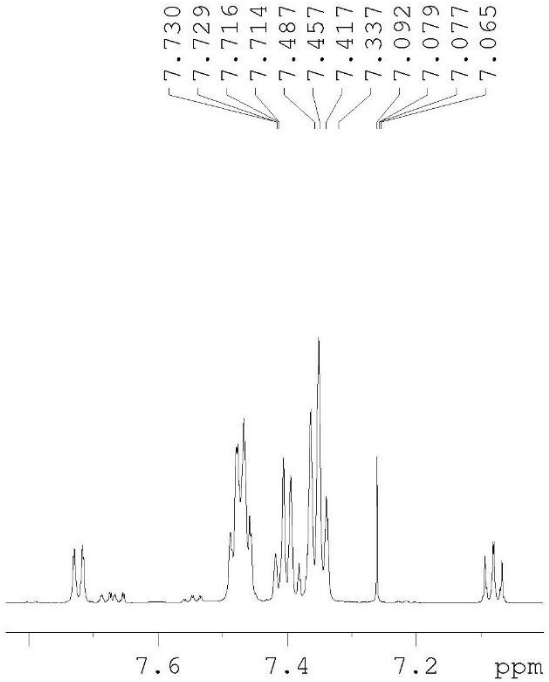

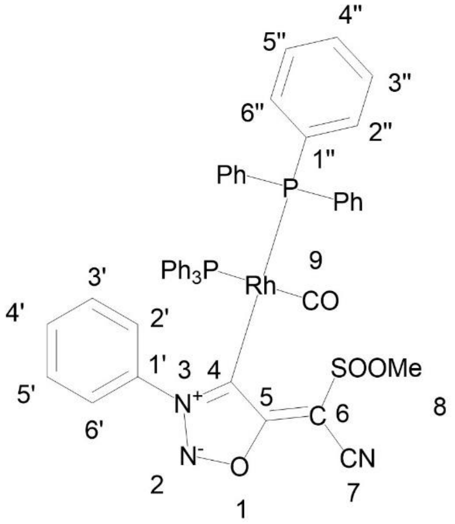

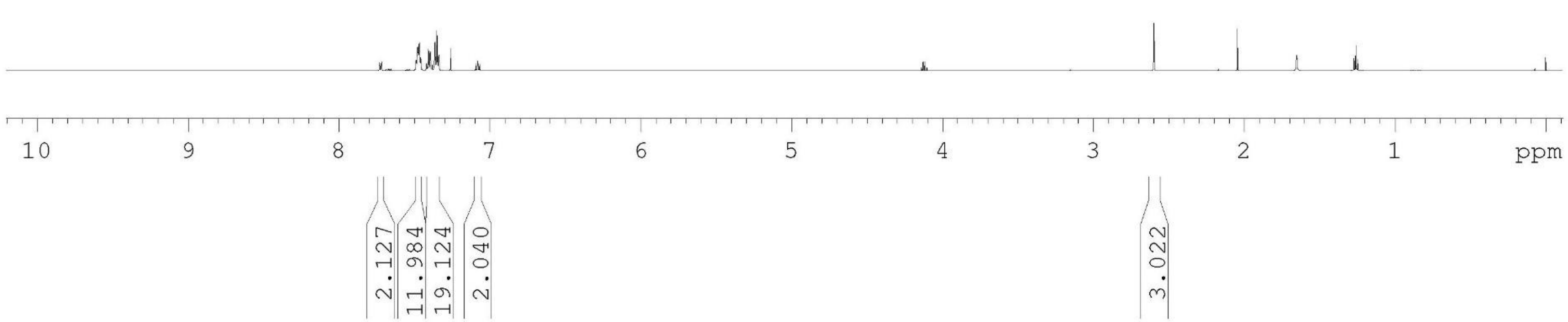

9c ^{13}C-NMR (150 MHz)

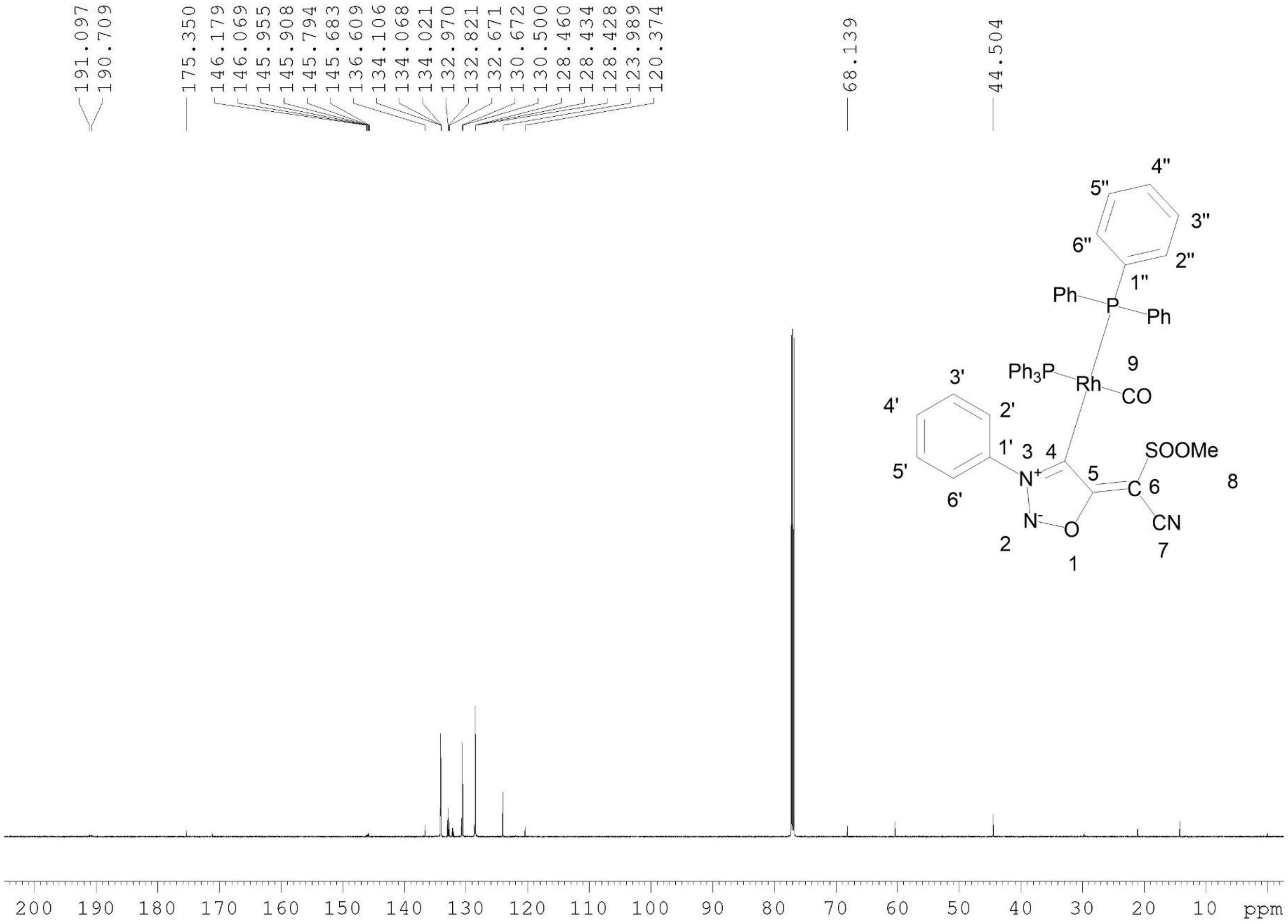

9c ^{13}C-NMR DEPT (150 MHz)

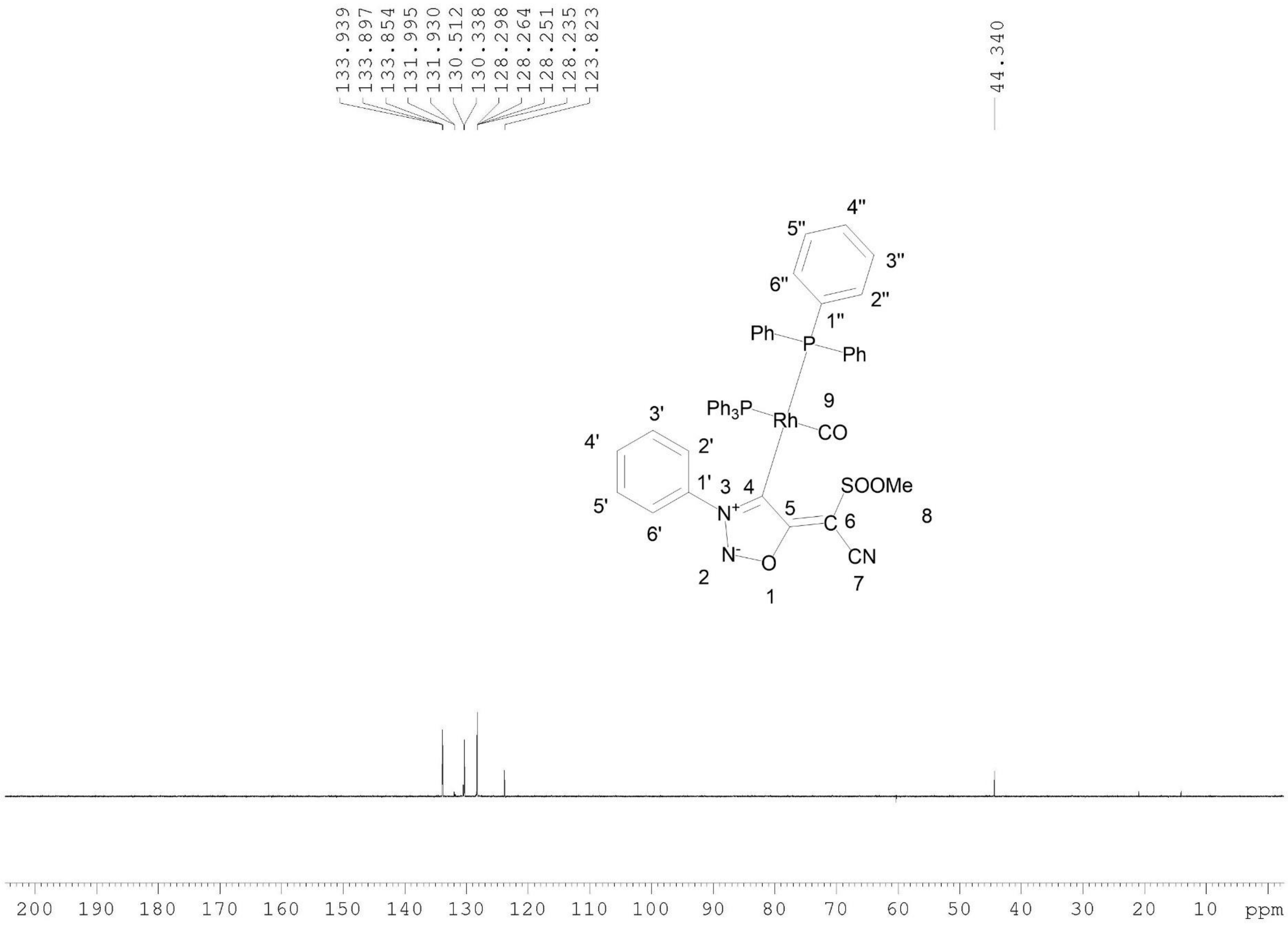

10a ^{1}H-NMR (600 MHz)

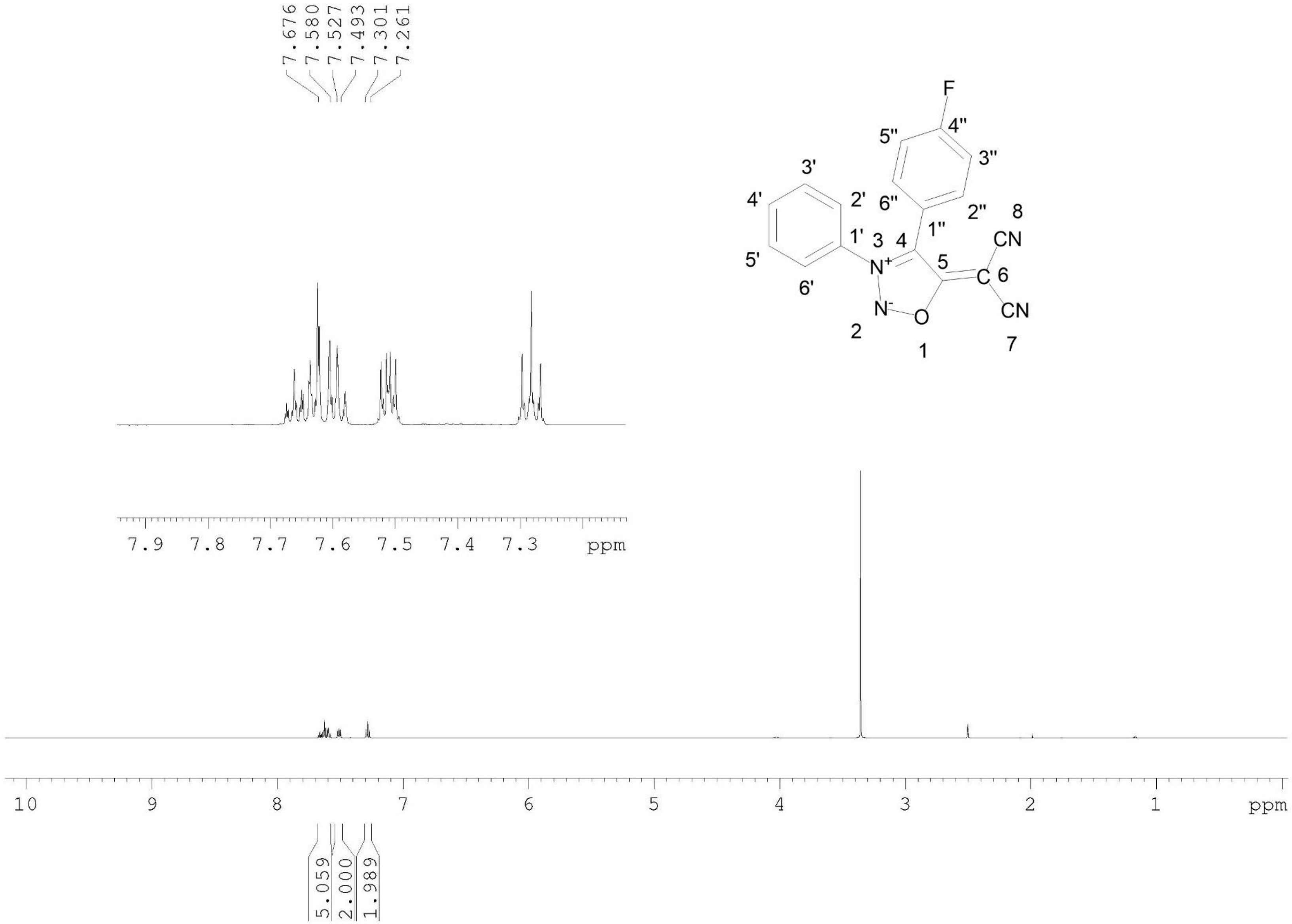

10a ^{13}C-NMR (150 MHz)

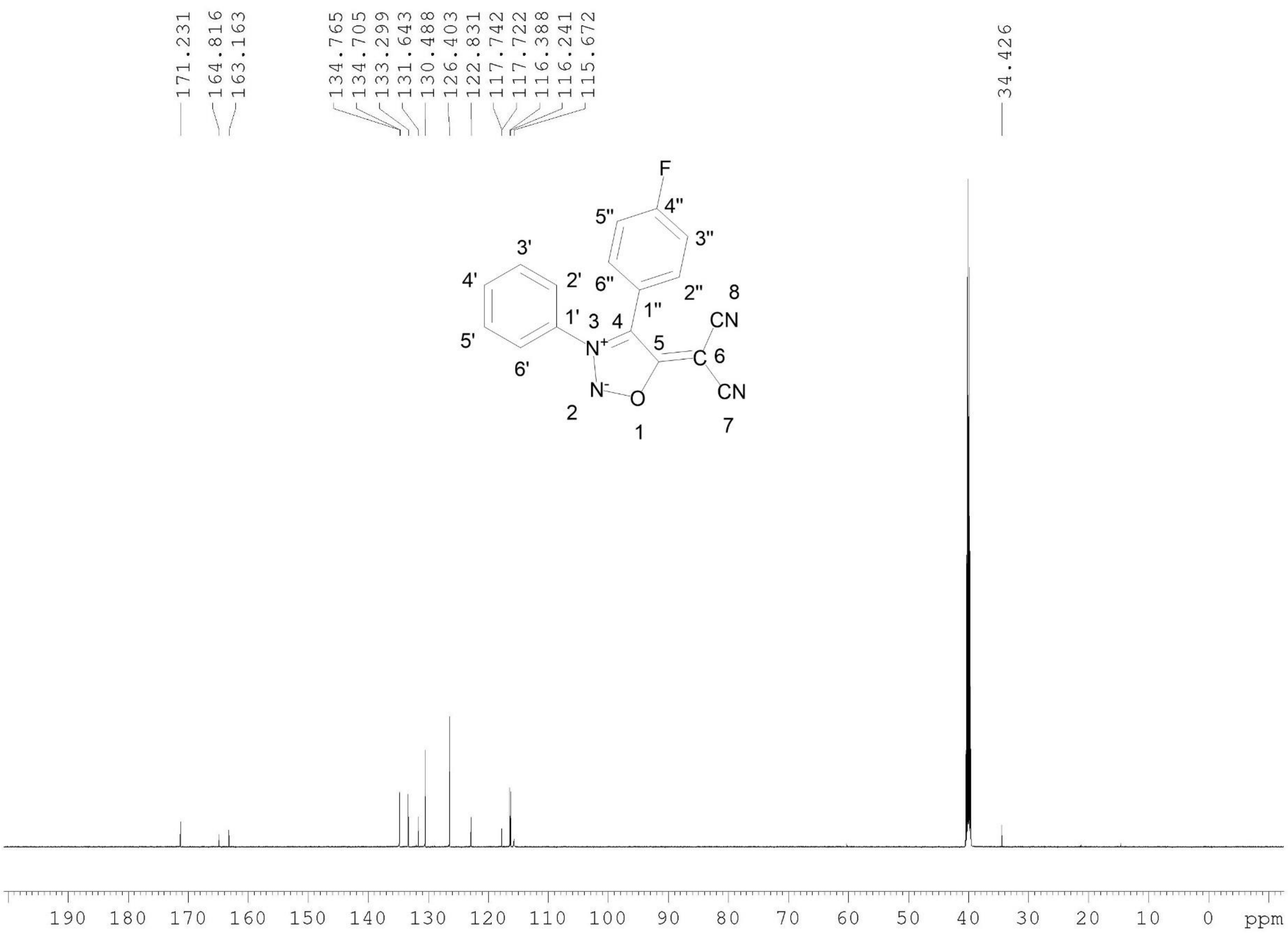

10a ^{19}F-NMR (376.5 MHz)

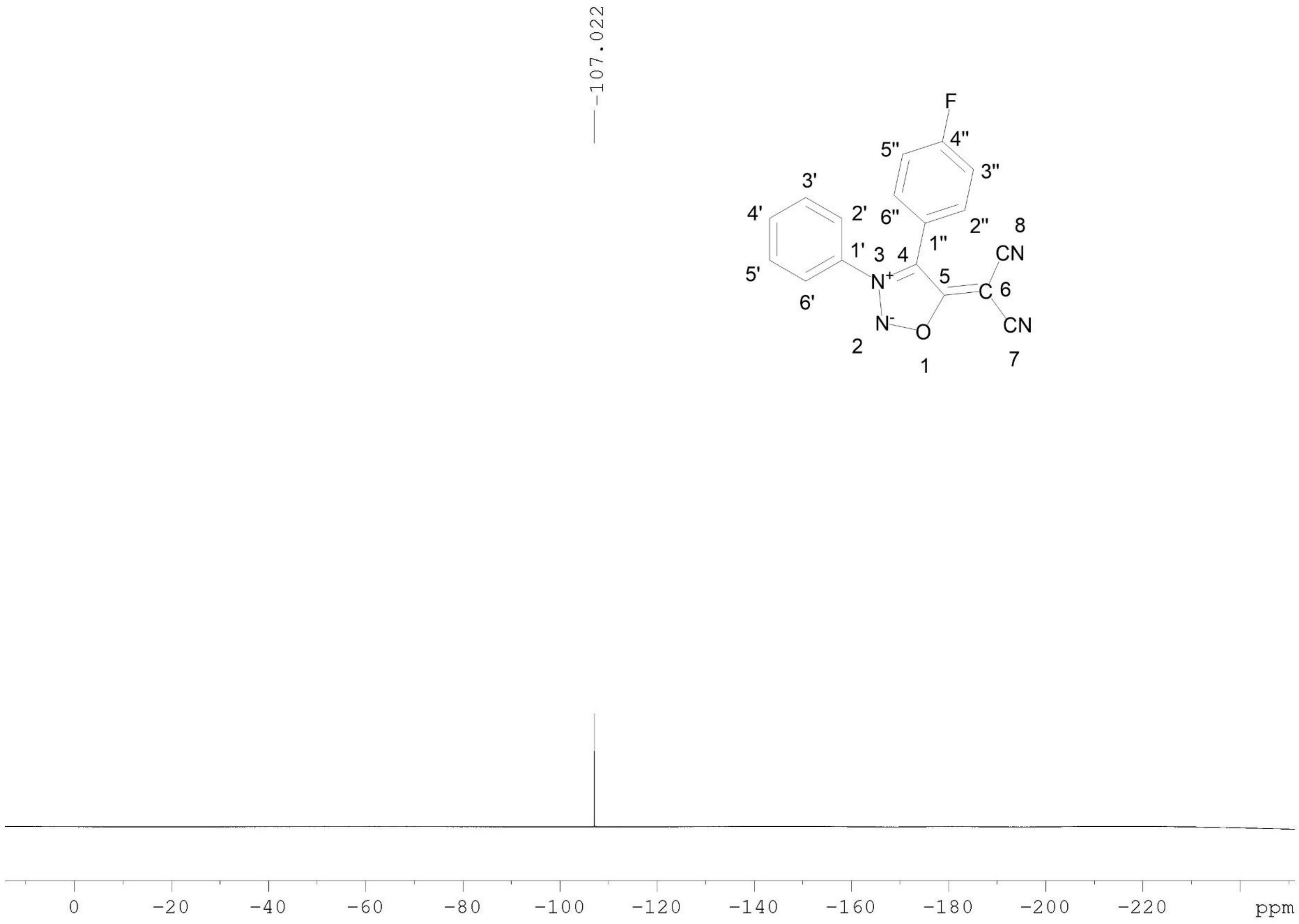

10a ^{13}C-NMR DEPT (150 MHz)

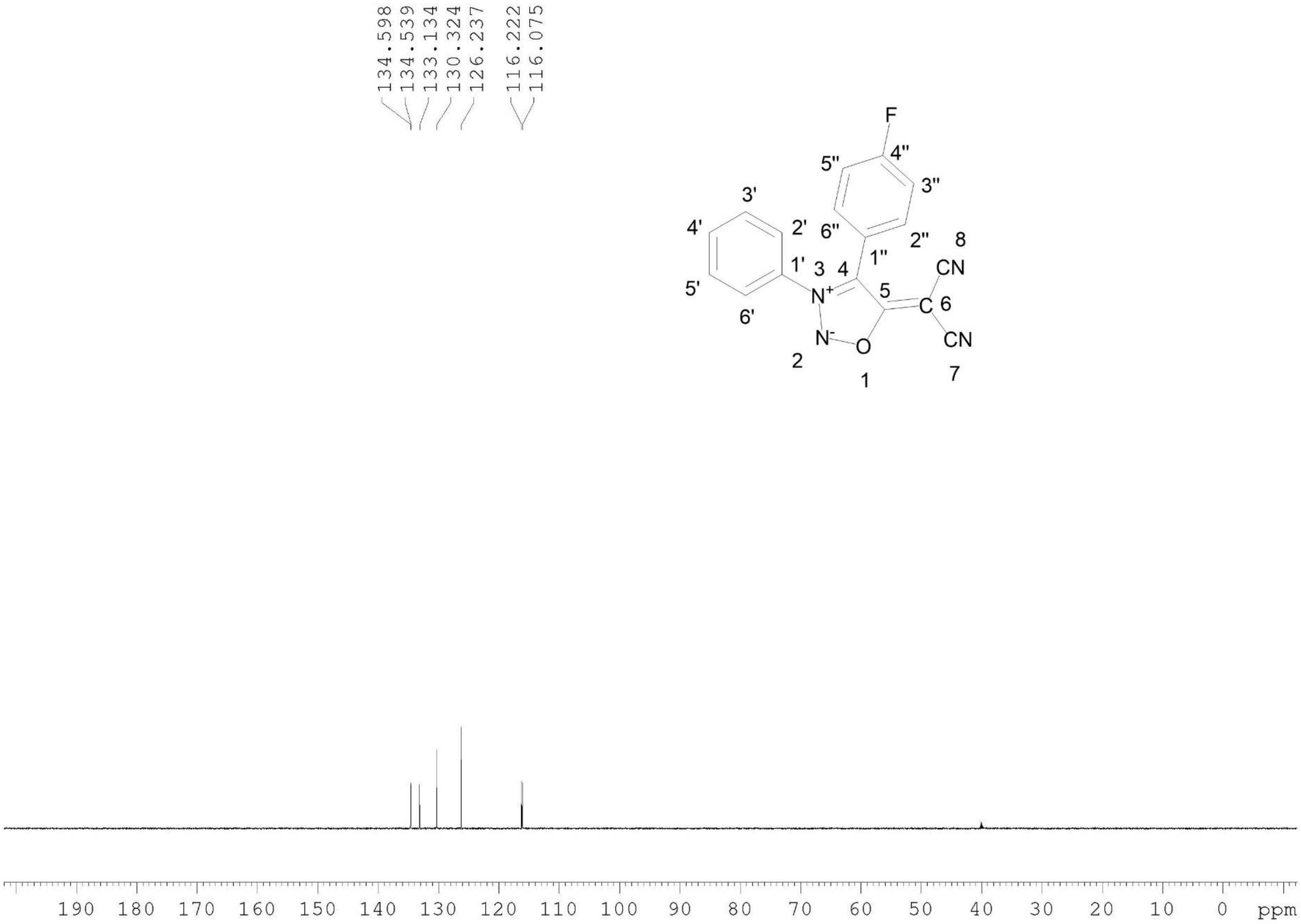

10b ^{1}H-NMR (600 MHz)

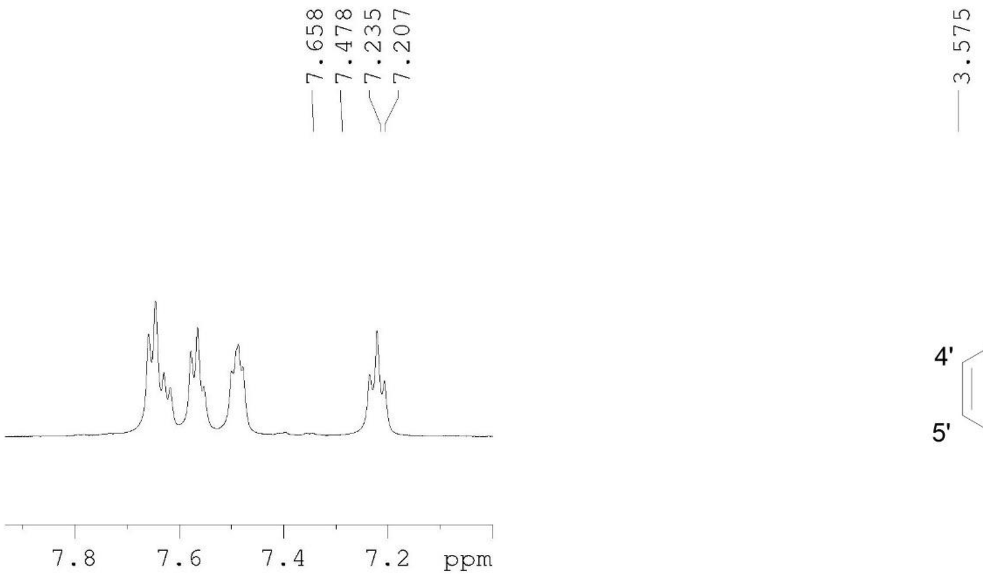

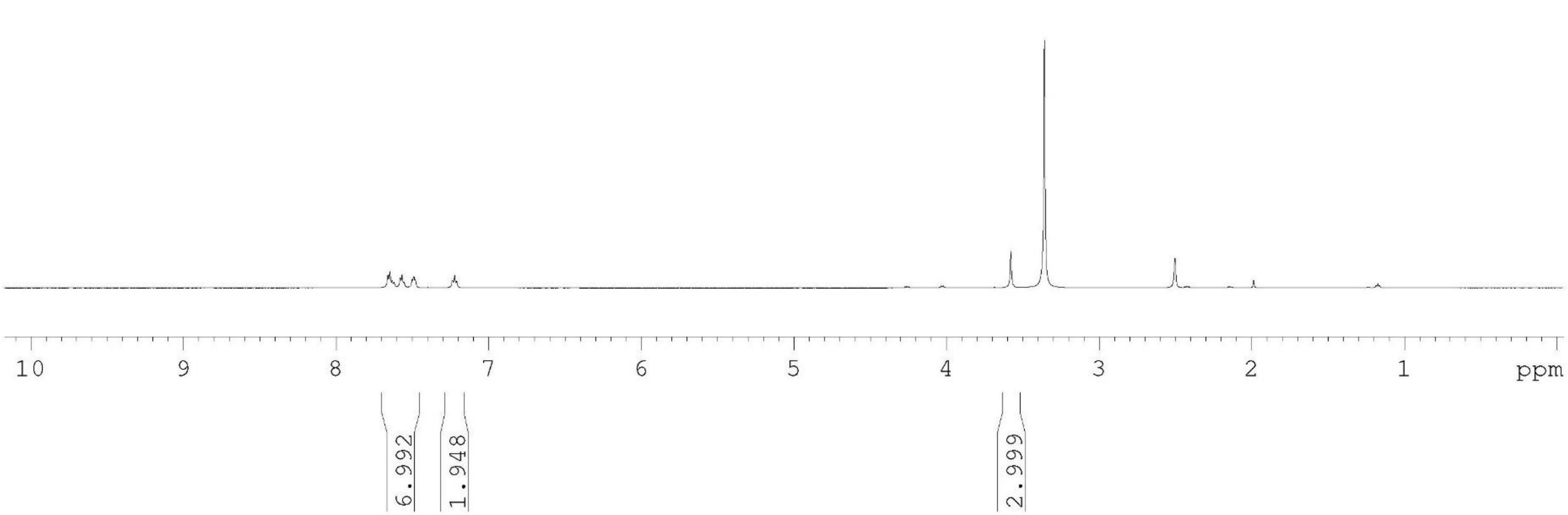

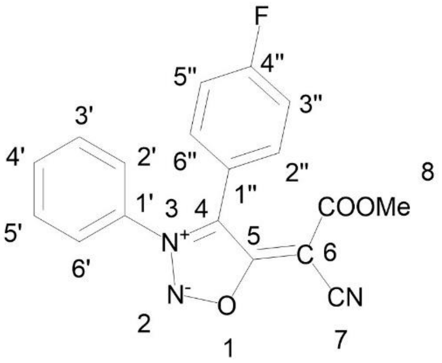

10b ^{13}C-NMR (150 MHz)

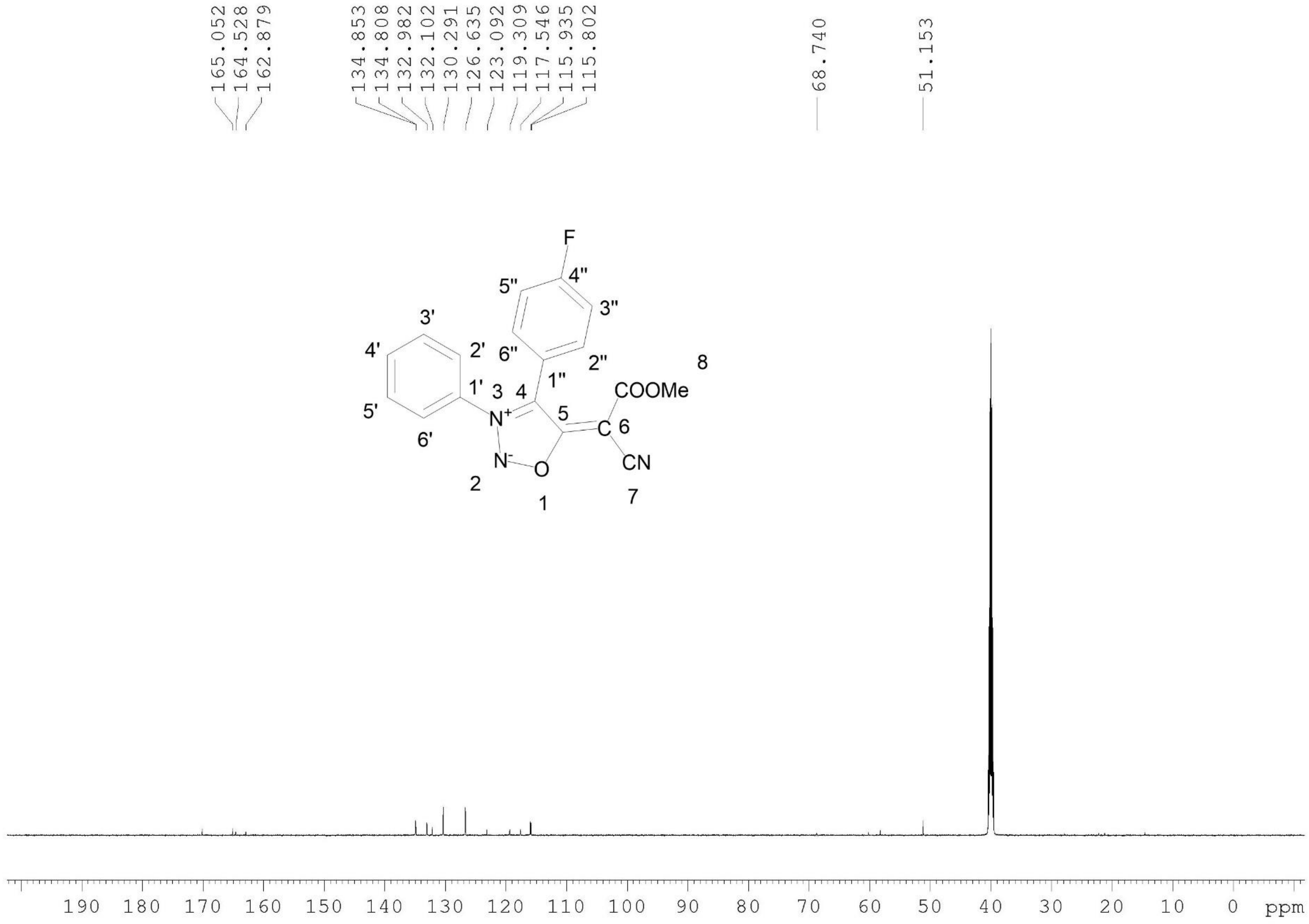

10b ^{19}F-NMR (376.5 MHz)

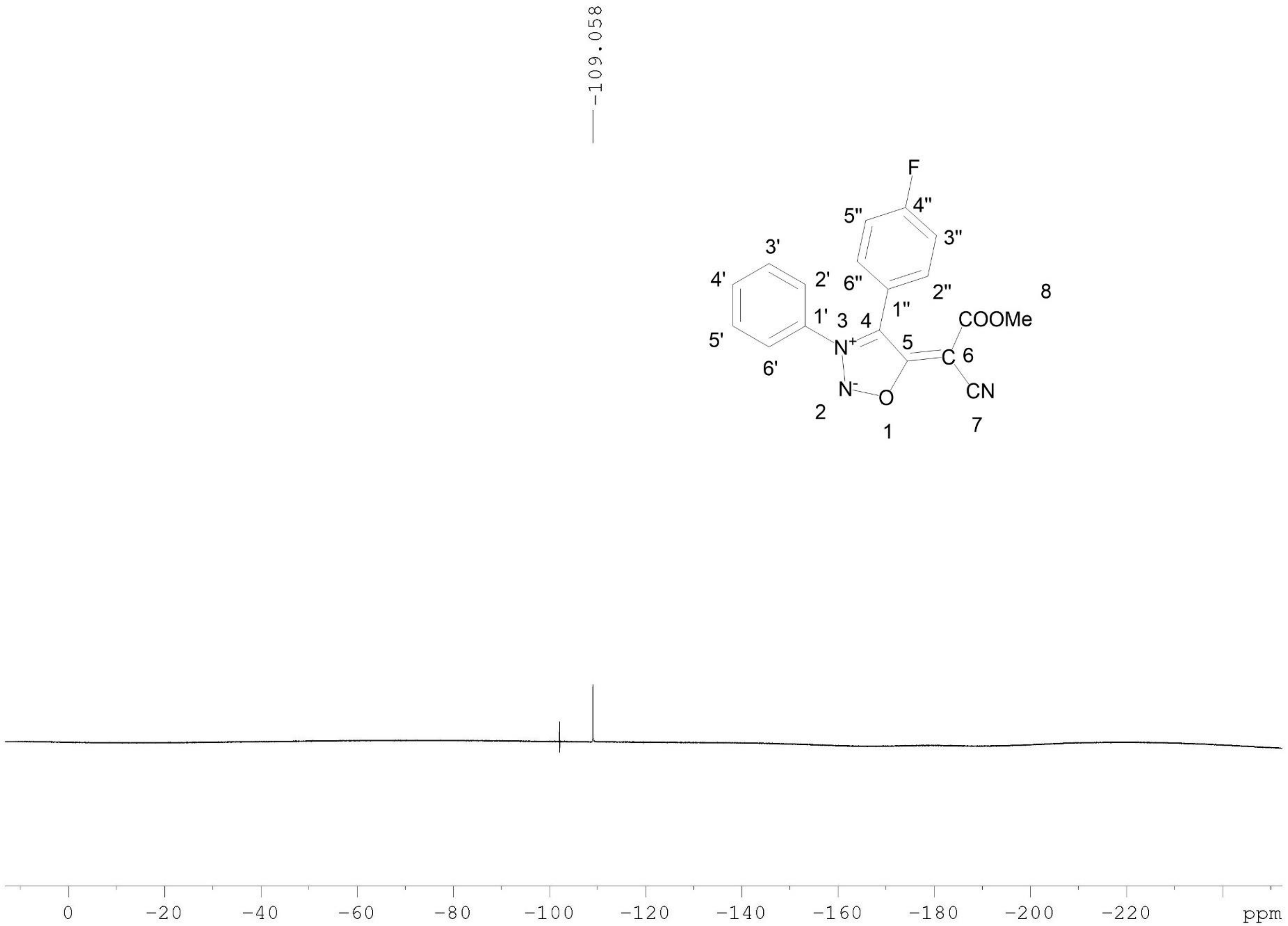

10b ^{13}C-NMR DEPT (150 MHz)

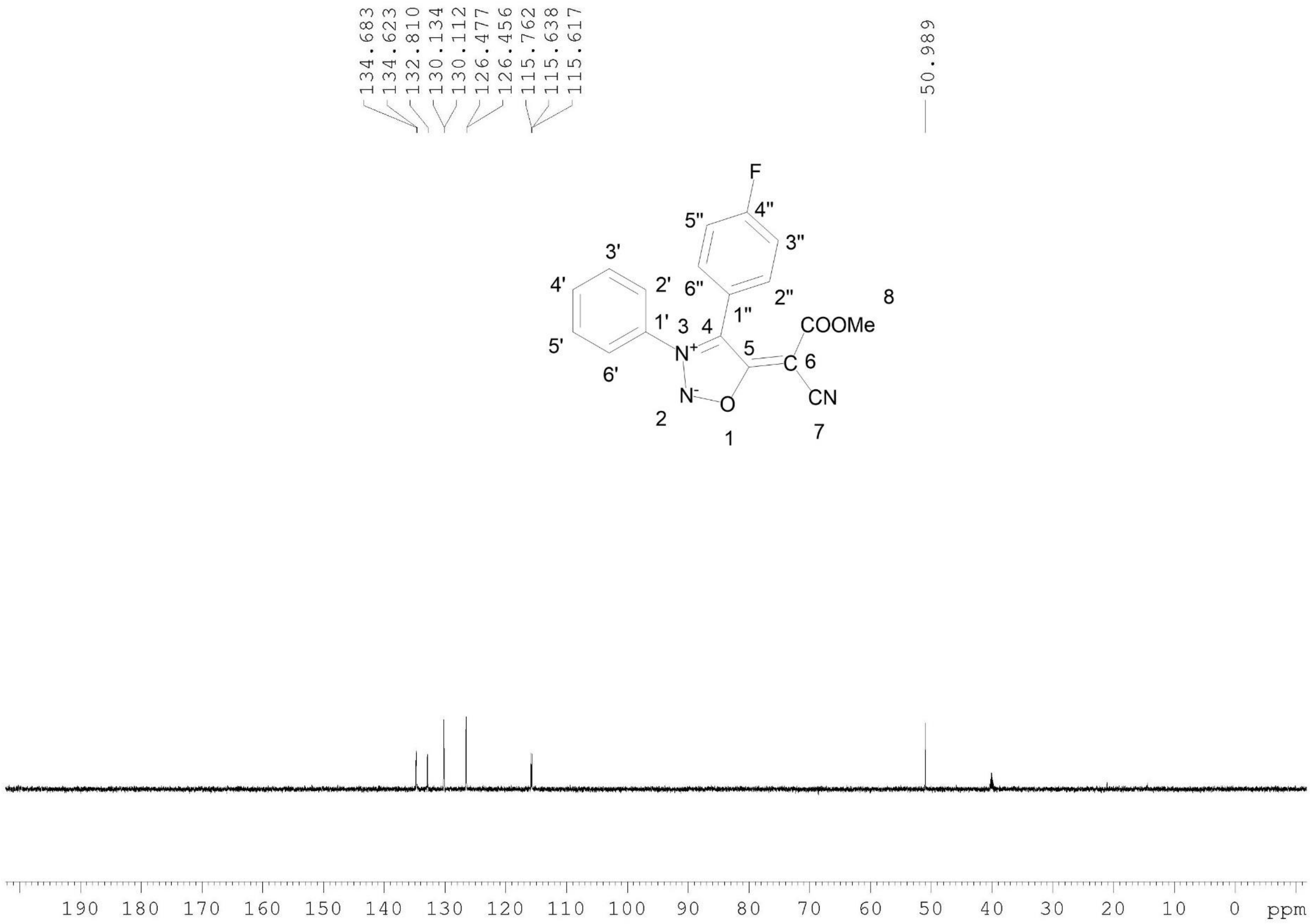

10c ^{1}H-NMR (600 MHz)

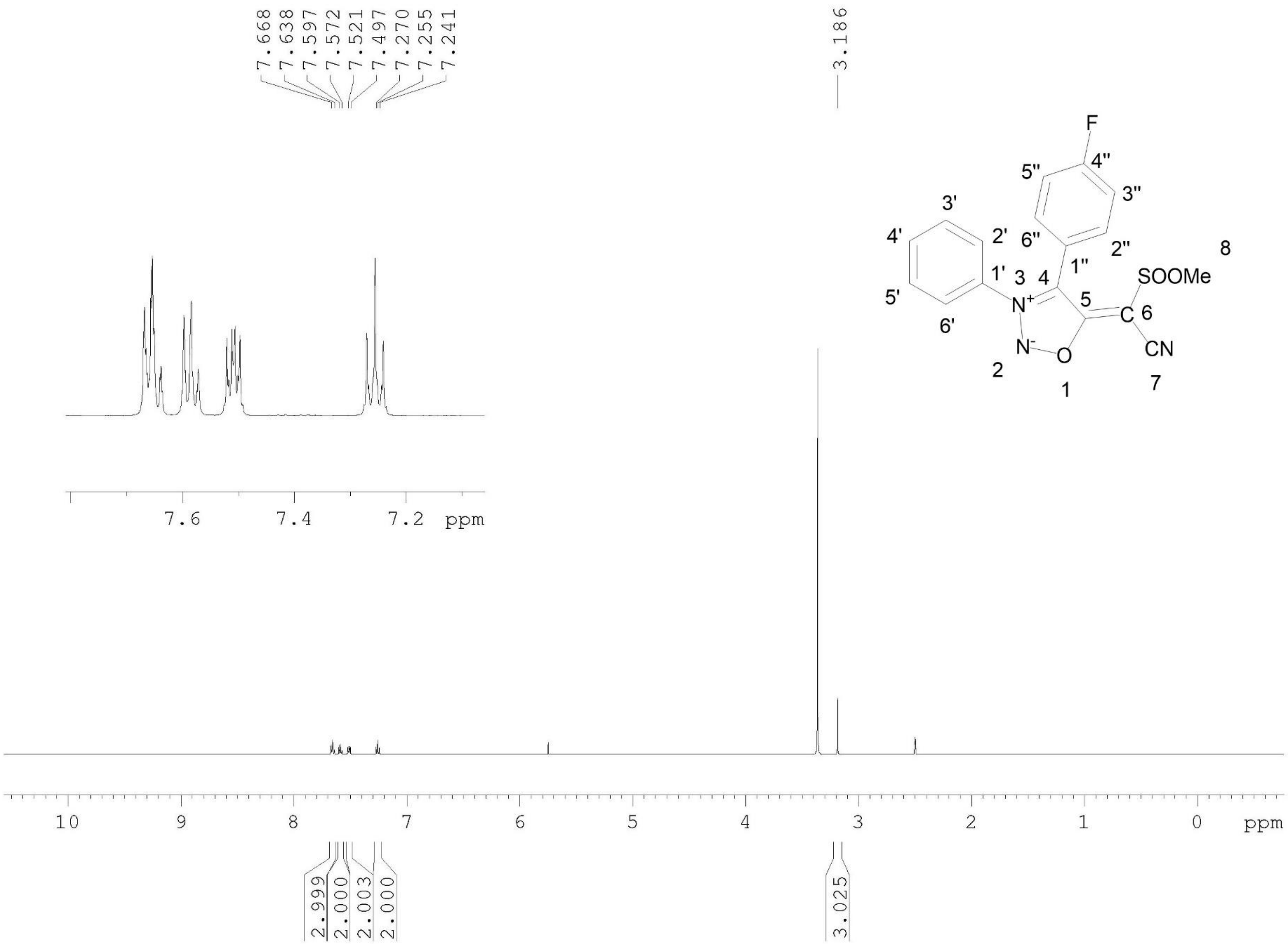

10c ^{13}C-NMR (150 MHz)

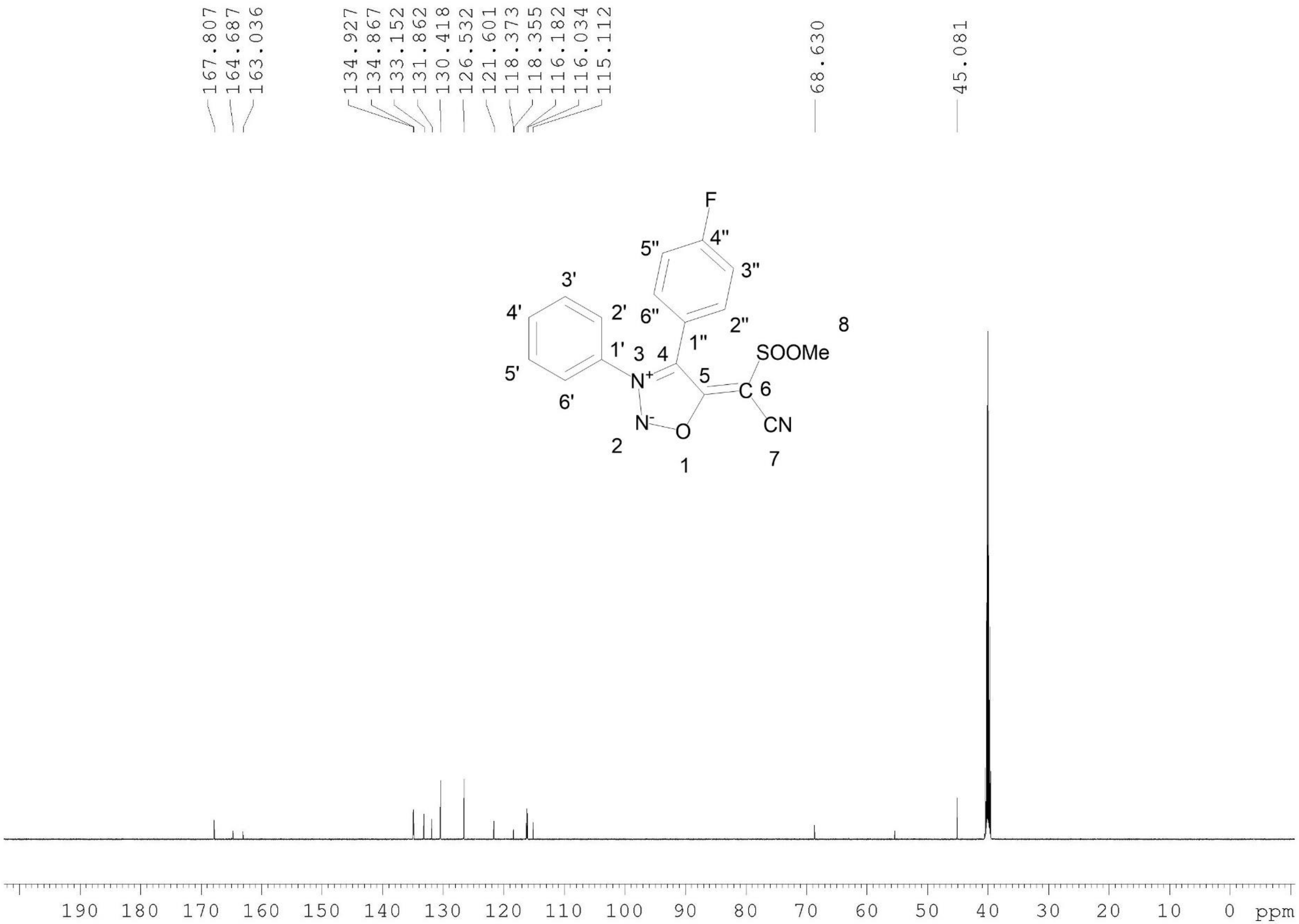

10c ^{19}F-NMR (376.5 MHz)

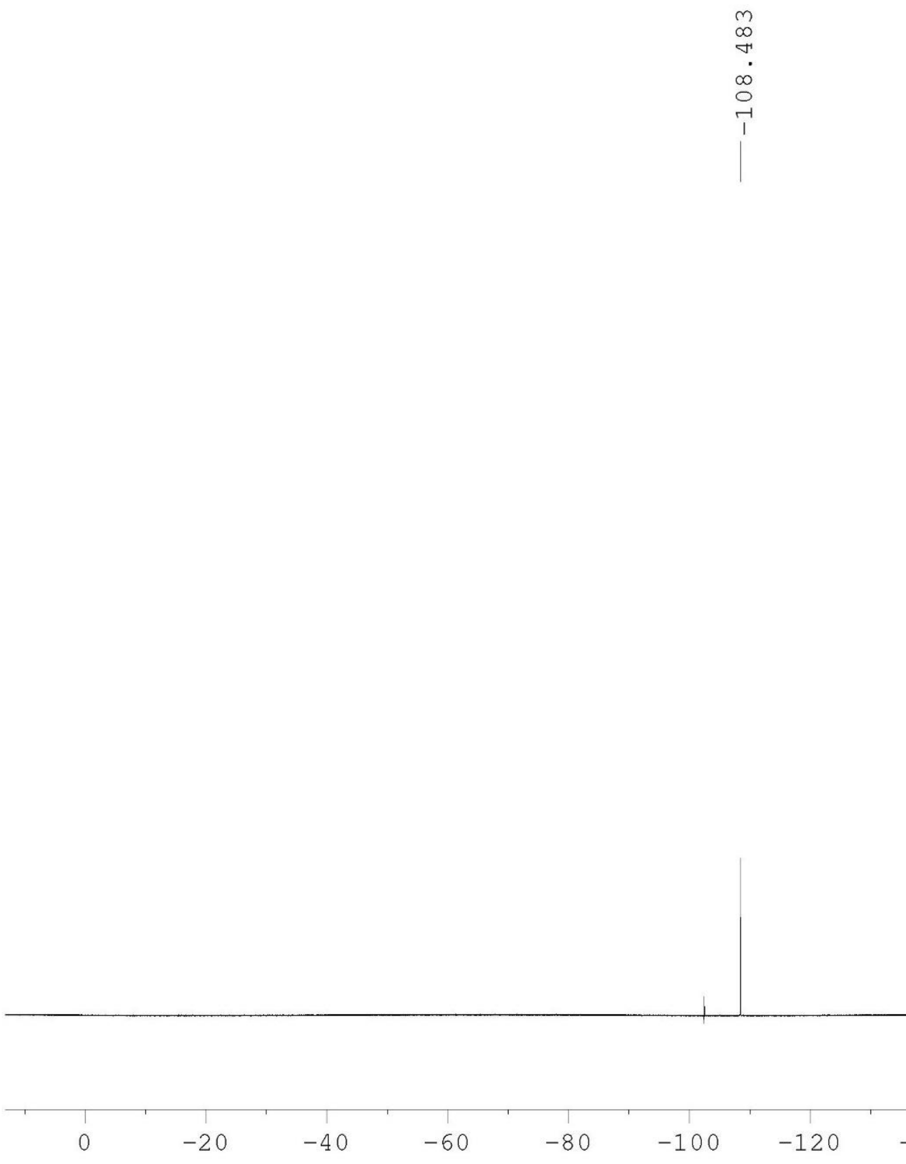

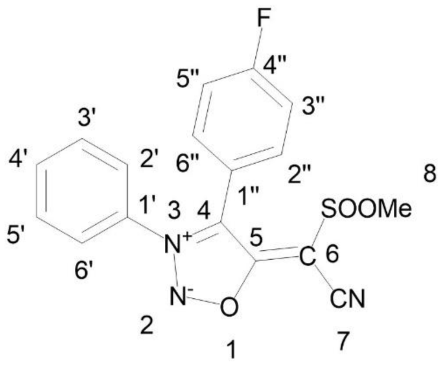

10c ^{13}C-NMR DEPT (150 MHz)

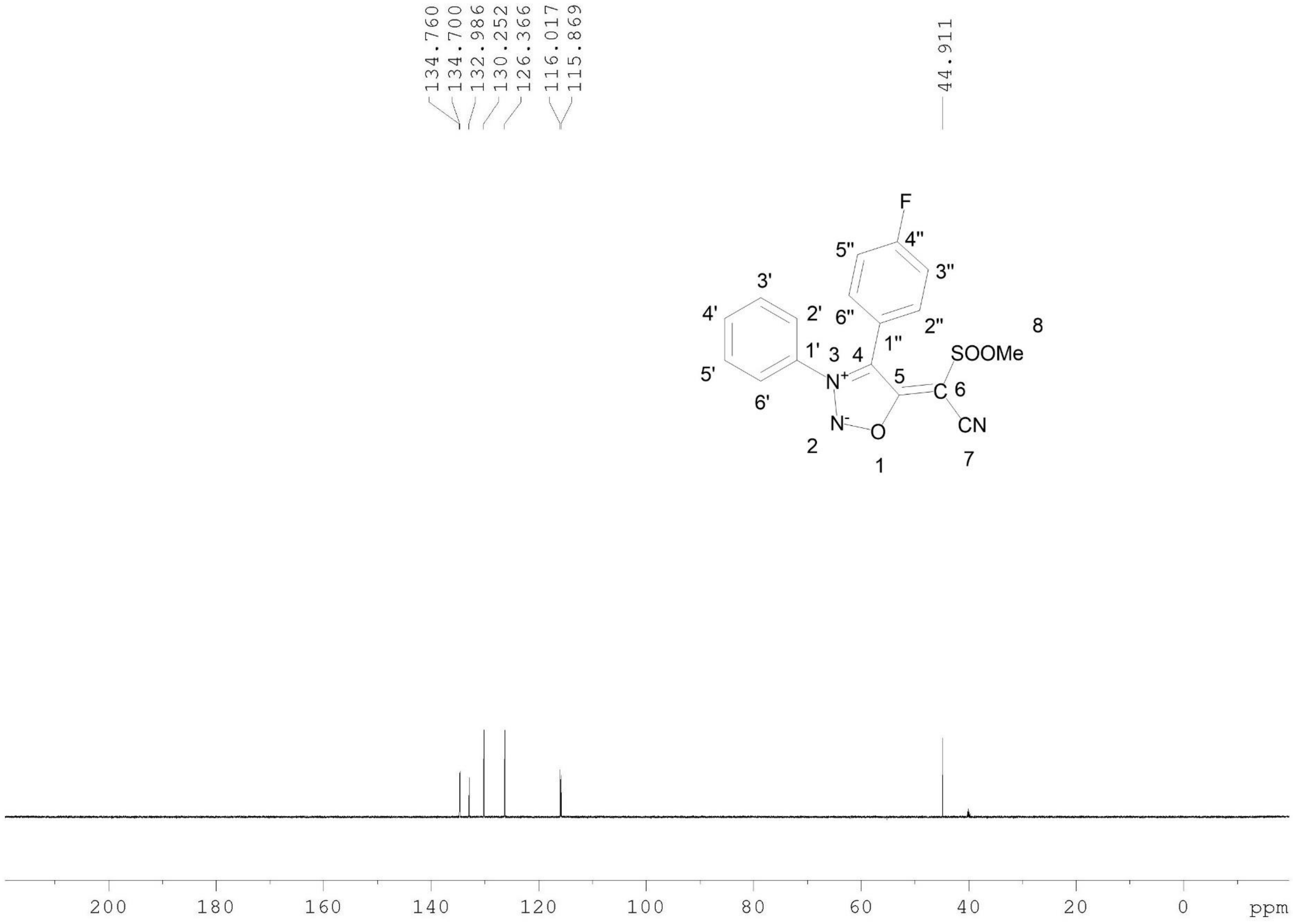

S5. Spectra of the anionic NHC formation: **3j ^{1}H-NMR (600 MHz)**

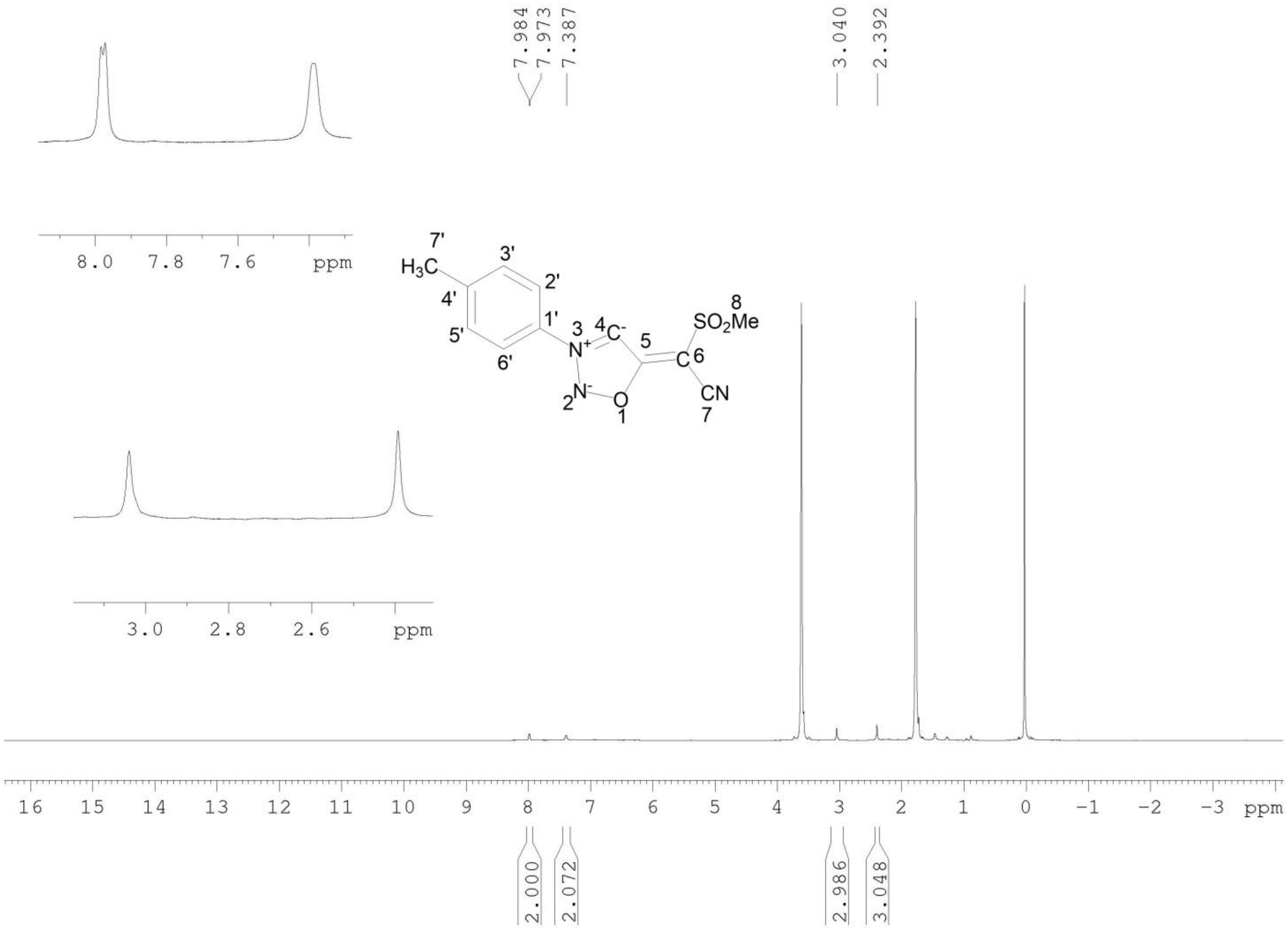

3j ^{13}C-NMR (150 MHz)

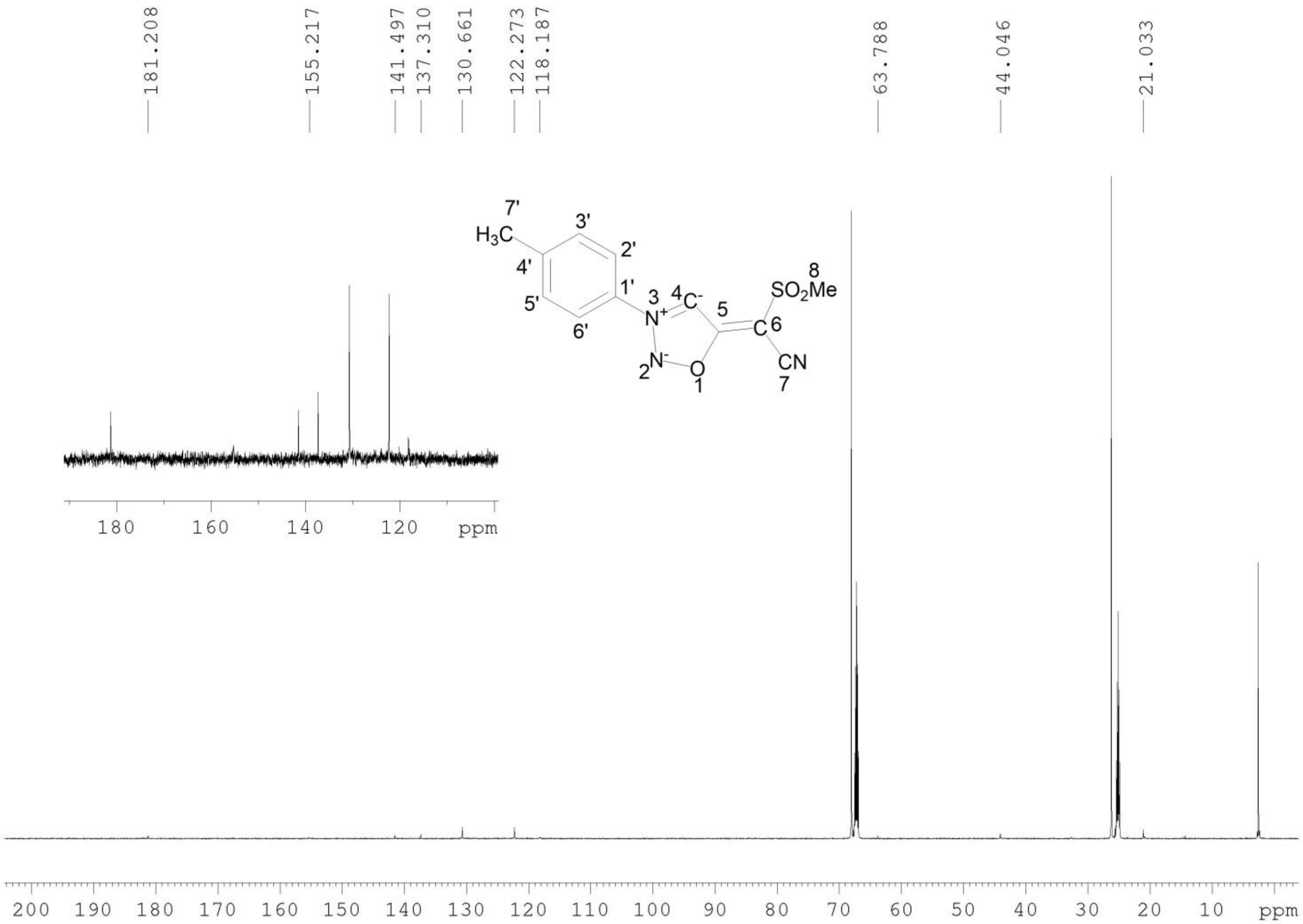

2j ^{1}H-NMR in deuterated THF

3j ^{1}H-NMR in deuterated THF

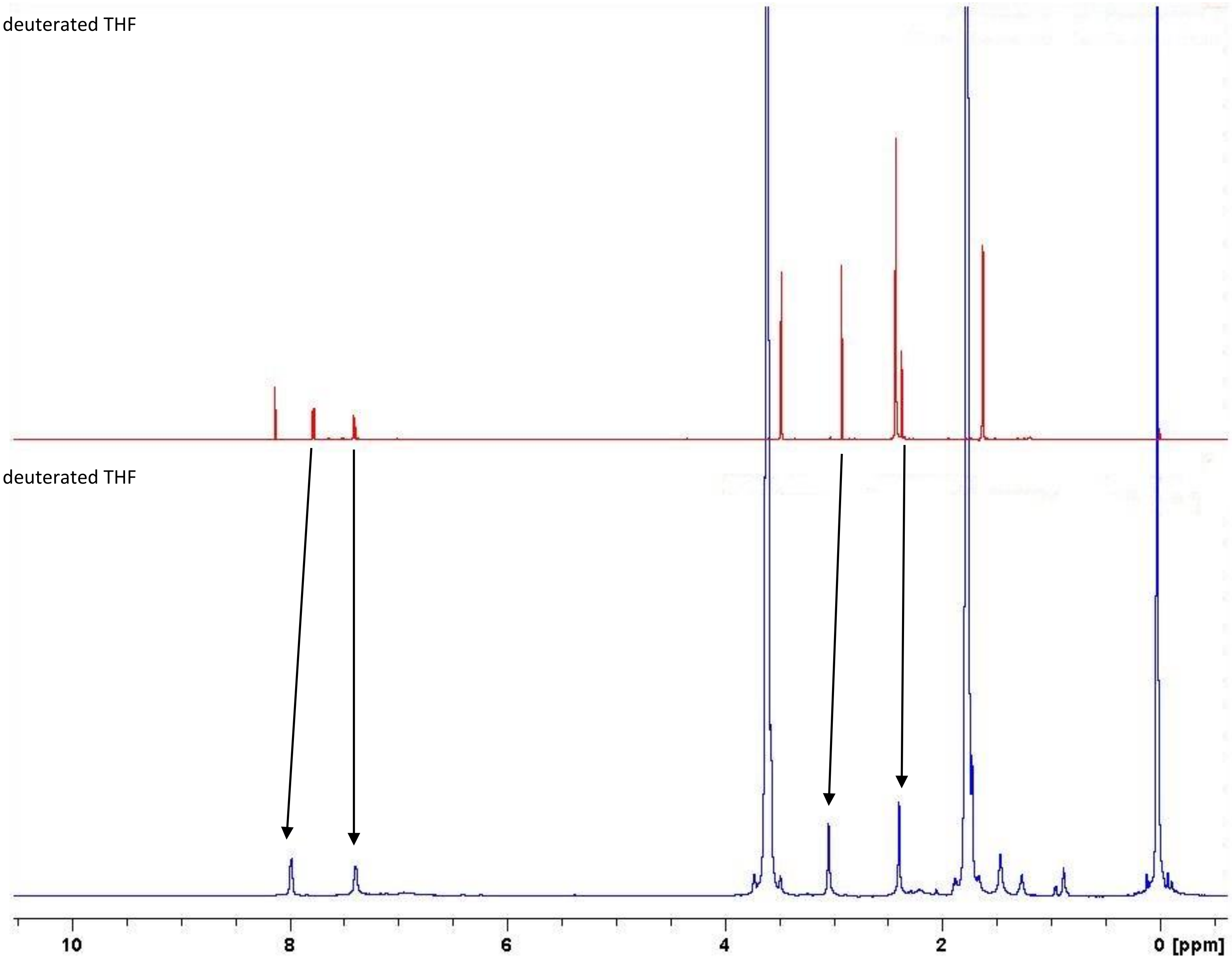

Comparison: 2j ^{13}C-NMR in deuterated THF

3j ^{13}C-NMR in deuterated THF

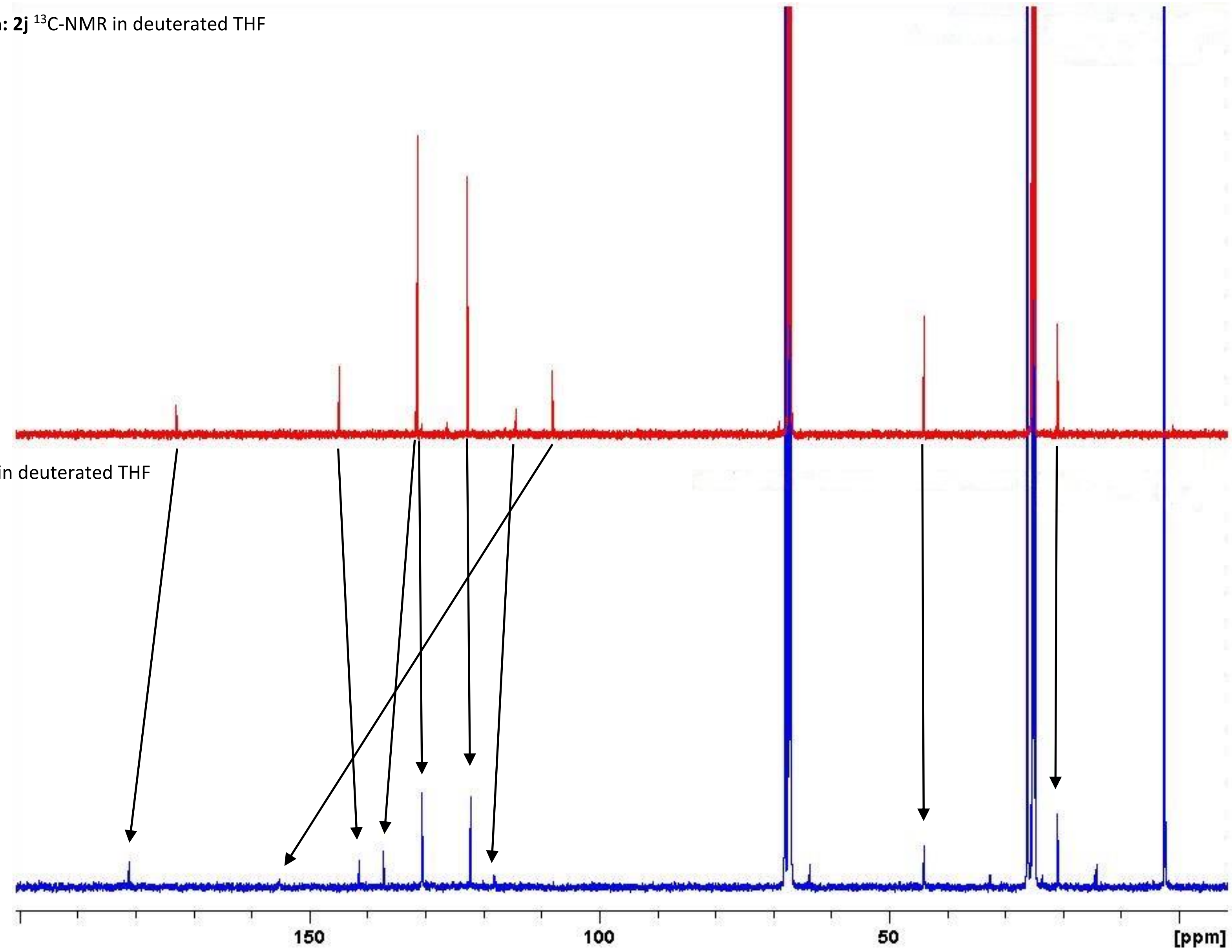

Table S2. ^{13}C NMR chemical shift differences on deprotonation of **2j** to give **3j.** Values in ppm.

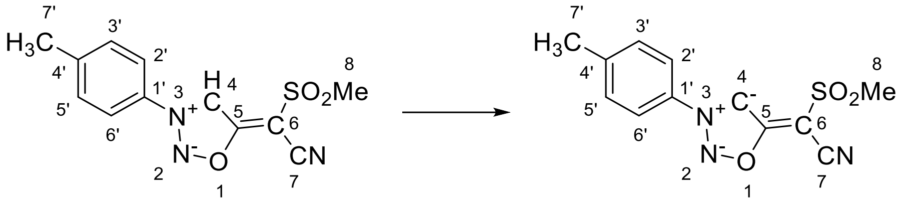

Carbon atom	Sydnone methide **2j**	Sydnone methide carbene **3j**
C-5	173.0	181.2
C-1‘	144.9	141.5
C-4‘	131.8	137.3
C-3‘ and C-5‘	131.4	130.7
C-2‘ and C-6‘	122.9	122.3
C-7	114.5	118.2
C-4	108.2	155.2
C-6	69.0	63.8
C-8	44.1	44.0
C-7‘	21.1	21.0

S6. Fluorescence spectra

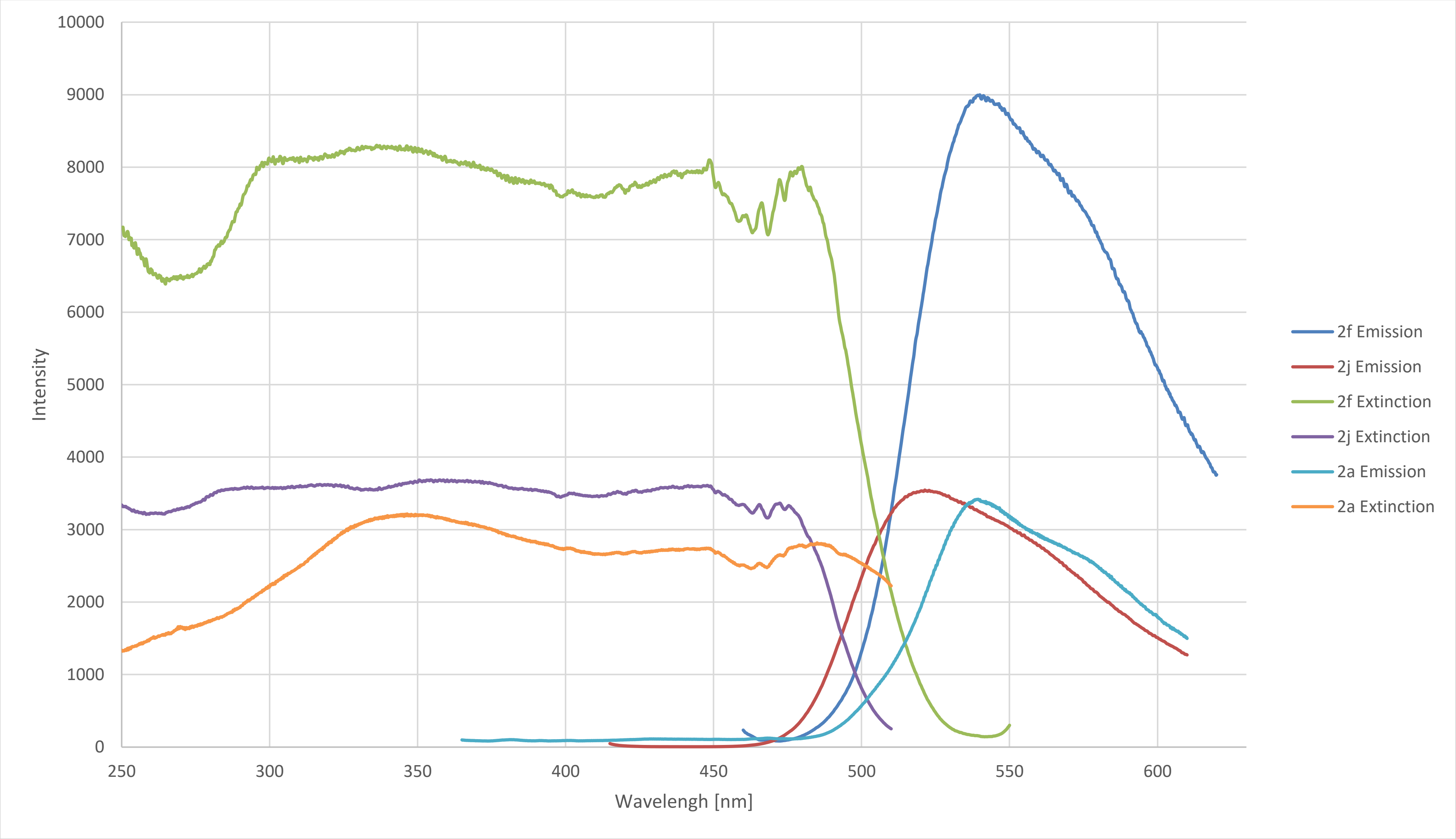

Supplement: Supplementary file 1 — Supporting Information [file ANIE-60-18882-s001.pdf]
